# Evidence for a Common Origin of Blacksmiths and Cultivators in the Ethiopian Ari within the Last 4500 Years: Lessons for Clustering-Based Inference

**DOI:** 10.1371/journal.pgen.1005397

**Published:** 2015-08-20

**Authors:** Lucy van Dorp, David Balding, Simon Myers, Luca Pagani, Chris Tyler-Smith, Endashaw Bekele, Ayele Tarekegn, Mark G. Thomas, Neil Bradman, Garrett Hellenthal

**Affiliations:** 1 University College London Genetics Institute (UGI), University College London, London, United Kingdom; 2 Centre for Mathematics and Physics in the Life Sciences and EXperimental Biology (CoMPLEX), University College London, London, United Kingdom; 3 Schools of BioSciences and of Mathematics & Statistics, University of Melbourne, Melbourne, Australia; 4 Department of Statistics, University of Oxford, Oxford, United Kingdom; 5 The Wellcome Trust Sanger Institute, Hinxton, United Kingdom; 6 Department of Archaeology and Anthropology, University of Cambridge, Cambridge, United Kingdom; 7 Addis Ababa University, Addis Ababa, Ethiopia; 8 Henry Stewart Group, London, United Kingdom; 9 Research Department of Genetics, Evolution and Environment, University College London, London, United Kingdom; University of Chicago, UNITED STATES

## Abstract

The Ari peoples of Ethiopia are comprised of different occupational groups that can be distinguished genetically, with Ari Cultivators and the socially marginalised Ari Blacksmiths recently shown to have a similar level of genetic differentiation between them (*F*_*ST*_ ≈ 0.023 − 0.04) as that observed among multiple ethnic groups sampled throughout Ethiopia. Anthropologists have proposed two competing theories to explain the origins of the Ari Blacksmiths as (i) remnants of a population that inhabited Ethiopia prior to the arrival of agriculturists (e.g. Cultivators), or (ii) relatively recently related to the Cultivators but presently marginalized in the community due to their trade. Two recent studies by different groups analysed genome-wide DNA from samples of Ari Blacksmiths and Cultivators and suggested that genetic patterns between the two groups were more consistent with model (i) and subsequent assimilation of the indigenous peoples into the expanding agriculturalist community. We analysed the same samples using approaches designed to attenuate signals of genetic differentiation that are attributable to allelic drift within a population. By doing so, we provide evidence that the genetic differences between Ari Blacksmiths and Cultivators can be entirely explained by bottleneck effects consistent with hypothesis (ii). This finding serves as both a cautionary tale about interpreting results from unsupervised clustering algorithms, and suggests that social constructions are contributing directly to genetic differentiation over a relatively short time period among previously genetically similar groups.

## Author Summary

While it is widely recognized that DNA patterns vary across world-wide human populations, the primary features that drive these differences are less well understood. As an example, the Ari peoples of Ethiopia are presently socially divided according to occupation, with Ari Blacksmiths marginalised relative to Ari Cultivators. Two competing theories proposed by anthropologists to explain the existence of these occupational groupings suggest very different histories: (i) the Cultivators reflect migrants who moved into the region occupied by ancestors of the Blacksmiths perhaps many thousands of years ago, versus (ii) the Blacksmiths and Cultivators comprised the same ancestral group before the former was marginalised due solely to their trade. Recent genetic studies showed that Blacksmiths and Cultivators are distinguishable by their DNA, and suggested that overall DNA patterns among the two groups were consistent with (i). However, we demonstrate here that interpreting the results of currently popular algorithms that compare DNA is not always straight-forward. Instead we use a variety of analyses to show that (ii) seems a more likely explanation, perhaps illustrating how social marginalisation can lead to groups becoming genetically distinguishable over a relatively short time period.

## Introduction

Different ethnic groups in present-day Ethiopia show a substantial amount of cultural [1] and genetic [2] diversity. Some of this diversity falls along societal divisions, e.g. across distinct groups that are segregated through social barriers to interaction and co-operation [1]. Marginalised groups are largely comprised of craft workers (artisans) and hunters [3]. For example, the Ari Cultivators, who are farmers, are said to have limited interaction with the Ari Blacksmiths, who specialize in iron and wood-work and live on the periphery of settlements [4]. Blacksmithing communities are widely regarded as the most marginalised of artisan groups, not just within the Ari but throughout southern Ethiopia [3].

Two alternative hypotheses proposed by anthropologists to explain the origin of marginalised groups in Ethiopia, such as are present in the Ari community, imply very different ancestral histories [1, 3]:

- Remnants model (RN)—Under the Remnants model, originally proposed by Biasutti (1905) [5, 1], the Ari Blacksmiths are designated as an early, possibly hunter-gatherer group which existed in Ethiopia prior to the arrival of farmers. The arrival of the Cultivators displaced the remnant group, resulting in the Blacksmiths becoming segregated from society.
- Marginalisation model (MA)—Under the internal specialisation or Marginalisation model [6, 7, 3], the Ari Blacksmiths and Cultivators share the same ancient history. The adoption of an artisan trade by the Blacksmiths led to their marginalisation within the existing society.

Studying patterns of DNA variation among Ari occupational groups can help shed light on which of these theories is more likely. Under the MA model, which is currently favoured among anthropologists to explain the existence of caste-like occupational groups in southwest Ethiopia [1], observed genetic differences between the two groups should be explained largely by a bottleneck effect in the Blacksmiths consistent with their current isolation, even if the two groups only became isolated from each other very recently. In contrast, under the RN model the two groups descend from two anciently related groups that split perhaps many thousands of years ago, though possibly with subsequent admixture between them. There are alternative theories to the MA and RN hypotheses, including one suggesting the Blacksmiths—along with other artisan groups—migrated to southern Ethiopia after it was occupied by Cultivators, either due to demand for their craft skills or possibly while accompanying invading groups [8, 9]. Here we assume such migrations would result in a genetic relationship between Blacksmiths and Cultivators similar to that expected under the RN model, i.e. such that the two groups split from one another substantially further in the past than under the MA model.

We note that these models are not mutually exclusive [1], as even under a RN model there may have been substantial recent bottleneck effects in the Blacksmiths, as might be expected given their present-day marginalisation. Nonetheless, even after accounting for any bottleneck effects in the Blacksmiths, the RN model implies likely additional genetic differentiation between the two groups due to their ancient relatedness, as we demonstrate using simulations. For example, assuming the remants group consisted of hunter-gatherers [5], the Cultivators might look more genetically similar to other agricultural groups within Ethiopia than the Blacksmiths do.

The most comprehensive genome-wide study of Ethiopians to date [2] analysed 235 individuals from 10 Ethiopian groups, including Ari Blacksmiths and Ari Cultivators. They found that the genetic differentiation between the two Ari occupational groups (*F*_*ST*_ = 0.04) was at a similar level to that observed between multiple ethnic groups sampled across Ethiopia (*F*_*ST*_ range 0.02–0.06). The authors used ADMIXTURE [10] to assign individuals’ genetic variation data into clusters based on shared allele frequency patterns, using an “unsupervised” approach which allows each individual’s genetic data to be assigned to multiple clusters. They noted that the Ari Blacksmiths were assigned almost entirely to a single cluster and that a smaller proportion of this cluster was found at varying levels in all other Ethiopian groups including the Cultivators. The authors suggested the ADMIXTURE results were consistent with the RN model of Ari Blacksmith origins, with subsequent assimilation of their indigenous ancestors into the expanding farming community (including the ancestors of present-day Ari Cultivators) [2]. More recently, other researchers [11] applied the same unsupervised model of ADMIXTURE to these data and additional world-wide samples. They similarly suggested that Cultivators were likely the result of admixture between an ancestral group best represented by the Blacksmiths and another ancestral group that diverged from the Blacksmith-like group > 31kya [11]. Note that the original Remnants model proposed by anthropologists, which indeed is not mentioned in [11], does not on its own imply one-way migration from the ancestors of the Blacksmiths into those of the Cultivators, but we will assume that is the case here to match the observations of these two papers. I.e. our RN model, as simulated below, assumes this asymmetrical migration took place, making the groups more similar genetically than they would otherwise be.

Clustering algorithms such as ADMIXTURE [10] and the closely related approaches STRUCTURE [12, 13] and FRAPPE [14] have been applied in a similar manner in many previous studies to explore the genetic ancestries of world-wide [15] and geographically localized [2] populations. For example, STRUCTURE has been used to suggest the presence of distinct (perhaps anciently-related) ancestral groups that have intermixed to form present-day populations in Africa [16]. However, similar to using principal-components analysis (PCA) [17, 18], it can be difficult to assess whether clustering patterns among groups are due to recent admixture between distinct historical populations or to ancestry shared prior to the populations diverging [15], making interpretation challenging.

**Fig 1:**
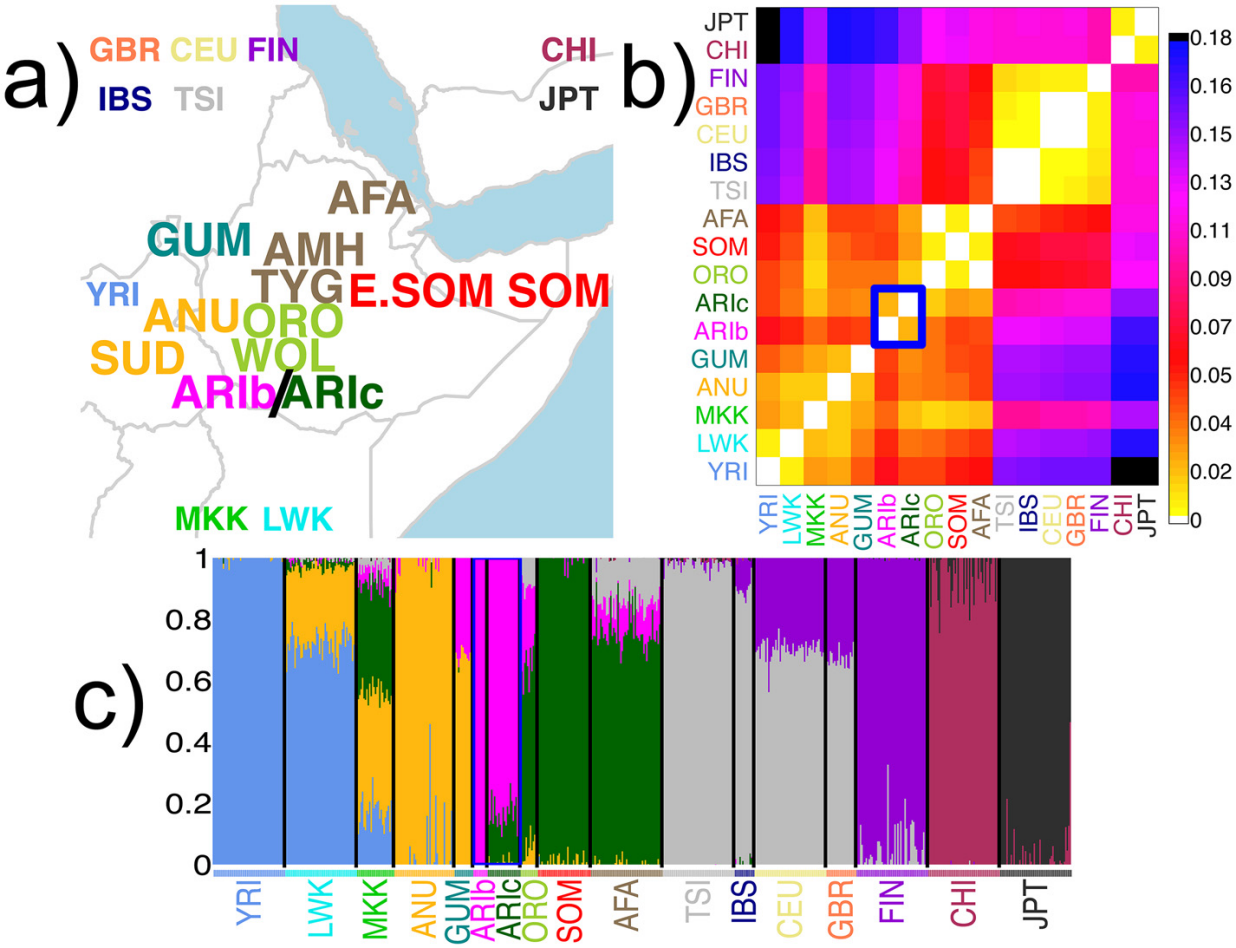
Description of sampled groups, ADMIXTURE clustering and *F*_*ST*_ values. (a) Geographic locations of sampled populations analysed, with the 12 Pagani populations [2] in larger font. The remaining 10 populations include the MKK and those from the 1000 Genomes Project (see www.1000genomes.org for details; CHI = CHB/CHS); locations on map for these 10 populations are indicative. All populations are colored by the group that many of their individuals were assigned to using fineSTRUCTURE; these 17 groups are referred to throughout using the label of one of the majority populations (see S3 Table). (b) Pairwise *F*_*ST*_ comparing all groups (see S3 Table). (c) ADMIXTURE assuming 8 clusters applied to Pagani, 1KGP and MKK individuals, as labeled by the fineSTRUCTURE groups. In (b)-(c), the two Ari groups (ARIb, ARIc) are highlighted with the blue rectangle.

We used an alternative approach to study 237 samples from 10 Ethiopian and 2 neighbouring (Somalia, South Sudan) populations from [2], which we will refer to as the “Pagani” samples. We also incorporated 850 additional samples from 10 other groups from the 1000 Genomes Project (hencefore “1KGP”; http://www.1000genomes.org/) and 28 individuals from one group (MKK) from HapMap Phase3 [19], giving 23 total labeled populations (Fig 1a, S1 Table). We jointly phased all samples with the program SHAPEIT [20] using 659,857 SNPs. We then used CHROMOPAINTER [21] to explore patterns of haplotype sharing among individuals, which has been shown to be both more powerful than techniques that ignore haplotype information [21] and less susceptible to biases arising from SNP ascertainment schemes [22, 23] such as those leading to the chip data used here. Specifically, CHROMOPAINTER uses a Hidden-Markov-Model (HMM) approach [24, 21] to “paint” each haplotype of a sampled “recipient” individual, identifying—at each location of each recipient’s two haploid genomes—the best matching DNA segment from a set of sampled “donor” individuals. I.e. it infers the donor haplotype with which the recipient shares most recent ancestry relative to all other donor haplotypes at the given genomic locus. Using this approach, for each recipient individual we infer their proportion of genome-wide DNA that shares most recent common ancestry with each donor haplotype, identifying the donors (and groups of donors) that appear to be most related genetically to the recipient individual. By comparing results when using different donor sets, we can distinguish whether genetic differences between groups are more likely attributable to ancient or recent isolation, as described below.

We first clustered the 1115 individuals into 17 groups using CHROMOPAINTER and fineSTRUCTURE [21] (Fig 1a, S1–S4 Figs and S1 Table), removing 56 individuals with ancestry signals inconsistent with that of the majority of individuals with the same population label (S1 and S2 Figs) or that failed other quality control metrics, including 7 Ari Blacksmiths and 1 Ari Cultivator (see Methods). In total, our dataset analysed 10 Ari Blacksmiths (ARIb) and 23 Ari Cultivators (ARIc).

To distinguish between the MA and RN hypotheses, we performed three distinct CHROMOPAINTER analyses that differ in which of the 17 groups are used as donors:

- *all-donors*—recipient groups copy from (i.e. are painted using) all other sampled groups (i.e. MKK and all Pagani and 1KGP groups, including their own) as donors
- *non-Ari-donors*—recipient groups copy from all other sampled groups *except* the ARIb and ARIc as donors
- *non-Pagani-donors*—recipient groups copy from 1KGP and MKK groups only as donors

Under each of (A)-(C), we infer a “painting profile” for each individual and world-wide group by measuring the amount of DNA that they copy from each donor group. To compare the ARIb and ARIc under each of (A)-(C), we use a distance-based measure (“total-variation-distance (TVD)”; [25]) that calculates the difference *TVD*_*XY*_ in the average “painting profiles” between any two groups (or two individuals) *X* and *Y* (see Methods). To account for independent drift effects along independent regions of the genome, we also constructed an alternative measure *F*_*XY*_ that scales *TVD*_*XY*_ by differences among chromosomes within each of *X* and *Y* (see Methods).

Like the unsupervised ADMIXTURE analyses of [2] and [11], our analysis (A) allows any sampled individual to copy from any other individual regardless of group label. In contrast, analyses (B) and (C) compose the Blacksmiths (“ARIb”) and Cultivators (“ARIc”) as genetic mixtures of other non-Ari sampled groups only, which is more similar to a “supervised” ADMIXTURE analysis that pre-defines some clusters using surrogate groups. The important distinction is that ARIb and ARIc are allowed to copy from individuals with their own label only under (A). In a scenario where the Blacksmiths and Cultivators shared identical ancestry prior to recent isolation of the Blacksmiths (i.e. MA hypothesis) and have received no DNA from outside groups since, the inferred ancestry patterns of the two groups are expected to be similar under analyses (B) and (C) even if they are very different under analysis (A) [25]. I.e. analyses (B) and (C) would substantially attenuate the signal of genetic differentiation between the two Ari groups under analysis (A) if that signal is attributable solely to strong bottleneck effects in either of the groups after their split. In contrast, under the RN hypothesis the two Ari groups are expected to look genetically different under analyses (B) and (C) in addition to analysis (A), so long as one of the Ari groups is more recently related to at least one other sampled group, regardless of any bottleneck effects in either group since their split. The key difference between analyses (B) and (C) is that the latter allows the comparison of genetic differences between the ARIb and ARIc to those between other geographically near groups in Ethiopia and surrounding areas, under a scenario where each such group uses identical donors and importantly is not allowed to copy from individuals within their own label. Meanwhile, analysis (B) might have more power to distinguish between the two Ari groups, since it uses more geographically near groups as donors compared to analysis (C).

**Fig 2:**
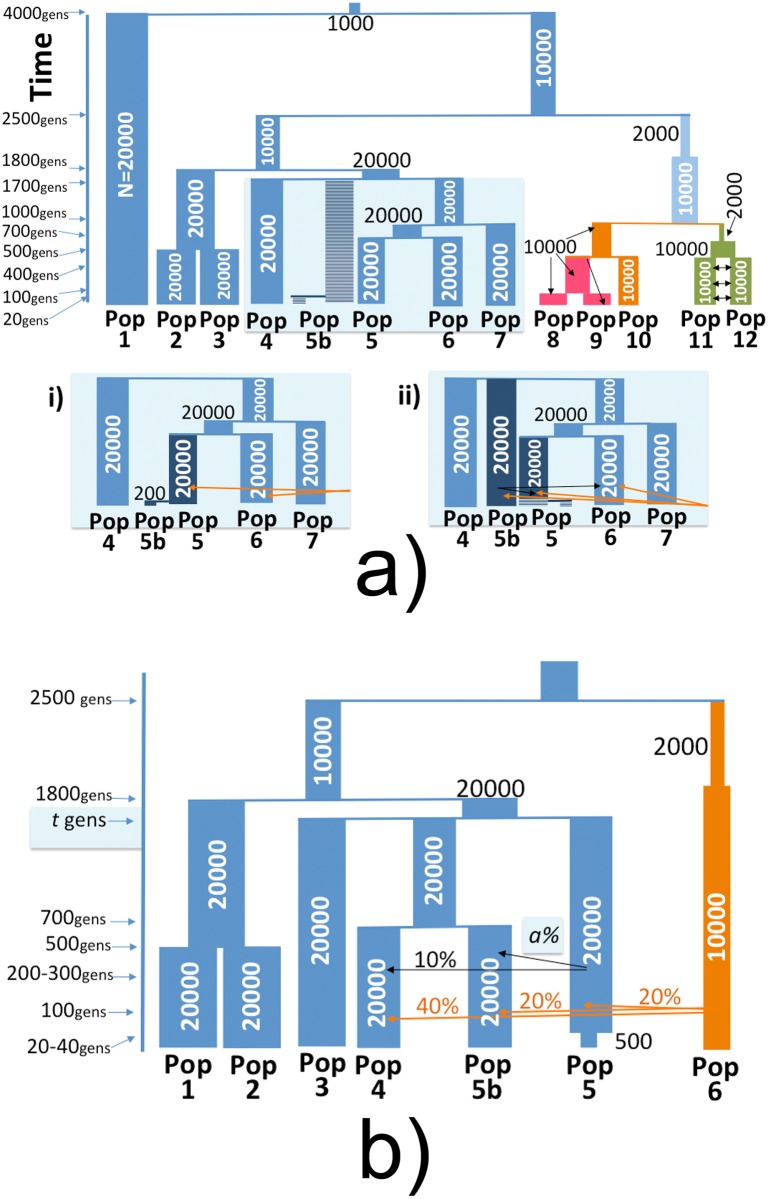
Full and simplified simulated histories under the marginalisation (MA) and remnants (RN) hypotheses. History of populations simulated using MaCS [26]. (a) Thirteen populations simulated under the (i) Marginalisation (MA) model with Pop5 and Pop5b (representing the ARIc and ARIb, respectively) splitting at 20 gens, with a subsequent bottleneck in Pop5b, versus (ii) the Remnants model where Pop5b splits from Pop5/Pop6/Pop7 at 1700 gens and contributes migrants to Pop5/Pop6 between 200 and 300 gen ago. Otherwise all other groups and split times are the same between the two simulation scenarios. Orange arrows indicate migration from Pop10 into Pop5, Pop5b and Pop6. (b) Seven populations simulated under the Remnants model, with black arrows indicating migration from Pop5b into Pop5 and Pop4, and orange arrows indicating migration from Pop6 into Pop5, Pop5b and Pop6 with the given proportions. Pop5 and Pop5b split at varying times *t* ∈ {750, …, 1700}, with a bottleneck in Pop5b occurring 20–40 generations ago and the proportion of Pop5 comprised of Pop5b migrants varying from 50–90%.

We illustrate expected genetic patterns under analyses (A)-(C) by performing several simulations designed to capture key features of the Marginalisation and Remnants hypotheses, incorporating one-way migration in the latter to be consistent with previous interpretations of ADMIXTURE results [2, 11]. These include the following four different “full” simulations that simulate 13 world-wide populations with *F*_*ST*_ values matching that of several of our sampled populations (Fig 2a, S2, S4–S7 Tables):

- “MA”—The simulated “Ari” groups split 20 generations ago, followed immediately by a strong bottleneck in the simulated “ARIb”.
- “RN”—The “Ari” groups split 1700 generations ago, after which migrants from “ARIb” form ≈ 50% of the simulated “ARIc” population over a period 200–300 generations ago.
- “RN+BN”—The “Ari” groups split 1700 generations ago with subseqent migration from “ARIb” into “ARIc” as in “RN”, followed by a strong bottleneck in the “ARIb” starting 20 generations ago.
- “RN+BN+80%”—The “Ari” groups split 1700 generations ago with subseqent migration from “ARIb” into “ARIc” as in “RN” but forming ≈ 80% of the “ARIc” population, followed by a strong bottleneck in the “ARIb” starting 20 generations ago.

While it is difficult to discern appropriate parameters for these simulations given the uncertainty surrounding the history of groups in this region, we followed values proposed by [11] as a guide for our Remnants (“RN”) simulations. In particular the authors suggested that the Cultivators likely resulted from a mixture between a group represented by the Blacksmiths and another group that diverged from the Blacksmiths-like group at least 31kya [11]. For the Marginalisation (“MA”) simulations, the aim was to determine whether a very recent split time between the two groups, which we chose as 20 generations, followed by a strong bottleneck in the simulated Blacksmiths can explain observations similar to those we see in our data. We also performed an additional 24 “simplified” simulations that considered only 7 populations in order to explore how different split times between the “ARIb” and “ARIc”, rates of migration from “ARIb” into “ARIc”, and strength of “ARIb” bottleneck affect our power to distinguish the two groups using our CHROMOPAINTER analyses (Fig 2b, S8 Table) under a hypothetical Remnants setting. Throughout we compare the results from our simulations to those from the real data.

## Results

**Fig 3:**
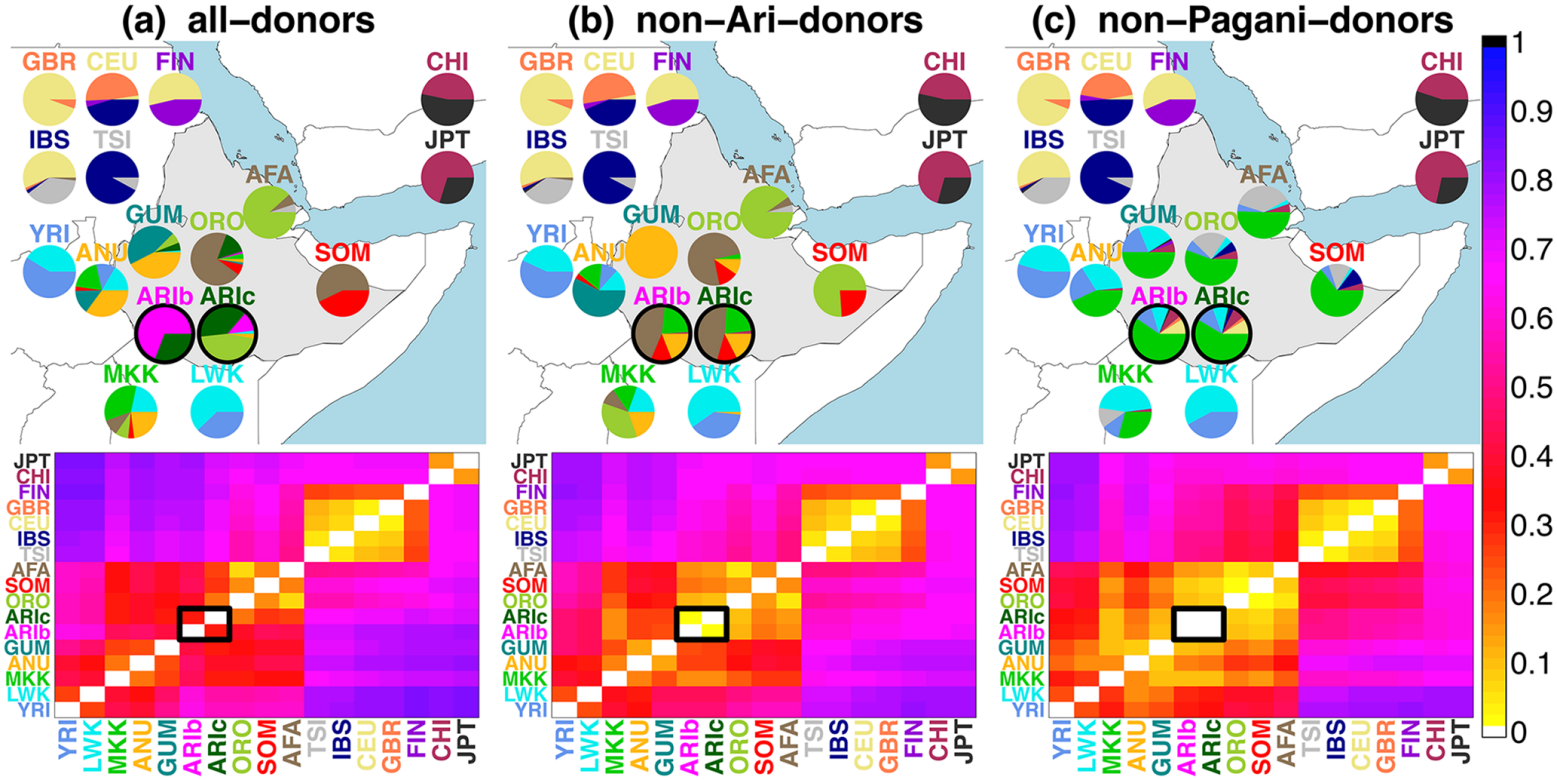
Inferred ancestry composition of groups under each analysis. (top) Inferred ancestry composition of recipient groups when forming each group as mixtures of (a) all sampled groups, (b) all sampled groups except the Ari, and (c) all non-Pagani groups only. The colour of each group’s label provides the key for each pie, with Pagani groups geographically located on the map (roughly) according to the label most represented in the given group. All 1KGP groups and MKK are placed on the map loosely according to their relative geographic positions. (bottom) *TVD*_*XY*_ values comparing the painting profiles for all pairwise comparisons of groups *X*, *Y* under each analysis, with scale at far right. Ari groups (ARIb/ARIc) are highlighted with black outlines in each plot.

Our *F*_*ST*_ (Fig 1b, S3 Table), ADMIXTURE (Fig 1c), fineSTRUCTURE (S1 Fig) and CHROMOPAINTER analysis (A) (Fig 3a, S7a and S11a Figs) results support previous findings [2, 11] that the ARIb appear genetically distinct from the ARIc.

Below we first show how these results and those from [2] and [11] can be consistent with an MA hypothesis, i.e. where the Blacksmiths and Cultivators have a relatively recent split time with the Blacksmiths experiencing a subsequent strong bottleneck. We then outline several lines of evidence using CHROMOPAINTER analyses (A)-(C) that support the MA over the RN hypothesis as the more plausible explanation of observed DNA patterns among the Ari given these sampled data. In particular the MA hypothesis would predict the following genetic patterns, any one of which is not necessarily expected to be true under the RN hypothesis and thus jointly provide substantial support for the alternative MA hypothesis:

1. Any differences in inferred ancestry between the Blacksmiths and Cultivators can be explained by bottleneck effects in the Blacksmiths.
2. The Blacksmiths and Cultivators are similarly related genetically to other groups, both within and outside of Ethiopia.
3. After accounting for drift effects likely attributable to a bottleneck in the Blacksmiths, genetic differences between Blacksmiths and Cultivators are similar to differences among Cultivators.
4. The Blacksmiths and Cultivators have similar signals of recent admixture from other sources, including sources likely from both inside and outside of Ethiopia.
5. DNA segments inherited from distinct admixing sources are genetically similar among Blacksmiths and Cultivators. Furthermore, segments from these differenct sources within Blacksmiths show the same strength of bottleneck effects, consistent with the split between the Blacksmiths and Cultivators occurring more recently than the recent admixture.

Under a hypothetical RN setting, we again note that in order to have any power to distinguish the ARIc and ARIb genetically, our analyses must include at least one sampled group whose ancestors split more recently from those of the ARIc than those of the ARIb and ARIc split from each other. Our dataset contains several Ethiopian groups (Afar, Amhara, Anuak, Tigray, Wolayta) assigned as agriculturalists in [27], whose ancestors plausibly could have split more recently from those of the Cultivators than those of the Blacksmiths and Cultivators split from each other, under a hypothetical RN setting. Also, two groups (ORO,MKK) have *F*_*ST*_ values with ARIc that are lower than those between ARIb and ARIc (Fig 1b, S3 Table), suggesting either or both could represent such a sampled group(s).

We further note that one-way migration from the ancestors of the Blacksmiths into those of the Cultivators, as suggested in [2, 11], will mitigate any genetic differences between the two Ari groups today even if the RN model were true. We assess our power to distinguish the two Ari using simulations under an RN setting, in particular determining the amount of one-way migration that would be necessary to explain observed genetic patterns.

While there are an infinite number of historical scenarios that could be consistent with observed genetic patterns in present-day Blacksmiths and Cultivators, some of which may reflect the RN model, our primary aim is to assess whether the MA hypothesis alone can fit the observations of [2] and [11] as well as the further analyses we perform here. In such a case, we propose the MA model is a more parsimonious explanation given the current marginalised status of Blacksmiths.

### Strong bottleneck effects in the Blacksmiths can entirely explain ADMIXTURE, *F*_*ST*_, and CHROMOPAINTER analysis (A) results

If the Blacksmiths experienced a strong bottleneck relative to the Cultivators, then the genetic diversity among Blacksmiths should be lower than that among Cultivators. Consistent with this, the inferred proportion of Identity-by-descent (IBD) sharing among the ARIc is lower than that among the ARIb (PLINK v1.07 [28] PI_HAT = 0.08 compared to 0.18; S13 Table). Indeed, the inferred proportion of IBD sharing was higher for the ARIb than all other groups in our study, with the next highest the Japanese (JPT; PI_HAT = 0.15; S13 Table).

As separate evidence using a different approach, we painted each ARIb separately with CHROMOPAINTER using only other ARIb as donors, and analogously painted ARIc using only other ARIc as donors, after first matching the two groups for sample size (see Methods). In contrast to the IBD approach implemented in PLINK, our haplotype-based approach should be robust to any potential biases arising from ascertainment of chip data [22]. Supporting this, the inferred average size of shared haplotype segments in the ARIb, which we propose as a measure of relative homogeneity under this approach, is no longer the highest out of all 17 groups and instead is lower than that of GBR and FIN (S14 Table, S14 Fig). This pattern is more consistent with the presumed recent bottlenecks in these latter two populations [25, 29] following the major out-of-Africa bottleneck event [30, 31]. Nevertheless under this second analysis, the median length of matching haplotype segments among ARIb is ≈2 times higher than in ARIc (S14 Table), again consistent with bottleneck effects in the ARIb.

In our “MA” full simulations consistent with the Marginalisation hypothesis, we simulated a split time between the Blacksmiths and Cultivators of only 20 generations ago, and then chose a strength of bottleneck in the simulated Blacksmiths that gave a value of *F*_*ST*_ = 0.025 between the two groups (S4 Table), which is very similar to that of our observed data (*F*_*ST*_ = 0.023; S3 Table). Under this set-up, we note that the patterns seen in ADMIXTURE results (S6a Fig) and CHROMOPAINTER analysis (A) (S12 Fig, top) are very similar to that observed in the real data. This suggests that a bottleneck event in the Blacksmiths, with a very recent split time between the two Ari groups as expected under the MA model, can explain genetic differences observed between them under these approaches.

### After accounting for effects of bottleneck, Blacksmiths and Cultivators are similarly related genetically to other world-wide groups

The differences we observe between ARIb and ARIc under CHROMOPAINTER analysis (A), ADMIXTURE and *F*_*ST*_ are no longer present under CHROMOPAINTER analyses (B) and (C) (Figs 1b–1c and 3, S7 and S11 Figs and S10–S12 Tables). A key difference is that for each Ari group we disallow “self-copying” from individuals with the same label under analyses (B) and (C), which should reduce the magnitude of any differences seen in the other approaches that are attributable to bottleneck effects in the Blacksmiths.

Under our approach, we measured differences in inferred ancestry using TVD (Fig 3, S15 Fig, S15 Table). We also used an alternative score *F*_*XY*_ (S17 Fig, S16 Table) that is proportional to the TVD score between individuals/groups *X* and *Y* but scales this value by ancestry differences across chromosomes within each individual/group to incorporate independent drift effects along the genome (see Methods). Relative to comparisons between other Pagani groups, each of these measures dropped substantially in analyses (B)-(C) compared to analysis (A) when comparing the two Ari groups (S15 and S16 Tables). We also clustered all Ari individuals into two groups based on their inferred ancestry using a novel statistical Markov-Chain-Monte-Carlo (MCMC) algorithm (see Methods). This algorithm correctly classified all Ari individuals by occupational label under analysis (A) but randomly assigned them to the two clusters under analyses (B) and (C) (S19 Table) despite separating other Pagani groups under analyses (A)-(C) (S20–S22 Figs). Furthermore, clustering and *TVD*_*XY*_, *F*_*XY*_ patterns closely follow those when applying the same methods to the simulated “Ari” individuals in the “MA” “full” simulations and noticeably differs from the three “RN” “full” simulation scenarios we considered (S15–S18 Figs and S19 Table).

Informatively, even though all Pagani groups are painted using identical donors under analysis (C), the *TVD*_*XY*_ and *F*_*XY*_ scores between the two Ari groups under analysis (C) are smaller than those between any two other Pagani groups. In particular they are smaller than *TVD*_*XY*_ and *F*_*XY*_ between groups “AFA” and “ORO” (S15 and S16 Tables), who have the smallest *F*_*ST*_ among all pairwise comparisons of Pagani groups (Fig 1b, S3 Table) and show similar patterns in our ADMIXTURE results (Fig 1c, S5 Fig). This suggests the Ari groups are more similar to each other, in terms of how their ancestry relates to the non-Pagani donors, than any other groups sampled within Ethiopia used in this study. Furthermore in analysis (B), in contrast to what you might expect under the RN hypothesis as originally formulated [5], the ARIc are not more closely related to groups currently classified as farmers [27] than the ARIb (Fig 3, S8 Fig, S11, S15 and S16 Tables).

Our 24 “simplified” simulations under the “RN” model (Fig 2b) illustrate scenarios where our CHROMOPAINTER analysis has power to tell apart the two groups under hypothetical Remnants scenarios. As we note below (see GLOBETROTTER results), the two Ari groups have similar sources of recent admixture, likely between a West Eurasian source and an African source as inferred by our analyses and other researchers [32], as well as an additional likely African source inferred by our analyses here. Given these similar recent admixture signals, we likely would only have power to distinguish between the two groups under analyses (B) and (C) if there is at least one sampled group whose ancestors split with one of the two Ari more recently than the ancestors of the two Ari groups split from each other. Such a hypothetical setting, which supports the RN model, seems plausible given *F*_*ST*_(ARIc,ORO) = 0.015 and *F*_*ST*_(ARIc,MKK) = 0.20 are both lower than *F*_*ST*_(ARIc,ARIb) = 0.023 (S3 Table), e.g. the ARIc plausibly may have split more recently from the ORO and/or MKK than from the ARIb. Therefore, while it is impossible to evaluate all historical parameters that may lead to diversity patterns observed today, for these “simplified” simulations we fixed the split time between our simulated “ARIc” (i.e. Pop5 in Fig 2b) and “ORO” (Pop4) groups to 700 generations ago, which gave an *F*_*ST*_ similar to that observed in the real data (*F*_*ST*_(Pop5,Pop4) = 0.011 − 0.014, S8 Table) while accounting for levels of inferred recent West Eurasian admixture in the two groups (see Methods). We then altered the split time between the simulated “ARIc” and “ARIb” (Pop5b) from {750, 800, 900, 1000, 1100, 1200, 1300, 1700} generations ago, choosing a strength of bottleneck in Pop5b for each split time that gave similar *F*_*ST*_ values between the two real Ari groups (*F*_*ST*_(Pop5,Pop5b) = 0.019 − 0.027, S8 Table). We also tried three separate rates of migration from Pop5b into Pop5, the direction of migration suggested by ADMIXTURE results as interpreted in [2] and [11], such that ≈ {50%, 75%, 90%} of Pop5 was comprised of migrants from Pop5b over the period 200 to 300 generations ago.

For these “simplified” simulations, we performed an analysis mimicking CHROMOPAINTER analysis (B) in the real data, though note that we used only five surrogate groups to infer ancestry, which could decrease power. Using techniques described in the next section, in these “simplified” simulations we were able to distinguish Pop5 and Pop5b when the split time was ≥ 1300 generations, even when the proportion of admixture from Pop5b into Pop5 was 90%, and when the split time was ≥ 1100 generations when the admixture proportion was 50–75% (S19 Fig, S18 Table). For split times of 1000 generations or less, i.e. such that the split of Pop5 and Pop5b was at most 300 generations older than the split of Pop5 and Pop4, we could not always distinguish the ancestry of Pop5 and Pop5b under our analysis (B). We note that [11] suggest the split occurred > 31kya (i.e. > 1100 generations ago assuming 28 years per generation), which is older than these split times for which our model has no power. Taking these simulation results at face value, our model’s power to distinguish the two Ari groups requires that the split time between the two Ari groups be ≥ 400 generations older than the split time between the ARIc and another sampled group (e.g. ORO) so long as any one-way admixture from the ancestors of the Blacksmiths to those of the Cultivators was ≤ 75%.

### Genetic differences between Blacksmiths and Cultivators are similar to differences among Cultivators

In addition to having similar genetic profiles under analysis (B)-(C), a recent split between Blacksmiths and Cultivators followed by a bottleneck in the Blacksmiths (i.e. MA hypothesis) predicts that the genetic diversity of the ARIb might fall somewhere along the spectrum of genetic diversity in the ARIc, assuming drift is relatively low in the Cultivators following this split. In particular, after accounting for bottleneck effects in the ARIb, the differences in inferred ancestry between the ARIb and ARIc should not be substantially greater than differences in inferred ancestry among the ARIc. For example, under the MA hypothesis the following should be true for two Ari individuals *X* and *Y*:

1. Due to the bottleneck, on average *F*_*XY*_ should be smaller if *X*, *Y* are both ARIb relative to if *X*, *Y* are both ARIc.
2. In analyses (B) and (C), *F*_*XY*_ where *X* is ARIb and *Y* is ARIc should be similar to *F*_*XY*_ when *X*, *Y* are both ARIc.

**Fig 4:**
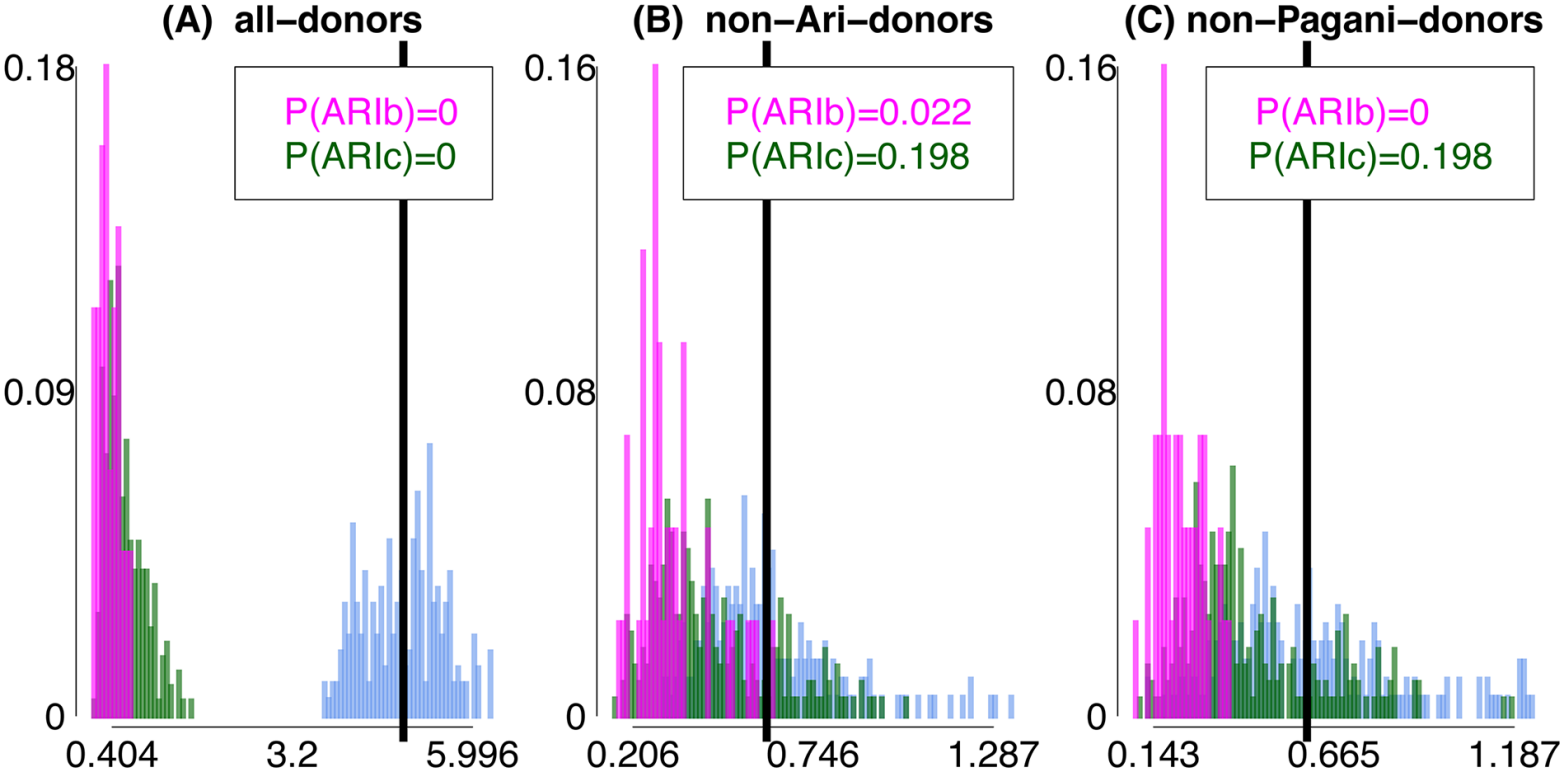
Differences in inferred ancestry under analyses (A)-(C) using *F*_*XY*_. Differences in inferred ancestry under analyses (A)-(C) (using *F*_*XY*_; see Methods) between all pairings of ARIb individuals (pink), all pairings of ARIc individuals (green), and all pairings of one ARIb and one ARIc individual (cyan). In each plot the black vertical line gives the mean difference across the pairings of one ARIb and one ARIc, with *P*(ARIb), *P*(ARIc) giving the proportion of ARIb and ARIc pairings, respectively, with a difference greater than or equal to this mean.

**Fig 5:**
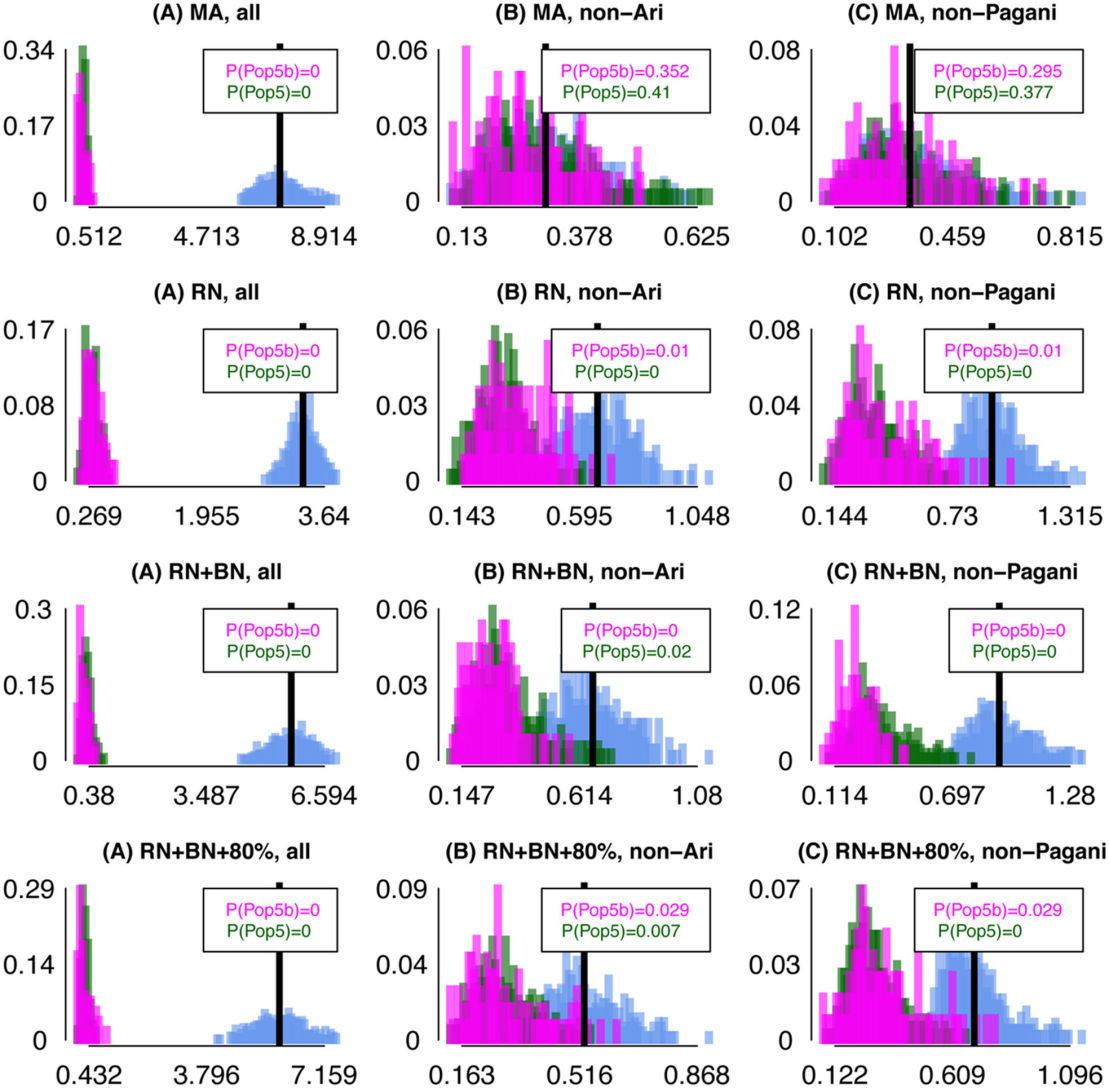
Differences in inferred ancestry under analyses (A)-(C) using *F*_*XY*_ applied to simulated data. Differences in inferred ancestry under analyses (A)-(C) (using *F*_*XY*_; see Methods) between all pairings of simulated “ARIb” individuals (Pop5b, pink), all pairings of simulated “ARIc” individuals (Pop5, green), and all pairings of one “ARIb” and one “ARIc” individual (cyan), for the “MA”, “RN”, “RN+BN” and “RN+BN+80%” “full” simulations. In each plot the black vertical line gives the mean difference across the pairings of one Pop5b and one Pop5, with *P*(Pop5b), *P*(Pop5) giving the proportion of Pop5b and Pop5 pairings, respectively, with a difference greater than or equal to this mean.

Point (1) depends primarily on the magnitude of the bottleneck in ARIb relative to ARIc, while point (2) primarily depends on when the ARIb and ARIc split and any subsequent admixture between them. For each of analyses (A)-(C), in Fig 4 we show the distribution of *F*_*XY*_ for all pairings of individuals *X*, *Y* such that (i) *X*, *Y* are both ARIb, (ii) *X*, *Y* are both ARIc, and (iii) *X* is ARIb and *Y* is ARIc. Our real data results show the trends expected under point (1) for all three analysis, and for point (2) under analyses (B)-(C). To assess how well point (2) fits the observed data, we calculated the proportion *P*(ARIc) of ARIc pairs *X*, *Y* with *F*_*XY*_ greater than the mean *F*_*XY*_ across all pairings where *X* is ARIb and *Y* is ARIc (Fig 4; see Methods). Under analysis (C), *P*(ARIc) ≈0.2 is higher than the maximum analogous proportions comparing any two other Pagani groups (S17 Table). Comparing to the results of our “full” simulations, the observed data proportions are similar to their analogues under the “MA” simulations but consistently larger than the “RN”, “RN+BN” and “RN+BN+80%” simulations (Fig 5).

We can also calculate *P*(ARIb), the proportion of ARIb pairs *X*, *Y* with *F*_*XY*_ greater than the mean *F*_*XY*_ across all pairings where *X* is ARIb and *Y* is ARIc (Fig 4). Note that this is < 0.025 under analyses (B) and (C). In general we expect *P*(ARIb) to be less than *P*(ARIc) due to the bottleneck in the ARIb. However, even in the presence of a bottleneck in our Remnants simulations, *P*(Pop5b) is often greater than *P*(Pop5), in both the “full” (Fig 5) and the “simplified” simulations (S19 Fig, S18 Table), recalling that simulated Pop5b and Pop5 are meant to reflect the ARIb and ARIc, respectively. This suggests that, in contrast to the MA model, under the RN model it is unclear whether the variation among ARIb in inferred genetic relatedness to outside groups should be less than that among ARIc. For example, for some “simplified” simulations where our model has no power to distinguish between the two simulated “Ari” groups, i.e. when migration from Pop5b into Pop5 is ≥ 75% and the split time between Pop5b and Pop5 is ≤ 300 generations older than that between Pop5 and another sampled group (Pop4), nonetheless give *P*(Pop5b) > *P*(Pop5). This in turn suggests that historical parameters behind these simulations are less consistent with the real data observation of *P*(ARIb) < *P*(ARIc).

### The Blacksmiths and Cultivators have similar signals of recent admixture

We also applied GLOBETROTTER [33] separately to each Ari group for analyses (A)-(C), in order to infer recent admixture events in each group. In brief, within each Ari group GLOBETROTTER explores linkage disequilibrium patterns in order to identify and date any putative DNA admixture event(s) from (unknown) ancestral source groups that have occurred in the past ≈4500 years, using other sampled groups as surrogates for the admixing sources (see Methods). Under each of analyses (A)-(C), GLOBETROTTER found significant evidence (*p*-value < 0.01) for at least one admixture event in each of the ARIb and ARIc.

In each of analyses (B) and (C), we infer a simple admixture event at a single time between two sources in both Ari groups, with similar inferred dates, admixture proportions, and sources of ancestry (Table 1 and S20 Table, S23, S25 and S26 Figs) between the two groups. Any small discrepancies in inference between the two Ari groups are likely attributable to differences in sample size, with for example inferred values often as consistent between ARIb and ARIc than between all ARIc and a subset of 10 randomly-chosen ARIc (S21 Table). The inferred admixture event corroborates previous inferences of an admixture event ≈3K years ago involving a West Eurasian source [32, 2, 11] and suggests the same such signals in each Ari group. We refer to this admixing source henceforth as originating from “West Eurasia”, noting that our lack of a comprehensive set of world-wide samples, e.g. with no samples from the Near East, prevents interpretation of the precise source of this admixture. The fact the dates under analysis (B) are significantly more recent than those under analysis (C) likely reflects the different surrogates used and/or different inferred sources and proportions. In particular analysis (C) perhaps picks up signals of the original admixture between “West Eurasia” (from a source best represented by CEU out of our sampled groups) and a more “African”-like source (best represented by MKK), which matches results from previous analyses using similar surrogates [2, 11]. In contrast, the date in analysis (B) could reflect admixture between more geographically local groups at a more recent date, i.e. between an already admixed group (best represented by AFA in each Ari group) and another likely African group (best represented by ANU in each Ari group). As the inferred dates in (B) are relatively old and separated by only ≈30–40 generations from the analysis (C) results (Table 1), and/or as there may have been continuous admixture over this timeframe, GLOBETROTTER may not have the power to separate these events/dates reliably with these sample sizes. Indeed there is suggestive evidence of two or more distinct dates of admixture in both Ari groups under analysis (B) (S25 Fig, S22 Table), though the wide confidence intervals in our date estimates when assuming two dates reflects the difficulty in reliably characterizing this signal. If multiple dates or continuous admixture is indeed the case, our inferred dates under analysis (B) might be biased towards more recent intermixing.

**Table 1:** GLOBETROTTER’s inference under analysis (A), (B) and (C).

Analysis	Group	**First Event**							
		Date (gen)	Date (years)	%	Source 1		%	Source 2	
A	ARIb	52 (27–74)	466CE (150BCE-1166CE)	32	SOM	GBR(16%),ANU(28%),SOM(54%)	68	ARIc	ARIc(100%)
	ARIc	71 (57–83)	66BCE (402BCE-326CE)	24	ARIb	ANU(15%),GUM(19%),ARIb(53%)	76	ORO	ORO(80%)
		**Second Event**							
		Date (gen)	Date (years)	%	Source 1		%	Source 2	
	ARIc	Same	Same	19	ANU	MKK(11%),GUM(12%),ANU(62%)	81	ORO	ARIb(18%),ORO(72%)
B	Group	**First Event**							
		Date (gen)	Date (years)	%	Source 1		%	Source 2	
	ARIb	72 (53–85)	94BCE (458BCE-438CE)	41	ANU	SOM(16%),ANU(84%)	59	AFA	AFA(90%)
	ARIc	73 (54–82)	122BCE (374BCE-410CE)	41	ANU	SOM(16%),ANU(84%)	59	AFA	AFA(100%
C	Group	**First Event**							
		Date (gen)	Date (years)	%	Source 1		%	Source 2	
	ARIb	121 (91–149)	1466BCE (2250BCE-626BCE)	30	CEU	CEU(32%),MKK(46%)	70	MKK	YRI(17%),LWK(20%),MKK(55%)
	ARIc	100 (85–115)	878BCE (1298BCE-458BCE)	39	CEU	IBS(14%),CEU(16%),MKK(52%)	61	MKK	YRI(19%),LWK(20%),MKK(52%)

GLOBETROTTER’s inferred dates (in both generations from present and years, bootstrap 95% CIs given in parenthesis; CE = common era; BCE = before common era), admixing sources (single best matching sampled surrogate is given first, followed by mixing proportions > 10% giving more precise inference on the haplotype make-up of the source; see Methods), and proportion (%) of admixture contributed from each source for inferred admixture events in the ARIb and ARIc under analyses (A), (B) and (C). Assuming a generation time of 28 years, generations *g* were converted to years *y* using the formula: *y* = 1950 − (*g* + 1) × 28.

GLOBETROTTER results under analysis (A) are more difficult to compare between the two Ari groups, as they do not use the same set of surrogates here as is the case in analyses (B) and (C). Nonetheless, signals in each group are similar and suggest a complex signal where both Ari groups are admixed with a third group, with this admixture dated to a similar time period as that inferred under analysis (B). For example, the ARIc show mixing around 400BCE-330CE between three distinct sources most similar to the ARIb, ORO and ANU, respectively (S20 Table). For the ARIb, GLOBETROTTER under analysis (A) infers mixing between three groups in some analysis (S21 Table) but only two groups in others (S20 Table). This is likely attributable to decreased power in the ARIb due to their smaller sample size relative to the ARIc, as well as the strong bottleneck in the ARIb, which can be thought of as further reducing the effective number of individuals relative to ARIc. To simplify our analysis (A) results, we also applied GLOBETROTTER to each Ari group using only four surrogate groups: ANU, ORO, TSI and the other Ari group (“A-sim” results in S20 Table). This analysis concluded three-way intermixing in both groups, with confidence intervals of inferred dates overlapping (ARIb: 402BCE-690CE; ARIc: 542BCE-270CE) and with at least one inferred source in each group best represented by the other Ari surrogate.

The complexity of the inferred admixture under GLOBETROTTER analysis (A) makes it difficult to interpret reliably [33]. For example, interpreting the three inferred source groups is challenging, as both Ari groups and many other Ethiopia groups (such as ORO) are thought to have substantial admixture from a West Eurasian source (S10 Table, [32]) and hence are subject to the same interpretation difficulties discussed above for analysis (B). Furthermore, as in analysis (B) there is suggestive evidence of multiple dates of admixture in each Ari group (S23 and S24 Figs, S22 Table), though again GLOBETROTTER does not conclude multiple dates of intermixing, perhaps due to the relatively small number of samples in this analysis.

Nonetheless, the GLOBETROTTER results under analysis (A) support three distinct sources intermixing (e.g. see S23 Fig), either at roughly the same time in the past or perhaps with some of the sources intermixing more recently than others. As there was no clear evidence of three separate groups intermixing under analysis (B) in either Ari group, the additional third source captured in analysis (A) is likely more related to the two Ari than any other sampled group. Determining the contribution from this group is difficult. For example, for the strongest inferred events (i.e. “First Event” in S20 Table), the total inferred contribution from the ARIb into the ARIc is ≈12–13% across analyses, while the total inferred contribution from the ARIc into the ARIb is much larger at ≈68–72%. However, these very different proportions are still consistent with the same group contributing DNA to each. In particular, GLOBETROTTER and our related linear modeling methods (see Methods; [25]) tend to down-weight heavily bottlenecked groups (like the ARIb) as surrogates for any putative admixture events that occurred further in the past than the bottleneck (e.g. see simulation results in S12 and S13 Figs). This is not unexpected or necessarily undesirable, as present-day descendants that are heavily bottlenecked from the original admixing source will look less genetically similar to that source. However, as a consequence, if a group equally related to each Ari group contributed DNA to each at the same proportion prior to a bottleneck in the Blacksmiths, GLOBETROTTER’s inferred ARIb contribution to the ARIc would likely be down-weighted relative to the inferred ARIc contribution to the ARIb. We demonstrate this phenomenon using simulations under a MA hypothesis (S27–S30 Figs and S23 and S24 Tables; see Methods).

Therefore we cannot determine whether this additional admixing source inferred under analysis (A) supports an MA model suggesting the same source contributed DNA to the recent shared common ancestor of the two Ari groups, or whether it supports an RN model where the ARIb and ARIc are anciently related and have each intermixed with one another since their initial split. Nonetheless, the inferred date of intermixing is recent (< 3kya) and thus consistent with the Blacksmiths and Cultivators being anciently or relatively recently related. Furthermore, we again note that whether assuming one or two distinct dates of admixture, the inference under each of analyses (A)-(C) is similar between the two Ari groups (Table 1, S20–S22 Tables) and thus consistent with them having recent common shared ancestry.

### Introgressed and non-introgressed segments are similar in both Ari groups

To further assess whether the Ari share similar genetic origins, we performed an analysis independent of CHROMOPAINTER analyses (A)-(C), based on separating segments inherited from African and “West Eurasian” ancestral source groups. To identify segments from different sources, within each haploid genome of each Ari individual, we fixed the YRI and CEU as surrogates for the two admixing source groups. We then used CHROMOPAINTER to identify all segments containing ≥ 100 contiguous SNPs that we could confidently assign to one of the two surrogates based on new simulations mimicking the presumed recent admixture history of these groups (S31 and S32 Figs; see Methods). Due to a lack of proper surrogate for an ancestral “Ari”-like source outside of the two sampled Ari groups, we did not attempt to characterize all three source groups identified in GLOBETROTTER analysis (A), but instead focused on segments of likely non-African versus African origin. We took each pair of Ari individuals and first extracted all segments within the haploids of each individual that were assigned to one of the two surrogates (i.e. YRI or CEU). Then, separately for each surrogate, we found the proportion of allele matches between haploids from different individuals at all SNPs that overlapped within segments assigned to that surrogate. In this manner, we inferred the genetic similarity between each pair of Ari individuals separately for segments inherited from each source. We used two different methods, called “E-M” and “NNLS” (see Methods), for matching segments to YRI and CEU; each method gave similar results.

Analogous to our comparison of inferred ancestry results for CHROMOPAINTER analyses (B) and (C), for both CEU and YRI segments, the distribution of similarity scores between ARIb and ARIc individuals falls on the distribution of similarity scores among ARIc individuals (Table 2, S33 Fig). Overall patterns match those expected if the Blacksmiths’ ancestors experienced a strong bottleneck effect after splitting from those of the Cultivators under the MA model, as explained above. In contrast, if the RN model were true, genetic differences between the ARIb and ARIc in at least the non-“West Eurasian” segments (i.e. for which YRI acts as a surrogate) are expected to be larger than differences among the ARIc.

**Table 2:** Proportion of matching alleles within segments inferred as CEU and YRI.

	Method	Within ARIb		Within ARIc		Between ARIb and ARIc	
		n_SNPs	%	n_SNPs	%	n_SNPs	%
CEU	E-M	24.8 (22.6–27.0)	0.697 (0.684–0.708)	25.3 (23.2–28.7)	0.681 (0.672–0.69)	23.4 (21.4–25.6)	0.68 (0.671–0.689)
CEU	NNLS	64.6 (61.7–69.9)	0.699 (0.69–0.707)	69.4 (65.3–76.7)	0.683 (0.678–0.689)	64.3 (60.8–69.4)	0.682 (0.678–0.688)
YRI	EM	110.3 (106.8–114.5)	0.702 (0.695–0.71)	98.6 (91.8–103.1)	0.685 (0.681–0.69)	101.2 (95.4–105.1)	0.685 (0.681–0.69)
YRI	NNLS	277.0 (270.1–282.2)	0.705 (0.7–0.718)	251.8 (236.8–260.9)	0.692 (0.689–0.697)	261.0 (248.3–268.2)	0.692 (0.689–0.696)

The proportion of SNPs (%) whose allele types matched out of n_SNPs(× 1000) total comparisons, when comparing all $\left(\begin{array}{c} 2\\ 1 \end{array}\right)\times \left(\begin{array}{c} 2\\ 1 \end{array}\right)=4$ pairwise combinations of the haploid genomes from a pair of individuals. Only SNPs within segments with ≥ 100 contiguous SNPs inferred as CEU or YRI with probability > 0.94 for E-M and > 0.66 for NNLS are considered. The median values across all pairwise comparisons of ARIb individuals, ARIc individuals, and ARIb-ARIc individuals are provided in the columns, with the inner 95% empirical quantiles given in brackets.

### Placing an upper bound on the start of genetic isolation between the Blacksmiths and Cultivators

**Fig 6:**
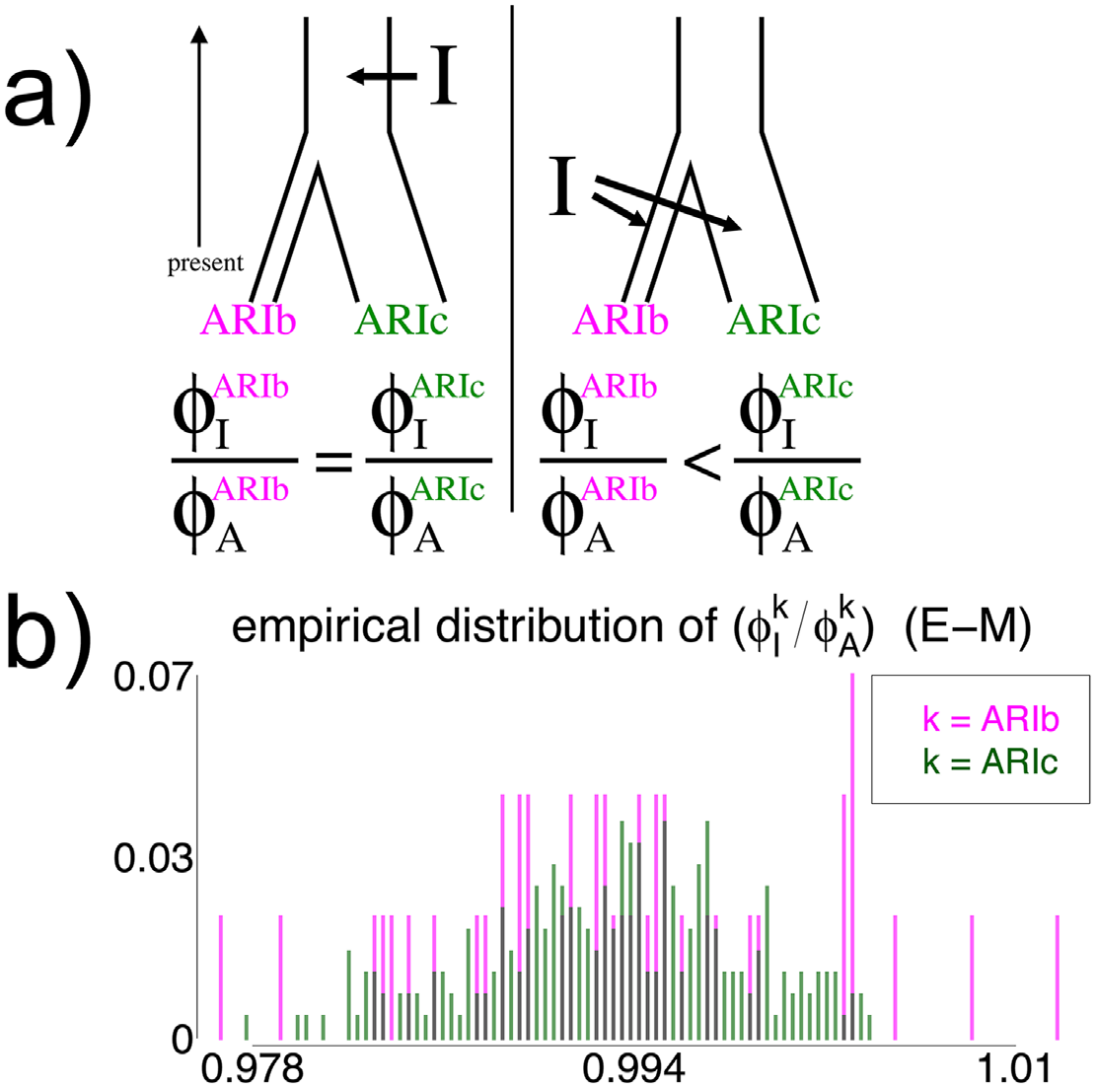
Effect on genetic similarity of two possible timings of DNA introgression. (a) If the DNA introgression from source *I* occurred before the split of the Blacksmiths (ARIb) and Cultivators (ARIc) and subsequent genetic isolation of the Blacksmiths, as in the left depiction, the genetic similarity among ARIb for segments inherited from *I* (i.e. $\phi_{I}^{\text{ARIb}}$) relative to the pre-introgression segments from the ancestral population *A* ($\phi_{A}^{\text{ARIb}}$) should be the same as the analogous ratio of genetic similarity among the ARIc (i.e. $\phi_{I}^{\text{ARIc}}/\phi_{A}^{\text{ARIc}}$). In contrast, if the introgression from *I* occurred more recently than the split and isolation, as in the right depiction, the ratio $\left(\phi_{I}^{\text{ARIb}}/\phi_{A}^{\text{ARIb}}\right)$ should be less than the ratio $\left(\phi_{I}^{\text{ARIc}}/\phi_{A}^{\text{ARIc}}\right)$, since the component from *I* has experienced less drift effects than the component from *A* in the ARIb. (b) The distributions of $\left(\phi_{I}^{\text{ARIb}}/\phi_{A}^{\text{ARIb}}\right)$ and $\left(\phi_{I}^{\text{ARIc}}/\phi_{A}^{\text{ARIc}}\right)$ across all pairwise comparisons of individuals within each Ari group, when segments’ sources were inferred using the E-M model with a threshold of 0.94 when assuming *I* is the “West Eurasian” source (see Methods). The strong similarity in distributions is consistent with the introgression *I* occurring less recently than the split.

While it is difficult with these data to ascertain precisely when the Blacksmiths and Cultivators split (which might be possible with sequencing data in these groups; see Discussion), we can infer whether the split occurred before or after the admixture involving “West Eurasia” if we assume (i) the bottleneck in the Blacksmiths occurred immediately after the two groups split and (ii) the “West Eurasian” and non-“West Eurasian” intermixing ancestral source groups are the same between the two Ari (consistent with results here; Table 2, S33 Fig). We illustrate this in Fig 6a. If the admixture is older than the isolation, then both the introgressed and non-introgressed segments in the Blacksmiths have been subjected to the same amount of drift effects from the bottleneck. In contrast, if the admixture event occurred more recently than the isolation and subsequent bottleneck in the Blacksmiths, then under assumption (i) the introgressed segments have been affected less by drift than the non-introgressed segments. Therefore we can compare the levels of genetic similarity among Blacksmiths within introgressed and non-introgressed segments to infer whether the split occurred before the admixture or vice versa. We assume here the introgressing source is the “West Eurasia” source, though a similar argument follows if the introgressing DNA comes from the non-“West Eurasian” source.

However, in addition to separate drift effects, the levels of genetic diversity within introgressed versus non-introgressed segments can also differ due to varying amounts of diversity in the two source groups at the time of admixture. Therefore, to calibrate differences in the relative amount of genetic homogeneity between the two sources, we infer the relative levels of similarity among ARIc within introgressed and non-introgressed segments. Incorporating assumption (ii), if the introgression is older than the split, then the ratio of genetic similarity in the ARIb among introgressed versus non-introgressed segments should be the same as the analogous ratio in the ARIc. In contrast, if the introgression occurred more recently than the split and bottleneck in the Blacksmiths, the ratio of genetic similarity among ARIb in introgressed versus non-introgressed segments should be less than the analogous ratio in the ARIc (see Fig 6a).

Using the same segments inferred using CEU and YRI as surrogates for each of the two admixing sources, Fig 6b and S34 Fig give the ratios of inferred similarity in introgressed segments versus non-introgressed segments for every pairing of Ari individuals within each of the ARIb and ARIc. Under each of the “E-M” and “NNLS” methods, there is no noticeable difference between the ratios of the two groups. These results are consistent with the split and subsequent bottleneck in the Blacksmiths occurring more recently than the “West Eurasia” introgression event, or at least not substantially before the introgression, and therefore sometime within the last 2.5–4.5K years or so. We note that this observation assumes we have enough power to detect different strengths of bottleneck effects if the split were substantially older than the introgression. Encouragingly, empirical quantiles for similarity scores do not overlap between the ARIb and ARIc under each method for YRI-matched segments and sometimes for CEU-matched segments (Table 2). Assuming the MA hypothesis is true, this demonstrates the approach has some power to separate DNA segments subjected to different strengths of bottleneck effect, plausibly arguing against the split at least being substantially older than the introgression event, even if we cannot be more precise using this technique.

## Discussion

Overall our analyses here suggest evidence for strong bottleneck effects in the Blacksmiths (S13 and S14 Tables), and that these effects appear to be driving differences between the two Ari groups observed today using *F*_*ST*_, unsupervised ADMIXTURE, and our CHROMOPAINTER analysis (A). For example, *F*_*ST*_(ARIc,ORO) is lower than *F*_*ST*_(ARIc,ARIb) (Fig 1b, S3 Table), and *TVD*_*XY*_ and *F*_*XY*_ under analysis (A) suggest smaller genetic differences between the ARIc and other sampled groups including the ORO than that between ARIc and ARIb (Fig 3, S15 and S16 Tables). Nonetheless our analyses (B) and (C), designed to attenuate bottleneck effects in the Blacksmiths, show discernible differences between the inferred ancestry of ARIc and all other groups, including ORO, but no clear difference between the inferred ancestries of ARIc and ARIb (e.g. Fig 3, S15, S16 and S17 Tables). However, as we demonstrate via simulations, distinguishing between the MA and RN models is challenging if one assumes there was substantial one-way migration from the ancestors of Blacksmiths to those of the Cultivators under an RN model, as suggested from previous interpretations of unsupervised clustering algorithms [2, 11].

These difficulties notwithstanding, we believe the MA hypothesis is a more parsimonious explanation given the Blacksmiths’ currently marginalised status. I.e. such marginalisation can plausibly lead to a substantial bottleneck effect in the Blacksmiths, which in turn is consistent with all of our results. In contrast, harmonizing the RN model with the data analysed here requires an additional assumption beyond this bottleneck effect, namely (1) that we have not sampled a group whose ancestors split more recently from either Ari group than the Ari groups’ ancestors split from each other, or (2) that there were substantial levels of intermixing between the ancestors of ARIb and ARIc since the two groups initially were isolated from one another. Assumption (1) is perhaps less likely given our analyses included other Ethiopian groups described as agriculturalists [27] and groups that are more genetically similar to the ARIc than the ARIc are to the ARIb using the measures noted above. Assumption (2) is plausible under a RN model, given the two groups currently reside together. Indeed we detected likely very recent intermixing (perhaps occuring only a generation ago) between the two Ari groups in a “Blacksmiths” individual that we excluded from our analyses (S3 Fig), though this was the only case of such very recent intermixing observed in these data. Presuming assumption (1) is false, any older intermixing between the two Ari groups’ ancestors would have to be substantial enough to decrease our power to tell the two groups apart today. For example, our analysis (C) results suggest that the two Ari groups are more similar to one another when compared to outside groups than any other pairwise combination of Pagani groups (Fig 3, S11 Fig, S12, S15, S16 and S17 Tables), which is difficult to reconcile with the two Ari groups being anciently related without large amounts of subsequent intermixing.

If the RN model were true, simulations that replicate patterns in our observed data (Fig 5, S19 Fig, S18 Table) suggest our model should have power to distinguish the ancestries of the two Ari groups so long as one other sampled group, which we argue could be the ORO or MKK, split ≥ 400 generations more recently from the Cultivators than the two Ari groups split from each other, even if the Cultivators were comprised of 75% migrants from the Blacksmiths over the period 200 to 300 generations ago. We note again that one-way intermixing from Blacksmiths to Cultivators was proposed based on genetic evidence [2, 11] rather than anthropological findings, and that the overall inferred contribution of the ARIb to the ARIc’s ancestry profile is < 20% in all of our analysis (A) results. In contrast, our analysis (A) GLOBETROTTER results infer the ARIc contribution to the ARIb’s ancestry profile to be > 65%, which might argue for substantial asymmetric migration from the ancestors of the Cultivators to that of the Blacksmiths. However, we note that this need not be the case. In particular the ARIb has its lowest *F*_*ST*_ with the ARIc out of all other sampled groups (S3 Table), so it is not surprising that GLOBETROTTER infers the ARIb to share the majority of its ancestry with the ARIc relative to the other groups. Overall we argue that there is no evidence in these data that clearly support the RN hypothesis over the MA, with or without moderate levels of intermixing between the two groups, including the difficult-to-interpret GLOBETROTTER analysis (A) results. We note that currently the MA hypothesis is favored among anthropologists for explaining the existence of caste-like occupational groupings in southwest Ethiopia [1], and we show here that this hypothesis is consistent with available genetic evidence.

As further confirmation of the common recent genetic origins of the Ari, we also used the alternative approach of *D*-statistics (see [34]) to discern whether the ARIc and ARIb form a clade relative to a clade containing any pairing of sampled African groups with little to no inferred recent West Eurasian admixture (see S25 Table). Among six such pairings, we found no *D*-statistics with a corresponding ∣*Z*∣-statistic greater than 3, suggesting we could not reject an Ari clade and confirming the Ari groups appear more genetically related to one another than to these other African groups (S25 Table).

An artefact leading to our observations of substantial bottleneck effects in the ARIb could arise if at least some of the sampled ARIb individuals were more closely related (i.e. at a family level) to one another relative to the ARIc, perhaps due to sampling artefacts. However, the ARIb and ARIc from [2] each contained individuals with reported birthplaces spanning a similar number of different locations within the region, suggesting that it is unlikely that any such sampling artefacts are playing a major role. A similar artefact might occur if phase information was captured more accurately for the ARIb than the ARIc via the phasing program SHAPEIT [20]. I.e. the ARIbs’ inferred haplotypes may have fewer “switch errors”, which in turn could lead to them appearing relatively more genetically homogeneous. In fact, better phasing for the ARIb might be expected if they are less genetically diverse than the ARIc, consistent with a bottleneck in the Blacksmiths and the MA hypothesis. However, we note that the average sizes of contiguous DNA segments painted by a single donor haplotype as inferred by CHROMOPAINTER were very similar when forming the ARIc or the ARIb using the non-Ari groups as donors (S9 Table), suggesting higher levels of phasing errors or other genotyping inconsistencies in the ARIc relative to the ARIb are not playing a major role. Furthermore our IBD sharing analysis ignoring phase information gave a similar conclusion of greater homogeneity among ARIb relative to ARIc (S13 Table).

CHROMPAINTER analyses (B) and (C) suggest that the ARIb and ARIc are roughly equally related to all other sampled non-Ari groups. There is some ability to tell the two groups’ inferred ancestries apart under these analyses (e.g. S8 Fig), though we note that these differences are small relative to those between all other sampled groups (Fig 3, S15 and S16 Tables). Strong bottleneck effects in the Blacksmiths can result in their appearing genetically distinct from the Cultivators even under analyses (B)-(C), plausibly over a short time period depending on the strength of the bottleneck, which we try to account for by considering variation in inferred ancestry patterns among individuals’ chromosomes within each Ari group. Increasing the number of sampled individuals from each group (Ari or otherwise) could further increase the power to distinguish Ari groups under these approaches to shed further light on the MA versus RN hypotheses. Increasing the number of outside groups used to describe the Ari ancestry might increase power as well, though likely only if incorporating additional geographically near groups, given that other world-wide groups are not featured prominently in analysis (B). In particular our GLOBETROTTER results under analysis (A) suggest that in addition to admixture from “West Eurasia”, there is admixture in both Ari groups from a source best represented by the Ari out of all of our sampled groups. Further dense sampling of Ethiopia might enable a better genetic description of this group, helping to confirm whether it is the same admixing source for the ARIb and ARIc and whether there were multiple episodes of admixture from varying sources over different time periods. In addition, as GLOBETROTTER is more likely to pick up recent signals over older ones, increased sample sizes might enable detection of any potential older intermixing between the ARIb and ARIc under a hypothetical RN setting.

Using more dense genetic data, e.g. from sequencing, might also increase power in a similar manner. Acquiring sequenced individuals from each Ari group would have the additional benefit of allowing inference of the split time between the two groups using pairwise sequentially Markovian coalescent (i.e. PSMC and MSMC) techniques [35, 36, 37]. For example, a recent study applying these approaches to individuals sampled from Ethiopian groups included in this paper suggested one such group, the Gumuz (GUM in our study), split from each of four other Ethiopian groups (Amhara, Ethiopian Somali, Oromo, Wolayta) ≈20–40K years ago [38]. While that study did not include data from Blacksmiths or Cultivators, given that genetic differences are substantially larger between GUM and each of {AFA,ORO,SOM} relative to differences between ARIb and ARIc in our analysis (C) (S15 and S16 Tables), it is plausible that 40kya provides a very conservative upper bound for the split time of Blacksmiths and Cultivators. Our attempts to refine this upper bound do not use the rich information from sequencing but are consistent with the bottleneck in the Blacksmiths occurring more recently than the “West Eurasia” admixture event, i.e. within the last ≈4,500 years, although this analysis may be influenced somewhat by a lack of power as discussed above. Evidence for the origins of blacksmithing in Ethiopia remain incomplete, but iron and bronze objects were first discovered on sites from the pre-Aksumite period, suggesting the existence of such practices in the mid to first Millennium BC [39, 40]. Therefore our results are consistent with the start of genetic isolation between Blacksmiths and Cultivators corresponding roughly to a time period near the introduction of blacksmithing in the region.

Our findings serve as a cautionary tale for over-interpreting clustering, e.g. ADMIXTURE plots or results from other unsupervised learning techniques applied to genetic data. In particular the ADMIXTURE plots appear similar in each of the “MA” and “RN” simulation scenarios in this case (S6 Fig), though the two hypotheses reflect very different ancestral histories. Previous studies have shown that individuals from a single genetically isolated group can be grouped into a distinct homogeneous cluster by these algorithms, for example the Kalash in an application of STRUCTURE to world-wide populations [41]. We believe a similar effect is causing the Blacksmiths to all be assigned to a single cluster here, although in this case one that is shared by nearby populations. In general this suggests that if such a homogeneous cluster is observed, one should check whether the individuals in the cluster appear to be more genetically homogeneous than the other sampled individuals, particularly when clustering individuals from isolated or geographically localised groups. If so, further investigations such as those performed here are warranted.

Importantly, a comparison of approaches here (analogous to supervised ADMIXTURE; [42]) allows us to distinguish genetic structure attributable to bottleneck effects within a population from that attributable to shared ancestry with outside groups. In particular, after accounting for “self-copying” or high levels of genetic similarity within the ARIb (analysis (A)), we demonstrate that the ARIb and ARIc look genetically similar in terms of shared ancestry with other sampled groups (analyses (B)-(C)). A more parsimonious explanation for this observation favours the Marginalisation model over the Remnants hypothesis, and helps towards resolving a long-standing controversy on the origins of different Ari caste-like occupational groups [1]. Furthermore, this provides evidence that a societal practice, namely the marginalisation of artisan communities, can drive strong genetic differences (*F*_*ST*_ = 0.02 − 0.04) between groups without involving any outside introgression and possibly occurring within the last 4,500 years.

It is straight-forward to apply these models to samples from other geographic regions, and may be particularly helpful in similar cases where different groups might be subjected to strong isolation effects driving genetic differences due to societal divisions, such as in India [43]. Such careful analyses can help to resolve major questions about whether genetic diversity is primarily driven by ancient demography or by more recent factors such as admixture, social exclusion and drift.

## Materials and Methods

### Data

Our dataset consisted of 237 individuals from 12 different populations from Ethiopia, Somalia and South Sudan (“Pagani”, [2]), provided by the authors, 850 individuals from 10 populations from the 1000 Genomes Project [44] (“1KGP”; www.1000genomes.org), taken from the file “ALL_1000G_phase1integrated_v3_impute_macGT1.tgz” from https://mathgen.stats.ox.ac.uk/impute/data_download_1000G_phase1_integrated.html, and 28 individuals from 1 population (MKK) from HapMap Phase3 [19]. These datasets had 659,857 SNPs in common. Our aim was to incorporate data from several world-wide groups in our analyses of the Pagani resource, while still maintaining a large number of densely genotyped SNPs to ensure increased power using our haplotype-based approach. As noted in the Discussion, we do not expect that including individuals from populations not closely related to Ethiopian groups would alter power to test our hypothesis (e.g. given the results of analysis (B)). As noted in [2], all Pagani samples were ascertained such that their self-reported ethnicity matched that reported for the donor’s parents, paternal grandfather and maternal grandmother.

We removed 33 individuals who had an identity-by-descent (IBD) score as inferred by PLINK v1.07 [28] (PI_HAT) ≥ 0.2 with any other individual. Based on this IBD analysis we removed 6 Ari Blacksmiths (the same ones removed in [2] for the same reason), 1 Ari Cultivator (including one of the two removed in [2]), 1 Sudanese (including one of the three removed in [2]), 4 British individuals (GBR), 9 Chinese individuals (CHS), 2 Masaii individuals (MKK) and 10 Luhya (LWK) individuals.

Clustering analysis using fineSTRUCTURE [21] (details below) removed a further 23 individuals whose inferred ancestry looked different from other members assigned to their cluster group (S1 and S2 Figs). In particular, in addition to one Blacksmith with clear Cultivator ancestry (S3 Fig), we removed 2 Ethiopian Somalis, 2 Amhara (including the single Amhara individual removed in [2]), 6 Wolayta, 1 Oromo, 1 Somali, 6 Gumuz (including the single Gumuz individual removed in [2]) and 4 Sudanese individuals (including those removed in [2]). Therefore along with the IBD analysis, 56 individuals were removed in total. As an example of our removal procedure based on this visual inspection, the 6 Wolayta and 6 Gumuz individuals we removed are highlighted with green rectangles in S1 Fig. Note that these 6 Wolayta individuals were clustered together using fineSTRUCTURE, and split quite early (i.e. near the top) of the inferred fineSTRUCTURE tree, suggesting they are not very closely related to the other Oromo and Wolayta individuals (i.e. the ones assigned to the final “ORO” group and hence labeled as “ORO” in S1 Fig). Visual inspection of the heatmap (S1 Fig) showed that these 6 Wolayta individuals were inferred to share a relatively large proportion of ancestry to a set of 6 individuals labeled as Gumuz, perhaps indicating recent admixture between Wolayta and Gumuz individuals. Thus any inferred shared ancestry with these 12 Wolayta/Gumuz individuals could reflect sharing with either the ancestors of the Gumuz and/or the ancestors of the Wolayta, making any such inference difficult to interpret. Therefore we removed these 6 Wolayta and 6 Gumuz individuals from subsequent analyses. Similar decisions were made for the other exclusions based on these fineSTRUCTURE and CHROMOPAINTER results (e.g. S1 and S2 Figs).

Here we explain our exclusions of 7 labeled “Blacksmith” individuals, and how these exclusions relate to those in the Pagani paper [2]. We started with 18 individuals labeled as “Blacksmith” in the dataset provided to us by the authors of [2]. We retained one Blacksmith individual that appeared from our fineSTRUCTURE analysis to be misclassified as an “Ari” and is instead assigned to our “AFA” group; we note that this individual was removed from [2] for a similar reason and was not included among the 17 Blacksmiths reported in that paper. Therefore ignoring this misclassified individual, the 17 Blacksmiths labeled as “ARIb” in our S1–S3 Figs are the same ones reported in [2]. We then removed 6 “ARIb” based on IBD sharing; these are the same 6 Blacksmiths excluded by [2] for the same reason. Finally, in addition we removed one other “ARIb” that appeared to have a high proportion of Cultivator ancestry (see S3 Fig). Thus in total we used 10 “ARIb” individuals in our final analysis, which are the only ones analysed throughout the remainder of this paper, compared to 11 Blacksmiths in the final analysis of [2].

The final 17 clustered groupings, comprising 1059 individuals, are depicted on Fig 1a, with the sample sizes and description of each population label given in S1 Table.

### Haplotype phasing

All samples were phased jointly using SHAPEIT [20] incorporating the build 37 genetic map combined across populations available at https://mathgen.stats.ox.ac.uk/impute/data_download_1000G_phase1_integrated.html, using an effective population size (“—effective-size”) of 15000 and otherwise default parameters. Phasing was initially performed across 1176 individuals and 659,881 SNPs. Of these, 24 SNPs monomorphic across individuals were removed. The ASW (61 individuals) from the 1000 Genomes Project dataset were excluded from further analysis because they are known to be recently admixed with Africans and Europeans [45], leaving 1115 individuals and 659,857 SNPs prior to quality control measures mentioned described in the previous section that removed additional individuals.

### ADMIXTURE analysis

We ran ADMIXTURE [10] using the 1059 sampled individuals kept after sample exclusions (see below), using several different numbers of clusters *K*. In this analysis, SNPs were thinned such that no two SNPs within 250kb had squared correlation coefficient (i.e. *r* ^2^) greater than 0.1. This left 95,648 SNPs for ADMIXTURE analysis. In order to better visualise the Ari groups, ADMIXTURE results for *K* = 8 are shown for at most 50 individuals for each of the 17 groups in Fig 1c. ADMIXTURE results for *K* = 7 − 11 for all 1059 individuals are shown in S5 Fig.

### Inferring “painting profiles” using CHROMOPAINTER

We ran CHROMOPAINTER to infer “painting profiles” of each individual for the fineSTRUCTURE analysis and each of analyses (A)-(C). In each case, we initially estimated the mutation/emission (“-M”) and switch rate (“-n”) parameters using 10 steps of Expectation-Maximisation (E-M) algorithm (i.e. “-i 10 -in -iM”), starting with default values and running on a subset of individuals for a subset of chromosomes. We then averaged inferred values of each parameter across these chromosomes, weighting the average by number of SNPs, and then across individuals. We then fixed these values (i.e. using “-M” and “-n”) and ran on all chromosomes and all individuals. We otherwise used all default values, except that for the fineSTRUCTURE analysis we set the size of regions (“-k”) in CHROMOPAINTER to 50 in order to infer the “c” parameter in fineSTRUCTURE.

For the initial analysis of all 1115 individuals for use in fineSTRUCTURE, to estimate the emission and switch rates we used at most 20 individuals from each of the 23 labeled populations (or all individuals for populations with fewer than 20) and chromosomes {4, 10, 15, 22}, giving values of 0.00122 and 419.9 for the emission and switch rates, respectively. For the remaining analyses using the 17 fineSTRUCTURE-inferred groups, we used all individuals and chromosomes {1, 4, 15, 22} to estimate the emission and switch rates across all individuals. Under analysis (A) this gave values of 0.00064 and 390.1, under analysis (B) values of 0.0069 and 403.5, and under analysis (C) values of 0.00119 and 457.2 for the emission and switch rates respectively.

We note that individuals are not allowed to copy from themselves, so that e.g. under analysis (A), each of the 10 Ari Blacksmith individuals is allowed to copy from the other 9 Ari Blacksmith individuals and all individuals from each of the other 16 groups, including all 23 Ari Cultivator individuals. Similarly, under analysis (A) each of the 23 Ari Cultivator individuals is allowed to copy from only 22 Ari Cultivator individuals and all individuals from each of the other 16 groups, including all 10 Ari Blacksmith individuals. This slight asymmetry of donor panels potentially makes comparing the Ari Blacksmiths’ and Ari Cultivators’ copying vectors problematic under analysis (A), though we expect it to have only a small effect. In particular, we have found in practice that removing a single donor individual out of a group of ≈10 donor individuals generally results in a slight increase in copying from the remaining donor individuals, i.e. so that the overall copying from the entire group is not much changed.

### Clustering analysis using fineSTRUCTURE

We used fineSTRUCTURE [21] to cluster individuals into genetically homogeneous groups. To do so, we first used CHROMOPAINTER as described above to summarize each of the *N* = 1115 individuals’ ancestries as the total number of haplotype segments they copied from each of the other *N* − 1 individuals, so that we did not use any group label information when clustering. We set the starting value as 1 cluster and then ran fineSTRUCTURE for 1,000,000 “burn-in” iterations of MCMC, followed by another 1,000,000 iterations where we sampled inferred clusterings every 10,000 iterations, otherwise using default values. This inferred *C* = 154 final clusters. We next used fineSTRUCTURE to perform 100,000 additional hill-climbing steps to improve the posterior probability and then merge clusters in a greedy step-wise fashion. In particular, starting from the hill-climbing solution, at each step of the tree-building procedure fineSTRUCTURE considers the merging of all $\left(\begin{array}{c} C\\ 2 \end{array}\right)$ pairwise combinations of clusters, selects the pairwise merging that minimises the decrease in posterior probability over all such combinations, and continues this process until only *C* = 2 clusters remain, building a “tree” of relatedness.

Based on the fineSTRUCTURE tree, we classified the 878 individuals from MKK and 1KGP into ten groups. These ten groups differed from the 11 original population labels in two ways: (i) the two groups from China (CHB,CHS) were merged into a single group, and (ii) 23 individuals from Britain (GBR) were separated into their own group (perhaps representing substructure within Britain) and the remaining GBR individuals were merged with the Utah (CEU) samples.

As the MKK and 1KGP individuals comprised a large proportion of the overall sample set yet were not of direct interest in this analysis, we performed a second fineSTRUCTURE run that attempted to further refine clustering in only the Pagani samples. To do so, we treated our ten non-Pagani groups as “super individuals” (“-F”) in this second fineSTRUCTURE run. This means that each of the ten non-Pagani groups (as well as each Pagani individual) was represented as only a single “individual” containing the average number of haplotype segments they copied from each Pagani individual and each of the ten non-Pagani groups. We clustered this new set containing 237 + 10 = 247 “individuals” using fineSTRUCTURE, as before setting the starting value as 1 cluster and running for 1,000,000 “burn-in” iterations, followed by another 1,000,000 iterations where we sampled inferred clusterings every 10,000 iterations and otherwise using default values. This analysis inferred *C* = 87 clusters (including the ten non-Pagani groups). We considered two independent runs of fineSTRUCTURE using “super individuals” and the final clustering results were very consistent across the two (S4 Fig).

We next performed the re-classification procedure first described in [25]. Briefly for each individual *i* and each MCMC sample *m*, this procedure identifies the individuals clustered with *i* in sample *m* and calculates the proportion of these individuals contained in each of the *c* ∈ [1, …, *C*] final fineSTRUCTURE clusters (e.g. initially *C* = 87 here, with the 10 1KGP+MKK clusters remaining fixed for this procedure). For each cluster *c* ∈ [1, …, *C*] we then average these proportions across all MCMC samples, and then (potentially) re-classify individual *i* to the *c* containing the maximum such average proportion across all clusters *C*. Taking these new re-classifications of all individuals as the new “final fineSTRUCTURE cluster”, we repeat this procedure for 50 iterations. This gave our final classification of *C* = 87 clusters, though we note that cluster assignments were very similar to those prior to this re-classification procedure. As before we then used fineSTRUCTURE to merge clusters in a greedy step-wise fashion and build the fineSTRUCTURE tree.

These final 87 clusters and corresponding tree are shown in S1 Fig. Labels on the axes refer to the code (i.e. “Pop ID” in S1 Table) we assigned each group based on the population labels among individuals in the given cluster. Using this tree, we removed 23 individuals and grouped the remaining 1059 individuals into 17 genetically homogeneous groups for all subsequent analyses, with these 17 groups detailed in S1 Table and denoted by distinct colors on the axes of S1 Fig. Specifically, we first moved down the tree until reaching the level immediately prior to the Blacksmiths splitting into two distinct groups. The clusters resulting from this level of the fineSTRUCTURE tree are shown in S2 Fig. We then removed 23 individuals whose inferred ancestry visually looked different from the other members assigned to their group; these individuals and all other removed individuals are highlighted with translucent vertical grey bars in S1 Fig and with grey vertical dashed lines in S2 Fig. Including inds removed for having high IBD (see above), from left to right in S2 Fig we removed the Ari individual admixed between Cultivators and Blacksmiths (also shown in S3 Fig), 6 further high IBD Ari Blacksmiths, 1 Ari Cultivator (high IBD), 2 Ethiopian Somali individuals (assigned to the “AFA” group), 2 Amhara individuals (assigned to the “AFA” group), 5 Wolayta (assigned to the “ORO” group), 1 Oromo (assigned to the “ORO” group), another Wolayta (assigned to the “ORO” group), 1 Somali individual (assigned to the “SOM” group), 6 Gumuz (assigned to the “GUM” group) and 5 Sudanese (assigned to the “ANU” group). We removed 31 individuals in total from the Pagani dataset. While not all of our ten non-Pagani “super-individuals” were split into distinct groups at the fineSTRUCTURE tree level depicted in S2 Fig, we nonetheless kept all ten separated for subsequent analyses, giving 17 total groups.

### CHROMOPAINTER analyses to infer relative amounts of genetic diversity within groups (i.e. assess evidence of bottleneck effects)

In our additional CHROMOPAINTER analysis that compared the sizes of haplotype segments across groups to assess the relative genetic diversity within each group, we painted each of the 17 world-wide groups using only individuals from their own group as donors. We infered the switch rate separately for each group using 50 steps of E-M algorithm (i.e. “-i 10 -in”) and using the default mutation/emission rate (which was 0.00771). As the expected lengths of segments copied intact from a single donor in the painting can be affected by the number of donor individuals, we used 10 randomly sampled individuals from each group, matching the sample size of our smallest group (“ARIb”). We used all 659,857 SNPs, allowing individuals to copy only from the 9 other individuals of their own group. Our median inferred values across individuals for average haplotype segment size (in cM) and switch rate, plus the 95% empirical quantiles across the 10 individuals, are provided for each group in S14 Table. For each individual, we calculate average segment size by dividing the total proportion of genome-wide DNA copied from all donors by the total expected number of haplotype segments copied from all donors. We note that the often “noisy” process of painting in CHROMOPAINTER [33] suggests exact sizes of haplotype segments should be interpreted with caution and not e.g. related directly to split times as in other approaches [46], though comparing relative sizes across groups is still meaningful.

### Inferring “proportions of ancestry” using CHROMOPAINTER output

Our inferred “painting profiles” from CHROMOPAINTER suffer some limitations. For example *a priori* groups with more individuals will be copied more often when running CHROMOPAINTER, potentially leading to a biased interpretation of results. To cope with this, we use additional linear modeling described in this section to “clean” the raw CHROMOPAINTER inference as in [33, 25].

Following notation in [33], let $f^{j}\equiv \{f_{1}^{j},...,f_{K}^{j}\}$ be the observed “painting profile” inferred by CHROMOPAINTER for recipient group *j*, with $\sum_{k=1}^{K}f_{k}^{j}=1.0$ and $f_{k}^{j}$ the proportion of genome-wide DNA that group *j* paints (or copies) from donor group *k* ∈ [1, …, *K*] using CHROMOPAINTER. We use CHROMOPAINTER to calculate analogous painting profiles for each group *k* ≠ *j* ∈ [1, …, *K*] as described above. To measure the relative amount of drift (or “self-copying”) in group *j*, we introduce a *K*-vector *f* ^j^* with $f_{j}^{j*}=f_{j}^{j}$ and all other entries 0. We “clean” the painting of group *j* using the following linear model:

$$\begin{array}{c} f^{j}=[\sum_{k\neq j}^{K}\beta_{k}^{j}f^{k}]+\beta_{\text{SELF}}^{j}f^{j*}+\epsilon . \end{array}$$ (1)

Here *ϵ* is a vector of errors, and we seek the estimates ${\hat{\beta }}_{1}^{j},\ldots ,{\hat{\beta }}_{K}^{j},{\hat{\beta }}_{{}_{\text{SELF}}}^{j}$ to replace $\beta_{{}^{{}_{1}}}^{j},\ldots ,\beta_{{}^{{}_{K}}}^{j},\beta_{{}^{{}_{\text{SELF}}}}^{j}$, respectively, that minimize *ϵ* using least-squares. (Note that $f_{{}^{{}_{j}}}^{j*}=0$ in analyses (B) and (C) for some groups, such as the Ari, so that $\beta_{{}^{{}_{\text{SELF}}}}^{j}=0$ in these cases.) We use the non-negative-least-squares “nnls” package in R to estimate the $\beta_{{}^{{}_{k}}}^{j}\text{s}$ under the constraints that all ${\hat{\beta }}_{k}^{j}\geq 0$, ${\hat{\beta }}_{{}_{\text{SELF}}}^{j}\geq 0$, and $({\hat{\beta }}_{{}_{\text{SELF}}}^{j}+\overset{K}{\underset{k\neq j}{\sum }}{\hat{\beta }}_{k}^{j})=1.0$. To avoid over-fitting, in practice any ${\hat{\beta }}_{i}^{j}\leq 0.001$ is set to 0, and we then re-scale so that $({\hat{\beta }}_{{}_{\text{SELF}}}^{j}+\overset{K}{\underset{k\neq j}{\sum }}{\hat{\beta }}_{k}^{j})=1.0$. We refer to $\left\{{\hat{\beta }}_{1}^{j},\ldots ,{\hat{\beta }}_{k}^{j}\right\}$ as our inferred “proportions of ancestry” for group *j*.

In Fig 3 (top row) and S11 Fig, our inferred *β*s are shown for each of analyses (A)-(C). To measure uncertainty in the $\hat{\beta }\text{s}$, we take an approach analogous to [34] and calculate standard errors using a weighted Block Jackknife [47] approach that removes each chromosome one-at-a-time (from each group *k* ∈ [1, …, *K*]) and re-calculates the $\hat{\beta }\text{s}$, weighting each jackknife sample by that chromosome’s number of SNPs. This contrasts from the approach used to measure uncertainty in [25], who instead used bootstrap re-samples of individuals’ chromosomes. In contrast to that approach, the jackknife technique we use here accounts for independent drift effects across chromosomes, as we are particularly interested here in mitigating any differences among groups attributable to drift. We report our $\hat{\beta }\text{s}$, plus and minus two standard errors, for all 17 groups in S10–S12 Tables for each of analyses (A)-(C).

### Measuring differences in inferred painting profiles (TVD, *F*_*XY*_)

To compare differences in copying vectors, we use total variation distance (TVD) as in [25]. In particular let $f_{k}^{X}$ be the genome-wide proportion of DNA that recipient *X* copies from donor group *k* ∈ [1, …, *K*] as inferred by CHROMOPAINTER. Then to compare the copying vectors of two recipients *X* and *Y*, we calculate *TVD*_*XY*_, with:

$$\begin{array}{c} TVD_{XY}=0.5\sum_{k=1}^{K}\left|f_{k}^{X}-f_{k}^{Y}\right|. \end{array}$$ (2)

Note that $f_{k}^{X}$ might be the copying vector of a single recipient individual, or the average copying vector across all individuals from a particular recipient group (e.g. the average copying vector across all ARIb individuals). For example, in S15 Table and Fig 3 (bottom row) we report *TVD*_*XY*_ for $f_{k}^{X},f_{k}^{Y}$ the average copying vector across all individuals from the given group. In contrast, for S15 and S16 Figs we report *TVD*_*XY*_ for $f_{k}^{X},f_{k}^{Y}$ the copying vectors of single individuals.

Assessing formally whether the observed value of *TVD*_*XY*_ is significantly different from 0, i.e. assessing the evidence (or *p*-value) for rejecting the null hypothesis that *X* and *Y* are ancestrally related in the same way to other groups, is not straight-forward when trying to negate founder and/or population-specific drift effects. For example, if a small number of individuals leave a relatively large population and form a “new” group, the mean inferred such ancestries between the new and old groups may be significantly different (i.e. *TVD*_*XY*_ >> 0) due to the decreased variance in the smaller group. But this significant difference is not important if one is only interested in genetic differences attributable to ancient relatedness. One way to account for this is to consider the differences in ancestry between the two groups scaled by the differences within each group among independent genetic regions (i.e. regions separated by historical recombination events). Each such region will relate ancestrally to other groups differently due to independent drift effects, i.e. because drift affects any two unlinked segments of the genome independently, and hence can provide a means to measure variation attributable to drift effects. Here we use each chromosome as separate regions.

Let $f_{ik}^{X}$ be the proportion of DNA that recipient *X* copies from donor group *k* ∈ [1, …, *K*] across chromosome *i* as inferred by CHROMOPAINTER. Then we calculate ${\tilde{TVD}}_{X}$ as:

$$\begin{array}{c} {\tilde{TVD}}_{X}=0.5\sum_{i=1}^{22}\frac{L_{i}}{L}[\sum_{k=1}^{K}\left|f_{ik}^{X}-f_{k}^{X}\right|], \end{array}$$ (3)

with *L*_*i*_ the number of SNPs in chromosome *i* ∈ [1, …, 22] and $\sum_{i=1}^{22}L_{i}=L$. We define a new statistic, *F*_*XY*_, to measure the difference between the inferred ancestries of two groups *X* and *Y* relative to the difference between chromosomes *within* each of *X* and *Y*:

$$\begin{array}{c} F_{XY}=TVD_{XY}/\left[0.5\left({\tilde{TVD}}_{X}+{\tilde{TVD}}_{Y}\right)\right]. \end{array}$$ (4)

(This formulation has parallels to *F*_*ST*_ statistics [48].)

Again $f_{{}^{{}_{ik}}}^{X}$ can refer to the copying vector for chromosome *i* in a single individual (i.e. so that *X* refers to an individual) or alternatively can be the average copying vector across chromosome *i* for all individuals from a particular recipient group (i.e. so that *X* refers to a group). For example, in S17 and S18 Figs we report *F*_*XY*_ for $f_{{}^{{}_{ik}}}^{X},f_{{}^{{}_{ik}}}^{Y}$ the copying vectors of single individuals. In contrast, in S16 Table, we report *F*_*XY*_ with $f_{ik}^{X},f_{ik}^{Y}$ the average copying vector across chromosome *i* for all individuals from the given group.

However, this latter version of *F*_*XY*_ also has undesirable properties, again attributable to differential drift effects (and/or different sample sizes) between groups *X* and *Y*. In particular assuming ancestry component *k* for each chromosome of an individual from population *X* is a random draw from some distribution with mean $f_{k}^{X}$, taking the mean across *n*_*X*_ individuals from *X* to calculate $f_{ik}^{X}$ should move $f_{ik}^{X}$ towards the mean $f_{k}^{X}$. Now assume another population *Y* also draws its ancestry components *k* from some distribution with mean $f_{k}^{X}$, but the difference is that *Y* is highly drifted relative to *X*. In this case, for any given chromosome *i* the *n*_*Y*_ individuals from *Y* are much more similar (relative to individuals from *X*) in their ancestry component *k*, so that taking the mean across the *n*_*Y*_ individuals to calculate $f_{ik}^{Y}$ will be some value not necessarily as close to $f_{k}^{X}$. Therefore in this scenario ${\tilde{TVD}}_{Y}>{\tilde{TVD}}_{X}$, as we observed in our “MA” simulations. Sample size differences, i.e. having *n*_*Y*_ << *n*_*X*_, can also lead to the same effect.

One potential approach to assess evidence of whether the difference in inferred ancestry between *X* and *Y* is “significantly different”, analogous to those used by other groups [34], is to divide *TVD*_*XY*_ or *F*_*XY*_ by its standard error calculated using the weighted Block Jackknife [47], weighting by removing e.g. whole chromosomes. However, unlike in [34] it is unclear what the distribution of such a statistic should be, given that *TVD*_*XY*_ ≥ 0 and *F*_*XY*_ ≥ 0 and so cannot follow e.g. a normal distribution with mean 0. Assessing significance of ancestry differences under these approaches in a manner to mitigate founder and group-specific drift effects remains an area of important future work.

Despite these difficulties, when comparing two groups *X* and *Y* we attempt to assess the evidence of a bottleneck (i.e. founder or drift effect) in one of the groups but otherwise very similar ancestry. To do so, we provide an empirical measure of the “significance” of differences in inferred ancestry among sampled individuals within one group (i.e. the non-bottlenecked one) relative to that among individuals between *X* and *Y*. In particular, letting *X* represent the non-bottlenecked group and 1_*Z*_ an indicator that *Z* is true, we calculate:

$$\begin{array}{c} P\left(X\right)=\frac{1}{\left(\begin{array}{c} n_{X}\\ 2 \end{array}\right)}\sum_{j=1}^{n_{X}}\sum_{k=1}^{n_{X}}\left[1_{j\neq k}1_{\left[F_{jk}\geq {\hat{F}}_{XY}\right]}\right], \end{array}$$ (5)

where

$$\begin{array}{c} {\hat{F}}_{XY}\equiv \frac{1}{n_{X}n_{Y}}\sum_{j=1}^{n_{X}}\sum_{k=1}^{n_{Y}}F_{jk}. \end{array}$$ (6)

Values of (Eq 5) range from 0–1 and are provided in Figs 4 and 5 of the main text, and S17, S18 and S24 Tables and S19 and S30 Figs of the SOM. Under a bottleneck in group *Y* but otherwise identical ancestry patterns between *X* and *Y*, or in general if the ancestries of *X* and *Y* are not significantly different, we expect (Eq 5) to be relatively large (i.e. closer to 0.5). In contrast, if the ancestries of *X* and *Y* are significantly different from each other, or possibly if the ancestries are identical except for a strong bottleneck in *X* and not *Y*, we expect (Eq 5) to be at or near 0. We demonstrate the power of this approach to assess evidence of different ancestry between *X* and *Y* in our simplified simulation results under a Remnants (“RN”) setting (S19 Fig, S18 Table), results of which are described in “Results”.

### Clustering analyses using CHROMOPAINTER output

We developed a new algorithm to cluster individuals into genetically homogeneous groups according to their CHROMOPAINTER inferred paintings. We did this clustering separately for analyses (A)-(C).

Similar to the algorithm implemented in fineSTRUCTURE [21], but without the restriction that each individual must be painted in CHROMOPAINTER using all other sampled individuals as donors, we cluster based on the inferred genome-wide length of DNA copied from each donor. (We note clustering in fineSTRUCTURE [21] is done based on the inferred counts of DNA segments rather than lengths of DNA copied, though in practice clustering based on the inferred lengths often provides similar results.) In particular, let $l^{j}\equiv \{l_{1}^{j},...,l_{K}^{j}\}$ be the observed CHROMOPAINTER painting for recipient individual *j*, with $l_{k}^{j}$ the centimorgan (cM) length of genome-wide DNA that individual *j* paints (or copies) from donor group *k* ∈ [1, …, *K*]. Here $\sum_{k=1}^{K}l_{k}^{j}$ equal to the total genome length of DNA in cM (or double that for diploid individuals), and note that $f_{k}^{j}\equiv l_{k}^{j}/[\sum_{i=1}^{K}l_{i}^{j}]$.

We want to cluster our *N* individuals into *C* sub-groups, where *C* is fixed (also in contrast to fineSTRUCTURE, which infers *C* in its current implementation). Let *ψ*_*j*_ ∈ [1, …, *C*] be the cluster assignment for individual *j*, with each cluster equally likely *a priori*. Let *γ* ≡ {*γ* ^1^, …, *γ* ^*C*^}, with $\gamma^{c}\equiv \{\gamma_{1}^{c},...,\gamma_{K}^{c}\}$ and $\gamma_{k}^{c}$ the probability of being painted by donor group *k* if assigned to cluster *c*. Note $\sum_{k=1}^{K}\gamma_{k}^{c}=1$. We assume:

$$\begin{array}{cc} \left(l^{j}|\gamma^{c},\psi_{j}=c\right)∼\text{Multinomial}\left(\gamma_{1}^{c},...,\gamma_{K}^{c}\right) & \text{for}\;j=1,...,N\;\text{and}\;c=1,...,C,\\ \left(\gamma^{c}|\delta \right)∼\text{Dirichlet}\left(\delta ,...,\delta \right) & \text{for}\;c=1,...,C,\\ \mathrm{Pr}(\psi_{j}=c)=1/C & \text{for}\;c=1,...,C, \end{array}$$

with *δ* controlling the probability distribution of *γ* ^*c*^. Intuitively, a larger value of *δ* makes the parameters of the *C* clusters more similar to one another, hence discouraging sub-group formation.

We wish to sample the cluster assignments *ψ*_*j*_ for *j* ∈ [1, …, *N*] based on their posterior probabilities conditional on *l* ≡ {*l* ^1^, …, *l* ^*N*^}. We do so using the following Markov Chain Monte Carlo (MCMC) technique. We start with initial cluster assignments *ψ*(0) ≡ {*ψ*_1_(0), …, *ψ*_*N*_(0)} by assigning each of the *N* individuals randomly to one of the *C* clusters.

Then for *m* = 1, …, *M*:

1. Sample *γ*(*m*) using a Gibbs step, with
  $$\left(\gamma^{c}\left(m\right)|l,\psi \left(m-1\right),\delta \right)∼\text{Dirichlet}(\delta +\sum_{j=1}^{N}l_{1}^{j}1_{\left[\psi_{j}\left(m-1\right)=c\right]},...,\delta +\sum_{j=1}^{N}l_{K}^{j}1_{\left[\psi_{j}\left(m-1\right)=c\right]}),$$
  for *c* = 1, …, *C*, with 1_*S*_ an indicator for whether *S* is true.
2. Sample *ψ*(*m*) using a Gibbs step, with
  $$\mathrm{Pr}\left(\psi_{j}\left(m\right)=c|l^{j},\gamma \left(m\right)\right)=\frac{\mathrm{Pr}(l^{j}|\gamma^{c}\left(m\right),\psi_{j}=c)}{\sum_{i=1}^{C}\mathrm{Pr}\left(l^{j}|\gamma^{i}\left(m\right),\psi_{j}=i\right)},$$
  for *j* = 1, …, *N*.
3. If any of the *c* = 1, …, *C* clusters contain 0 individuals, we randomly assign a single distinct individual to each empty cluster. This enables the cluster painting probabilities in step (A) to move away from the prior distribution defined by *δ*.

For numerical stability, any $\gamma_{k}^{c}$ values < 1*e* ^−7^ or > (1 − 1*e* ^−7^) in step (A) were set to 1*e* ^−7^ and (1 − 1*e* ^−7^), respectively, with the values in *γ* ^*c*^ subsequently re-scaled to sum to 1.

For large *M*, this algorithm is guaranteed to converge to the true posterior distribution of the *ψ*_*j*_’s (e.g. [49]). In practice, for all results presented here we use *M* = 2,000,000, sampling every 10,000th iteration after an initial “burn-in” of 1,000,000 iterations. Also, for all analyses we combined results across ten independent runs of the above procedure.

When clustering the Ari individuals only, we set *C* = 2 and present our cluster results for analyses (A)-(C) in S19 Table, including analogous results for the simulations, separately for two choices of the prior value *δ* = {50, 100}. When clustering all 206 Pagani individuals (after sample exclusions), we used *δ* = 100 and considered several fixed number of clusters *C* = {2, 3, 4, 5, 6, 7, 8, 9, 10, 11, 12, 13, 14, 15, 16, 17, 18, 19, 20, 50}, providing results for a subset of these values in S20–S22 Figs.

### Dating recent admixture events using GLOBETROTTER

We used GLOBETROTTER to identify, describe and date any putative admixture events occurring within the last ≈4,500 years in the Ari groups, using painting results from analyses (A)-(C) and closely following the application of GLOBETROTTER described in [33]. In short, CHROMOPAINTER identifies the segments of DNA within each Ari individual’s genome that are most closely related ancestrally to each of the *S* “surrogate” groups. I.e. *S* = 16 for analysis (A) since your own group is excluded as a surrogate group, while *S* = 15 for analysis (B) and *S* = 10 for analysis (C). GLOBETROTTER then measures the decay of linkage disequilibrium (LD) versus genetic distance among the segments copied from a given pair of surrogate groups. Assuming a single “pulse” of admixture between two or more distinct admixing source groups, theoretical considerations predict that this decay will be exponentially distributed with rate equal to the time (in generations ago) that this admixture occurred [13]. GLOBETROTTER jointly fits an exponential distribution to the decay curves for all $\left(\begin{array}{c} S\\ 2 \end{array}\right)+S$ pairwise combinations of the *S* surrogate groups (including where both surrogates in the pair are the same group) and determines the single best fitting rate, hence dating the admixture event. Instead of requiring specific genetic surrogates to represent each admixing source group involved in the admixture, which is necessary for other dating approaches such as ROLLOFF [34], GLOBETROTTER aims to infer each source group as a linear combination of the DNA of sampled groups (i.e. as a linear combination of the *S* groups). In the case of *X* distinct pulses of admixture occuring at different times, the decay of LD among segments is expected to be a mixture of *X* independent exponential distributions. GLOBETROTTER tests for evidence of two distinct admixture events by fitting two exponential distributions jointly to all curves and determining the best fitting rates for each. If two exponential distributions fit the data “significantly” better than a single event (assessed via simulations described in [33]), GLOBETROTTER infers the source groups involved in each of the two events.

We applied GLOBETROTTER to infer any admixture event(s) separately in the ARIb and ARIc. When doing so, we used 10 painting samples from each haploid of each Ari individual. Note that under analysis (A), following [33] we repainted individuals within each Ari group excluding members of their own group as donors to get these painting samples, though we note that (also following [33]) the original painting used elsewhere in this paper (e.g. for Fig 3) was also used here to determine mixing coefficients in GLOBETROTTER analysis (A). Define a “chunk” within one of these painting samples to be a segment of contiguous DNA painted intact from a single donor haploid from one of *D* donor groups. (For each analysis here, the donor groups were the same as the surrogate groups, so that *D* = *S*.) For every pairing of painting samples between and within each Ari individual’s two haploids, we pairwise compared each chunk on one sample to each chunk on the other sample, tabulating the donor group(s) represented at each chunk in the pair, the product of the two chunks’ sizes in cM (with any sizes > 1cM set to 1) and the genetic distance between the two chunks’ midpoints. Then for every pair of donor groups *D*_1_ and *D*_2_, for each 0.1cM bin *g* ∈ [0.1, …, 50cM] we sum the products across all chunk pairs separated by *g* for which one chunk is painted by *D*_1_ and one by *D*_2_. We repeat this for all pairings *D*_1_, *D*_2_ of the *D* donor groups, and re-scale these counts by inferred ancestry coefficients for each of the *S* surrogate groups (i.e. the ${\hat{\beta }}_{k}^{j}$ values from “inferring mixing coefficients using CHROMOPAINTER output”) as described in [33], subsequently removing any surrogate groups with ${\hat{\beta }}_{k}^{j}<0.001$. We refer to the plot of these re-scaled counts versus genetic distance *g* as the “coancestry curves” for surrogate groups *S*_1_, *S*_2_, of which there are $\left(\begin{array}{c} S\\ 2 \end{array}\right)+S$ in total.

We plot these coancestry curves for *S*_1_, *S*_2_ = {ANU, ARIb, ARIc, ORO, SOM, TSI} for analysis (A) (S23 and S24 Figs), *S*_1_, *S*_2_ = {ANU, AFA} for analysis (B) (S25 Fig) and *S*_1_, *S*_2_ = {CEU, MKK} for analysis (C) (S26 Fig). On these plots, we also include our best-fitting exponential distribution assuming a single pulse of admixture (green lines), the rate of which corresponds to our inferred date of admixture (in generations from present). We furthermore include our best-fitting exponential distribution assuming two distinct pulses of admixture (red lines), i.e. the sum of two exponential distributions whose rates correspond to our inferred dates for each event. For these figures and all reported results, we used five iterations of GLOBETROTTER’s alternating source composition and admixture date inference, followed by 100 bootstrap re-samples of individuals’ chromosomes to infer confidence intervals (CIs) around our date estimates. Also, for all results presented here we standardized each coancestry curve by a “NULL” individual designed to eliminate any spurious linkage disequilibrium patterns not attributable to that expected under a genuine admixture event, which simulations suggest is particularly important when the target population has undergone a strong bottleneck (see [33] for details). This gave a *p*-value < 0.01 testing for evidence of at least one admixture event in each of the ARIb and ARIc under each of analyses (A)-(C), defined as in [33] as the proportion of bootstrap re-samples with an inferred single date = 1 or ≥ 400. We note that under each analysis, each Ari group was inferred to have only a single pulse (i.e. date) of admixture, though we provide results assuming multiple dates of admixture in S22 Table for comparison. For this multiple-date inference, we excluded results if CIs of the two inferred dates overlapped or if the recent data had a point estimate of 1, resulting in the exclusion of analysis (C) results for both Ari groups. In contrast, analyses (A)-(B) suggest visual evidence of multiple admixture dates (i.e. compare red to green lines in S23–S25 Figs), which GLOBETROTTER may not have enough power to detect using the given sample sizes. When there is evidence of only two admixing source groups, we provide the best genetically matching sampled group to each source and the mixing coefficients (for surrogates with coefficients > 10%) that provide a more detailed description of each source as a mixture of sampled groups (Table 1 and S20–S22 Tables). When there is evidence of more than two admixing source groups at a single date, we provide both the best genetically matching sampled groups to each source, the mixing coefficients, and the sampled groups that reflect the greatest difference between inferred ancestries for the two depicted sources (S20–S22 Tables; see [33] for details).

### Inferring genetic diversity within segments inherited from different admixing source groups

To infer which segments in the Ari were inherited from the “African” and “non-African” (i.e. “West Eurasian”) admixing sources as inferred by GLOBETROTTER, we used CHROMOPAINTER to paint each Ari haploid genome using only the YRI and CEU as donors (using the “-b” switch, which produces the file with suffix .*copyprobsperlocus.out*). While we could have used TSI or IBS instead of CEU to represent the “non-African” source (which may in fact originate from e.g. the Levant [2]), we were concerned that each of TSI and IBS may have recent admixture from Africa [33] which would diminish accuracy in separating the “African” and “non-African” segments. In contrast, CEU should not have any recent admixture from Africa. Similarly YRI should not have received any recent admixture from outside Sub-Saharan Africa.

We used two different approaches to assign segments to CEU/YRI, which we term “E-M” and “NNLS”.

#### “E-M” approach to assign local ancestry

For the first approach (“E-M”), we painted each haploid Ari genome *i* using 50 steps of the CHROMOPAINTER Expectation-Maximization (E-M) algorithm (i.e. “-i 50 -in -iM -ip”) to jointly infer—separately within each chromosome—the switch and emission rates and the average probability of copying from each donor group. We then took $p^{i_{l}}(D)$, the final probability of copying from each donor group *D* ∈ {CEU,YRI} at each SNP *l* in Ari haploid *i* under this approach, to be our final data; i.e.

$$\begin{array}{c} \mathrm{Pr}\left(D\;\text{at}\;l\right)=p_{l}^{i}\left(D\right). \end{array}$$ (7)

#### “NNLS” approach to assign local ancestry

For the second approach (“NNLS”), we painted the Ari, YRI, and CEU using the YRI and CEU as donors, with the usual restriction that individuals are not allowed to copy from themselves. To do so, we fixed the switch and emission rates to 403.5 and 0.00069, respectively, (i.e. “-n 403.5 -M 0.00069”) based on values inferred using E-M as described before but using data from all chromosomes. Using the painting profiles for CEU, YRI and the Ari groups (averaged across all individuals within each label), for each of the ARIb and ARIc we inferred the average proportion of ancestry related to the CEU and YRI in a manner analogous to that described above. This analysis assumes these values are constant across all individuals within the ARIb and ARIc, respectively. Using the same notation as before, let ${\hat{\beta }}_{\text{CEU}}^{\text{ARIb}}$, ${\hat{\beta }}_{\text{YRI}}^{\text{ARIb}}$ be the inferred proportion of ancestry from CEU and YRI, respectively, in the ARIb, with analogous notation for the ARIc. Also as before, let $f_{X}^{D}$ be the average proportion of DNA across all individuals in group *D* that is painted by donor group *X*. Then for the ARIb, the genome-wide probability of the true ancestry being *D* ∈ {CEU,YRI} given you are painted by *X* ∈ {CEU,YRI} is:

$$\text{Pr}\left(D|\text{copy}\;X\;\text{in}\;\text{ARIb}\right)={\left(\underset{j\in \left\{\text{CEU,YRI}\right\}}{\sum }f_{X}^{j}{\hat{\beta }}_{j}^{\text{ARIb}}\right)}^{-1}f_{X}^{D}{\hat{\beta }}_{D}^{\text{ARIb}},$$

with an analogous expression for the ARIc. Therefore the probability that the true ancestry is *D* ∈ {CEU,YRI} at a given SNP *l* of haploid ARIb genome *i* given the SNP is painted with *X* ∈ {CEU,YRI} at *l* is:

$$\begin{array}{c} \mathrm{Pr}\left(D\;\text{at}\;l|\text{copy}\;X\;\text{at}\;l\;\text{in}\;\text{hap}\;i\right)=\sum_{X\in \{\text{CEU,YRI}\}}[\mathrm{Pr}(D|\text{copy}\;X\;\text{in}\;\text{ARIb})\mathrm{Pr}(\text{copy}\;X\;\text{at}\;l\;\text{in}\;\text{hap}\;i)], \end{array}$$ (8)

again with an analogous expression for ARIc, and with Pr (copy *X* at *l* in hap *i*) the expected probability of being painted by group *X* at SNP *l* in ARIb haploid *i* as inferred by CHROMOPAINTER when painting the Ari using only the CEU and YRI as donors using the switch and emission rates mentioned above.

#### Simulations of recent admixture to test accuracy in identifying local ancestry

To assess the ability of CHROMOPAINTER to identify segments inherited by admixing sources similar to those thought to have contributed to modern-day Ethiopians [2, 11], we simulated individuals as mixtures of the DNA from 8 Bantu South Africa and 10 Saudi individuals taken from the phased data in [33]. We then painted our simulated individuals using DNA from 21 Yoruba and 28 French individuals to act as surrogates for the admixing groups, again using the phased data from [33]. As these data have a slightly smaller number of SNPs (474,491) compared to our own study (659,857), we note that performance may be better for the real data relative to these simulations due to the increased SNP density.

Following the protocol followed in [33, 50, 51, 52], we simulated 20 haploid genomes as mixtures of 60% Bantu South Africa and 40% Saudi DNA, with the mixture simulated to occur 120 generations ago. Briefly to make each simulated haploid: we (i) sampled a centimorgan distance *g* from an exponential distribution with rate 120/100, then (ii) composed the first *g* centimorgans of the simulated haploid with the corresponding DNA from a random Bantu haploid with probability 0.6 and otherwise a random Saudi haploid, and (iii) repeated this process until an entire haploid genome was simulated. We then ran the “E-M” and “NNLS” techniques described above, though using a switch rate of 400000/(2 * 21 + 2 * 28) = 170.068 and default mutation rates under the “NNLS” approach. We present results here combined across all 20 simulated haploids for chromosome 2, though note that all 22 autosomes were used to infer the genome-wide proportions of ancestry (i.e. $\hat{\beta }\text{s}$) as described above for the “NNLS” approach.

S32 Fig shows the proportion of SNPs inferred correctly as the “non-African” source, i.e. that in truth were simulated using Saudi DNA, among DNA segments containing varying numbers of contiguous SNPs inferred as CEU using different thresholds of Eq (7) under the “E-M” approach. Similarly, S31 Fig shows the proportion of correct calls among DNA segments containing contiguous SNPs inferred as CEU at different thresholds of Eq (8) under the “NNLS” approach. Based on these simulation results, we decided to only include segments within each painted Ari haploid that contained at least 100 contiguous SNPs called as CEU with a threshold > 0.94 for “E-M” and (in a separate analysis) a threshold > 0.66 for “NNLS”, which corresponds to cases where approximately all SNPs were correctly assigned to the “non-African” source.

#### Assessing genetic similarity within segments inferred as CEU and YRI

After identifying all segments with at least 100 consecutive SNPs inferred as CEU (analogously YRI) at the appropriate thresholds under the “E-M” (> 0.94) or “NNLS” (> 0.66) analyses, we calculated the genetic similarity among these segments between each pair of Ari individuals. To do so, we first paired one of the two haploids from one individual with one of the two haploids from the other. We next found all SNPs among CEU (analogously YRI) segments passing our threshold that overlapped among these two haploids. We repeated this for all $\left(\begin{array}{c} 2\\ 1 \end{array}\right)\times \left(\begin{array}{c} 2\\ 1 \end{array}\right)=4$ pairings of haploids from distinct individuals in the pair. Finally we found the proportion out of all such SNPs whose allele types matched, which we report for all pairings of individuals in S33 Fig and summarize across subsets of individual pairings in Table 2. Note that $\phi_{I}^{k}$ and $\phi_{A}^{k}$ in Fig 6 and S34 Fig denote these proportions for pairings of ARIb or ARIc individuals, with *I* = CEU and *A* = YRI.

### Simulations using MaCS

#### “Full” simulations

We also tested our model using the approximate coalescence simulation software MaCS [26]. Under the “full” simulations, we simulated 13 populations under four separate histories, which we refer to as the Marginalisation (“MA”) and three Remnants (“RN”) simulations, depicted in Fig 2a. For both simulation scenarios, 100 generations (gens) denotes the split between Pop8 and Pop9; 375 gens the split between Pop11 and Pop12; 400 gens the split of Pop8/Pop9 and Pop10; 500 gens the split of Pop2 and Pop3; 700 gens the split of Pop5 and Pop6; 1000 gens the split of Pop5/Pop6 and Pop7, as well as the split of Pop8/Pop9/Pop10 and Pop11/Pop12; 1700 gens the split of Pop4 and Pop5/Pop6/Pop7; 1800 gens the split of Pop2/Pop3 and Pop4/Pop5/Pop6/Pop7; 2500 gens the split of Pop2/Pop3/Pop4/Pop5/Pop6/Pop7 and Pop8/ Pop9/Pop10/Pop11/Pop12; and 4000 gens the split of Pop1 and all other populations.

While it is impossible to simulate a scenario that perfectly captures the history of modern human groups, with references in the literature on appropriate split times and population sizes often uncertain or disagreeing, we followed [33] and tried to capture major features of world-wide human migrations and splits, informing our scenario using a number of recent publications [36, 35, 53, 54, 55]. For example, in Fig 2a, populations 1–7 are meant to mimic genetic diversity among African populations [36]. The split at 2500 generations ago and subsequent bottleneck in populations 8–12 mimics the “out-of-Africa” event [36, 35, 55], and the split between populations 8–10 and populations 11–12 at 1000 generations ago mimics the split between Western Eurasia populations and East Asian populations [54, 53], with a subsequent bottleneck in the latter. We also included continuous symmetric migration between populations 11 and 12 such that each population’s fraction of new migrants increased by 0.00025 each generation for 375 generations (continuing until present-day), in order to test our model’s robustness to a scenario of stable long-term migration between nearby groups. To mimic the proposed admixture from outside of Africa [2], we also include one-way admixture from (presumed unsampled) population 10 into populations 5 and 6 (also population 5b in the “Remnants” model discussed below), such that the fraction of new migrants from Pop10 into these groups increased by 0.02 for 10 generations, starting 100 generations ago. Thus the total proportion of admixture from Pop10 should be ≈20% in present-day samples from these groups. Overall diversities in these groups are meant to mimic our sampled populations, e.g. in having similar *F*_*ST*_ values (S4–S7 Tables).

Population 5 and 5b are meant to mimic the Cultivators and Blacksmiths, respectively. The “MA” and “RN” simulation scenarios differ only in how Pop5b relates to the 12 other simulated groups. In the Marginalisation simulations (Fig 2a-(i)), Pop5b splits from Pop5 relatively recently at 20 generations ago, after which Pop5b undergoes a severe instantaneous bottleneck that reduces its effective population size from 20,000 to 200 until present-day. In the Remnants simulations (“RN”, “RN+BN”, “RN+BN+80%”; Fig 2a-(ii)), Pop5b instead splits 1700 generations ago from the ancestors of Pop5, with both groups maintaining an effective population size of 20,000. An exception to this is that two of the Remnants “full” simulations (“RN+BN”, “RN+BN+80%”) also include a bottleneck in Pop5b that starts 20 generations ago and continues until present-day, as in the “MA” simulations, but with a reduction in population size from 20,000 to 500. Also under each of the three “Remnants” models, Pop5b contributes migrants to Pop5 at a rate of 0.005 (“RN”, “RN+BN”) or 0.008 (“RN+BN+80%”) beginning at 300 and ending at 200 generations ago, so that ≈50% (“RN”, “RN+BN”) or ≈80% (“RN+BN+80%”) of Pop5 consists of migrants from Pop5b over this time frame. These parameters were chosen so that *F*_*ST*_ between Pop5b and Pop5, which is 0.025 in the “MA” simulations and ranges from 0.013–0.022 across the three “RN” “full” simulations, were similar to the *F*_*ST*_ = 0.23 between the ARIb and ARIc in the real data. In addition, under all three “Remnants” models Pop5b contributes migrants to Pop6 at a rate of 0.002 over the time period 200 to 300 generations ago, so that ≈20% of Pop6 should consist of migrants from Pop5b over this time.

Due to the large memory requirements of running MaCS with this number of populations and individuals, we simulated 20 independent regions of size 64Mb each, but with variation in recombination rates based on HapMap Phase 2 build 36 genetic maps (www.hapmap.org) for chromosomes 1 to 20, respectively (i.e. such that the HapMap total genome-wide variation in recombination rate for each chromosome was condensed or expanded into a 64Mb region). We assumed a genome-wide average recombination rate of 1.25 × 10^−8^, a mutation rate of 4 × 10^−8^ per basepair per generation, and sampled 200 haplotypes from each of the 13 populations. For each of the 20 regions, we subsequently selected 14,225 SNPs (284,500 SNPs in total) such that the minor allele frequency spectrum of our simulated dataset (across all populations) matched that of our real dataset (across all populations) across 100 equally spaced bins from 0 to 0.5. (We randomly selected SNPs to fill bins where we did not have enough such candidate SNPs.) We provide pairwise *F*_*ST*_ values among all 13 populations for the Marginalisation sims in S4 Table and for the Remnants sims in S5 Table, which reinforce that our simulation scenarios exhibit genetic diversity levels similar to those among our sampled populations (see S3 Table). For all analyses considered below, we treated phase as known and sampled varying numbers of individuals from each population as shown in S2 Table, the latter again roughly mimicking our real data collection.

For each simulation scenario, we performed three CHROMOPAINTER analyses similar to those we did on our real data. The first analysis ((A); “all-donors”) painted each sampled individual using every other sampled individual, excluding themselves, as a potential donor. The second ((B); “non-Ari-donors”) painted each sampled individual using every other sampled individual except those from populations 5 and 5b (i.e. the two “Ari” groups). The third analysis ((C); “non-Pagani-donors”) painted each sampled individual using only sampled individuals from populations 1, 2, 3, 7, 8, 9, 11 and 12, excluding themselves, as donors (i.e. the “Pagani”-like populations 4, 5, 5b, and 6 were excluded as donors). To save computational time, for analyses depicted in Fig 5 and S12 Fig we used the default CHROMOPAINTER mutation/emission rate (“-M” switch) and fixed the CHROMOPAINTER switch rate (“-n” switch) to 400000 divided by the total number of donor haplotypes considered for each analysis. When calculating *F*_*XY*_ and jack-knife values for ancestry proportion standard errors, we gave each of the 20 chromosomes an identical weighting as they were all simulated to have the same number of SNPs.

We also applied ADMIXTURE [10] to each simulation scenario, including the sampled individuals from all 12 groups excluding Pop10. As with our real data ADMIXTURE analysis, SNPs were thinned such that no two SNPs within 250kb had squared correlation coefficient (i.e. *r* ^2^) greater than 0.1. This left 63,599 SNPs for the Marginalisation simulations and 63,382 SNPs for the Remnants analysis. Results for each when using several different fixed numbers of clusters *K* are shown in S6 Fig.

#### “Simplified” simulations

We also generated a set of “simplified” simulations (Fig 2b), consisting of only 7 populations simulated under a “Remnants” hypothesis, to assess the power of our analyses to distinguish two groups that are anciently related but have experienced substantial one-way migration between them (i.e. from “ARIb” into “ARIc”) and a bottleneck in one of the groups (i.e. “ARIb”). These simulations closely followed components of our “full” simulations. For example, we included an “out-of-Africa” split between Pops 1–5 and Pop6 occurring 2500 generations ago, followed immediately by a bottleneck in “non-Africa” Pop6 that reduced its population size from 10000 to 2000 for 500 generations before it increased to 10000 again. All remaining “African” populations experienced an increase in population size from 10000 to 20000 starting 1800 generations ago and continuing to present-day (with the exception of Pop5b—see below). Furthermore, among these “African” populations, Pops 1–2 split from Pops 3–5 1800 generations ago, Pop3 split from Pops 4–5 1700 generations ago, and Pop1 and Pop2 split 500 generations ago. To mimic admixture from a “West Eurasia” source into some of our “Africa” groups, we included one-way migration from Pop6 into Pops 4–5 starting 100 generations ago and lasting for 10 generations, such that 40% of Pop4 and 20% of Pop5 and Pop5b were comprised of migrants from Pop6 over this time period.

For the populations that we particularly focus on, Pop4 (meant to reflect the ORO in our real data) and Pop5 (meant to reflect the ARIc) split 700 generations ago, which gave values of *F*_*ST*_ between Pop4 and Pop5 that are similar to that between ORO and ARIc in our real data (see *F*_*ST*_ values between all pairs of populations in these simulations in S8 Table). In addition, Pop5b (meant to reflect the ARIb) contributed migrants to Pop4 over the period 200 to 300 generations ago, such that 10% of Pop4 is comprised of Pop5b migrants over this time period. We varied the split times between Pop5 and Pop5b to {750, 800, 900, 1000, 1100, 1200, 1300, 1700} generations ago. We then choose corresponding bottleneck lengths in Pop5b, reflecting the current marginalised status of the Blacksmiths, that gave *F*_*ST*_ values between Pop5 and Pop5b that closely match that observed among our real data ARIc and ARIb. For the split times above, this gave bottleneck lengths in Pop5b starting at {40, 40, 35, 35, 35, 30, 30, 20} generations ago, respectively, and continuing until present-day. In each case, the bottleneck reduced the population size of Pop5b to 5000. Finally, for each of the above split time and bottleneck pairs, we simulated three different rates of one-way migration from Pop5b to Pop5 occurring from 200 to 300 generations ago, such that {50, 75, 90%} of Pop5 was comprised of migrants from Pop5b over this time period.

We sampled {50, 50, 25, 50, 25, 15, 50} individuals from {Pop1,Pop2,Pop3,Pop4,Pop5,Pop5b,Pop6}, respectively. We performed two CHROMOPAINTER analyses analogous to (A) and (B) applied to the real data, where for analysis (A) each individual used all other sampled individuals as donors (excluding themselves) and for analysis (B) each individual used all other sampled individuals except those from Pop5 and Pop5b as donors. In each of (A) and (B), only 10 Pop5b and 23 Pop5 individuals were painted to mimic the sample sizes of the ARIb and ARIc, respectively, though all individuals from each group were used as donors in (A). When running CHROMOPAINTER, as in the “full” simulations we used the default CHROMOPAINTER mutation/emission rate (“-M” switch) and fixed the CHROMOPAINTER switch rate (“-n” switch) to 400000 divided by the total number of donor haplotypes considered for each analysis. We calculated *TVD*_*XY*_, *F*_*XY*_ and *P*(*X*) in the same manner as previously described. Results for these “simplified” simulations are shown in S10, S13 and S19 Figs and S18 Table.

#### Additional “simplified” simulations to mimic admixture into Ari from a genetically similar group

We performed 6 further “simplified” simulations under a Marginalisation hypothesis which incorporate an additional population Pop5a designed to represent an unsampled Ethiopian population, which split at the same time from both Ari groups (which were assumed to form one group at the time), and subsequently contributed DNA to the single common Ari group (15% admixture over 5 gens beginning 70 gens ago; see S27 Fig). As in the “MA” “full” simulations, immediately after splitting from Pop5, Pop5b (meant to represent a simulated ARIb) undergoes a severe bottleneck reducing the effective population size from 20,000 to 500 until present day. Consistent with the other “simplified” simulations, we included one-way migration from Pop6 into Pops 4–5 starting 100 generations ago and lasting for 10 generations. In these simulations we varied the split times between Pop5a and Pop5 to {300, 400, 500} generations ago and the bottleneck lengths in Pop5b to {30, 35} generations ago, giving *F*_*ST*_ values similar to that between the ORO, ARIb and ARIc in our real data (S27 Fig, S23 Table). In all of our analyses, we assumed Pop5a was not sampled, in order to reflect a scenario under our CHROMOPAINTER analysis (A) where each of Pop5 and Pop5b likely would have to use the other as the best surrogate for this introgressing group.

As in the “simplified” simulations we sampled {50, 50, 25, 50, 25, 15, 50} individuals from {Pop1,Pop2,Pop3,Pop4,Pop5,Pop5b,Pop6}, respectively. We performed two CHROMOPAINTER analyses analogous to (A) and (B) applied to the real data, where for analysis (A) each individual used all other sampled individuals as donors (excluding themselves) and for analysis (B) each individual used all other sampled individuals except those from Pop5 and Pop5b as donors. For each we used default CHROMOPAINTER mutation/emission rates. In each of (A) and (B), only 10 Pop5b and 23 Pop5 individuals were painted to mimic the sample sizes of the ARIb and ARIc, respectively, though all individuals from each group were used as donors in (A). *TVD*_*XY*_, *F*_*XY*_ and *P*(*X*) were calculated as before, with the results shown in S28, S29 and S30 Figs and S24 Table.

We also conducted an additional analysis, (A-), which took the results from analysis (A) but—separately when analysing each of Pop5 and Pop5b—subtracted out self-copying within that group, to mimic the way GLOBETROTTER analysis (A) infers proportions and source groups. Specifically, separately for *x* ∈ {Pop5,Pop5b}, we set $f_{x}^{j}=0$ for each of the *j* ∈ [1, …, *K*] sampled populations (where *K* = 7 here), rescaled so that $\sum_{k=1}^{K}f_{k}^{j}=1.0$ for *j* ∈ [1, …, *K*], and then inferred the $\beta_{k}^{x}\text{s}$ in the linear model of (Eq 1) after fixing $\beta_{\text{SELF}}^{x}=0$. The results for (A-) in S29 Fig show the ${\hat{\beta }}_{k}^{x}\text{s}$ under this setting for *k* ≠ *x* ∈ [1, …, *K*].

## Supporting Information

S1 Table Sampled groups and fineSTRUCTURE cluster assignments. The 17 groups used in this analysis, with groups defined using clustering assignments from fineSTRUCTURE [21]. The fourth column gives the number of individuals from each population label contained in the given group.
(PDF)

S2 Table Numbers of sampled individuals in each “full” simulation scenario. Number of sampled individuals per simulated group for both the Marginalisation and Remnants “full”simulations.
(PDF)

S3 Table Pairwise *F*_*ST*_ among 17 sampled groups. Pairwise *F*_*ST*_ [48] values among 17 sampled groups based on fineSTRUCTURE clusters (see S1 Table), as shown in Fig 1B of main text.
(PDF)

S4 Table Pairwise *F*_*ST*_ among all populations analysed in “MA” “full” simulations. Pairwise *F*_*ST*_ [48] values among all populations in the “MA” “full” simulations of Fig 2a, i.e. mimicking the Marginalisation model with Pop5 and Pop5b splitting 20 generations ago, with a subsequent bottleneck in Pop5b.
(PDF)

S5 Table Pairwise *F*_*ST*_ among all populations analysed in “RN” “full” simulations. Pairwise *F*_*ST*_ [48] values among all populations in the “RN” “full” simulations of Fig 2a, i.e. mimicking the Remnants model with Pop5 and Pop5b splitting 1700 generations ago and migrants from Pop5b comprising 50% of Pop5 over the period 200 to 300 generations ago.
(PDF)

S6 Table Pairwise *F*_*ST*_ among all populations analysed in “RN+BN” “full” simulations. Pairwise *F*_*ST*_ [48] values among all populations in the “RN+BN” “full” simulations of Fig 2a, i.e. mimicking the Remnants model with Pop5 and Pop5b splitting 1700 generations ago, migrants from Pop5b comprising 50% of Pop5 over the period 200 to 300 generations ago, and a subsequent bottleneck in Pop5b.
(PDF)

S7 Table Pairwise *F*_*ST*_ among all populations analysed in “RN+BN+80%” “full” simulations. Pairwise *F*_*ST*_ [48] values among all populations in the “RN+BN+80%” “full” simulations of Fig 2a, i.e. mimicking the Remnants model with Pop5 and Pop5b splitting 1700 generations ago, migrants from Pop5b comprising 80% of Pop5 over the period 200 to 300 generations ago, and a subsequent bottleneck in Pop5b.
(PDF)

S8 Table Pairwise *F*_*ST*_ among all populations analysed in “simplified” simulations. Pairwise *F*_*ST*_ [48] values among all populations in the “simplified” simulations of Fig 2b, i.e. mimicking the Remnants model with Pop5 and Pop5b splitting 750–1700 generations ago, a subsequent bottleneck of varying strength in Pop5b, and migrants from Pop5b comprising 50–80% of Pop5 over the period 200 to 300 generations ago. The minimum and maximum *F*_*ST*_ values between each population pair across all “simplified” simulations is given.
(PDF)

S9 Table CHROMOPAINTER inferred average haplotype segment sizes for Ari sampled groups and “full” simulations. Median and 95% empirical quantiles for the average sizes of haplotype segments (in cM) that match to a single donor individual, across recipient individuals from the ARIc and ARIb under CHROMOPAINTER analyses (A)-(C). Also shown are corresponding values for simulated “Ari” groups Pop5/Pop5b under the Marginalisations (MA) and Remnants (RN) models in the “full” simulations.
(PDF)

S10 TableInferred proportions of ancestry for all-donors analysis (A).Inferred proportions of ancestry (i.e. $\hat{\beta }\text{s}$) for all-donors analysis (A), plus and minus two standard errors calculated using a weighted block jackknife approach.(PDF)

S11 TableInferred proportions of ancestry for non-Ari-donors analysis (B).Inferred proportions of ancestry (i.e. $\hat{\beta }\text{s}$) for non-Ari-donors analysis (B), plus and minus two standard errors calculated using a weighted block jackknife approach.(PDF)

S12 TableInferred proportions of ancestry for non-Pagani-donors analysis (C).Inferred proportions of ancestry (i.e. $\hat{\beta }\text{s}$) for non-Pagani-donors analysis (C), plus and minus two standard errors calculated using a weighted block jackknife approach.(PDF)

S13 Table PI_HAT homogeneity estimates using PLINK. Median PI_HAT values across all pairwise combinations of individuals within each group as calculated using PLINK v1.07 [28], with 95% empirical quantiles given in parenthesis. Results are shown when analysing all SNPs (“unpruned”) or a dataset where SNPs were pruned to remove those in high linkage disequilibrium (*r* ^2^ > 0.2) in a sliding 250 SNP window using the command –*indep-pairwise 250 10 0.2*.
(PDF)

S14 Table CHROMOPAINTER’s inferred average haplotype segment sizes for within-group painting. CHROMOPAINTER’s inferred average haplotype segment sizes (in cM) copied intact from a single donor, and corresponding number of SNPs per segment and switch rate (with emission rate fixed at 0.00771 for all groups), when allowing 10 randomly-sampled individuals from each group to copy from the other 9 individuals with the same label, using 50 steps of Expectation-Maximisation (E-M). Median and empirical quantile values across the 10 individuals are given for each group. The segment sizes capture (roughly) the relative amount of haplotype diversity in each group. Note the high values for the ARIb relative to all other groups, including the ARIc.
(PDF)

S15 Table *TVD*_*XY*_ between all Pagani group pairwise comparisons under analyses (A)-(C). *TVD*_*XY*_ scores (see Methods) measuring the difference in inferred ancestry between each pairing of Pagani groups under analyses (A)-(C); these values are described with a heatmap in Fig 3. Note for analysis (C) that no pairing has a lower value than that of the ARIb and ARIc (in red), suggesting the two Ari groups share more common ancestry, as related to the non-Pagani-donors, than any other pairing of Pagani groups.
(PDF)

S16 Table *F*_*XY*_ between all Pagani group pairwise comparisons under analyses (A)-(C). *F*_*XY*_ scores (see Methods) measuring the difference in inferred ancestry between each pairing of Pagani groups under analyses (A)-(C). Note for analysis (C) that no pairing has a lower value than that of the ARIb and ARIc (in red), suggesting the two Ari groups share more common ancestry, as related to the non-Pagani-donors, than any other pairing of Pagani groups.
(PDF)

S17 Table Proportion of *F*_*XY*_ greater among within-group comparisons versus between group comparisons, under analysis (C). The proportion of pairwise *F*_*XY*_ scores under analysis (C) between individuals from the same group (rows) that are greater than or equal to the mean *F*_*XY*_ across all pairwise combinations of individuals with one from the group given in the row and the other from the group given in the column.
(PDF)

S18 Table Proportion of *F*_*XY*_ greater among within-group comparisons versus between group comparisons in “simplified” simulations. The proportion of pairwise *F*_*XY*_ scores under analysis (B) between individuals from the same group (i.e. either Pop5b or Pop5, as given in the columns above, which are meant to represent the “ARIb” and “ARIc”, respectively) that are greater than or equal to the mean *F*_*XY*_ across all pairwise combinations of individuals with one from Pop5b and the other from Pop5. Results from each of 24 “simplified” simulations are given, which vary in the number of generations ago Pop5 and Pop5b split (“split”), the number of generations Pop5b is bottlenecked (“BN”), and the proportion of migrants contributed from Pop5b to Pop5 (“% admixture”).
(PDF)

S19 Table Clustering the ARIb and ARIc individuals based on their inferred ancestries under analyses (A)-(C). Proportion of MCMC samples for which all ARIb individuals are clustered separately from all ARIc individuals, when inferring two clusters under our MCMC clustering algorithm for each of analyses (A)-(C). The median and range across 10 independent runs of the MCMC chain are shown. Analogous results are given for the “full” simulations: “MA”, “RN”, “RN+BN”, and “RN+BN+80%”. Results are shown for two different choices of prior value *δ* (see Methods).
(PDF)

S20 Table GLOBETROTTER’s inferred dates and admixing sources for analyses (A), (A-sim), (B) and (C). GLOBETROTTER’s inferred dates (both generations and years from present, with bootstrap 95% CIs given in parenthesis), admixing sources (single best matching sampled surrogate is given first, followed by mixing proportions > 10% giving more precise inference on the haplotype make-up of the source; see Methods), and proportion (%) of admixture contributed from each source for inferred admixture events in the Ari groups under analyses (A), (A-sim), (B) and (C). Here (A-sim) refers to a simplified GLOBETROTTER (A) analysis where the surrogates specified are TSI, ANU, ORO and {ARIb, ARIc}. “Props” gives more stably estimated source compositions than the mixing coefficients when multi-way admixture is inferred (as described in [33]) and are reported when the value is > 0.05. Assuming a generation time of 28 years, generations *g* were converted to years *y* using the formula: *y* = 1950 − (*g* + 1) × 28.
(PDF)

S21 Table GLOBETROTTER’s inferred dates and admixing sources for analyses (A*), (B*) and (C*) when only 10 ARIc are used. GLOBETROTTER’s inferred dates (both generations and years from present, with bootstrap 95% CIs given in parenthesis), admixing sources (single best matching sampled surrogate is given first, followed by mixing proportions > 10% giving more precise inference on the haplotype make-up of the source; see Methods), and proportion (%) of admixture contributed from each source for inferred admixture events in the Ari groups under analyses (A*),(B*) and (C*). (A*), (B*) and (C*) give the inferred dates and admixing sources under each analysis (A), (B) and (C) when only 10 ARIc individuals are used. “Props” gives more stably estimated source compositions than the mixing coefficients when multi-way admixture is inferred (as described in [33]) and are reported for sources where the value is > 0.05. Assuming a generation time of 28 years, generations *g* were converted to years *y* using the formula: *y* = 1950 − (*g* + 1) × 28.
(PDF)

S22 TableGLOBETROTTER’s inferred dates and admixing sources assuming two dates of admixture for analyses (A), (A-sim) and (B).GLOBETROTTER’s inferrence when assuming two distinct dates of admixture, with date estimates (both in generations and years from present, with bootstrap 95% CIs given in parenthesis), admixing sources (single best matching sampled surrogate is given first, followed by proportions (>10%) giving more precise inference on the haplotype make-up of the source), and proportion (%) of admixture contributed from each source for each admixture event in the Ari groups under analyses (A), (A-sim) and (B). “Props” gives more stably estimated source compositions than the mixing coefficients where multiple-date admixture is inferred (as in [33]) and are reported for the groups with the two maximal proportions. Here *R*_*E*_ refers to the goodness-of-fit (i.e. *R* ^2^; see [33] for details) of the model assuming *E* = 1,2 distinct dates of admixture, and $R^{{*}_{2}}$ refers to the additional proportion of remaining fit explained by adding by adding the second date. We note that no analysis here suggests significant evidence of multiple dates of admixture. Assuming a generation time of 28 years, generations *g* were converted to years *y* using the formula: *y* = 1950 − (*g* + 1) × 28.(PDF)

S23 Table Pairwise *F*_*ST*_ among Pop4, Pop5 and Pop5b analysed in simulations including Pop5a. Pairwise *F*_*ST*_ [48] between Pop4, Pop5 and Pop5b under a simulation scenario including Pop5a. Simulations differ in the split time (split*) between Pop5 and Pop5a and the length of the Pop5b bottleneck (BN), both in generations (see S27 Fig). In these simulations, {Pop4,Pop5,Pop5b} are meant to mimic the {ORO,ARIc,ARIb}, respectively
(PDF)

S24 Table Proportion of *F*_*XY*_ greater among within-group comparisons versus between group comparisons, in simulations including Pop5a under analysis (B). For simulations under the MA model with an unsampled contributing population 5a, the proportion of pairwise *F*_*XY*_ scores under analysis (B) between individuals from the same group (i.e. either Pop5b or Pop5, as given in the columns above, which are meant to represent the “ARIb” and “ARIc”, respectively) that are greater than or equal to the mean *F*_*XY*_ across all pairwise combinations of individuals with one from Pop5b and the other from Pop5. Each of 6 simulations are given, which vary in the number of generations ago Pop5a and Pop5 split (“split*”) and the number of generations Pop5b is bottlenecked (“BN”).
(PDF)

S25 Table *D*-statistics testing {ARIc,ARIb;X,Y}. *D*-statistics testing {ARIc,ARIb;X,Y}, i.e. testing whether {ARIc,ARIb} form a clade relative to {X,Y} using the program qpDstat with default settings, with the corresponding standardized *Z* statistic showing the strength of evidence for rejecting this tree structure (see [34] for details). We tested all combinations of {X,Y} ∈ {ANU,GUM,LWK,YRI}, as these were the only sampled African groups with < 5% inferred ancestry from a West Eurasian source according to [32]. Note that no test gives ∣*Z*∣ > 3, suggesting the tree structure {ARIc,ARIb;X,Y} is a good fit to the data in these cases.
(PDF)

S1 Fig fineSTRUCTURE heatmap with clusters and tree. CHROMOPAINTER’s inferred proportion of genome-wide DNA that each of 87 fineSTRUCTURE-inferred clusters (columns) copy from these 87 clusters (rows). Clusters containing any of the Pagani individuals removed from analysis are highlighted with translucent grey vertical bars. The tree at top shows fineSTRUCTURE’s inferred hierarchical merging of these 87 clusters, and colors on the axes show which clusters were assigned to the 17 groups in S1 Table used for analyses. The two Ari groups (ARIb, ARIc) are highlighted with the blue rectangle. The groups highlighted with green rectangles give examples of merged clusters containing 6 Wolayta (left) and 6 Gumuz (right) individuals that were removed due to appearing intermixed among divergent groups (see Methods).
(PDF)

S2 Fig CHROMOPAINTER inferred painting profiles under fineSTRUCTURE analysis. CHROMOPAINTER’s inferred painting profiles for all individuals in the fineSTRUCTURE analysis, showing the proportion of genome-wide DNA that each individual (column) copies from each of the 17 groups (color) in S1 Table (group labels on x-axis, with “1KGP” denoting the ten non-Pagani groups, i.e. the MKK and nine 1000 Genomes groups; color codes in Fig 1 of main text). Grey dashed vertical lines denote excluded individuals; the proportions of DNA copied from any of these individuals are colored in white. Black vertical bars separate the fineSTRUCTURE clusters inferred at a level of the tree where there are *K* = 15 total clusters, which is the level immediately prior to the Blacksmiths (ARIb) separating into two distinct clusters. (Note that we split some 1KGP clusters from this level and removed one of the 15 clusters to get the 17 final groupings we use in our analyses here.)
(PDF)

S3 Fig CHROMOPAINTER inferred painting heatmap for Ari individuals under fineSTRUCTURE analysis. CHROMOPAINTER’s inferred proportion of genome-wide DNA that each Ari individual (column) copies from every other Ari individual (row), for all Ari individuals prior to sample exclusions (except one “Blacksmith” individual that clustered with genetically different groups under fineSTRUCTURE). One of the Ari individuals, labeled a “Blacksmith” (ARIb) and highlighted with the green ellipse, shows clear mixture between Blacksmiths and Cultivators, suggesting that this individual is recently descended from individuals of each of these groups.
(PDF)

S4 Fig Pairwise coincidence for two independent fineSTRUCTURE runs. Proportion of MCMC samples for which each individual (or “super individual”) was clustered with each other individual in the fineSTRUCTURE analysis under two independent fineSTRUCTURE runs (separated by the bottom-left to top-right diagonal). Black ticks along each axis denote the midpoints of each of the *K* = 87 final inferred clusters for one of these fineSTRUCTURE runs.
(PDF)

S5 Fig ADMIXTURE analysis of sampled groups for several numbers of clusters *K*. ADMIXTURE applied to individuals from the seven Pagani groups [2], the MKK and groups from the 1000 Genomes Project using various numbers of clusters *K*; labels are based on the 17 inferred fineSTRUCTURE groups. The two Ari groups (ARIb, ARIc) are highlighted with the blue rectangle.
(PDF)

S6 Fig ADMIXTURE results for “full” simulations. ADMIXTURE results for “full” simulations under the (a) “Marginalisation (MA)”, (b) “Remants (RN)”, (c) “Remnants + Bottleneck (RN+BN)” and (d) “Remnants + Bottleneck + 80% Mixture (RN+BN+80%)” models (see Methods), with “ARIc” = Pop5 and “ARIb” = Pop5b, including all individuals and using various numbers of cluster *K*. Pop5 and Pop5b are highlighted with the blue rectangle. Note that Pop5 looks like a mixture of ancestries related to Pop5b and other populations for various *K* across all four simulations (e.g. for *K* = 11).
(PDF)

S7 Fig CHROMOPAINTER inferred painting profiles for all Ari individuals. CHROMOPAINTER’s inferred painting profiles for each Ari individual, showing the proportion of DNA copied from each world-wide donor group (color), under each of analyses (A)-(C). Group labels (ARIb/ARIc) are given on the x-axis, with group means (E[ARIb],E[ARIc]) at far right. Donor groups are colored according to Fig 1a of the main text.
(PDF)

S8 Fig CHROMOPAINTER averaged inferred painting profiles, ARIb versus ARIc. Average proportion of DNA that the ARIb (x-axis) and ARIc (y-axis) copy from each donor group under CHROMOPAINTER analyses (A)-(C). Inner 95% empirical quantiles across individuals within each Ari group are shown with the vertical and horizontal lines. Donor groups are colored according to Fig 1a of the main text.
(PDF)

S9 Fig CHROMOPAINTER inferred painting profiles for simulated “Ari” individuals in “full” simulations. CHROMOPAINTER’s inferred painting profiles for each simulated “Ari” individual in the four different “full” simulations (see Methods), showing the proportion of DNA copied from each simulated donor group (color), under each of analyses (A)-(C). Group labels (Pop5b = “ARIb” / Pop5 = “ARIc”) are given on the x-axis, with group means (E[Pop5b],E[Pop5]) at far right. Donor groups are colored according to x-axis labels in S12 Fig.
(PDF)

S10 Fig CHROMOPAINTER inferred painting profiles for simulated “Ari” individuals in “simplified” simulations. CHROMOPAINTER’s inferred painting profiles for each simulated “Ari” individual in 15 different “simplified” simulations (see Methods), showing the proportion of DNA copied from each simulated donor group (color). Group labels (Pop5b = “ARIb” / Pop5 = “ARIc”) are given on the x-axis, with group means (E[Pop5b],E[Pop5]) at far right. The titles above each plot describe the simulation parameters, which vary in the number of generations ago Pop5 and Pop5b split (“split”), the number of generations Pop5b is bottlenecked (“BN”), and the proportion of migrants contributed from Pop5b to Pop5 (%). Results are shown for CHROMOPAINTER analysis (B) using all non-Pop5 groups only as donors. Each distinct color denotes the inferred painting from a distinct group, with the legend for each non-Pop5 group given in the third bar of the top left plot of S13 Fig.
(PDF)

S11 Fig Inferred proportions of ancestry for real data. Proportions of ancestry each Pagani and 1KGP+MKK group (column) shares with other groups, matching Fig 3 of main text as inferred using CHROMOPAINTER and linear modelling (color codes in Fig 1a of main text). The two Ari groups are enclosed in a blue border. Under analysis (A) including all groups as donors, the ARIb have “self-copying” levels higher than any other group, indicative of strong drift effects. Under analyses that exclude the Ari groups as donors (B) or exclude all non-Pagani groups as donors (C), the ARIb and ARIc look genetically similar when ignoring this drift, suggesting recent shared ancestry.
(PDF)

S12 Fig Inferred proportions of ancestry for “full” simulations. Proportions of ancestry, as inferred using CHROMOPAINTER and linear modelling, for simulations under the Marginalisation (MA; top row) and three different Remants (RN; rows 2–4) models in the “full” simulations (see Methods) for each of CHROMOPAINTER analyses (A)-(C), with groups Pop5 = “ARIc” and Pop5b = “ARIb” enclosed in a blue border. Note that the MA model (top row) shows nearly identical inference for Pop5 and Pop5b under CHROMOPAINTER in analyses (B) and (C), contrasting with the three RN models.
(PDF)

S13 Fig Inferred proportions of ancestry for “simplified” simulations. Proportions of ancestry, as inferred using CHROMOPAINTER and linear modelling, for simulations under the simplified Remnants simulations (see Methods). Pop5b is meant to represent “ARIb” and Pop5 is meant to represent “ARIc”. The titles above each plot describe the simulation parameters, which vary in the number of generations ago Pop5 and Pop5b split (“split”), the number of generations Pop5b is bottlenecked (“BN”), and the proportion of migrants contributed from Pop5b to Pop5 (%). Results are shown for CHROMOPAINTER analyses using (A) all available groups as donors to describe ancestry and (B) using all non-Pop5 groups only. Each distinct color denotes the inferred ancestry from a distinct group, with the legend for each non-Pop5 group given in the third bar of the top left plot.
(PDF)

S14 Fig Inferred average haplotype segment sizes for within-group painting versus PLINK PI_HAT estimates. CHROMOPAINTER’s inferred average haplotype segment sizes (in cM) from S14 Table, i.e. when allowing individuals to copy only from members of their own group, versus PI_HAT values inferred by PLINK v1.07 [28] from S13 Table for the same groups. Median and 95% empirical quantile values across all individuals (“segment size”) or across all pairwise comparisons of individuals (“PI_HAT”) are shown. Each technique measures homogeneity within a group, though note that the haplotype-based technique clearly separates African and non-African groups, with the exception of the likely bottlenecked Blacksmiths (ARIb), while ignoring haplotypes leads to several non-African groups having lower values than African groups, likely due to ascertainment bias. These patterns were similar when inferring PI_HAT values after sub-sampling SNPs to decrease linkage disequilibrium levels (S13 Table).
(PDF)

S15 Fig *TVD*_*XY*_ between pairs of Ari individuals. *TVD*_*XY*_ between every pair of Ari individuals *X*, *Y*, with group labels (ARIb/ARIc) on the axes, under each of analyses (A)-(C).
(PDF)

S16 Fig *TVD*_*XY*_ between pairs of “full” simulation individuals. *TVD*_*XY*_ between every pair of individuals *X*, *Y* simulated under the Marginalisation (MA; top row) and Remnants (RN; rows 2–4) models in the “full” simulations, with group labels (Pop5/Pop5b) on the axes, under each of analyses (A)-(C).
(PDF)

S17 Fig *F*_*XY*_ between pairs of Ari individuals. *F*_*XY*_ between every pair of Ari individuals *X*, *Y*, with group labels (ARIb/ARIc) on the axes, under each of analyses (A)-(C).
(PDF)

S18 Fig *F*_*XY*_ between pairs of “full” simulation individuals. *F*_*XY*_ between every pair of individuals *X*, *Y* simulated under the Marginalisation (MA; top row) and Remnants (RN; rows 2–4) models in the “full” simulations, with group labels (Pop5/Pop5b) on the axes, under each of analyses (A)-(C).
(PDF)

S19 Fig Differences in inferred ancestry for pairs of simulated “Ari” individuals under analysis (B) in the “simplified” simulations. Differences in inferred ancestry under analysis (B) between all pairings of simulated “ARIb” individuals (Pop5b, pink), all pairings of simulated “ARIc” individuals (Pop5, green), and all pairings of one “ARIb” and one “ARIc” individual (cyan), for 15 of the “simplified” simulations. In each plot the black vertical line gives the mean difference across the pairings of one “Pop5b” and one “Pop5”, with *P*(Pop5b), *P*(Pop5) giving the proportion of Pop5b and Pop5 pairings, respectively, with a difference greater than or equal to this mean.
(PDF)

S20 Fig Clustering of all individuals’ inferred ancestries under analysis (A). Proportion of MCMC samples for which each pair of Pagani dataset individuals were clustered together when fixing various numbers of clusters *C*, using inferred ancestries from the “all-donors” CHROMOPAINTER analysis (A). Ari individuals are enclosed in a green border. Results are averaged over ten different runs of the MCMC chain.
(PDF)

S22 Fig Clustering of all individuals’ inferred ancestries under analysis (B). Proportion of MCMC samples for which each pair of Pagani dataset individuals were clustered together when fixing various numbers of clusters *C*, using inferred ancestries from the “non-Ari-donors” CHROMOPAINTER analysis (B). Ari individuals are enclosed in a green border, and are not separated when *C* = 50. Results are averaged over ten different runs of the MCMC chain.
(PDF)

S22 Fig Clustering of all individuals’ inferred ancestries under analysis (C). Proportion of MCMC samples for which each pair of Pagani dataset individuals were clustered together when fixing various numbers of clusters *C*, using inferred ancestries from the “non-Pagani-donors” CHROMOPAINTER analyses (C). Ari individuals are enclosed in a green border, and are not separated when *C* = 50. Results are averaged over ten different runs of the MCMC chain.
(PDF)

S23 Fig GLOBETROTTER coancestry curves under analysis (A). GLOBETROTTER coancestry curves under all-donors analysis (A) for the ARIb (top row) and the ARIc (rows 2–3). Black lines give the (scaled) probability that two DNA segments within the Ari group are inferred as most ancestrally related to the two donor groups given in the title (y-axis) versus the genetic distance between the two segments’ midpoints (x-axis). Green lines give the best fitting exponential distributions to the black lines assuming a single date of admixture, and red lines give the best fit assuming two distinct dates of admixture. Note in the ARIc that the coancestry curves for each pairwise combination of {ARIb,ANU,ORO} increase with increasing genetic distance, suggesting clear evidence of admixture from three distinct source groups around the same time.
(PDF)

S24 Fig GLOBETROTTER coancestry curves under simplified analysis (A-sim). GLOBETROTTER coancestry curves under simplified all-donors analysis (A-sim) for the ARIb (top row) and the ARIc (bottom row) using ANU, TSI, ORO and {ARIc,ARIb} as surrogates. Black lines give the (scaled) probability that two DNA segments within the Ari group are inferred as most ancestrally related to the two donor groups given in the title (y-axis) versus the genetic distance between the two segments’ midpoints (x-axis). Green lines give the best fitting exponential distributions to the black lines assuming a single date of admixture, and red lines give the best fit assuming two distinct dates of admixture.
(PDF)

S25 Fig GLOBETROTTER coancestry curves under analysis (B). GLOBETROTTER coancestry curves under non-Ari-donors analysis (B) for the ARIb (top row) and the ARIc (bottom row). Black lines give the (scaled) probability that two DNA segments within the Ari group are inferred as most ancestrally related to the two donor groups given in the title (y-axis) versus the genetic distance between the two segments’ midpoints (x-axis). Green lines give the best fitting exponential distributions to the black lines assuming a single date of admixture, and red lines give the best fit assuming two distinct dates of admixture.
(PDF)

S26 Fig GLOBETROTTER coancestry curves under analysis (C). GLOBETROTTER coancestry curves under non-Pagani-donors analysis (C) for the ARIb (top row) and the ARIc (bottom row). Black lines give the (scaled) probability that two DNA segments within the Ari group are inferred as most ancestrally related to the two donor groups given in the title (y-axis) versus the genetic distance between the two segments’ midpoints (x-axis). Green lines give the best fitting exponential distributions to the black lines assuming a single date of admixture, and red lines give the best fit assuming two distinct dates of admixture.
(PDF)

S27 Fig Schematic describing additional simulations under the “MA” model that include Pop5a. Eight populations simulated under the Marginalisation (MA) model with an additional group Pop5a (shown in grey) providing admixture from an unsampled “Ethiopian” group. Orange arrows indicate migration from Pop6 into Pop4 and Pop5, and the grey arrow indicates migration from Pop5a into Pop5. Pop5 and Pop5a split at varying times *t* ∈ {300, …, 500}, with a bottleneck in Pop5b occurring {30, 35} generation ago. These parameters were chosen to give *F*_*ST*_ values similar to those observed between the ARIb, ARIc and ORO in the real data (see S23 Table).
(PDF)

S28 Fig CHROMOPAINTER inferred painting profiles for simulated “Ari” individuals in simulations including Pop5a. CHROMOPAINTER’s inferred painting profiles for each simulated “Ari” individual in 6 different simulations under the MA model incorporating an unsampled contributing population Pop5a (see Methods), showing the proportion of DNA copied from each simulated donor group (color). Group labels (Pop5b = “ARIb” / Pop5 = “ARIc”) are given on the x-axis, with group means (E[Pop5b],E[Pop5]) at far right. The titles above each plot describe the simulation parameters, which vary in the number of generations ago Pop5a and Pop5 split (“split*”) and the number of generations Pop5b is bottlenecked (“BN”). Results are shown for CHROMOPAINTER analyses using (B) using all non-Pop5 groups only as donors. Each distinct color denotes the inferred painting from a distinct group, with the legend for each non-Pop5 group given in the third bar of the top left plot of S13 Fig.
(PDF)

S29 Fig Inferred proportions of ancestry for simulations including Pop5a. Inferred proportions of ancestry for each simulated “Ari” individual in a simulation scenario under the MA model incorporating an unsampled contributing population 5a (see S27 Fig), as inferred using CHROMOPAINTER and additional linear modelling. The simulations vary in the number of generations Pop5a and Pop5 split (“split*”), and the length of subsequent bottleneck (“BN”) in Pop5b. Results are shown for CHROMOPAINTER analyses using all-donors (A), all-donors after removing self-copying (A-), and using all non-Pop5 groups as donors (B) (see Methods). Each distinct color denotes the inferred painting from a distinct group, with the legend for each non-Pop5 group given in the third bar of the top left plot of S13 Fig. Note that the inferred ancestry proportions under analysis (A-), which is analogous to the proportion and soruce estimation technique used in GLOBETROTTER analysis (A), illustrate that the inferred contribution of Pop5b to Pop5 is much less than the reverse, even though the same group (Pop5a) contributed equally to each.
(PDF)

S30 Fig Differences in inferred ancestry for pairs of simulated “Ari” individuals under analysis (B) in the simulations including Pop5a. Differences in inferred ancestry under analyses (B) between all pairings of simulated “ARIb” individuals (Pop5b, pink), all pairings of simulated “ARIc” individuals (Pop5, green), and all pairings of one “ARIb” and one “ARIc” individual (cyan), for simulations under an MA model that incorporate an unsampled contributing population 5a (see S27 Fig). In each plot the black vertical line gives the mean difference across the pairings of one “Pop5b” and one “Pop5”, with *P*(Pop5b), *P*(Pop5) giving the proportion of Pop5b and Pop5 pairings, respectively, with a difference greater than or equal to this mean. The simulations vary in the number of generations Pop5a and Pop5 split (“split*”) and the number of generations Pop5b is bottlenecked (“BN”).
(PDF)

S31 Fig Accuracy of local ancestry assignment to CEU in admixture simulations using “NNLS” approach. The proportion of SNPs whose true simulated local ancestry is Saudi (y-axis) among segments that have *X* contiguous SNPs (x-axis) all inferred as CEU under the CHROMOPAINTER “NNLS” approach. Each color represents a different threshold for confidently calling a SNP as CEU (see legend), which is meant to act as a surrogate for Saudi. Vertical solid lines show the range, dots the median, and crosses the mean of proportions across all segments whose number of contiguous CEU-inferred SNPs falls between a given pair of consecutive dashed lines (these SNP number bins are on a log_10_ scale). The number of segments falling within each SNP number bin are given at top for each threshold value; only chromosome 2 was simulated.
(PDF)

S32 Fig Accuracy of local ancestry assignment to CEU in admixture simulations using “E-M” approach. The proportion of SNPs whose true simulated local ancestry is Saudi (y-axis) among segments that have *X* contiguous SNPs (x-axis) all inferred as CEU under the CHROMOPAINTER “E-M” approach. Each color represents a different threshold for confidently calling a SNP as CEU (see legend), which is meant to act as a surrogate for Saudi. Vertical solid lines show the range, dots the median, and crosses the mean of proportions across all segments whose number of contiguous CEU-inferred SNPs falls between a given pair of consecutive dashed lines (these SNP number bins are on a log_10_ scale). The number of segments falling within each SNP number bin are given at top for each threshold value; only chromosome 2 was simulated.
(PDF)

S33 Fig Distributions of genetic similarity scores, separately within segments matched to YRI and CEU. The distributions of genetic similarity scores, separately within segments matched to CEU (top row) and YRI (bottom row), across all pairwise comparisons of individuals within the ARIb (pink), within the ARIc (green), and with one ARIb and one ARIc individual (cyan). Results are shown for segments assigned to CEU and YRI using (left) the E-M model with a threshold of 0.94, and (right) the NNLS model with a threshold of 0.66. The solid black vertical line gives the average across all pairwise combinations of one ARIb and one ARIc individual, with dotted black lines giving the 95% empirical quantile. Note that the ARIb have higher similarity scores within each of the CEU and YRI segments, as expected from bottleneck effects. In addition, differences between ARIb and ARIc pairs are very similar to those among ARIc pairs, consistent with identical non-African and African ancestral sources in each Ari group.
(PDF)

S34 FigDistributions of $\left(\phi_{I}^{\text{ARIb}}/\phi_{A}^{\text{ARIb}}\right)$ and $\left(\phi_{I}^{\text{ARIc}}/\phi_{A}^{\text{ARIc}}\right)$ for each Ari group under NNLS approach.The distributions of $\left(\phi_{I}^{\text{ARIb}}/\phi_{A}^{\text{ARIb}}\right)$ and $\left(\phi_{I}^{\text{ARIc}}/\phi_{A}^{\text{ARIc}}\right)$ (see text and Fig 6a) across all pairwise comparisons of individuals within each Ari group, for segments inferred as from the introgressing (*I*) or ancestral (*A*) sources using the NNLS model with a threshold of 0.66 and assuming *I* is the “West Eurasian” source (see Methods). As in the corresponding Fig 6b that instead used the E-M model to infer segments’ sources, the strong similarity in distributions is consistent with the introgression occurring before the split.(PDF)
