# Supplementary material for: Evidence for a Common Origin of Blacksmiths and Cultivators in the Ethiopian Ari within the Last 4500 Years: Lessons for Clustering-Based Inference
Source: PLoS Genet. 2015 Aug 20;11(8):e1005397. doi: 10.1371/journal.pgen.1005397 (PMC4546361; doi:10.1371/journal.pgen.1005397)
Supplement: S1 Table — The 17 groups used in this analysis, with groups defined using clustering assignments from fineSTRUCTURE [21]. The fourth column gives the number of individuals from each population label contained in the given group. (PDF) [file pgen.1005397.s001.pdf]

Pop ID	Description of cluster	Sample Size	Finestructure Clusters
CEU	British and Utah in Europe	149	GBR:64<br>CEU:85
GBR	British in Britain	21	GBR:21
CHI	Chinese in China	188	CHS:91<br>CHB:97
FIN	Finnish in Finland	93	FIN:93
IBS	Iberian populations in Spain	14	IBS:14
JPT	Japanese in Tokyo, Japan	89	JPT:89
LWK	Luhya form Webuye, Kenya	87	LWK:87
TSI	Tuscans in Italy	98	TSI:98
YRI	Yoruba in Ibadan, Nigeria	88	YRI:88
MKK	HapMap Masaii from Kenya	26	MKK:26
ARIb	South Omo, AriBlacksmiths	10	AriBlacksmith:10
ARIc	South Omo, AriCultivators	23	AriCultivator:23
AFA	Wag Hemra Zone	69	Afar:12<br>Amhara:24<br>AriBlacksmith:1<br>Gumuz:1<br>Oromo:10<br>Tigray:21
ORO	Oromia and Wolayta regions	12	Oromo:10<br>Wolayta:2
SOM	Somali Peninsula and Somali region	37	E.Somali:15<br>Somali:22
GUM	Beni-Shangul, Gumuz	13	Gumuz:13
ANU	Gambella and South Sudanese regions	42	Anuak:23<br>Sudanese:19
