# Supplementary material for: Evidence for a Common Origin of Blacksmiths and Cultivators in the Ethiopian Ari within the Last 4500 Years: Lessons for Clustering-Based Inference
Source: PLoS Genet. 2015 Aug 20;11(8):e1005397. doi: 10.1371/journal.pgen.1005397 (PMC4546361; doi:10.1371/journal.pgen.1005397)
Supplement: S2 Table — Number of sampled individuals per simulated group for both the Marginalisation and Remnants “full”simulations. (PDF) [file pgen.1005397.s002.pdf]

**pop**	**number of inds**	**pop**	**number of inds**
**Pop1**	25	**Pop7**	25
**Pop2**	75	**Pop8**	100
**Pop3**	95	**Pop9**	100
**Pop4**	60	**Pop10**	0
**Pop5**	25	**Pop11**	100
**Pop5b**	15	**Pop12**	75
**Pop6**	100		
