# Supplementary material for: Evidence for a Common Origin of Blacksmiths and Cultivators in the Ethiopian Ari within the Last 4500 Years: Lessons for Clustering-Based Inference
Source: PLoS Genet. 2015 Aug 20;11(8):e1005397. doi: 10.1371/journal.pgen.1005397 (PMC4546361; doi:10.1371/journal.pgen.1005397)
Supplement: S3 Table — Pairwise F ST [48] values among 17 sampled groups based on fineSTRUCTURE clusters (see S1 Table), as shown in Fig 1B of main text. (PDF) [file pgen.1005397.s003.pdf]

	**YRI**	**LWK**	**MKK**	**ANU**	**GUM**	**ARIb**	**ARIc**	**ORO**	**SOM**	**AFA**	**TSI**	**IBS**	**CEU**	**GBR**	**FIN**	**CHI**	**JPT**
**YRI**	0	0.008	0.028	0.024	0.043	0.066	0.048	0.05	0.056	0.062	0.15	0.151	0.154	0.156	0.157	0.182	0.183
**LWK**	0.008	0	0.018	0.016	0.033	0.056	0.038	0.039	0.046	0.051	0.138	0.14	0.143	0.145	0.146	0.172	0.174
**MKK**	0.028	0.018	0	0.016	0.026	0.04	0.02	0.012	0.018	0.02	0.095	0.097	0.101	0.102	0.104	0.14	0.142
**ANU**	0.024	0.016	0.016	0	0.02	0.052	0.033	0.036	0.041	0.048	0.143	0.144	0.147	0.149	0.15	0.175	0.176
**GUM**	0.043	0.033	0.026	0.02	0	0.048	0.03	0.037	0.045	0.049	0.139	0.141	0.144	0.145	0.146	0.173	0.174
**ARIb**	0.066	0.056	0.04	0.052	0.048	0	**0.023**	0.036	0.048	0.047	0.124	0.126	0.13	0.131	0.133	0.167	0.168
**ARIc**	0.048	0.038	0.02	0.033	0.03	**0.023**	0	0.015	0.027	0.026	0.102	0.104	0.108	0.109	0.111	0.146	0.148
**ORO**	0.05	0.039	0.012	0.036	0.037	0.036	0.015	0	0.007	0.002	0.06	0.062	0.067	0.069	0.072	0.119	0.12
**SOM**	0.056	0.046	0.018	0.041	0.045	0.048	0.027	0.007	0	0.008	0.067	0.07	0.074	0.076	0.079	0.126	0.127
**AFA**	0.062	0.051	0.02	0.048	0.049	0.047	0.026	0.002	0.008	0	0.047	0.05	0.054	0.056	0.06	0.113	0.114
**TSI**	0.15	0.138	0.095	0.143	0.139	0.124	0.102	0.06	0.067	0.047	0	0.003	0.004	0.006	0.012	0.11	0.111
**IBS**	0.151	0.14	0.097	0.144	0.141	0.126	0.104	0.062	0.07	0.05	0.003	0	0.003	0.005	0.011	0.111	0.113
**CEU**	0.154	0.143	0.101	0.147	0.144	0.13	0.108	0.067	0.074	0.054	0.004	0.003	0	0.002	0.007	0.11	0.112
**GBR**	0.156	0.145	0.102	0.149	0.145	0.131	0.109	0.069	0.076	0.056	0.006	0.005	0.002	0	0.008	0.11	0.112
**FIN**	0.157	0.146	0.104	0.15	0.146	0.133	0.111	0.072	0.079	0.06	0.012	0.011	0.007	0.008	0	0.101	0.103
**CHI**	0.182	0.172	0.14	0.175	0.173	0.167	0.146	0.119	0.126	0.113	0.11	0.111	0.11	0.11	0.101	0	0.007
**JPT**	0.183	0.174	0.142	0.176	0.174	0.168	0.148	0.12	0.127	0.114	0.111	0.113	0.112	0.112	0.103	0.007	0
