# Supplementary material for: Evidence for a Common Origin of Blacksmiths and Cultivators in the Ethiopian Ari within the Last 4500 Years: Lessons for Clustering-Based Inference
Source: PLoS Genet. 2015 Aug 20;11(8):e1005397. doi: 10.1371/journal.pgen.1005397 (PMC4546361; doi:10.1371/journal.pgen.1005397)
Supplement: S4 Table — Pairwise F ST [48] values among all populations in the “MA” “full” simulations of Fig 2a, i.e. mimicking the Marginalisation model with Pop5 and Pop5b splitting 20 generations ago, with a subsequent bottleneck in Pop5b. (PDF) [file pgen.1005397.s004.pdf]

	**Pop1**	**Pop2**	**Pop3**	**Pop4**	**Pop5b**	**Pop5**	**Pop6**	**Pop7**	**Pop8**	**Pop9**	**Pop10**	**Pop11**	**Pop12**
**Pop1**	0	0.122	0.122	0.122	0.139	0.113	0.112	0.123	0.205	0.205	0.206	0.254	0.254
**Pop2**	0.122	0	0.013	0.045	0.066	0.041	0.041	0.044	0.16	0.159	0.16	0.205	0.206
**Pop3**	0.122	0.013	0	0.044	0.066	0.041	0.041	0.044	0.159	0.159	0.159	0.205	0.206
**Pop4**	0.122	0.045	0.044	0	0.063	0.039	0.039	0.042	0.16	0.159	0.16	0.206	0.206
**Pop5b**	0.139	0.066	0.066	0.063	0	**0.025**	0.036	0.05	0.135	0.135	0.133	0.186	0.186
**Pop5**	0.113	0.041	0.041	0.039	**0.025**	0	0.012	0.025	0.114	0.113	0.111	0.165	0.165
**Pop6**	0.112	0.041	0.041	0.039	0.036	0.012	0	0.026	0.113	0.113	0.111	0.165	0.164
**Pop7**	0.123	0.044	0.044	0.042	0.05	0.025	0.026	0	0.159	0.159	0.159	0.207	0.207
**Pop8**	0.205	0.16	0.159	0.16	0.135	0.114	0.113	0.159	0	0.005	0.02	0.097	0.097
**Pop9**	0.205	0.159	0.159	0.159	0.135	0.113	0.113	0.159	0.005	0	0.02	0.097	0.096
**Pop10**	0.206	0.16	0.159	0.16	0.133	0.111	0.111	0.159	0.02	0.02	0	0.098	0.097
**Pop11**	0.254	0.205	0.205	0.206	0.186	0.165	0.165	0.207	0.097	0.097	0.098	0	0.016
**Pop12**	0.254	0.206	0.206	0.206	0.186	0.165	0.164	0.207	0.097	0.096	0.097	0.016	0
