# Supplementary material for: Evidence for a Common Origin of Blacksmiths and Cultivators in the Ethiopian Ari within the Last 4500 Years: Lessons for Clustering-Based Inference
Source: PLoS Genet. 2015 Aug 20;11(8):e1005397. doi: 10.1371/journal.pgen.1005397 (PMC4546361; doi:10.1371/journal.pgen.1005397)
Supplement: S5 Table — Pairwise F ST [48] values among all populations in the “RN” “full” simulations of Fig 2a, i.e. mimicking the Remnants model with Pop5 and Pop5b splitting 1700 generations ago and migrants from Pop5b comprising 50% of Pop5 over the period 200 to 300 generations ago. (PDF) [file pgen.1005397.s005.pdf]

	**Pop1**	**Pop2**	**Pop3**	**Pop4**	**Pop5b**	**Pop5**	**Pop6**	**Pop7**	**Pop8**	**Pop9**	**Pop10**	**Pop11**	**Pop12**
**Pop1**	0	0.122	0.122	0.122	0.112	0.105	0.107	0.121	0.208	0.208	0.207	0.254	0.254
**Pop2**	0.122	0	0.012	0.045	0.041	0.036	0.038	0.044	0.162	0.162	0.161	0.207	0.207
**Pop3**	0.122	0.012	0	0.045	0.042	0.036	0.038	0.044	0.162	0.162	0.162	0.207	0.208
**Pop4**	0.122	0.045	0.045	0	0.04	0.035	0.036	0.043	0.161	0.161	0.16	0.206	0.207
**Pop5b**	0.112	0.041	0.042	0.04	0	**0.013**	0.02	0.039	0.114	0.114	0.111	0.165	0.165
**Pop5**	0.105	0.036	0.036	0.035	**0.013**	0	0.009	0.026	0.109	0.109	0.106	0.16	0.161
**Pop6**	0.107	0.038	0.038	0.036	0.02	0.009	0	0.025	0.111	0.111	0.108	0.162	0.163
**Pop7**	0.121	0.044	0.044	0.043	0.039	0.026	0.025	0	0.161	0.161	0.161	0.206	0.207
**Pop8**	0.208	0.162	0.162	0.161	0.114	0.109	0.111	0.161	0	0.005	0.02	0.097	0.097
**Pop9**	0.208	0.162	0.162	0.161	0.114	0.109	0.111	0.161	0.005	0	0.02	0.096	0.097
**Pop10**	0.207	0.161	0.162	0.16	0.111	0.106	0.108	0.161	0.02	0.02	0	0.096	0.098
**Pop11**	0.254	0.207	0.207	0.206	0.165	0.16	0.162	0.206	0.097	0.096	0.096	0	0.016
**Pop12**	0.254	0.207	0.208	0.207	0.165	0.161	0.163	0.207	0.097	0.097	0.098	0.016	0
