# Supplementary material for: Evidence for a Common Origin of Blacksmiths and Cultivators in the Ethiopian Ari within the Last 4500 Years: Lessons for Clustering-Based Inference
Source: PLoS Genet. 2015 Aug 20;11(8):e1005397. doi: 10.1371/journal.pgen.1005397 (PMC4546361; doi:10.1371/journal.pgen.1005397)
Supplement: S6 Table — Pairwise F ST [48] values among all populations in the “RN+BN” “full” simulations of Fig 2a, i.e. mimicking the Remnants model with Pop5 and Pop5b splitting 1700 generations ago, migrants from Pop5b comprising 50% of Pop5 over the period 200 to 300 generations ago, and a subsequent bottleneck in Pop5b. (PDF) [file pgen.1005397.s006.pdf]

	**Pop1**	**Pop2**	**Pop3**	**Pop4**	**Pop5b**	**Pop5**	**Pop6**	**Pop7**	**Pop8**	**Pop9**	**Pop10**	**Pop11**	**Pop12**
**Pop1**	0	0.123	0.124	0.124	0.122	0.108	0.11	0.123	0.207	0.207	0.206	0.253	0.253
**Pop2**	0.123	0	0.012	0.043	0.05	0.036	0.038	0.044	0.16	0.161	0.16	0.207	0.207
**Pop3**	0.124	0.012	0	0.044	0.051	0.036	0.038	0.045	0.16	0.161	0.16	0.207	0.208
**Pop4**	0.124	0.043	0.044	0	0.049	0.034	0.037	0.042	0.161	0.162	0.161	0.207	0.208
**Pop5b**	0.122	0.05	0.051	0.049	0	**0.022**	0.029	0.048	0.122	0.122	0.12	0.173	0.174
**Pop5**	0.108	0.036	0.036	0.034	**0.022**	0	0.009	0.026	0.108	0.108	0.105	0.16	0.161
**Pop6**	0.11	0.038	0.038	0.037	0.029	0.009	0	0.025	0.11	0.11	0.107	0.162	0.163
**Pop7**	0.123	0.044	0.045	0.042	0.048	0.026	0.025	0	0.159	0.159	0.159	0.206	0.207
**Pop8**	0.207	0.16	0.16	0.161	0.122	0.108	0.11	0.159	0	0.005	0.02	0.096	0.096
**Pop9**	0.207	0.161	0.161	0.162	0.122	0.108	0.11	0.159	0.005	0	0.02	0.096	0.097
**Pop10**	0.206	0.16	0.16	0.161	0.12	0.105	0.107	0.159	0.02	0.02	0	0.096	0.096
**Pop11**	0.253	0.207	0.207	0.207	0.173	0.16	0.162	0.206	0.096	0.096	0.096	0	0.016
**Pop12**	0.253	0.207	0.208	0.208	0.174	0.161	0.163	0.207	0.096	0.097	0.096	0.016	0
