# Supplementary material for: Evidence for a Common Origin of Blacksmiths and Cultivators in the Ethiopian Ari within the Last 4500 Years: Lessons for Clustering-Based Inference
Source: PLoS Genet. 2015 Aug 20;11(8):e1005397. doi: 10.1371/journal.pgen.1005397 (PMC4546361; doi:10.1371/journal.pgen.1005397)
Supplement: S7 Table — Pairwise F ST [48] values among all populations in the “RN+BN+80%” “full” simulations of Fig 2a, i.e. mimicking the Remnants model with Pop5 and Pop5b splitting 1700 generations ago, migrants from Pop5b comprising 80% of Pop5 over the period 200 to 300 generations ago, and a subsequent bottleneck in Pop5b. (PDF) [file pgen.1005397.s007.pdf]

	**Pop1**	**Pop2**	**Pop3**	**Pop4**	**Pop5b**	**Pop5**	**Pop6**	**Pop7**	**Pop8**	**Pop9**	**Pop10**	**Pop11**	**Pop12**
**Pop1**	0	0.125	0.125	0.124	0.123	0.107	0.11	0.125	0.205	0.206	0.206	0.253	0.252
**Pop2**	0.125	0	0.012	0.045	0.051	0.035	0.038	0.044	0.159	0.16	0.159	0.205	0.204
**Pop3**	0.125	0.012	0	0.045	0.051	0.036	0.038	0.044	0.159	0.16	0.159	0.206	0.204
**Pop4**	0.124	0.045	0.045	0	0.049	0.034	0.036	0.042	0.159	0.159	0.159	0.205	0.204
**Pop5b**	0.123	0.051	0.051	0.049	0	**0.019**	0.029	0.049	0.12	0.12	0.118	0.171	0.169
**Pop5**	0.107	0.035	0.036	0.034	**0.019**	0	0.011	0.028	0.107	0.107	0.105	0.158	0.157
**Pop6**	0.11	0.038	0.038	0.036	0.029	0.011	0	0.025	0.11	0.11	0.107	0.16	0.159
**Pop7**	0.125	0.044	0.044	0.042	0.049	0.028	0.025	0	0.159	0.159	0.159	0.205	0.204
**Pop8**	0.205	0.159	0.159	0.159	0.12	0.107	0.11	0.159	0	0.005	0.02	0.095	0.095
**Pop9**	0.206	0.16	0.16	0.159	0.12	0.107	0.11	0.159	0.005	0	0.02	0.095	0.095
**Pop10**	0.206	0.159	0.159	0.159	0.118	0.105	0.107	0.159	0.02	0.02	0	0.095	0.094
**Pop11**	0.253	0.205	0.206	0.205	0.171	0.158	0.16	0.205	0.095	0.095	0.095	0	0.015
**Pop12**	0.252	0.204	0.204	0.204	0.169	0.157	0.159	0.204	0.095	0.095	0.094	0.015	0
