# Supplementary material for: Evidence for a Common Origin of Blacksmiths and Cultivators in the Ethiopian Ari within the Last 4500 Years: Lessons for Clustering-Based Inference
Source: PLoS Genet. 2015 Aug 20;11(8):e1005397. doi: 10.1371/journal.pgen.1005397 (PMC4546361; doi:10.1371/journal.pgen.1005397)
Supplement: S8 Table — Pairwise F ST [48] values among all populations in the “simplified” simulations of Fig 2b, i.e. mimicking the Remnants model with Pop5 and Pop5b splitting 750–1700 generations ago, a subsequent bottleneck of varying strength in Pop5b, and migrants from Pop5b comprising 50–80% of Pop5 over the period 200 to 300 generations ago. The minimum and maximum F ST values between each population pair across all “simplified” simulations is given. (PDF) [file pgen.1005397.s008.pdf]

Group	Pop1	Pop2	Pop3	Pop4	Pop5b	Pop5	Pop6
**Pop1**	0	0.012-0.013	0.044-0.045	0.044-0.047	0.051-0.061	0.035-0.04	0.142-0.149
**Pop2**	0.012-0.013	0	0.044-0.045	0.044-0.047	0.05-0.061	0.035-0.04	0.142-0.148
**Pop3**	0.044-0.045	0.044-0.045	0	0.042-0.044	0.049-0.058	0.033-0.037	0.142-0.148
**Pop4**	0.044-0.047	0.044-0.047	0.042-0.044	0	0.031-0.034	0.011-0.014	0.069-0.072
**Pop5b**	0.051-0.061	0.05-0.061	0.049-0.058	0.031-0.034	0	**0.019-0.027**	0.11-0.123
**Pop5**	0.035-0.04	0.035-0.04	0.033-0.037	0.011-0.014	**0.019-0.027**	0	0.095-0.102
**Pop6**	0.142-0.149	0.142-0.148	0.142-0.148	0.069-0.072	0.11-0.123	0.095-0.102	0
