# Supplementary material for: Evidence for a Common Origin of Blacksmiths and Cultivators in the Ethiopian Ari within the Last 4500 Years: Lessons for Clustering-Based Inference
Source: PLoS Genet. 2015 Aug 20;11(8):e1005397. doi: 10.1371/journal.pgen.1005397 (PMC4546361; doi:10.1371/journal.pgen.1005397)
Supplement: S9 Table — Median and 95% empirical quantiles for the average sizes of haplotype segments (in cM) that match to a single donor individual, across recipient individuals from the ARIc and ARIb under CHROMOPAINTER analyses (A)-(C). Also shown are corresponding values for simulated “Ari” groups Pop5/Pop5b under the Marginalisations (MA) and Remnants (RN) models in the “full” simulations. (PDF) [file pgen.1005397.s009.pdf]

**Group**	**(a) all-donors**	**(b) non-Ari-donors**	**(c) non-Pagani-donors**
**ARIc**	0.215 (0.201 - 0.223)	0.179 (0.17 - 0.183)	0.144 (0.14 - 0.147)
**ARIb**	0.269 (0.264 - 0.286)	0.179 (0.176 - 0.181)	0.142 (0.14 - 0.143)
**Pop5 (MA)**	0.229 (0.226 - 0.235)	0.177 (0.176 - 0.179)	0.149 (0.148 - 0.151)
**Pop5b (MA)**	0.596 (0.54 - 0.653)	0.176 (0.175 - 0.178)	0.148 (0.148 - 0.149)
**Pop5 (RN)**	0.214 (0.212 - 0.219)	0.176 (0.175 - 0.177)	0.149 (0.148 - 0.15)
**Pop5b (RN)**	0.206 (0.204 - 0.21)	0.175 (0.174 - 0.177)	0.147 (0.146 - 0.148)
**Pop5 (RN+BN)**	0.215 (0.209 - 0.22)	0.176 (0.175 - 0.178)	0.149 (0.148 - 0.15)
**Pop5b (RN+BN)**	0.317 (0.301 - 0.331)	0.174 (0.173 - 0.176)	0.146 (0.145 - 0.148)
**Pop5 (RN+BN+80%)**	0.215 (0.212 - 0.222)	0.176 (0.175 - 0.178)	0.149 (0.148 - 0.15)
**Pop5b (RN+BN+80%)**	0.312 (0.291 - 0.341)	0.175 (0.174 - 0.176)	0.147 (0.146 - 0.147)
