# Supplementary material for: Evidence for a Common Origin of Blacksmiths and Cultivators in the Ethiopian Ari within the Last 4500 Years: Lessons for Clustering-Based Inference
Source: PLoS Genet. 2015 Aug 20;11(8):e1005397. doi: 10.1371/journal.pgen.1005397 (PMC4546361; doi:10.1371/journal.pgen.1005397)
Supplement: S10 Table — Inferred proportions of ancestry (i.e. β^s) for all-donors analysis (A), plus and minus two standard errors calculated using a weighted block jackknife approach. (PDF) [file pgen.1005397.s010.pdf]

Group	YRI	LWK	MKK	ANU	GUM	ARIb	ARIc	ORO	SOM	AFA
**YRI**	58.7 (58-59.5)	41.3 (40.5-42)	0 (0-0)	0 (0-0)	0 (0-0)	0 (0-0)	0 (0-0)	0 (0-0)	0 (0-0)	0 (0-0)
**LWK**	37.9 (37.1-38.8)	62.1 (61.2-62.9)	0 (0-0)	0 (0-0)	0 (0-0)	0 (0-0)	0 (0-0)	0 (0-0)	0 (0-0)	0 (0-0)
**MKK**	0 (0-0)	21.7 (20.9-22.5)	33.7 (31.4-36)	23 (21.6-24.3)	0 (0-0)	0 (0-0)	0 (0-0)	7.9 (-2.6-18.4)	3.7 (2.5-4.9)	10 (2.2-17.8)
**ANU**	11.7 (11.3-12.1)	16.7 (16.1-17.2)	19.3 (17.8-20.9)	35 (33.5-36.5)	14.6 (13.3-15.8)	0 (0-0)	0 (0-0)	0 (0-0)	2.7 (2.2-3.2)	0 (0-0)
**GUM**	0 (0-0)	1 (0.6-1.4)	0 (0-0)	42.5 (41.3-43.6)	45.1 (42.7-47.4)	0 (0-0)	4.9 (4.1-5.7)	6.5 (2.6-10.5)	0 (0-0)	0 (-2.4-2.4)
**ARIb**	0 (0-0)	0 (0-0)	0 (0-0)	0 (0-0)	0 (0-0)	69.1 (66.7-71.6)	30.9 (28.4-33.3)	0 (0-0)	0 (0-0)	0 (0-0)
**ARIc**	0 (0-0)	2 (1.8-2.3)	0 (0-0)	2.9 (2.2-3.5)	0 (0-0)	11.7 (10.9-12.6)	38 (36-40)	45.4 (43.6-47.1)	0 (0-0)	0 (0-0)
**ORO**	0.5 (0.3-0.8)	0.2 (-0.1-0.6)	3.4 (2.7-4.1)	2.1 (1.4-2.7)	1.4 (1.2-1.6)	1.2 (0.9-1.5)	14.4 (13.5-15.3)	0.2 (-0.1-0.5)	6.4 (5.4-7.4)	70 (68.1-72)
**SOM**	0 (0-0)	0 (-0.1-0.1)	0 (0-0)	0 (0-0)	0 (0-0)	0 (0-0)	0 (0-0)	0 (0-0)	42.8 (41.2-44.5)	57.2 (55.5-58.8)
**AFA**	0 (0-0)	0 (0-0)	0 (0-0)	0 (0-0)	0 (0-0)	0 (0-0)	0 (0-0)	87.8 (86-89.7)	0.6 (-0.4-1.6)	6.5 (5.5-7.6)
**TSI**	0 (0-0)	0 (0-0)	0 (0-0)	0 (0-0)	0 (0-0)	0 (0-0)	0 (0-0)	0 (0-0)	0 (0-0)	0 (0-0)
**IBS**	0.5 (0.4-0.6)	0 (-0.1-0.1)	0 (0-0)	0 (0-0)	0 (0-0)	0 (0-0)	0 (0-0)	0 (0-0)	0 (0-0)	1.8 (1.4-2.2)
**CEU**	0 (0-0)	0 (0-0)	0 (0-0)	0 (0-0)	0 (0-0)	0 (0-0)	0 (0-0)	0 (0-0)	0 (0-0)	0 (0-0)
**GBR**	0 (0-0)	0 (0-0)	0 (0-0)	0 (0-0)	0 (0-0)	0 (0-0)	0 (0-0)	0 (0-0)	0 (0-0)	0 (0-0)
**FIN**	0 (0-0)	0 (0-0)	0 (0-0)	0 (0-0)	0 (0-0)	0 (0-0)	0 (0-0)	0 (0-0)	0 (0-0)	0 (0-0)
**CHI**	0 (0-0)	0 (0-0)	0 (0-0)	0 (0-0)	0 (0-0)	0 (0-0)	0 (0-0)	0 (0-0)	0 (0-0)	0 (0-0)
**JPT**	0 (0-0)	0 (0-0)	0 (0-0)	0 (0-0)	0 (0-0)	0 (0-0)	0 (0-0)	0 (0-0)	0 (0-0)	0 (0-0)

Group	TSI	IBS	CEU	GBR	FIN	CHI	JPT
**YRI**	0 (0-0)	0 (0-0)	0 (0-0)	0 (0-0)	0 (0-0)	0 (0-0)	0 (0-0)
**LWK**	0 (0-0)	0 (0-0)	0 (0-0)	0 (0-0)	0 (0-0)	0 (0-0)	0 (0-0)
**MKK**	0 (0-0)	0 (0-0)	0 (0-0)	0 (0-0)	0 (0-0)	0 (0-0)	0 (0-0)
**ANU**	0 (0-0)	0 (0-0)	0 (0-0)	0 (0-0)	0 (0-0)	0 (0-0)	0 (0-0)
**GUM**	0 (0-0)	0 (0-0)	0 (0-0)	0 (0-0)	0 (0-0)	0 (0-0)	0 (0-0)
**ARIb**	0 (0-0)	0 (0-0)	0 (0-0)	0 (0-0)	0 (0-0)	0 (0-0)	0 (0-0)
**ARIc**	0 (0-0)	0 (0-0)	0 (0-0)	0 (0-0)	0 (0-0)	0 (0-0)	0 (0-0)
**ORO**	0 (0-0)	0 (0-0)	0 (0-0)	0 (0-0)	0 (0-0)	0 (-0.1-0.2)	0.1 (-0.1-0.2)
**SOM**	0 (0-0)	0 (0-0)	0 (0-0)	0 (0-0)	0 (0-0)	0 (0-0)	0 (0-0)
**AFA**	5.1 (4.6-5.5)	0 (0-0)	0 (0-0)	0 (0-0)	0 (0-0)	0 (0-0)	0 (0-0)
**TSI**	7.9 (7.4-8.5)	92.1 (91.5-92.6)	0 (0-0)	0 (0-0)	0 (0-0)	0 (0-0)	0 (0-0)
**IBS**	37.4 (35.1-39.6)	2.2 (1.9-2.4)	55.7 (52.6-58.8)	1.6 (-0.5-3.6)	0 (0-0)	0.8 (0.6-1)	0.1 (-0.1-0.2)
**CEU**	0 (0-0)	45.3 (40.6-50)	2.5 (2.1-2.8)	48.3 (42.9-53.7)	3.9 (3.1-4.6)	0 (0-0)	0 (0-0)
**GBR**	0 (0-0)	0 (0-0)	93.8 (93.1-94.6)	5.6 (5.1-6.1)	0.6 (0-1.2)	0 (0-0)	0 (0-0)
**FIN**	0 (0-0)	0 (0-0)	53.7 (52.8-54.5)	0 (0-0)	46.3 (45.5-47.2)	0 (0-0)	0 (0-0)
**CHI**	0 (0-0)	0 (0-0)	0 (0-0)	0 (0-0)	0 (0-0)	46.7 (45.8-47.6)	53.3 (52.4-54.2)
**JPT**	0 (0-0)	0 (0-0)	0 (0-0)	0 (0-0)	0 (0-0)	70.1 (69.4-70.8)	29.9 (29.2-30.6)
