# Supplementary material for: Evidence for a Common Origin of Blacksmiths and Cultivators in the Ethiopian Ari within the Last 4500 Years: Lessons for Clustering-Based Inference
Source: PLoS Genet. 2015 Aug 20;11(8):e1005397. doi: 10.1371/journal.pgen.1005397 (PMC4546361; doi:10.1371/journal.pgen.1005397)
Supplement: S11 Table — Inferred proportions of ancestry (i.e. β^s) for non-Ari-donors analysis (B), plus and minus two standard errors calculated using a weighted block jackknife approach. (PDF) [file pgen.1005397.s011.pdf]

Group	YRI	LWK	MKK	ANU	GUM	ORO	SOM	AFA	TSI	IBS
**YRI**	56.9 (56.1-57.7)	43.1 (42.3-43.9)	0 (0-0)	0 (0-0)	0 (0-0)	0 (0-0)	0 (0-0)	0 (0-0)	0 (0-0)	0 (0-0)
**LWK**	38.6 (37.9-39.4)	59.7 (58.7-60.7)	0 (0-0)	1.7 (1.1-2.2)	0 (0-0)	0 (0-0)	0 (0-0)	0 (0-0)	0 (0-0)	0 (0-0)
**MKK**	1 (0.5-1.4)	19.4 (18.5-20.3)	14.8 (12.9-16.6)	19.4 (15.3-23.5)	0 (0-0)	35.5 (1.6-69.5)	0 (0-0)	10 (-19.8-39.8)	0 (0-0)	0 (0-0)
**ANU**	10.1 (9.8-10.4)	13.3 (12.9-13.8)	15.7 (14.5-16.9)	0 (0-0)	57.5 (56.1-59)	0 (0-0)	3.3 (2.7-3.9)	0 (0-0)	0 (0-0)	0 (0-0)
**GUM**	0 (0-0)	0 (0-0)	0 (0-0)	99.2 (98.9-99.6)	0 (0-0)	0 (0-0)	0 (0-0)	0 (0-0)	0 (0-0)	0 (0-0)
**ARIb**	0 (0-0)	0 (0-0)	22.2 (20.9-23.4)	19 (17.8-20.2)	0 (0-0)	0 (0-0)	12.4 (11.2-13.7)	45.3 (43.5-47)	0 (0-0)	0 (0-0)
**ARIc**	0.9 (0.7-1.2)	0 (0-0)	21.6 (20.4-22.8)	17.3 (16.4-18.1)	0 (0-0)	0 (0-0)	11.9 (10.9-12.9)	46.1 (44.4-47.7)	0 (0-0)	0 (0-0)
**ORO**	0 (-0.4-0.4)	0 (0-0)	3.3 (2.3-4.2)	9.4 (7.2-11.7)	0.4 (-0.7-1.5)	0 (0-0)	12.2 (10.6-13.7)	74.3 (72.3-76.3)	0 (0-0)	0 (0-0)
**SOM**	0 (0-0)	0 (0-0)	0 (0-0)	0 (0-0)	0 (0-0)	76.1 (75-77.2)	23.9 (22.8-25)	0 (0-0)	0 (0-0)	0 (0-0)
**AFA**	0 (0-0)	0 (0-0)	0 (0-0)	0 (0-0)	0 (0-0)	89.9 (89-90.7)	0 (0-0)	5.2 (4.5-6)	4.5 (3.5-5.6)	0 (0-0)
**TSI**	0 (0-0)	0 (0-0)	0 (0-0)	0 (0-0)	0 (0-0)	0 (0-0)	0 (0-0)	0 (0-0)	7.6 (7-8.1)	92.4 (91.9-93)
**IBS**	0.5 (0.3-0.7)	0 (-0.1-0.1)	0 (0-0)	0 (0-0)	0 (0-0)	0 (0-0)	0 (0-0)	1.8 (1.4-2.1)	38.1 (35.7-40.4)	1.9 (1.7-2.1)
**CEU**	0 (0-0)	0 (0-0)	0 (0-0)	0 (0-0)	0 (0-0)	0 (0-0)	0 (0-0)	0 (0-0)	0 (0-0)	43.6 (38.8-48.4)
**GBR**	0 (0-0)	0 (0-0)	0 (0-0)	0 (0-0)	0 (0-0)	0 (0-0)	0 (0-0)	0 (0-0)	0 (0-0)	0 (0-0)
**FIN**	0 (0-0)	0 (0-0)	0 (0-0)	0 (0-0)	0 (0-0)	0 (0-0)	0 (0-0)	0 (0-0)	0 (0-0)	0 (0-0)
**CHI**	0 (0-0)	0 (0-0)	0 (0-0)	0 (0-0)	0 (0-0)	0 (0-0)	0 (0-0)	0 (0-0)	0 (0-0)	0 (0-0)
**JPT**	0 (0-0)	0 (0-0)	0 (0-0)	0 (0-0)	0 (0-0)	0 (0-0)	0 (0-0)	0 (0-0)	0 (0-0)	0 (0-0)

Group	CEU	GBR	FIN	CHI	JPT
**YRI**	0 (0-0)	0 (0-0)	0 (0-0)	0 (0-0)	0 (0-0)
**LWK**	0 (0-0)	0 (0-0)	0 (0-0)	0 (0-0)	0 (0-0)
**MKK**	0 (0-0)	0 (0-0)	0 (0-0)	0 (0-0)	0 (0-0)
**ANU**	0 (0-0)	0 (0-0)	0 (0-0)	0 (0-0)	0 (0-0)
**GUM**	0 (0-0)	0 (0-0)	0.5 (0.3-0.7)	0.3 (0.2-0.5)	0 (0-0)
**ARIb**	0 (0-0)	0 (0-0)	0 (0-0)	1.1 (0.9-1.3)	0 (0-0)
**ARIc**	0 (0-0)	0 (0-0)	0 (0-0)	1.9 (1.7-2.1)	0.3 (0.1-0.4)
**ORO**	0 (0-0)	0 (0-0)	0 (0-0)	0.3 (0.2-0.5)	0.1 (-0.1-0.2)
**SOM**	0 (0-0)	0 (0-0)	0 (0-0)	0 (0-0)	0 (0-0)
**AFA**	0.4 (-0.6-1.3)	0 (0-0)	0 (0-0)	0 (0-0)	0 (0-0)
**TSI**	0 (0-0)	0 (0-0)	0 (0-0)	0 (0-0)	0 (0-0)
**IBS**	54.7 (51.7-57.7)	1.6 (-0.3-3.6)	0 (0-0)	0.2 (-0.1-0.5)	1.2 (1-1.5)
**CEU**	2.9 (2.5-3.3)	49.8 (44.2-55.4)	3.7 (2.9-4.5)	0 (0-0)	0 (0-0)
**GBR**	93.2 (92.4-94)	5.9 (5.4-6.4)	0.9 (0.3-1.5)	0 (0-0)	0 (0-0)
**FIN**	54.6 (53.8-55.5)	0 (0-0)	45.4 (44.5-46.2)	0 (0-0)	0 (0-0)
**CHI**	0 (0-0)	0 (0-0)	0 (0-0)	46.5 (45.6-47.4)	53.5 (52.6-54.4)
**JPT**	0 (0-0)	0 (0-0)	0 (0-0)	70.7 (70-71.4)	29.3 (28.6-30)
