# Supplementary material for: Evidence for a Common Origin of Blacksmiths and Cultivators in the Ethiopian Ari within the Last 4500 Years: Lessons for Clustering-Based Inference
Source: PLoS Genet. 2015 Aug 20;11(8):e1005397. doi: 10.1371/journal.pgen.1005397 (PMC4546361; doi:10.1371/journal.pgen.1005397)
Supplement: S12 Table — Inferred proportions of ancestry (i.e. β^s) for non-Pagani-donors analysis (C), plus and minus two standard errors calculated using a weighted block jackknife approach. (PDF) [file pgen.1005397.s012.pdf]

Group	YRI	LWK	MKK	TSI	IBS	CEU	GBR	FIN	CHI	JPT
**YRI**	54.5 (53.8-55.3)	45.5 (44.7-46.2)	0 (0-0)	0 (0-0)	0 (0-0)	0 (0-0)	0 (0-0)	0 (0-0)	0 (0-0)	0 (0-0)
**LWK**	41.9 (41.4-42.5)	57.7 (56.5-58.9)	0.4 (-0.5-1.3)	0 (0-0)	0 (0-0)	0 (0-0)	0 (0-0)	0 (0-0)	0 (0-0)	0 (0-0)
**MKK**	10.9 (10.3-11.5)	45.7 (44.6-46.7)	29.2 (27.5-31)	12.2 (11.5-12.9)	0 (0-0)	0 (0-0)	0 (0-0)	0 (0-0)	2 (1.7-2.3)	0 (0-0)
**ANU**	22.8 (22.1-23.5)	32.5 (31.6-33.5)	43.1 (41.9-44.4)	0 (0-0)	0 (0-0)	0 (0-0)	0 (0-0)	0 (0-0)	1.5 (1.3-1.8)	0 (0-0)
**GUM**	18.5 (17.7-19.2)	22.3 (20.9-23.7)	49.6 (47.3-52)	0 (0-0)	0 (0-0)	0 (0-0)	0.8 (0.3-1.4)	2.1 (1.9-2.2)	5.5 (5.2-5.7)	1.2 (1-1.4)
**ARIb**	10.7 (9.8-11.6)	10.4 (8.5-12.4)	59 (55.8-62.3)	0 (0-0)	0 (0-0)	8.9 (7.9-9.9)	2.1 (1.5-2.8)	0.9 (0.6-1.1)	6.7 (6.3-7)	1.3 (1.1-1.5)
**ARIc**	10.7 (10.2-11.3)	9.2 (7.4-11.1)	58 (54.9-61.1)	0 (0-0)	2.2 (-0.7-5.2)	8.6 (6.5-10.7)	2 (1.5-2.5)	0.9 (0.7-1.1)	7 (6.8-7.3)	1.2 (1.1-1.4)
**ORO**	6.6 (6.2-7)	4.3 (2.4-6.2)	55.3 (52.2-58.3)	22 (19.4-24.6)	6 (4-8)	0 (0-0)	0 (0-0)	0.4 (0.1-0.6)	5.3 (5-5.5)	0.2 (0-0.4)
**SOM**	5.3 (4.7-5.9)	2 (0.1-3.9)	64.2 (60.9-67.6)	13.5 (11.3-15.7)	10.1 (8.3-11.9)	0 (0-0)	0 (0-0)	0.4 (0.3-0.6)	4.5 (4.1-4.8)	0 (0-0)
**AFA**	4.4 (3.9-4.8)	2.7 (1.3-4.1)	50.5 (48.1-53)	37.3 (36.6-38)	0 (0-0)	0 (0-0)	0 (0-0)	0 (0-0)	5.1 (4.9-5.3)	0 (0-0)
**TSI**	0 (0-0)	0 (0-0)	0 (0-0)	6.7 (6.2-7.2)	93.3 (92.8-93.8)	0 (0-0)	0 (0-0)	0 (0-0)	0 (0-0)	0 (0-0)
**IBS**	0.7 (0.5-0.9)	0.2 (0.1-0.4)	0.2 (0.1-0.4)	39.2 (37-41.5)	1.7 (1.6-1.9)	54.9 (51.8-58.1)	1.4 (-0.6-3.4)	0 (0-0)	0.3 (0-0.6)	1.2 (1-1.5)
**CEU**	0 (0-0)	0 (0-0)	0 (0-0)	0 (0-0)	49 (44.2-53.8)	2.9 (2.5-3.3)	44.4 (38.8-50)	3.7 (2.9-4.5)	0 (0-0)	0 (0-0)
**GBR**	0 (0-0)	0 (0-0)	0 (0-0)	0 (0-0)	0 (0-0)	93.3 (92.6-94)	5.8 (5.4-6.3)	0.9 (0.3-1.5)	0 (0-0)	0 (0-0)
**FIN**	0 (0-0)	0 (0-0)	0 (0-0)	0 (0-0)	0 (0-0)	56.3 (55.5-57.2)	0 (0-0)	43.7 (42.8-44.5)	0 (0-0)	0 (0-0)
**CHI**	0 (0-0)	0 (0-0)	0 (0-0)	0 (0-0)	0 (0-0)	0 (0-0)	0 (0-0)	0 (0-0)	45 (44-45.9)	55 (54.1-56)
**JPT**	0 (0-0)	0 (0-0)	0 (0-0)	0 (0-0)	0 (0-0)	0 (0-0)	0 (0-0)	0 (0-0)	71.6 (70.9-72.3)	28.4 (27.7-29.1)
