# Supplementary material for: Evidence for a Common Origin of Blacksmiths and Cultivators in the Ethiopian Ari within the Last 4500 Years: Lessons for Clustering-Based Inference
Source: PLoS Genet. 2015 Aug 20;11(8):e1005397. doi: 10.1371/journal.pgen.1005397 (PMC4546361; doi:10.1371/journal.pgen.1005397)
Supplement: S13 Table — Median PI_HAT values across all pairwise combinations of individuals within each group as calculated using PLINK v1.07 [28], with 95% empirical quantiles given in parenthesis. Results are shown when analysing all SNPs (“unpruned”) or a dataset where SNPs were pruned to remove those in high linkage disequilibrium (r 2 > 0.2) in a sliding 250 SNP window using the command –indep-pairwise 250 10 0.2. (PDF) [file pgen.1005397.s013.pdf]

Group	Median unpruned PI_HAT	Median pruned PI_HAT
CEU	0.082 (0.075-0.09)	0.149 (0.143-0.156)
GBR	0.127 (0.12-0.137)	0.209 (0.204-0.216)
CHI	0.12 (0.114-0.127)	0.203 (0.197-0.209)
FIN	0.09 (0.084-0.098)	0.163 (0.157-0.169)
IBS	0.14 (0.132-0.153)	0.225 (0.219-0.236)
JPT	0.151 (0.145-0.16)	0.237 (0.232-0.243)
LWK	0.037 (0.031-0.045)	0.064 (0.058-0.072)
TSI	0.08 (0.074-0.086)	0.147 (0.142-0.153)
YRI	0.054 (0.049-0.06)	0.079 (0.074-0.086)
MKK	0.055 (0.048-0.076)	0.098 (0.092-0.119)
AFA	0.034 (0-0.044)	0.071 (0.061-0.081)
ORO	0.1 (0.095-0.106)	0.166 (0.161-0.172)
SOM	0.056 (0.048-0.065)	0.103 (0.094-0.112)
GUM	0.149 (0.138-0.162)	0.196 (0.189-0.208)
ANU	0.074 (0.062-0.086)	0.107 (0.092-0.12)
ARIb	**0.179 (0.168-0.218)**	**0.241 (0.233-0.278)**
ARIc	**0.084 (0.075-0.092)**	**0.14 (0.133-0.149)**
