# Supplementary material for: Evidence for a Common Origin of Blacksmiths and Cultivators in the Ethiopian Ari within the Last 4500 Years: Lessons for Clustering-Based Inference
Source: PLoS Genet. 2015 Aug 20;11(8):e1005397. doi: 10.1371/journal.pgen.1005397 (PMC4546361; doi:10.1371/journal.pgen.1005397)
Supplement: S14 Table — CHROMOPAINTER’s inferred average haplotype segment sizes (in cM) copied intact from a single donor, and corresponding number of SNPs per segment and switch rate (with emission rate fixed at 0.00771 for all groups), when allowing 10 randomly-sampled individuals from each group to copy from the other 9 individuals with the same label, using 50 steps of Expectation-Maximisation (E-M). Median and empirical quantile values across the 10 individuals are given for each group. The segment sizes capture (roughly) the relative amount of haplotype diversity in each group. Note the high values for the ARIb relative to all other groups, including the ARIc. (PDF) [file pgen.1005397.s014.pdf]

Group	segment size (95% CI)	#SNPs (95% CI)	switch rate (95% CI)
YRI	0.058 (0.057 - 0.058)	5.4 (5.3 - 5.5)	2612.4 (2583 - 2690.2)
LWK	0.054 (0.053 - 0.055)	5.1 (5 - 5.2)	2898.1 (2794.3 - 2958.6)
MKK	0.054 (0.052 - 0.06)	5.1 (4.9 - 5.6)	2884.8 (2504.1 - 3034.9)
ANU	0.054 (0.053 - 0.058)	5.1 (5 - 5.5)	2870 (2598.7 - 2992)
GUM	0.065 (0.059 - 0.068)	6.1 (5.6 - 6.4)	2224.9 (2106.2 - 2525.6)
**ARIb**	**0.101 (0.095 - 0.114)**	**9.5 (9 - 10.7)**	**1258.2 (1090.7 - 1353.4)**
**ARIc**	**0.058 (0.056 - 0.06)**	**5.4 (5.2 - 5.7)**	**2624 (2478.3 - 2758.8)**
ORO	0.056 (0.055 - 0.058)	5.3 (5.2 - 5.5)	2710.3 (2592.1 - 2816.6)
SOM	0.07 (0.064 - 0.073)	6.6 (6 - 6.9)	2007.2 (1899.5 - 2271.8)
AFA	0.061 (0.057 - 0.063)	5.7 (5.4 - 5.9)	2428.1 (2341.8 - 2649.6)
TSI	0.091 (0.09 - 0.094)	8.6 (8.5 - 8.9)	1440.1 (1386.5 - 1465.6)
IBS	0.096 (0.089 - 0.099)	9 (8.3 - 9.3)	1361.8 (1298.7 - 1495.6)
CEU	0.097 (0.096 - 0.098)	9.1 (9.1 - 9.2)	1336.3 (1317.7 - 1348.7)
GBR	0.102 (0.099 - 0.107)	9.6 (9.4 - 10.1)	1255.5 (1185.6 - 1294)
FIN	0.107 (0.105 - 0.11)	10.1 (9.9 - 10.3)	1177.8 (1142.3 - 1206.6)
CHI	0.093 (0.092 - 0.095)	8.8 (8.7 - 8.9)	1401.3 (1378.2 - 1423.1)
JPT	0.1 (0.099 - 0.102)	9.4 (9.3 - 9.6)	1283.3 (1261.2 - 1303.4)
