# Supplementary material for: Evidence for a Common Origin of Blacksmiths and Cultivators in the Ethiopian Ari within the Last 4500 Years: Lessons for Clustering-Based Inference
Source: PLoS Genet. 2015 Aug 20;11(8):e1005397. doi: 10.1371/journal.pgen.1005397 (PMC4546361; doi:10.1371/journal.pgen.1005397)
Supplement: S15 Table — TVD XY scores (see Methods) measuring the difference in inferred ancestry between each pairing of Pagani groups under analyses (A)-(C); these values are described with a heatmap in Fig 3. Note for analysis (C) that no pairing has a lower value than that of the ARIb and ARIc (in red), suggesting the two Ari groups share more common ancestry, as related to the non-Pagani-donors, than any other pairing of Pagani groups. (PDF) [file pgen.1005397.s015.pdf]

(A) all-donors							
**Group**	**ANU**	**GUM**	**ARIb**	**ARIc**	**ORO**	**SOM**	**AFA**
**ANU**	0	0.249	0.464	0.379	0.386	0.42	0.422
**GUM**	0.249	0	0.414	0.318	0.329	0.385	0.378
**ARIb**	0.464	0.414	0	**0.307**	0.385	0.438	0.425
**ARIc**	0.379	0.318	**0.307**	0	0.201	0.283	0.269
**ORO**	0.386	0.329	0.385	0.201	0	0.17	0.076
**SOM**	0.42	0.385	0.438	0.283	0.17	0	0.179
**AFA**	0.422	0.378	0.425	0.269	0.076	0.179	0

(B) non-Ari-donors							
**Group**	**ANU**	**GUM**	**ARIb**	**ARIc**	**ORO**	**SOM**	**AFA**
**ANU**	0	0.137	0.26	0.265	0.34	0.393	0.387
**GUM**	0.137	0	0.258	0.262	0.318	0.371	0.365
**ARIb**	0.26	0.258	0	**0.017**	0.12	0.199	0.166
**ARIc**	0.265	0.262	**0.017**	0	0.111	0.192	0.155
**ORO**	0.34	0.318	0.12	0.111	0	0.161	0.06
**SOM**	0.393	0.371	0.199	0.192	0.161	0	0.175
**AFA**	0.387	0.365	0.166	0.155	0.06	0.175	0

(C) non-Pagani-donors							
**Group**	**ANU**	**GUM**	**ARIb**	**ARIc**	**ORO**	**SOM**	**AFA**
**ANU**	0	0.09	0.188	0.203	0.285	0.259	0.342
**GUM**	0.09	0	0.101	0.113	0.195	0.173	0.253
**ARIb**	0.188	0.101	0	**0.015**	0.101	0.083	0.155
**ARIc**	0.203	0.113	**0.015**	0	0.089	0.075	0.144
**ORO**	0.285	0.195	0.101	0.089	0	0.025	0.058
**SOM**	0.259	0.173	0.083	0.075	0.025	0	0.083
**AFA**	0.342	0.253	0.155	0.144	0.058	0.083	0
