# Supplementary material for: Evidence for a Common Origin of Blacksmiths and Cultivators in the Ethiopian Ari within the Last 4500 Years: Lessons for Clustering-Based Inference
Source: PLoS Genet. 2015 Aug 20;11(8):e1005397. doi: 10.1371/journal.pgen.1005397 (PMC4546361; doi:10.1371/journal.pgen.1005397)
Supplement: S16 Table — F XY scores (see Methods) measuring the difference in inferred ancestry between each pairing of Pagani groups under analyses (A)-(C). Note for analysis (C) that no pairing has a lower value than that of the ARIb and ARIc (in red), suggesting the two Ari groups share more common ancestry, as related to the non-Pagani-donors, than any other pairing of Pagani groups. (PDF) [file pgen.1005397.s016.pdf]

| (A) all-donors | | | | | | | |
|---|---|---|---|---|---|---|---|
| **Group** | **ANU** | **GUM** | **ARIb** | **ARIc** | **ORO** | **SOM** | **AFA** |
| **ANU** | 0 | 12.529 | 16.774 | 19.678 | 21.293 | 22.87 | 27.558 |
| **GUM** | 12.529 | 0 | 13.475 | 14.294 | 15.539 | 17.99 | 20.571 |
| **ARIb** | 16.774 | 13.475 | 0 | **10.198** | 13.281 | 14.996 | 16.255 |
| **ARIc** | 19.678 | 14.294 | **10.198** | 0 | 9.774 | 13.635 | 15.157 |
| **ORO** | 21.293 | 15.539 | 13.281 | 9.774 | 0 | 8.649 | 4.578 |
| **SOM** | 22.87 | 17.99 | 14.996 | 13.635 | 8.649 | 0 | 10.625 |
| **AFA** | 27.558 | 20.571 | 16.255 | 15.157 | 4.578 | 10.625 | 0 |
| (B) non-Ari-donors | | | | | | | |
| **Group** | **ANU** | **GUM** | **ARIb** | **ARIc** | **ORO** | **SOM** | **AFA** |
| **ANU** | 0 | 7.372 | 14.048 | 16.234 | 20.68 | 22.415 | 26.473 |
| **GUM** | 7.372 | 0 | 12.135 | 13.75 | 16.594 | 18.312 | 21.043 |
| **ARIb** | 14.048 | 12.135 | 0 | **0.916** | 6.279 | 9.847 | 9.566 |
| **ARIc** | 16.234 | 13.75 | **0.916** | 0 | 6.555 | 10.639 | 10.23 |
| **ORO** | 20.68 | 16.594 | 6.279 | 6.555 | 0 | 8.865 | 3.941 |
| **SOM** | 22.415 | 18.312 | 9.847 | 10.639 | 8.865 | 0 | 10.744 |
| **AFA** | 26.473 | 21.043 | 9.566 | 10.23 | 3.941 | 10.744 | 0 |
| (C) non-Pagani-donors | | | | | | | |
| **Group** | **ANU** | **GUM** | **ARIb** | **ARIc** | **ORO** | **SOM** | **AFA** |
| **ANU** | 0 | 6.209 | 11.609 | 14.536 | 20.286 | 16.784 | 27.377 |
| **GUM** | 6.209 | 0 | 6.375 | 8.326 | 14.253 | 11.487 | 20.785 |
| **ARIb** | 11.609 | 6.375 | 0 | **0.951** | 6.499 | 4.917 | 11.137 |
| **ARIc** | 14.536 | 8.326 | **0.951** | 0 | 6.747 | 5.091 | 12.312 |
| **ORO** | 20.286 | 14.253 | 6.499 | 6.747 | 0 | 1.726 | 4.896 |
| **SOM** | 16.784 | 11.487 | 4.917 | 5.091 | 1.726 | 0 | 6.297 |
| **AFA** | 27.377 | 20.785 | 11.137 | 12.312 | 4.896 | 6.297 | 0 |
