# Supplementary material for: Evidence for a Common Origin of Blacksmiths and Cultivators in the Ethiopian Ari within the Last 4500 Years: Lessons for Clustering-Based Inference
Source: PLoS Genet. 2015 Aug 20;11(8):e1005397. doi: 10.1371/journal.pgen.1005397 (PMC4546361; doi:10.1371/journal.pgen.1005397)
Supplement: S17 Table — The proportion of pairwise F XY scores under analysis (C) between individuals from the same group (rows) that are greater than or equal to the mean F XY across all pairwise combinations of individuals with one from the group given in the row and the other from the group given in the column. (PDF) [file pgen.1005397.s017.pdf]

	**ANU**	**GUM**	**ARIb**	**ARIc**	**ORO**	**SOM**	**AFA**
**ANU**	NA	0.035	0	0	0	0	0
**GUM**	0	NA	0	0	0	0	0
**ARIb**	0	0	NA	**0**	0	0	0
**ARIc**	0	0	**0.198**	NA	0	0	0
**ORO**	0	0	0	0	NA	0.182	0
**SOM**	0	0	0	0	0.075	NA	0
**AFA**	0	0	0	0	0.003	0	NA
