# Supplementary material for: Evidence for a Common Origin of Blacksmiths and Cultivators in the Ethiopian Ari within the Last 4500 Years: Lessons for Clustering-Based Inference
Source: PLoS Genet. 2015 Aug 20;11(8):e1005397. doi: 10.1371/journal.pgen.1005397 (PMC4546361; doi:10.1371/journal.pgen.1005397)
Supplement: S18 Table — The proportion of pairwise F XY scores under analysis (B) between individuals from the same group (i.e. either Pop5b or Pop5, as given in the columns above, which are meant to represent the “ARIb” and “ARIc”, respectively) that are greater than or equal to the mean F XY across all pairwise combinations of individuals with one from Pop5b and the other from Pop5. Results from each of 24 “simplified” simulations are given, which vary in the number of generations ago Pop5 and Pop5b split (“split”), the number of generations Pop5b is bottlenecked (“BN”), and the proportion of migrants contributed from Pop5b to Pop5 (“% admixture”). (PDF) [file pgen.1005397.s018.pdf]

**Simulation**	**50% admixture**		**75% admixture**		**90% admixture**	
	**Pop5b**	**Pop5**	**Pop5b**	**Pop5**	**Pop5b**	**Pop5**
**split=1700, BN=20**	0	0	0	0	0	0
**split=1300, BN=30**	0	0	0	0	0	0
**split=1200, BN=30**	0	0	0	0	0	0.059
**split=1100, BN=35**	0	0	0.022	0.012	0	0.079
**split=1000, BN=35**	0	0.055	0.022	0.119	0.333	0.107
**split=900, BN=35**	0.333	0.316	0.422	0.387	0.444	0.427
**split=800, BN=40**	0.356	0.332	0.267	0.221	0.267	0.494
**split=750, BN=40**	0.222	0.138	0.156	0.103	0.111	0.213
