# Supplementary material for: Evidence for a Common Origin of Blacksmiths and Cultivators in the Ethiopian Ari within the Last 4500 Years: Lessons for Clustering-Based Inference
Source: PLoS Genet. 2015 Aug 20;11(8):e1005397. doi: 10.1371/journal.pgen.1005397 (PMC4546361; doi:10.1371/journal.pgen.1005397)
Supplement: S19 Table — Proportion of MCMC samples for which all ARIb individuals are clustered separately from all ARIc individuals, when inferring two clusters under our MCMC clustering algorithm for each of analyses (A)-(C). The median and range across 10 independent runs of the MCMC chain are shown. Analogous results are given for the “full” simulations: “MA”, “RN”, “RN+BN”, and “RN+BN+80%”. Results are shown for two different choices of prior value δ (see Methods). (PDF) [file pgen.1005397.s019.pdf]

$\delta = 100$			
**Data**	**(A) all-donors**	**(B) non-Ari-donors**	**(C) non-Pagani-donors**
**PAGANI DATA**	1 (1 - 1)	0 (0 - 0)	0 (0 - 0)
**SIMS: MA**	1 (1 - 1)	0 (0 - 0)	0 (0 - 0)
**SIMS: RN**	1 (1 - 1)	0.96 (0.94 - 0.98)	1 (0.99 - 1)
**SIMS: RN+BN**	1 (1 - 1)	0.93 (0.91 - 0.98)	0.99 (0.98 - 1)
**SIMS: RN+BN+80%**	1 (1 - 1)	0 (0 - 0)	0.72 (0.66 - 0.81)

$\delta = 50$			
**Data**	**(A) all-donors**	**(B) non-Ari-donors**	**(C) non-Pagani-donors**
**PAGANI DATA**	1 (1 - 1)	0 (0 - 0)	0 (0 - 0)
**SIMS: MA**	1 (1 - 1)	0 (0 - 0)	0 (0 - 0)
**SIMS: RN**	1 (1 - 1)	0.96 (0.93 - 0.99)	0.99 (0.98 - 1)
**SIMS: RN+BN**	1 (1 - 1)	0.94 (0.91 - 0.98)	0.99 (0.98 - 1)
**SIMS: RN+BN+80%**	1 (1 - 1)	0.36 (0.3 - 0.38)	0.74 (0.69 - 0.78)
