# Supplementary material for: Evidence for a Common Origin of Blacksmiths and Cultivators in the Ethiopian Ari within the Last 4500 Years: Lessons for Clustering-Based Inference
Source: PLoS Genet. 2015 Aug 20;11(8):e1005397. doi: 10.1371/journal.pgen.1005397 (PMC4546361; doi:10.1371/journal.pgen.1005397)
Supplement: S20 Table — GLOBETROTTER’s inferred dates (both generations and years from present, with bootstrap 95% CIs given in parenthesis), admixing sources (single best matching sampled surrogate is given first, followed by mixing proportions > 10% giving more precise inference on the haplotype make-up of the source; see Methods), and proportion (%) of admixture contributed from each source for inferred admixture events in the Ari groups under analyses (A), (A-sim), (B) and (C). Here (A-sim) refers to a simplified GLOBETROTTER (A) analysis where the surrogates specified are TSI, ANU, ORO and {ARIb, ARIc}. “Props” gives more stably estimated source compositions than the mixing coefficients when multi-way admixture is inferred (as described in [33]) and are reported when the value is > 0.05. Assuming a generation time of 28 years, generations g were converted to years y using the formula: y = 1950 − (g + 1) × 28. (PDF) [file pgen.1005397.s020.pdf]

Analysis	Group	First Event									
		Date (gen)	Date (years)	%	Source 1		Source 1 Props	%	Source 2		Source 2 Props
A	ARIb	52 (27-74)	466CE (150BCE-1166CE)	32	SOM	GBR(16%),ANU(28%),SOM(54%)	ARIc(0.13)	68	ARIc	ARIc(100%)	CEU(0.06), SOM(0.12)
	ARIc	71 (57-83)	66BCE (402BCE-326CE)	24	ARIb	ANU(15%),GUM(19%),ARIb(53%)	AFA(0.1)	76	ORO	ORO(80%)	ARIb(0.19)
		**Second Event**									
		Date (gen)	Date (years)	%	Source 1		Source 1 Props	%	Source 2		Source 2 Props
	ARIc	71 (57-83)	66BCE (402BCE-326CE)	19	ANU	MKK(11%),GUM(12%),ANU(62%)	AFA(0.08), ARIc(0.08)	81	ORO	ARIb(18%),ORO(72%)	YRI(0.06), LWK(0.07), ANU(0.12)
Analysis	Group	**First Event**									
		Date (gen)	Date (years)	%	Source 1		Source 1 Props	%	Source 2		Source 2 Props
A-sim	ARIb	62 (44-83)	186CE (402BCE-690CE)	26	ANU	TSI(26%),ANU(74%)	AFA(0.06), ARIc(0.20)	74	ARIc	ARIc(97%)	YRI(0.05), LWK(0.05), ANU(0.11)
	ARIc	72 (59-88)	94BCE (542BCE-270CE)	21	ARIb	ANU(38%),ARIb(58%)	AFA(0.09)	79	ORO	ANU(13%),ORO(83%)	ARIb(0.2)
		**Second Event**									
		Date (gen)	Date (years)	%	Source 1		Source 1 Props	%	Source 2		Source 2 Props
	ARIb	62 (44-83)	186CE (402BCE-690CE)	42	ARIc	ORO(15%),TSI(22%),ARIC(62%)	LWK(0.05), ANU(0.07)	58	ARIc	ANU(31%),ARIc(70%)	TSI(0.05), CEU(0.07)
	ARIc	72 (59-88)	94BCE (542BCE-270CE)	25	ANU	ORO(27%),ANU(71%)	AFA(0.07), ARIb(0.07)	75	ORO	ARIb(19%),ORO(80%)	YRI(0.06), LWK(0.07), ANU(0.13)
	Group	**First Event**									
		Date (gen)	Date (years)	%	Source 1			%	Source 2		
B	ARIb	72 (53-85)	94BCE (458BCE-438CE)	41	ANU	SOM(16%),ANU(84%)		59	AFA	AFA(90%)	
	ARIc	73 (54-82)	122BCE (374BCE-410CE)	41	ANU	SOM(16%),ANU(84%)		59	AFA	AFA(100%	
	Group	**First Event**									
		Date (gen)	Date (years)	%	Source 1			%	Source 2		
C	ARIb	121 (91-149)	1466BCE (2250BCE-626BCE)	30	CEU	CEU(32%),MKK(46%)		70	MKK	YRI(17%),LWK(20%),MKK(55%)	
	ARIc	100 (85-115)	878BCE (1298BCE-458BCE)	39	CEU	IBS(14%),CEU(16%),MKK(52%)		61	MKK	YRI(19%),LWK(20%),MKK(52%)	
