# Supplementary material for: Evidence for a Common Origin of Blacksmiths and Cultivators in the Ethiopian Ari within the Last 4500 Years: Lessons for Clustering-Based Inference
Source: PLoS Genet. 2015 Aug 20;11(8):e1005397. doi: 10.1371/journal.pgen.1005397 (PMC4546361; doi:10.1371/journal.pgen.1005397)
Supplement: S21 Table — GLOBETROTTER’s inferred dates (both generations and years from present, with bootstrap 95% CIs given in parenthesis), admixing sources (single best matching sampled surrogate is given first, followed by mixing proportions > 10% giving more precise inference on the haplotype make-up of the source; see Methods), and proportion (%) of admixture contributed from each source for inferred admixture events in the Ari groups under analyses (A*),(B*) and (C*). (A*), (B*) and (C*) give the inferred dates and admixing sources under each analysis (A), (B) and (C) when only 10 ARIc individuals are used. “Props” gives more stably estimated source compositions than the mixing coefficients when multi-way admixture is inferred (as described in [33]) and are reported for sources where the value is > 0.05. Assuming a generation time of 28 years, generations g were converted to years y using the formula: y = 1950 − (g + 1) × 28. (PDF) [file pgen.1005397.s021.pdf]

Analysis	Group	**First Event**									
		Date (gen)	Date (years)	%	Source 1		Source 1 Props	%	Source 2		Source 2 Props
A*	ARIb	82 (62-106)	374BCE (1046BCE-186CE)	22	SOM	GBR(37%),SOM(63%)	LWK(0.06),ARIc(0.09)	78	ARIc	ARIc(87%)	FIN(0.06),SOM(0.10),CEU(0.11)
	ARIc	78 (61-94)	262BCE (710BCE-214CE)	27	ARIb	ANU(17%),GUM(18%),ARIb(53%)	AFA(0.09)	73	ORO	ORO(81%)	ARIb(0.19)
		**Second Event**									
		Date (gen)	Date (years)	%	Source 1		Source 1 Props	%	Source 2		Source 2 Props
	ARIb	Same	Same	49	SOM	ANU(24%),SOM(71%)	ARIc(0.08)	51	AFA	GUM(18%),MKK(24%),AFA(59%)	LWK(0.06),SOM(0.1)
	ARIc	Same	Same	48	SOM	ANU(15%),SOM(76%)	ARIb(0.08),AFA(0.09)	52	AFA	MKK(43%),AFA(57%)	YRI(0.08),LWK(0.1),ANU(0.12)
B*	Group	**First Event**									
		Date (gen)	Date (years)	%	Source 1			%	Source 2		
	ARIb	72 (53-85)	94BCE (458BCE-438CE)	41	ANU	SOM(16%),ANU(84%)		59	AFA	AFA(90%)	
	ARIc	109 (88-130)	1130BCE (1718BCE-542BCE)	40	ANU	ANU(94%)		60	AFA	AFA(100%)	
C*	Group	**First Event**									
		Date (gen)	Date (years)	%	Source 1			%	Source 2		
	ARIb	121 (91-149)	1466BCE (2250BCE-626BCE)	30	CEU	CEU(32%),MKK(46%)		70	MKK	YRI(17%),LWK(20%),MKK(55%)	
	ARIc	138 (118-153)	1942BCE (2362BCE-1382BCE)	41	CEU	GBR(46%),MKK(47%)		59	LWK	YRI(24%),LWK(30%),MKK(36%)	
