# Supplementary material for: Evidence for a Common Origin of Blacksmiths and Cultivators in the Ethiopian Ari within the Last 4500 Years: Lessons for Clustering-Based Inference
Source: PLoS Genet. 2015 Aug 20;11(8):e1005397. doi: 10.1371/journal.pgen.1005397 (PMC4546361; doi:10.1371/journal.pgen.1005397)
Supplement: S22 Table — GLOBETROTTER’s inferrence when assuming two distinct dates of admixture, with date estimates (both in generations and years from present, with bootstrap 95% CIs given in parenthesis), admixing sources (single best matching sampled surrogate is given first, followed by proportions (>10%) giving more precise inference on the haplotype make-up of the source), and proportion (%) of admixture contributed from each source for each admixture event in the Ari groups under analyses (A), (A-sim) and (B). “Props” gives more stably estimated source compositions than the mixing coefficients where multiple-date admixture is inferred (as in [33]) and are reported for the groups with the two maximal proportions. Here R E refers to the goodness-of-fit (i.e. R 2; see [33] for details) of the model assuming E = 1,2 distinct dates of admixture, and R*2 refers to the additional proportion of remaining fit explained by adding by adding the second date. We note that no analysis here suggests significant evidence of multiple dates of admixture. Assuming a generation time of 28 years, generations g were converted to years y using the formula: y = 1950 − (g + 1) × 28. (PDF) [file pgen.1005397.s022.pdf]

Analysis	Group	**First Event**											
		Date (gen)	Date (years)	%	Source 1		Source 1 Props	%	Source 2		Source 2 Props	R_E	
A	ARIb	13 (1-25)	1599CE (1233CE-1894CE)	18	ORO	ORO(17%),GUM(18%),ANU(24%),AFA(34%)	ORO(0.01),ARIc(0.16)	82	ARIc	ARIc(84%)	AFA(0.04),ANU(0.05)	0.492	
	ARIc	8 (1-16)	1718CE (1483CE-1894CE)	16	ARIb	GUM(13%),ARIb(28%),ORO(38%)	TSI(0.01),AFA(0.01)	84	ORO	ARIb(11%),ORO(65%)	GUM(0.03),ANU(0.04)	0.683	
		**Second Event**											
		Date (gen)	Date (years)	%	Source 1		Source 1 Props	%	Source 2		Source 2 Props	R_E	R_2^*
	ARIb	111 (65-156)	1145BCE (2449BCE-100CE)	33	SOM	GBR(23%),SOM(73%)	LWK(0.03),ARIc(0.20)	67	ARIc	ARIc(100%)	CEU(0.07),SOM(0.13)	0.52	0.056
	ARIc	109 (84-131)	1109BCE (1733BCE-440BCE)	29	ARIb	ANU(11%),GUM(17%),ARIb(56%)	CEU(0.03),AFA(0.06)	71	ORO	ORO(29%),AFA(45%)	GUM(0.05),ARIc(0.07)	0.721	0.119

Analysis	Group	**First Event**											
		Date (gen)	Date (years)	%	Source 1		Source 1 Props	%	Source 2		Source 2 Props	R_E	
A-sim	ARIb	15 (1-24)	1541CE (1257CE-1894CE)	10	ANU	ORO(31%),ANU(59%)	AFA(0.03),ARIc(0.15)	90	ARIc	ORO(23%),ARIc(75%)	LWK(0.05),ANU(0.09)	0.434	
	ARIc	7 (1-17)	1756CE (1438CE-1894CE)	32	ORO	ANU(13%),ORO(80%)	ANU(0.02),ARIc(0.02)	68	ORO	ARIb(18%),ANU(20%),ORO(61%)	TSI(0.01),AFA(0.01)	0.694	
		**Second Event**											
		Date (gen)	Date (years)	%	Source 1		Source 1 Props	%	Source 2		Source 2 Props	R_E	R_2^*
	ARIb	118 (76-160)	1355BCE (2541BCE-201BCE)	24	TSI	ANU(48%),TSI(52%)	AFA(0.06),ARIc(0.2)	76	ARIc	ARIc(99%)	TSI(0.09),CEU(0.11)	0.472	0.067
	ARIc	108 (90-590)	1063BCE (2078BCE-590BCE)	24	ARIb	ANU(37%),ARIb(62%)	CEU(0.03),AFA(0.06)	76	ORO	ANU(12%),ORO(87%)	ANU(0.05),ARIc(0.06)	0.727	0.108

Analysis	Group	**First Event**											
		Date (gen)	Date (years)	%	Source 1		Source 1 Props	%	Source 2		Source 2 Props	R_E	
B	ARIb	14 (1-32)	1552CE (1028CE-1894CE)	41	ORO	ORO(92%)	ANU(0.02),LWK(0.03)	59	MKK	GUM(16%),SOM(40%),MKK(42%)	CEU(0.01),AFA(0.02)	0.395	
	ARIc	9 (3-17)	1696CE (1451CE-1826CE)	40	MKK	GUM(18%),SOM(40%),MKK(42%)	CEU(0.01),AFA(0.02)	60	ORO	SOM(14%),MKK(14%),ORO(72%)	LWK(0.02),ANU(0.02)	0.67	
		**Second Event**											
		Date (gen)	Date (years)	%	Source 1		Source 1 Props	%	Source 2		Source 2 Props	R_E	R_2^*
	ARIb	105 (57-155)	996BCE (2419BCE-328CE)	34	ANU	ANU(100%)	CEU(0.05),AFA(0.05)	66	AFA	SOM(19%),AFA(81%)	LWK(0.08),ANU(0.09)	0.409	0.023
	ARIc	100 (85-125)	849BCE (1565BCE-451BCE)	37	ANU	ANU(95%)	AFA(0.06)	63	AFA	AFA(100%)	LWK(0.07),ANU(0.09)	0.695	0.077
