# Supplementary material for: Evidence for a Common Origin of Blacksmiths and Cultivators in the Ethiopian Ari within the Last 4500 Years: Lessons for Clustering-Based Inference
Source: PLoS Genet. 2015 Aug 20;11(8):e1005397. doi: 10.1371/journal.pgen.1005397 (PMC4546361; doi:10.1371/journal.pgen.1005397)
Supplement: S23 Table — Pairwise F ST [48] between Pop4, Pop5 and Pop5b under a simulation scenario including Pop5a. Simulations differ in the split time (split*) between Pop5 and Pop5a and the length of the Pop5b bottleneck (BN), both in generations (see S27 Fig). In these simulations, {Pop4,Pop5,Pop5b} are meant to mimic the {ORO,ARIc,ARIb}, respectively (PDF) [file pgen.1005397.s023.pdf]

**Simulation**	F_{ST} **(Pop4, Pop5)**	F_{ST} **(Pop5, Pop5b)**	F_{ST} **(Pop4, Pop5b)**
split*=300, BN=30	0.0136	0.0154	0.0279
split*=400, BN=30	0.0136	0.0154	0.0279
split*=500, BN=30	0.0136	0.0154	0.0279
split*=300, BN=35	0.0139	0.0173	0.0304
split*=400, BN=35	0.0139	0.0173	0.0304
split*=500, BN=35	0.0139	0.0173	0.0304
