# Supplementary material for: Evidence for a Common Origin of Blacksmiths and Cultivators in the Ethiopian Ari within the Last 4500 Years: Lessons for Clustering-Based Inference
Source: PLoS Genet. 2015 Aug 20;11(8):e1005397. doi: 10.1371/journal.pgen.1005397 (PMC4546361; doi:10.1371/journal.pgen.1005397)
Supplement: S24 Table — For simulations under the MA model with an unsampled contributing population 5a, the proportion of pairwise F XY scores under analysis (B) between individuals from the same group (i.e. either Pop5b or Pop5, as given in the columns above, which are meant to represent the “ARIb” and “ARIc”, respectively) that are greater than or equal to the mean F XY across all pairwise combinations of individuals with one from Pop5b and the other from Pop5. Each of 6 simulations are given, which vary in the number of generations ago Pop5a and Pop5 split (“split*”) and the number of generations Pop5b is bottlenecked (“BN”). (PDF) [file pgen.1005397.s024.pdf]

**Simulation**	**Pop5b**	**Pop5**
split*=300, BN=30	0.427	0.219
split*=400, BN=30	0.470	0.371
split*=500, BN=30	0.51	0.324
split*=300, BN=35	0.36	0.428
split*=400, BN=35	0.437	0.39
split*=500, BN=35	0.463	0.352
