# Supplementary material for: Evidence for a Common Origin of Blacksmiths and Cultivators in the Ethiopian Ari within the Last 4500 Years: Lessons for Clustering-Based Inference
Source: PLoS Genet. 2015 Aug 20;11(8):e1005397. doi: 10.1371/journal.pgen.1005397 (PMC4546361; doi:10.1371/journal.pgen.1005397)
Supplement: S25 Table — D-statistics testing {ARIc,ARIb;X,Y}, i.e. testing whether {ARIc,ARIb} form a clade relative to {X,Y} using the program qpDstat with default settings, with the corresponding standardized Z statistic showing the strength of evidence for rejecting this tree structure (see [34] for details). We tested all combinations of {X,Y} ∈ {ANU,GUM,LWK,YRI}, as these were the only sampled African groups with < 5% inferred ancestry from a West Eurasian source according to [32]. Note that no test gives ∣Z∣ > 3, suggesting the tree structure {ARIc,ARIb;X,Y} is a good fit to the data in these cases. (PDF) [file pgen.1005397.s025.pdf]

**X**	**Y**	D**-statistic**	Z **statistic**
**LWK**	**YRI**	0.0004	2.803
**ANU**	**LWK**	-0.0003	-2.005
**GUM**	**LWK**	-0.0004	-1.759
**ANU**	**GUM**	0.0001	0.449
**GUM**	**YRI**	-0.0001	-0.264
**ANU**	**YRI**	0.0000	0.099
