# Supplementary figures and images for: Evidence for a Common Origin of Blacksmiths and Cultivators in the Ethiopian Ari within the Last 4500 Years: Lessons for Clustering-Based Inference
Source: PLoS Genet. 2015 Aug 20;11(8):e1005397. doi: 10.1371/journal.pgen.1005397 (PMC4546361; doi:10.1371/journal.pgen.1005397)

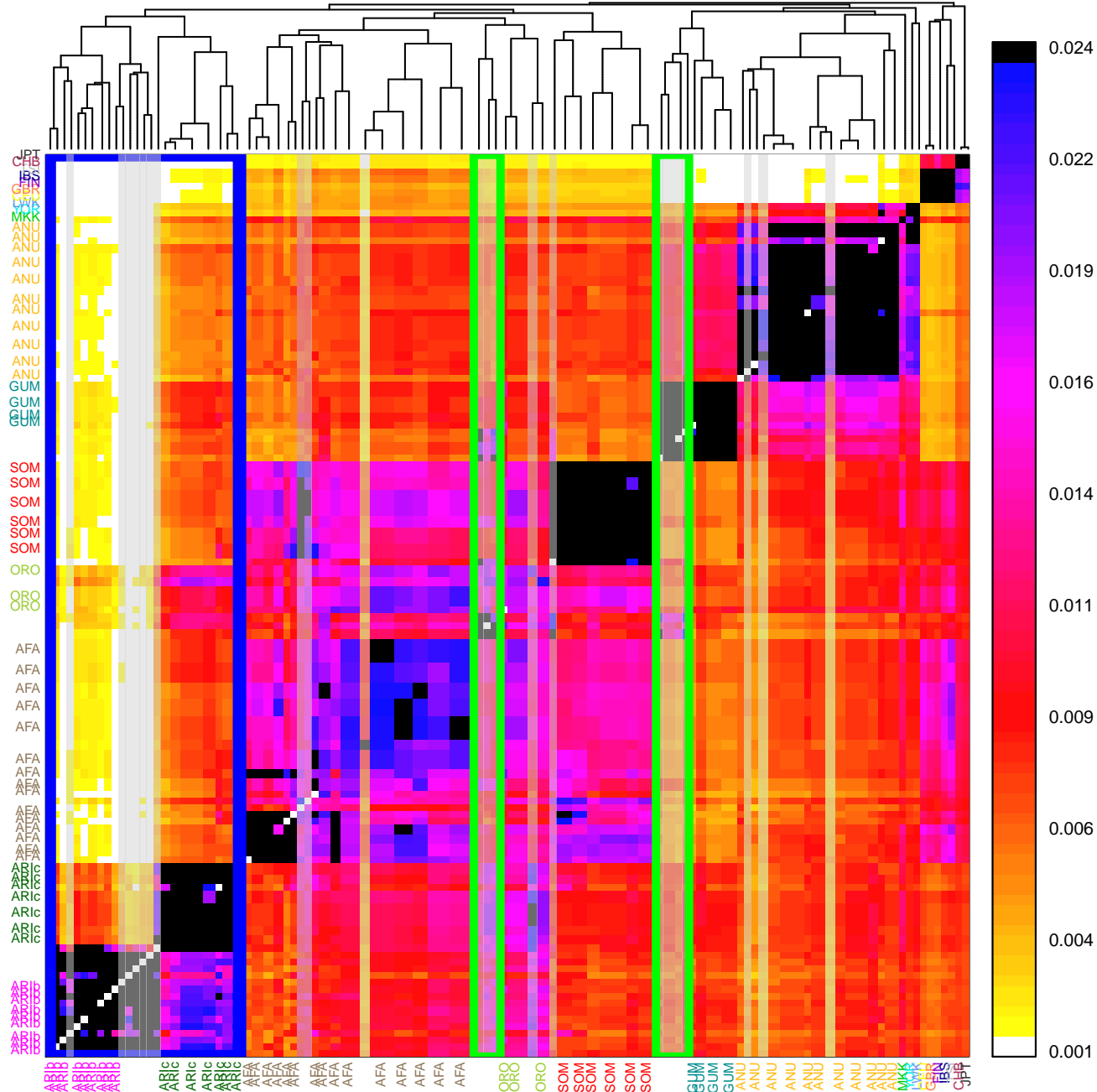

Supplement: S1 Fig — CHROMOPAINTER’s inferred proportion of genome-wide DNA that each of 87 fineSTRUCTURE-inferred clusters (columns) copy from these 87 clusters (rows). Clusters containing any of the Pagani individuals removed from analysis are highlighted with translucent grey vertical bars. The tree at top shows fineSTRUCTURE’s inferred hierarchical merging of these 87 clusters, and colors on the axes show which clusters were assigned to the 17 groups in S1 Table used for analyses. The two Ari groups (ARIb, ARIc) are highlighted with the blue rectangle. The groups highlighted with green rectangles give examples of merged clusters containing 6 Wolayta (left) and 6 Gumuz (right) individuals that were removed due to appearing intermixed among divergent groups (see Methods). (PDF) [file pgen.1005397.s026.pdf]

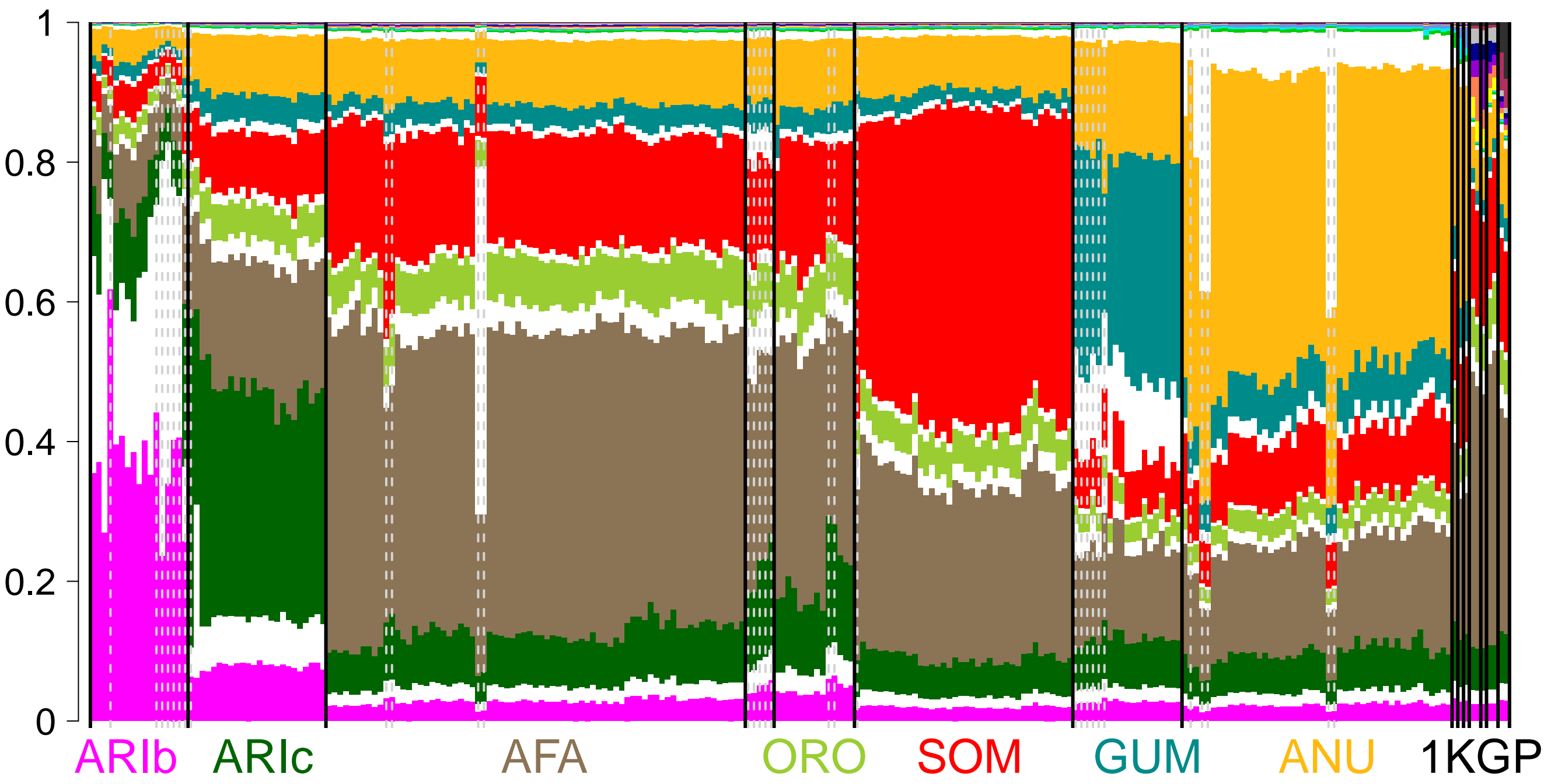

Supplement: S2 Fig — CHROMOPAINTER’s inferred painting profiles for all individuals in the fineSTRUCTURE analysis, showing the proportion of genome-wide DNA that each individual (column) copies from each of the 17 groups (color) in S1 Table (group labels on x-axis, with “1KGP” denoting the ten non-Pagani groups, i.e. the MKK and nine 1000 Genomes groups; color codes in Fig 1 of main text). Grey dashed vertical lines denote excluded individuals; the proportions of DNA copied from any of these individuals are colored in white. Black vertical bars separate the fineSTRUCTURE clusters inferred at a level of the tree where there are K = 15 total clusters, which is the level immediately prior to the Blacksmiths (ARIb) separating into two distinct clusters. (Note that we split some 1KGP clusters from this level and removed one of the 15 clusters to get the 17 final groupings we use in our analyses here.) (PDF) [file pgen.1005397.s027.pdf]

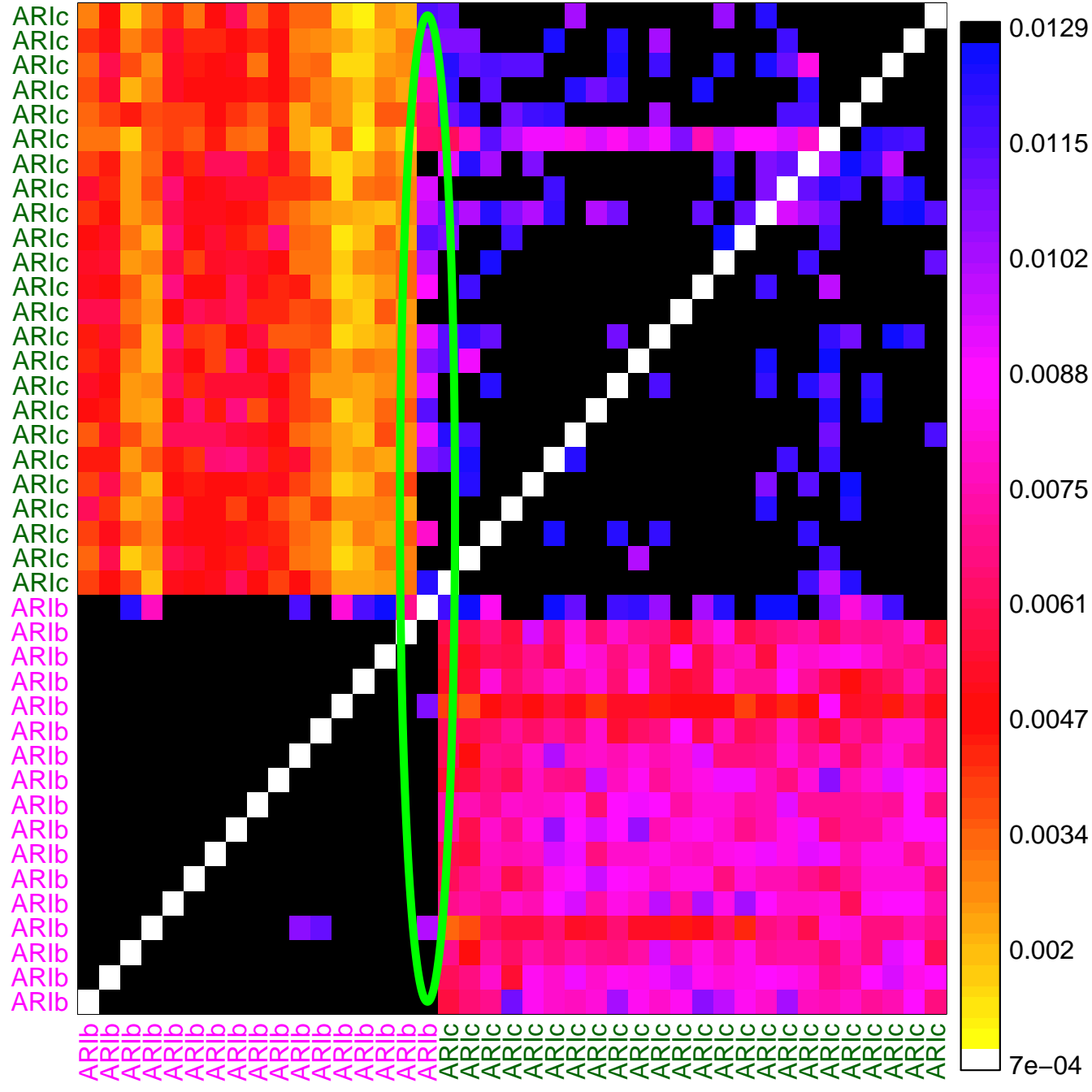

Supplement: S3 Fig — CHROMOPAINTER’s inferred proportion of genome-wide DNA that each Ari individual (column) copies from every other Ari individual (row), for all Ari individuals prior to sample exclusions (except one “Blacksmith” individual that clustered with genetically different groups under fineSTRUCTURE). One of the Ari individuals, labeled a “Blacksmith” (ARIb) and highlighted with the green ellipse, shows clear mixture between Blacksmiths and Cultivators, suggesting that this individual is recently descended from individuals of each of these groups. (PDF) [file pgen.1005397.s028.pdf]

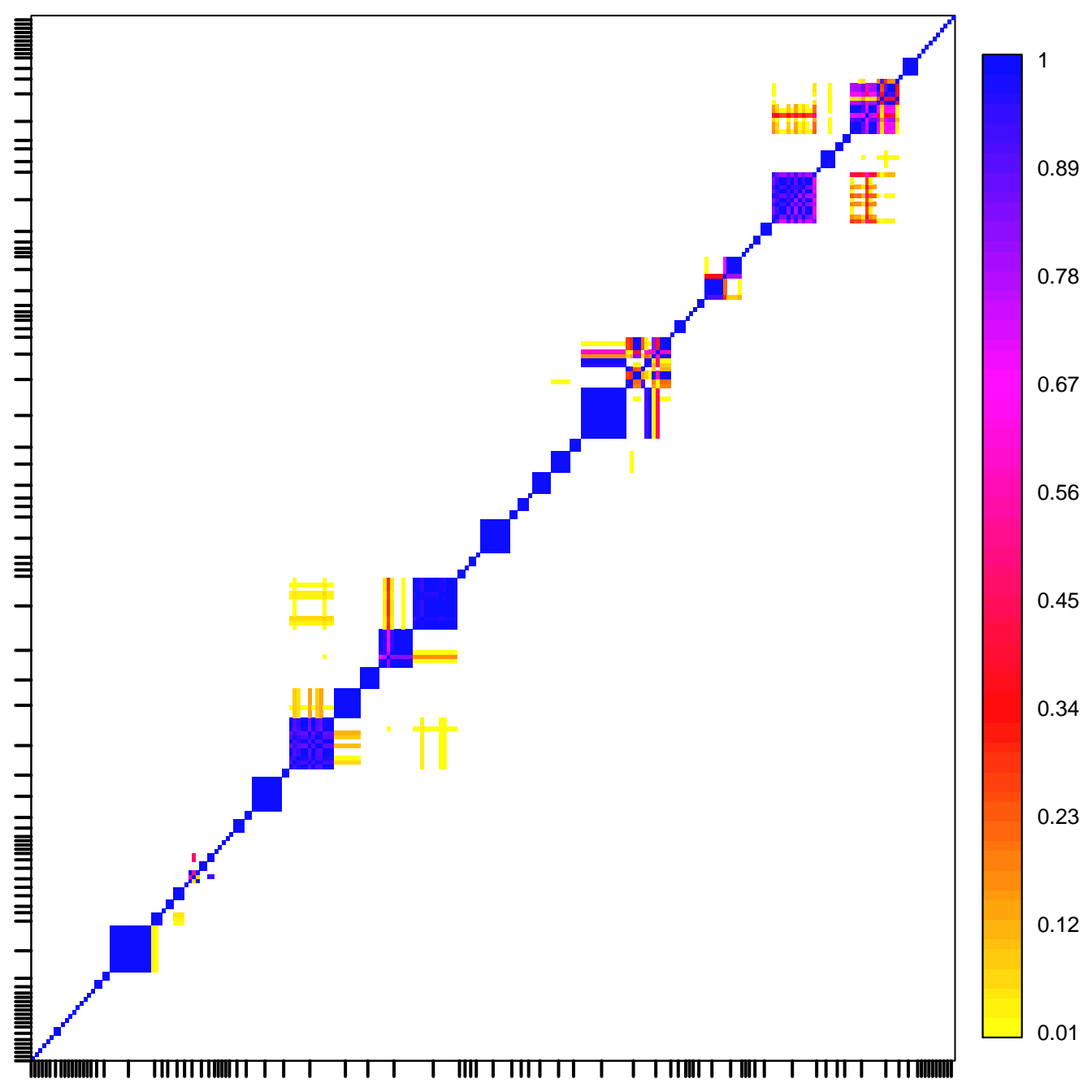

Supplement: S4 Fig — Proportion of MCMC samples for which each individual (or “super individual”) was clustered with each other individual in the fineSTRUCTURE analysis under two independent fineSTRUCTURE runs (separated by the bottom-left to top-right diagonal). Black ticks along each axis denote the midpoints of each of the K = 87 final inferred clusters for one of these fineSTRUCTURE runs. (PDF) [file pgen.1005397.s029.pdf]

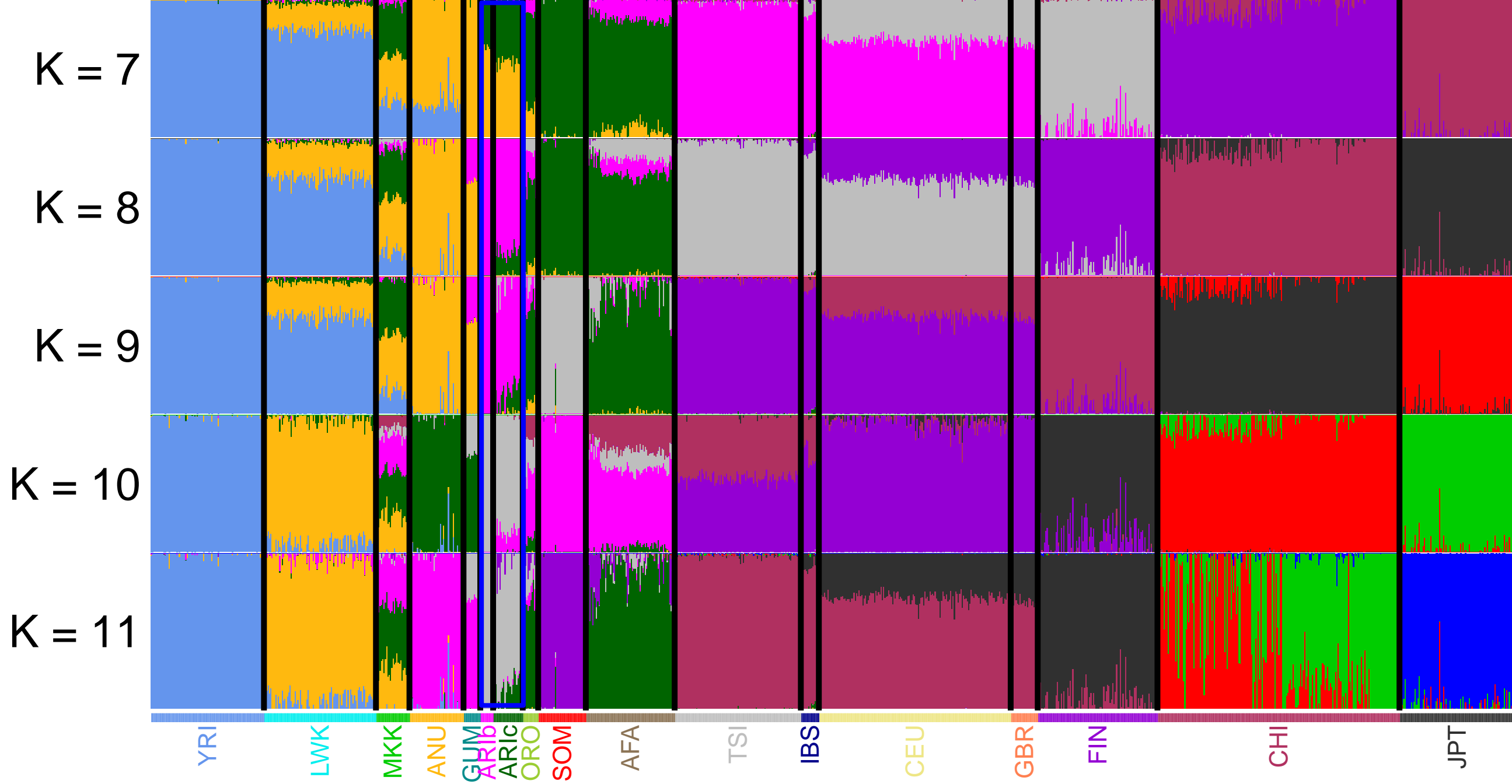

Supplement: S5 Fig — ADMIXTURE applied to individuals from the seven Pagani groups [2], the MKK and groups from the 1000 Genomes Project using various numbers of clusters K; labels are based on the 17 inferred fineSTRUCTURE groups. The two Ari groups (ARIb, ARIc) are highlighted with the blue rectangle. (PDF) [file pgen.1005397.s030.pdf]

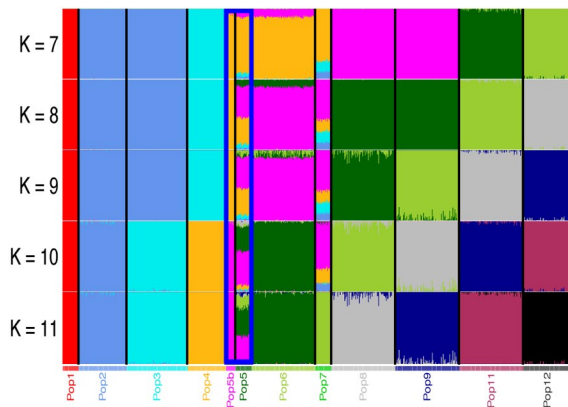

(a) MA “full” simulations

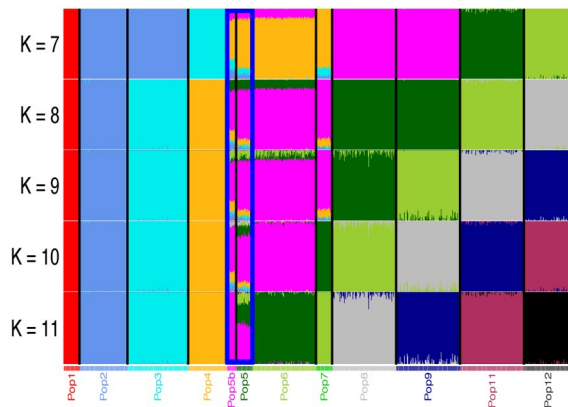

(b) RN “full” simulations

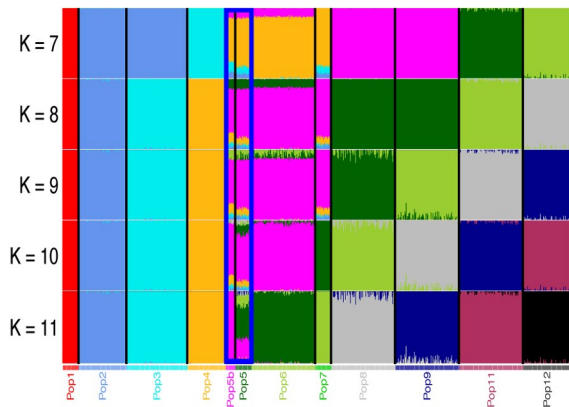

(c) RN+BN “full” simulations

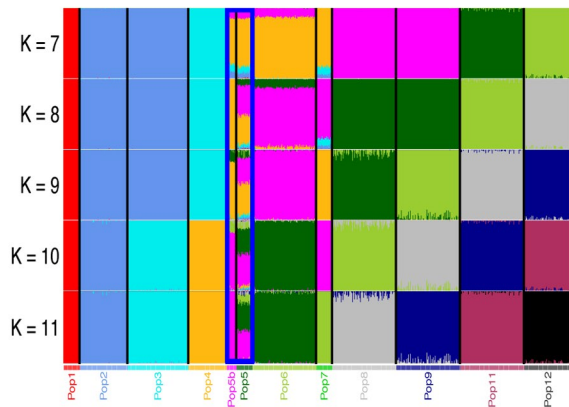

(d) RN+BN+80% “full” simulations

Supplement: S6 Fig — ADMIXTURE results for “full” simulations under the (a) “Marginalisation (MA)”, (b) “Remants (RN)”, (c) “Remnants + Bottleneck (RN+BN)” and (d) “Remnants + Bottleneck + 80% Mixture (RN+BN+80%)” models (see Methods), with “ARIc” = Pop5 and “ARIb” = Pop5b, including all individuals and using various numbers of cluster K. Pop5 and Pop5b are highlighted with the blue rectangle. Note that Pop5 looks like a mixture of ancestries related to Pop5b and other populations for various K across all four simulations (e.g. for K = 11). (PDF) [file pgen.1005397.s031.pdf]

**(A) all-donors**

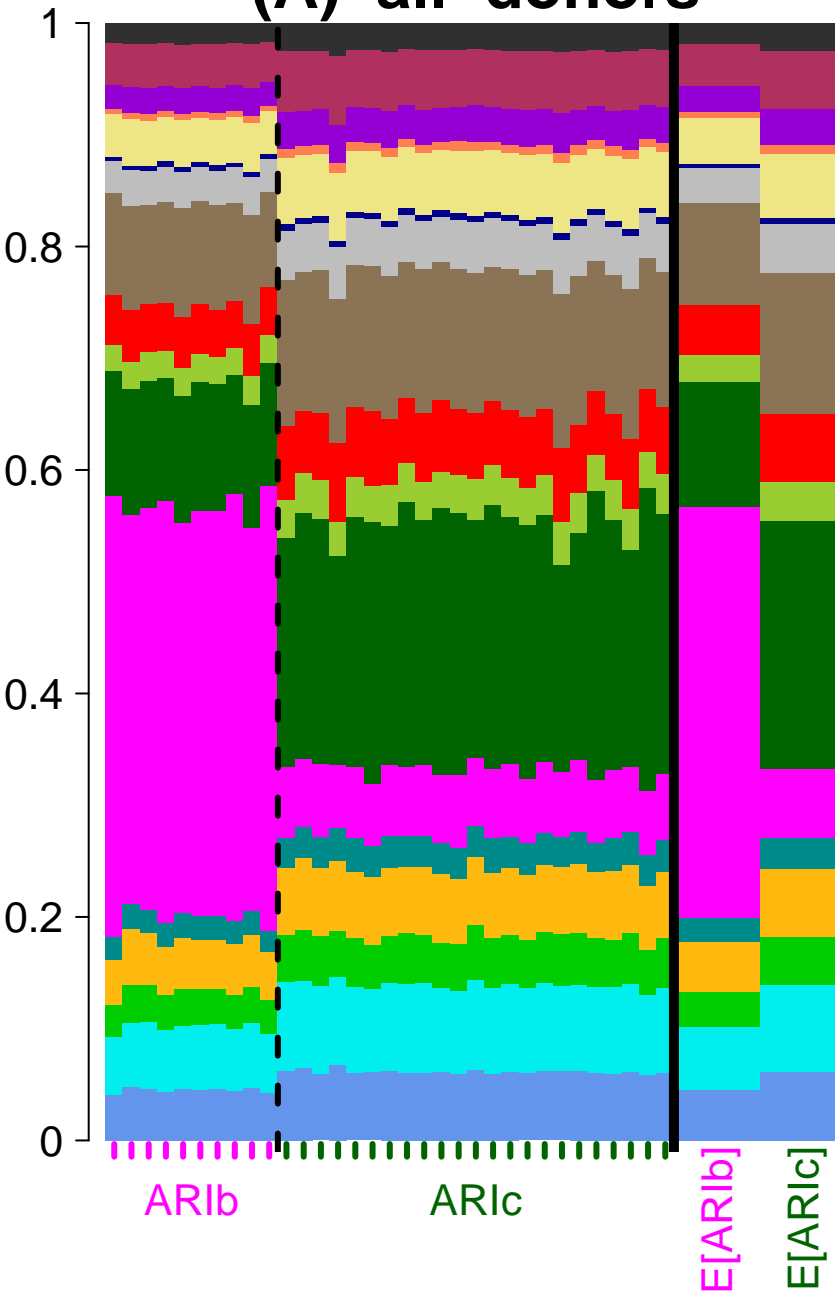

**(B) non-Ari-donors**

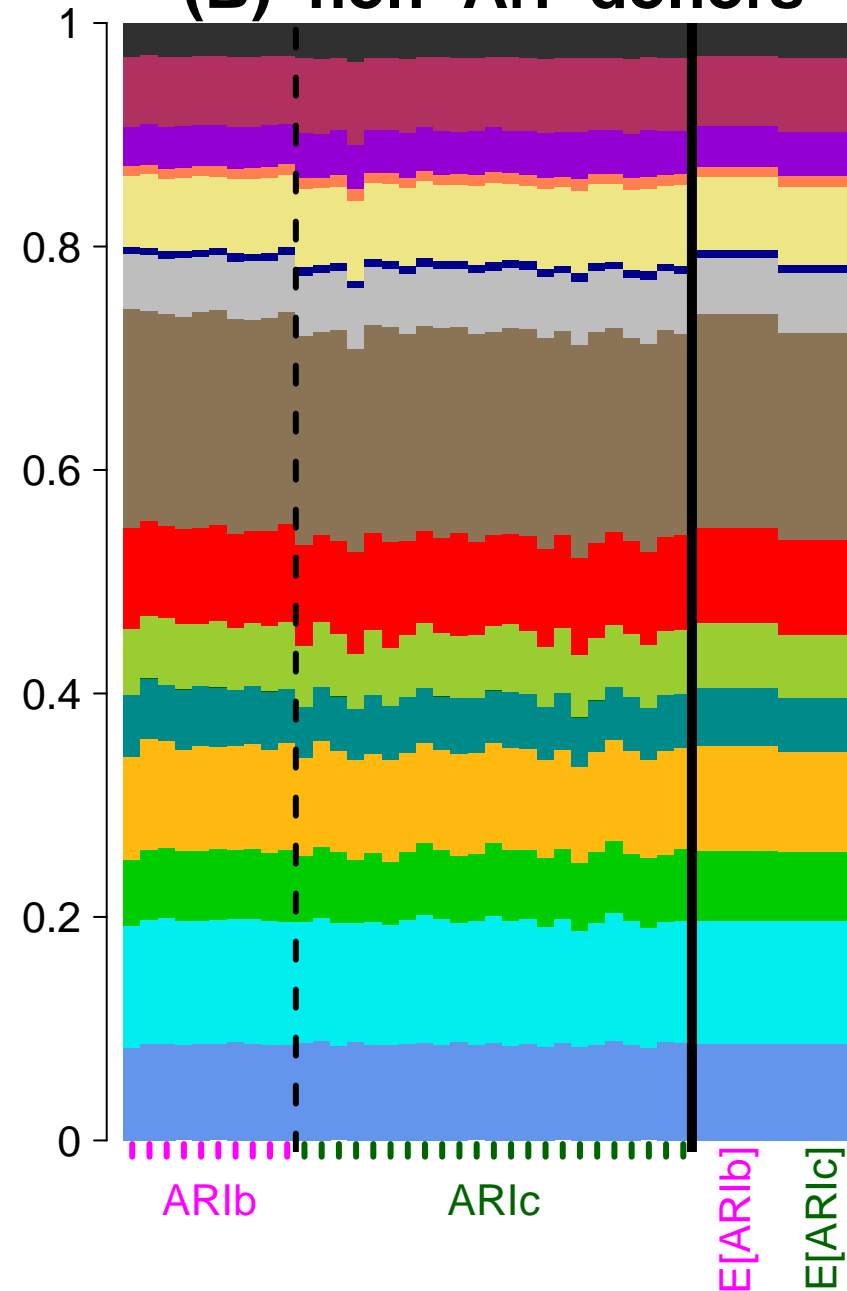

**(C) non-Pagani-donors**

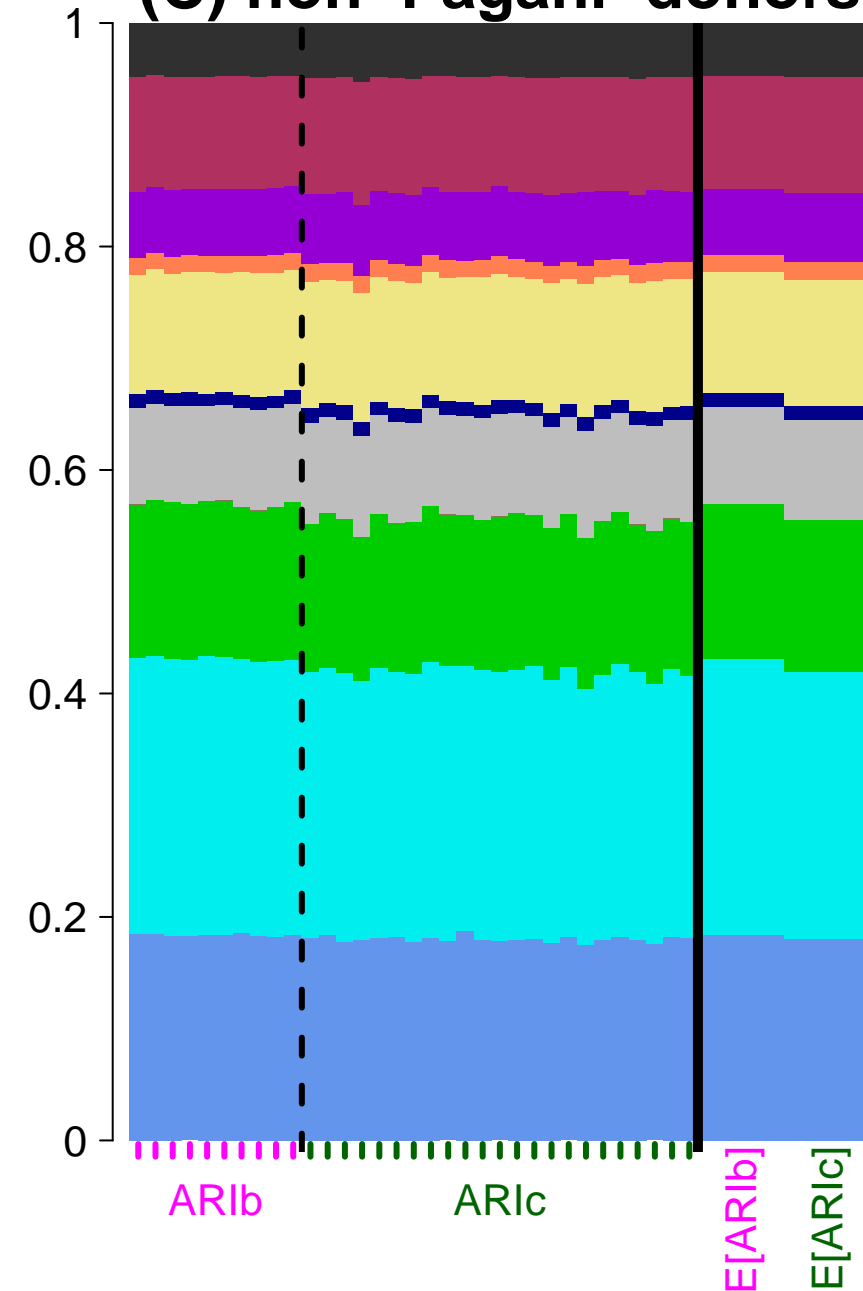

Supplement: S7 Fig — CHROMOPAINTER’s inferred painting profiles for each Ari individual, showing the proportion of DNA copied from each world-wide donor group (color), under each of analyses (A)-(C). Group labels (ARIb/ARIc) are given on the x-axis, with group means (E[ARIb],E[ARIc]) at far right. Donor groups are colored according to Fig 1a of the main text. (PDF) [file pgen.1005397.s032.pdf]

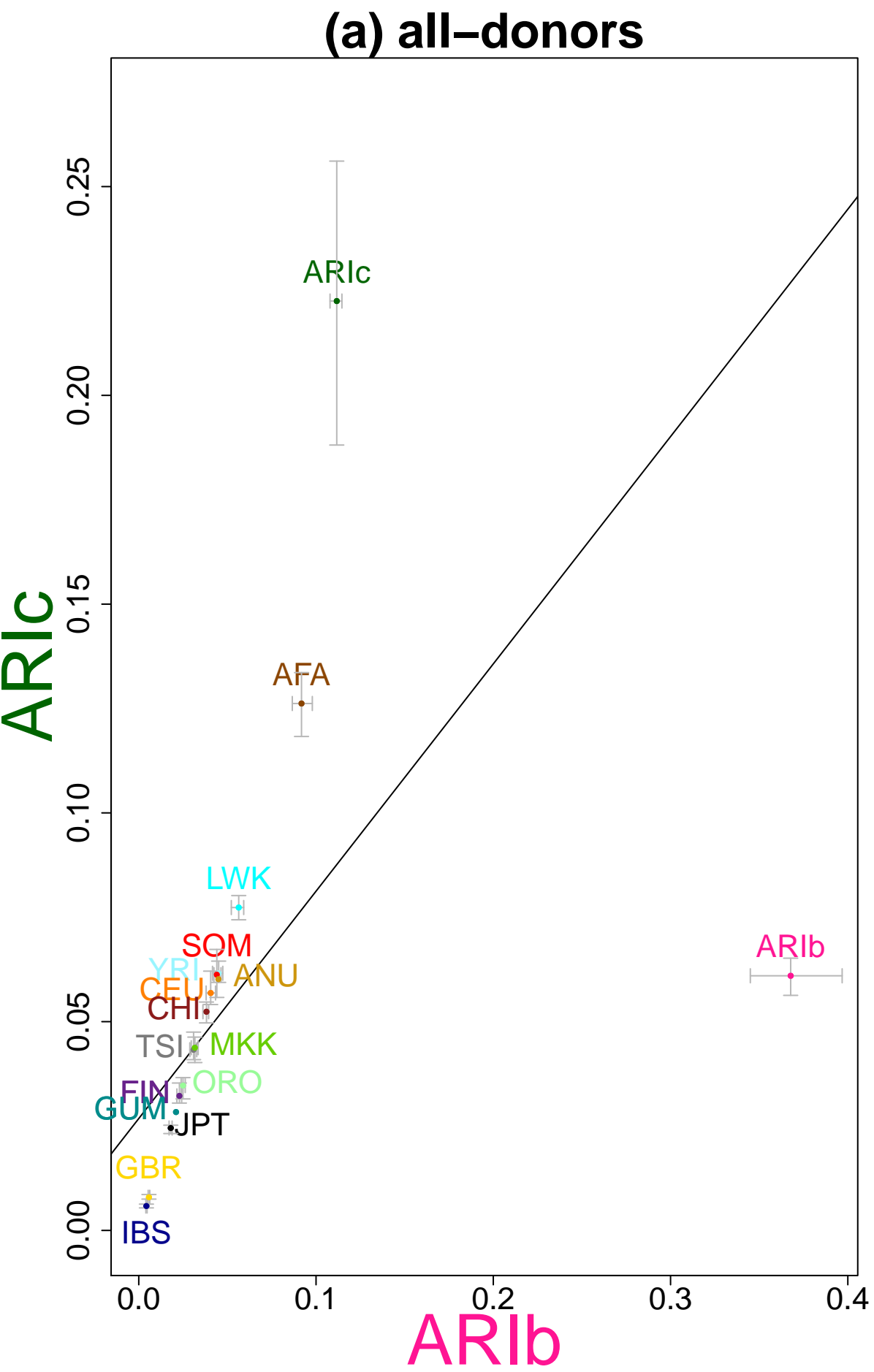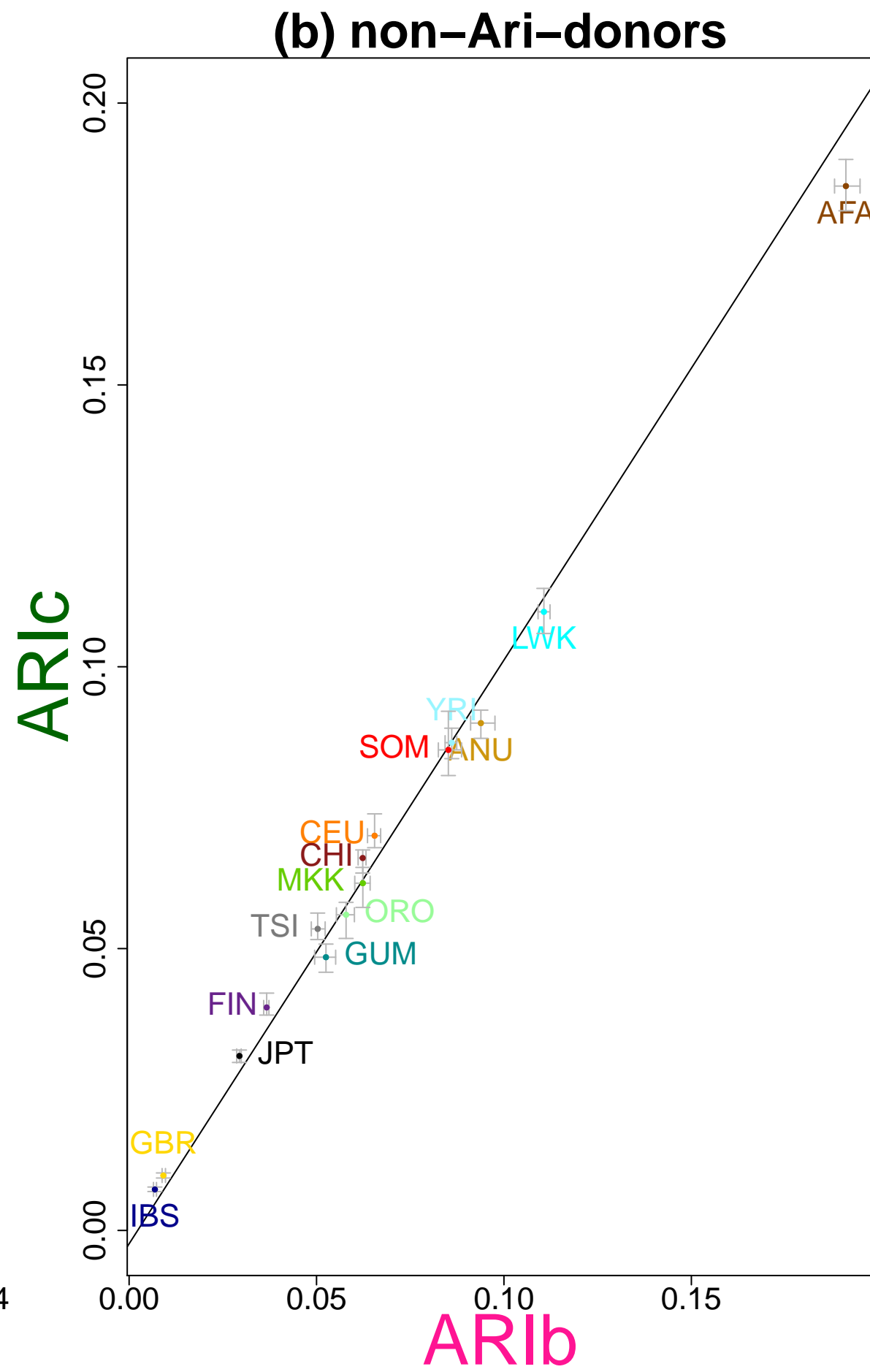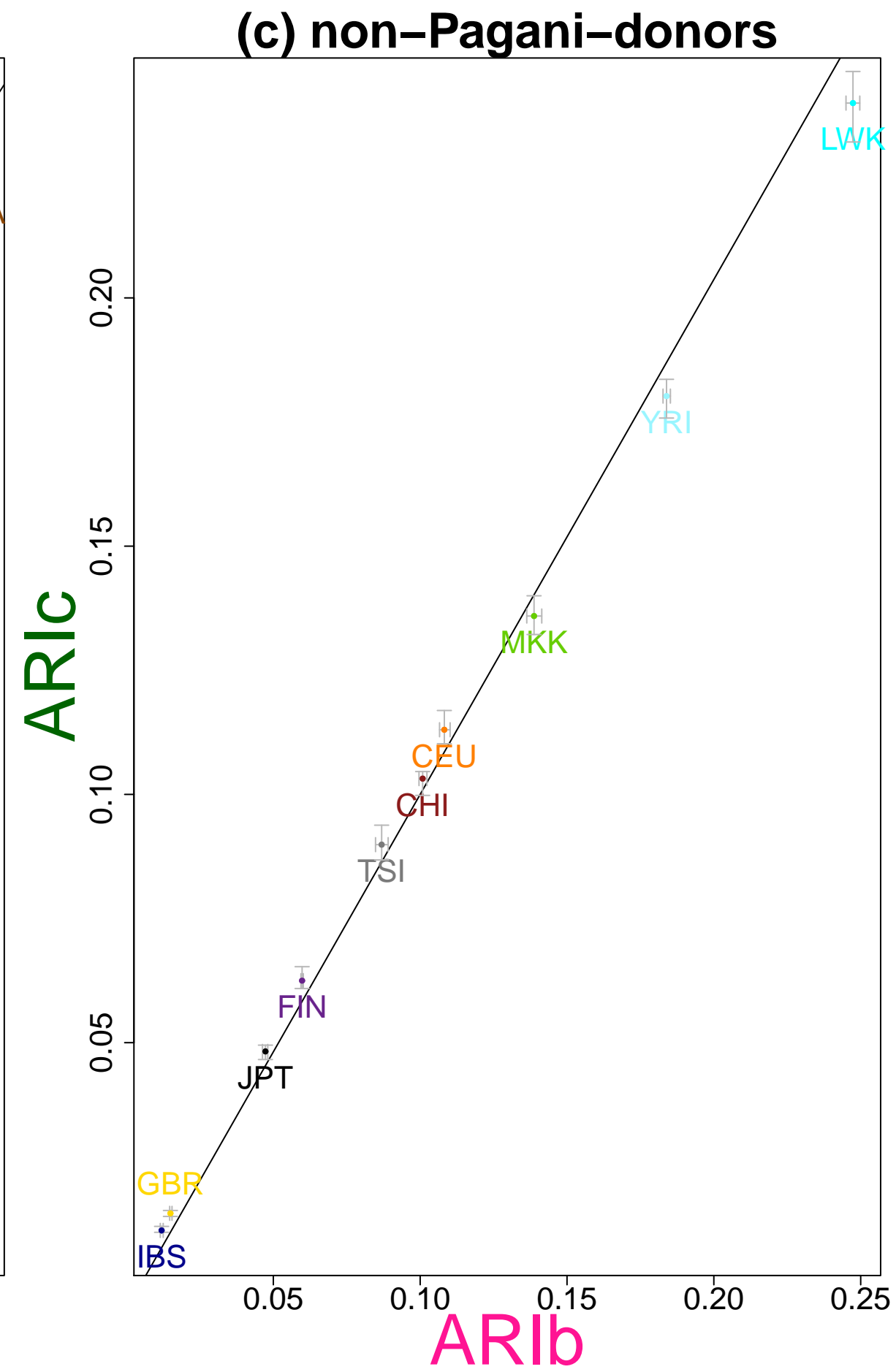

Supplement: S8 Fig — Average proportion of DNA that the ARIb (x-axis) and ARIc (y-axis) copy from each donor group under CHROMOPAINTER analyses (A)-(C). Inner 95% empirical quantiles across individuals within each Ari group are shown with the vertical and horizontal lines. Donor groups are colored according to Fig 1a of the main text. (PDF) [file pgen.1005397.s033.pdf]

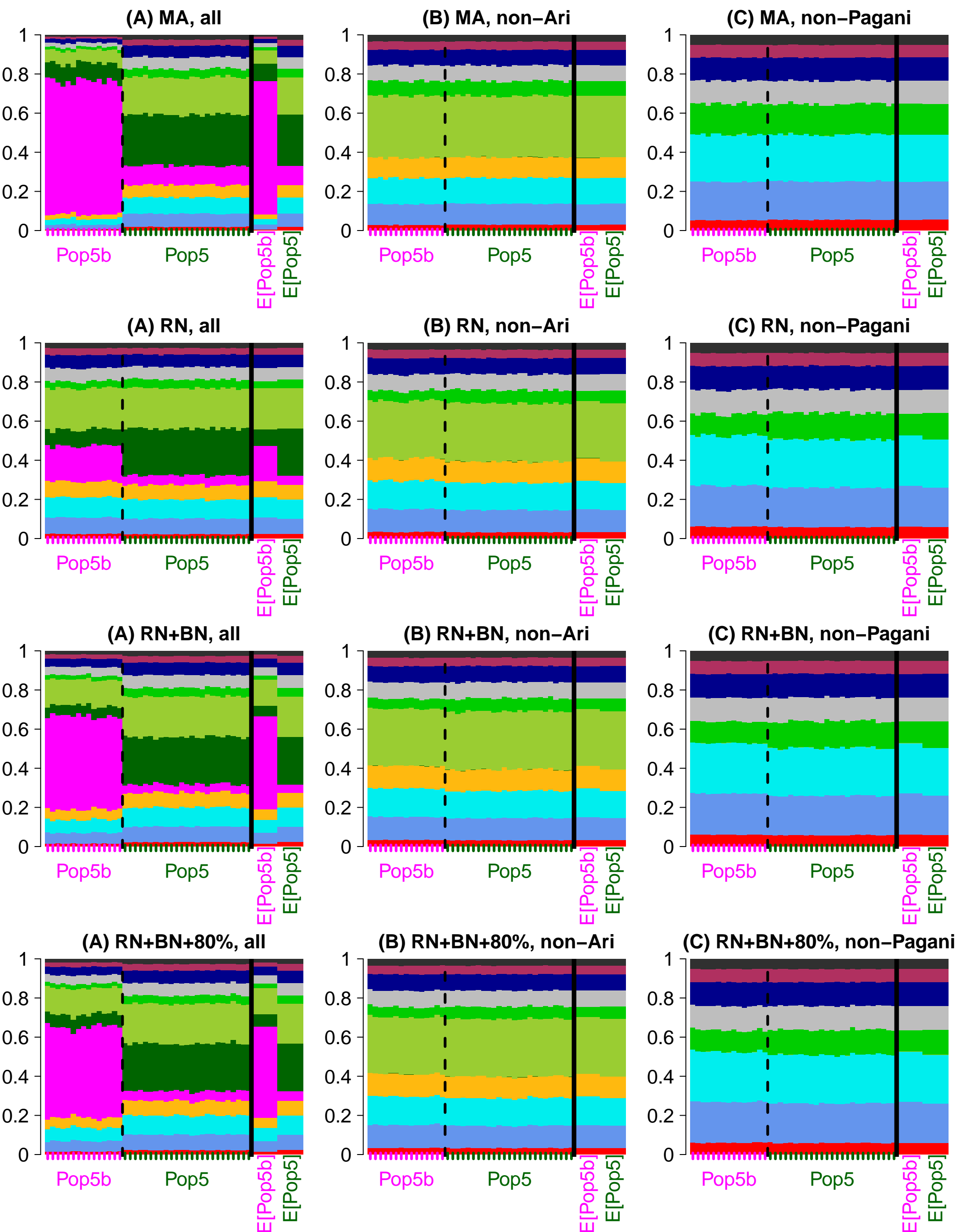

Supplement: S9 Fig — CHROMOPAINTER’s inferred painting profiles for each simulated “Ari” individual in the four different “full” simulations (see Methods), showing the proportion of DNA copied from each simulated donor group (color), under each of analyses (A)-(C). Group labels (Pop5b = “ARIb” / Pop5 = “ARIc”) are given on the x-axis, with group means (E[Pop5b],E[Pop5]) at far right. Donor groups are colored according to x-axis labels in S12 Fig. (PDF) [file pgen.1005397.s034.pdf]

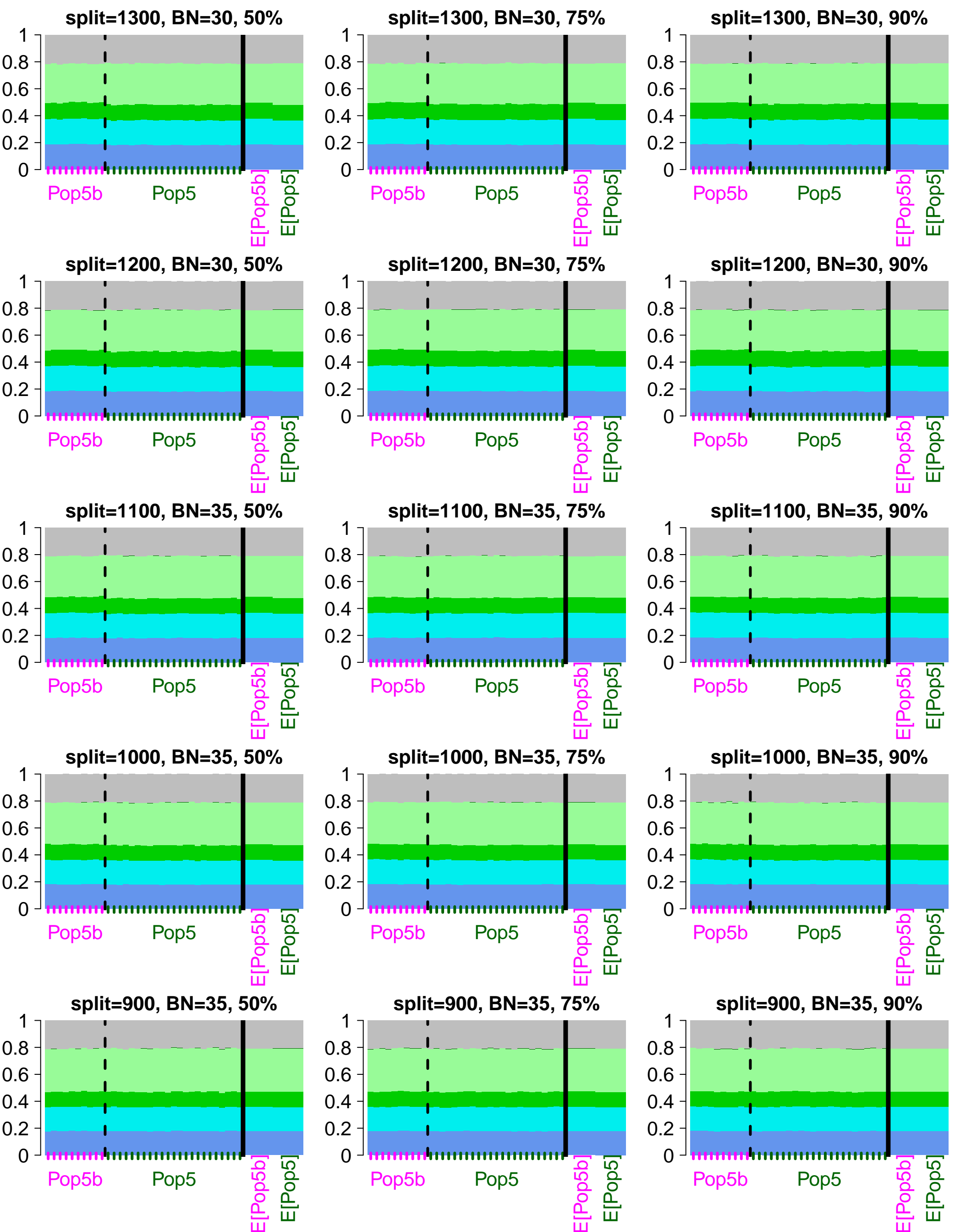

Supplement: S10 Fig — CHROMOPAINTER’s inferred painting profiles for each simulated “Ari” individual in 15 different “simplified” simulations (see Methods), showing the proportion of DNA copied from each simulated donor group (color). Group labels (Pop5b = “ARIb” / Pop5 = “ARIc”) are given on the x-axis, with group means (E[Pop5b],E[Pop5]) at far right. The titles above each plot describe the simulation parameters, which vary in the number of generations ago Pop5 and Pop5b split (“split”), the number of generations Pop5b is bottlenecked (“BN”), and the proportion of migrants contributed from Pop5b to Pop5 (%). Results are shown for CHROMOPAINTER analysis (B) using all non-Pop5 groups only as donors. Each distinct color denotes the inferred painting from a distinct group, with the legend for each non-Pop5 group given in the third bar of the top left plot of S13 Fig. (PDF) [file pgen.1005397.s035.pdf]

**(A) all-donors**

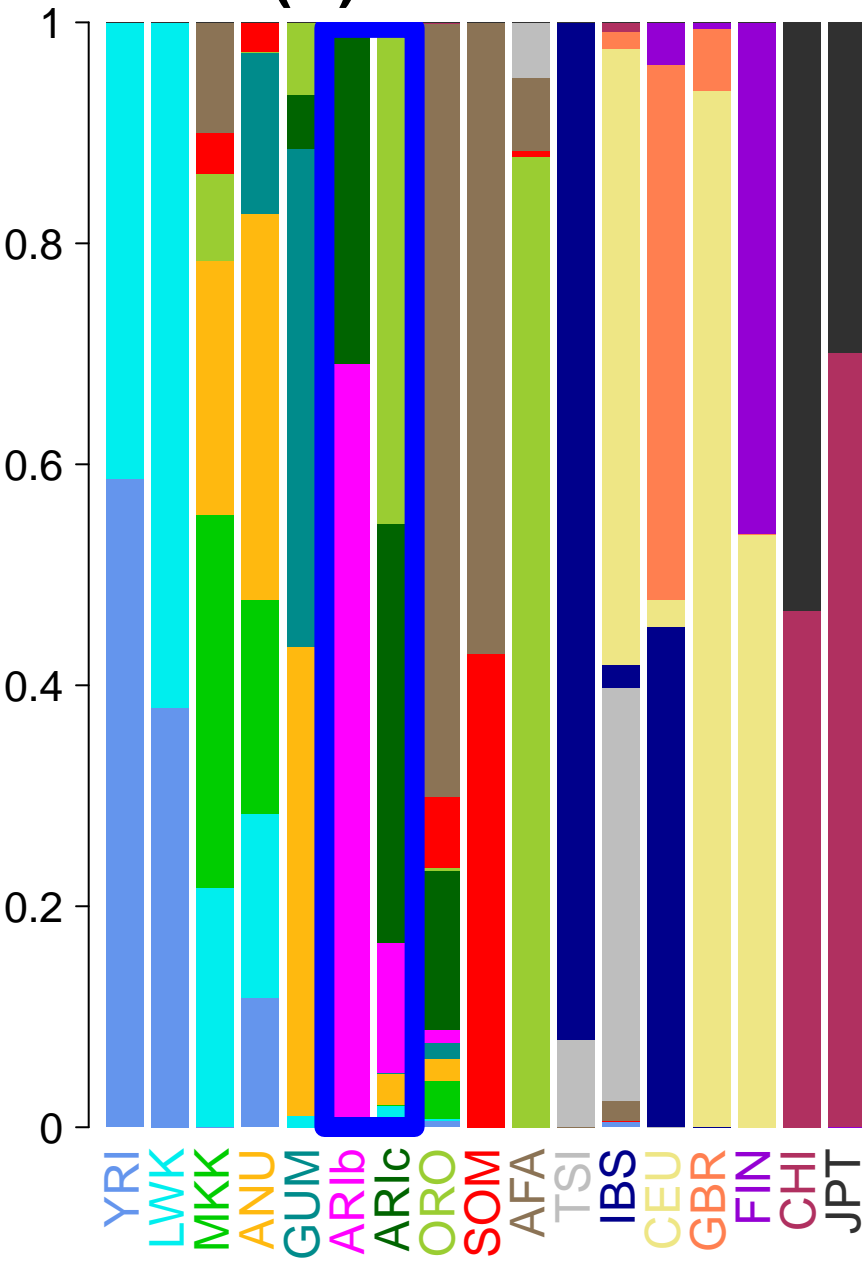

**(B) non-Ari-donors**

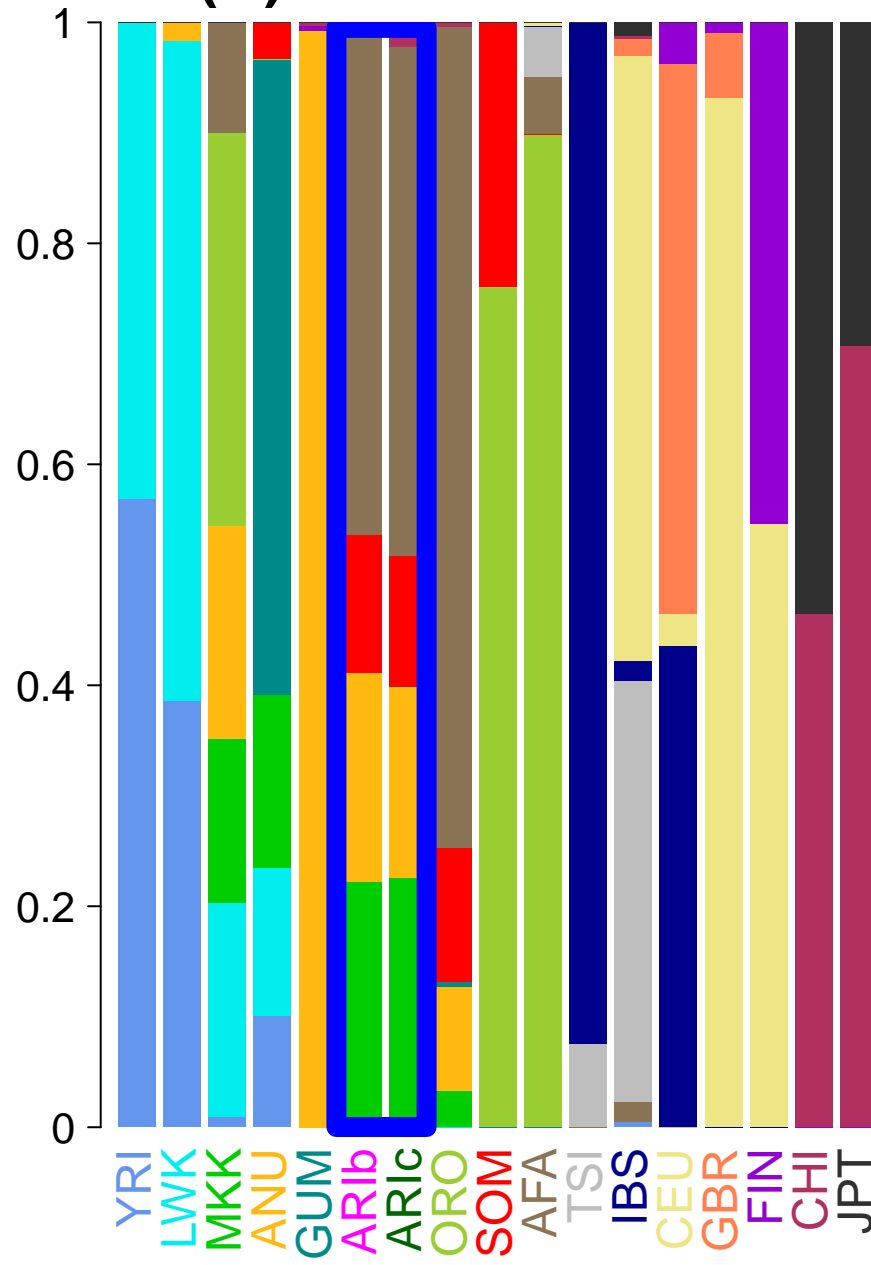

**(C) non-Pagani-donors**

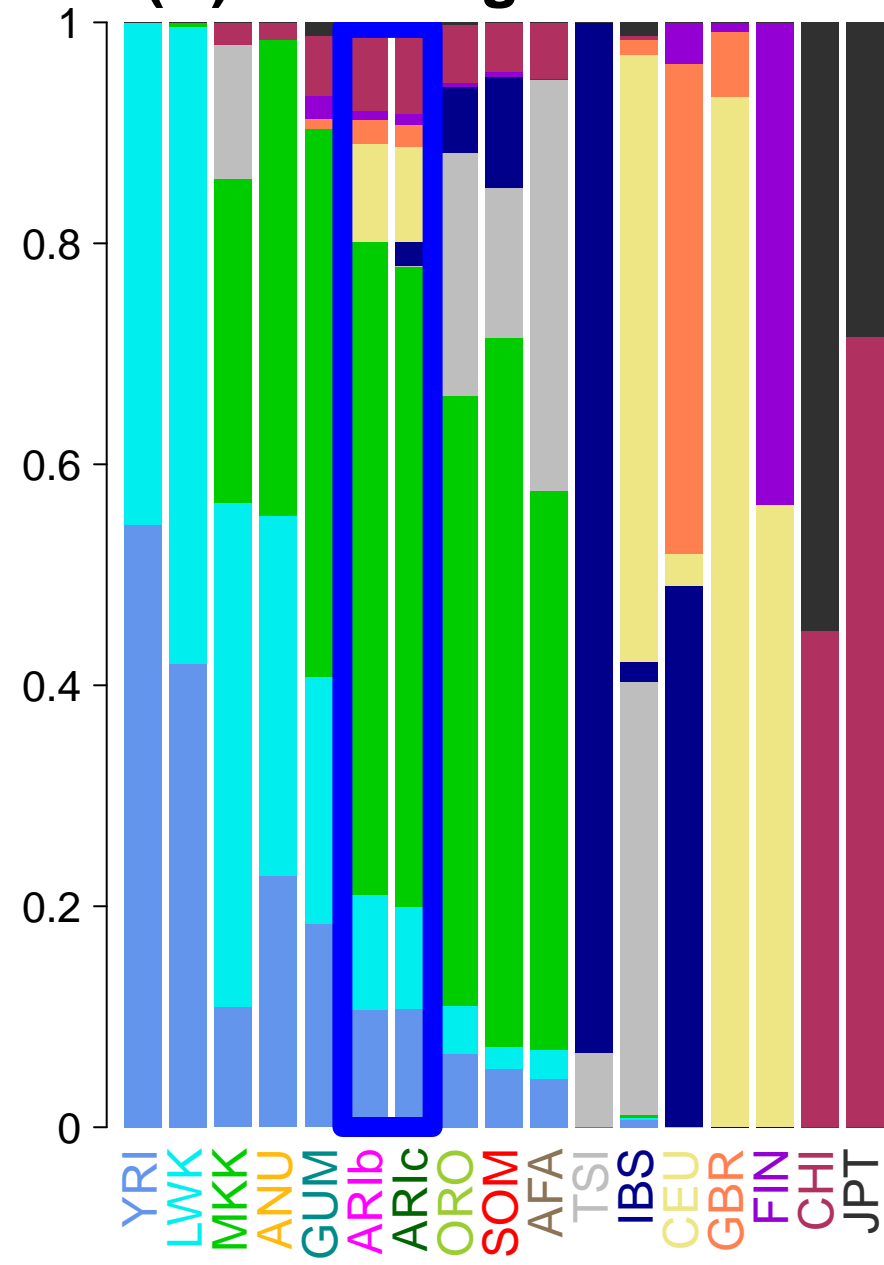

Supplement: S11 Fig — Proportions of ancestry each Pagani and 1KGP+MKK group (column) shares with other groups, matching Fig 3 of main text as inferred using CHROMOPAINTER and linear modelling (color codes in Fig 1a of main text). The two Ari groups are enclosed in a blue border. Under analysis (A) including all groups as donors, the ARIb have “self-copying” levels higher than any other group, indicative of strong drift effects. Under analyses that exclude the Ari groups as donors (B) or exclude all non-Pagani groups as donors (C), the ARIb and ARIc look genetically similar when ignoring this drift, suggesting recent shared ancestry. (PDF) [file pgen.1005397.s036.pdf]

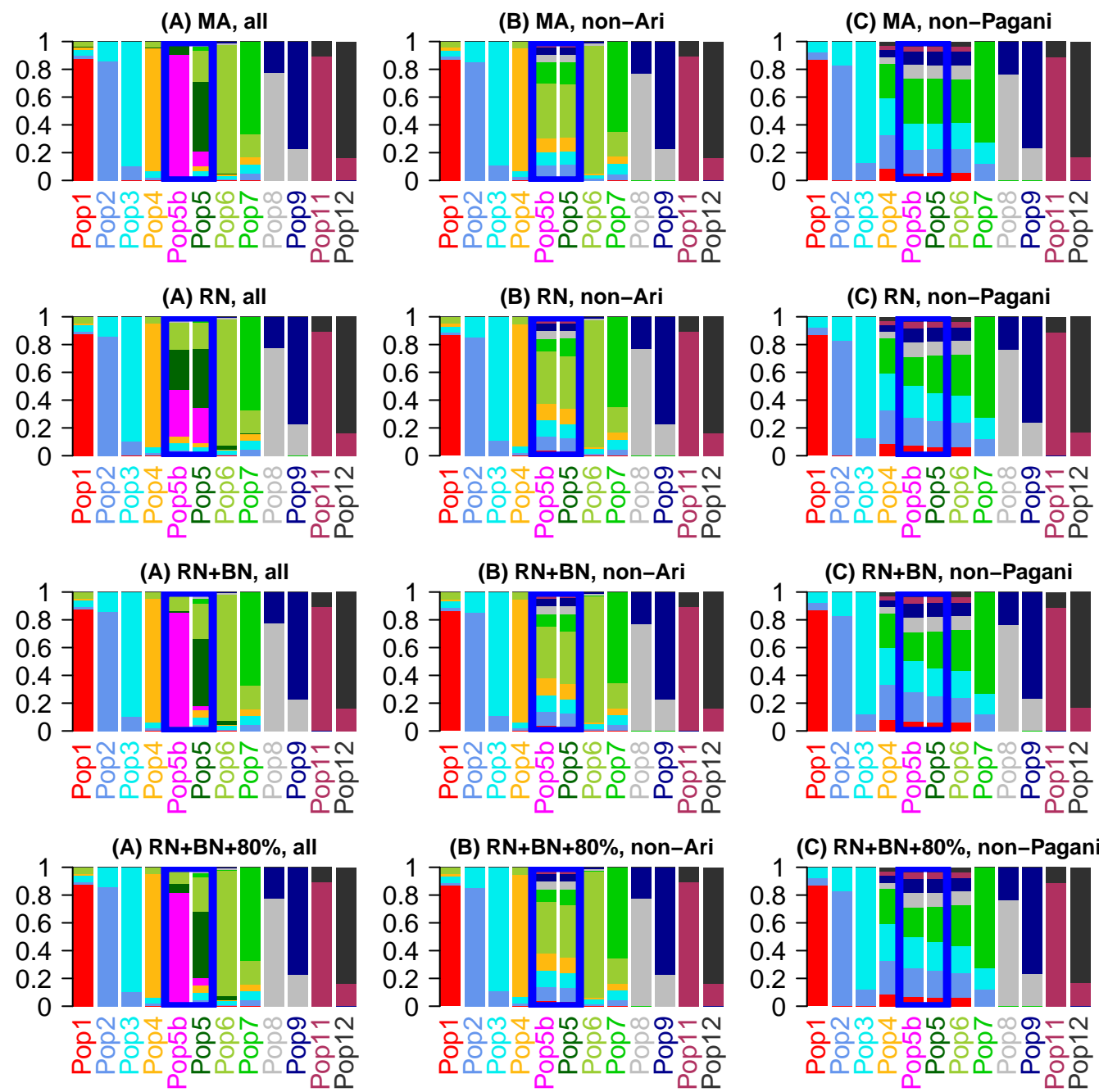

Supplement: S12 Fig — Proportions of ancestry, as inferred using CHROMOPAINTER and linear modelling, for simulations under the Marginalisation (MA; top row) and three different Remants (RN; rows 2–4) models in the “full” simulations (see Methods) for each of CHROMOPAINTER analyses (A)-(C), with groups Pop5 = “ARIc” and Pop5b = “ARIb” enclosed in a blue border. Note that the MA model (top row) shows nearly identical inference for Pop5 and Pop5b under CHROMOPAINTER in analyses (B) and (C), contrasting with the three RN models. (PDF) [file pgen.1005397.s037.pdf]

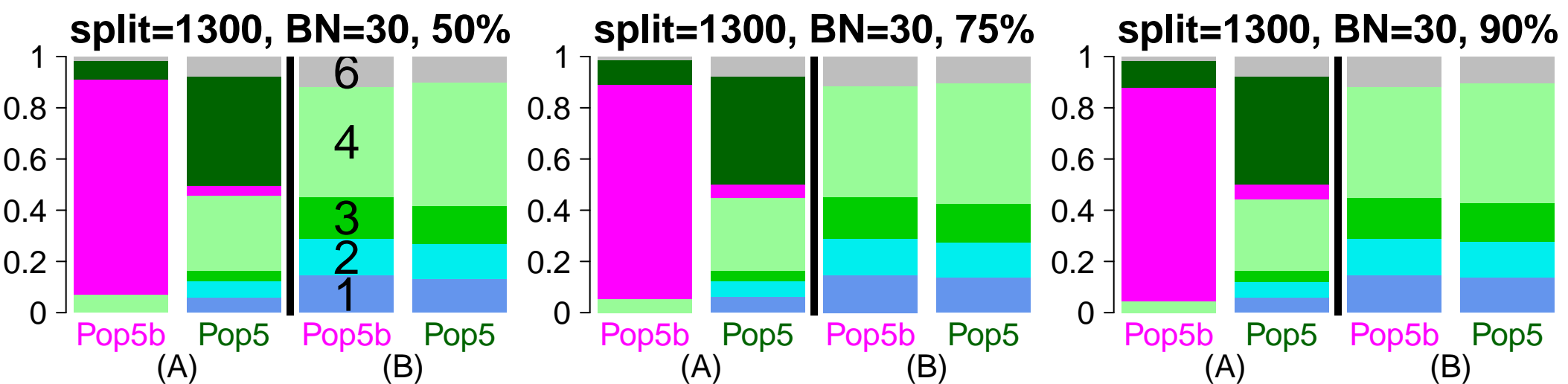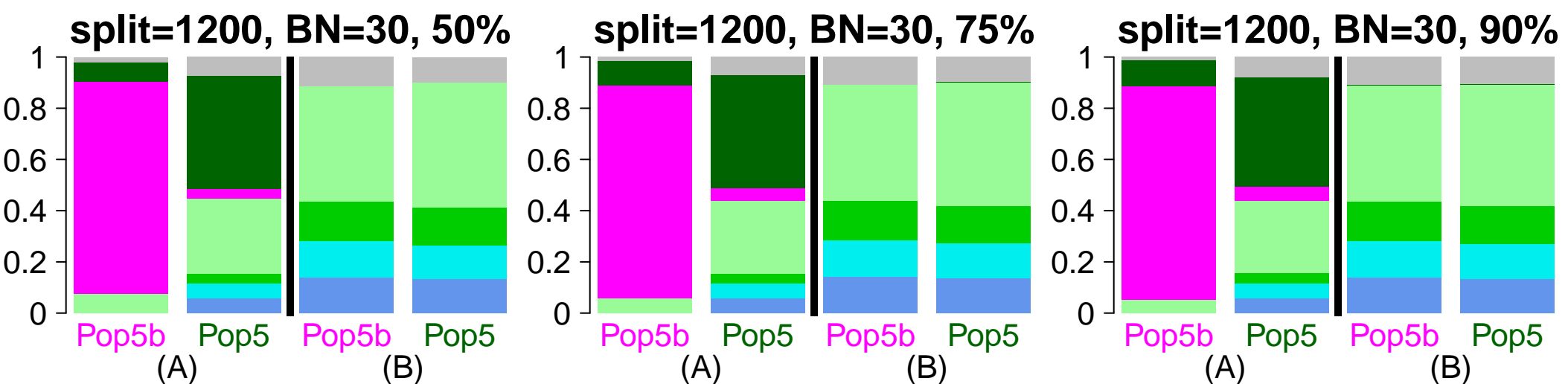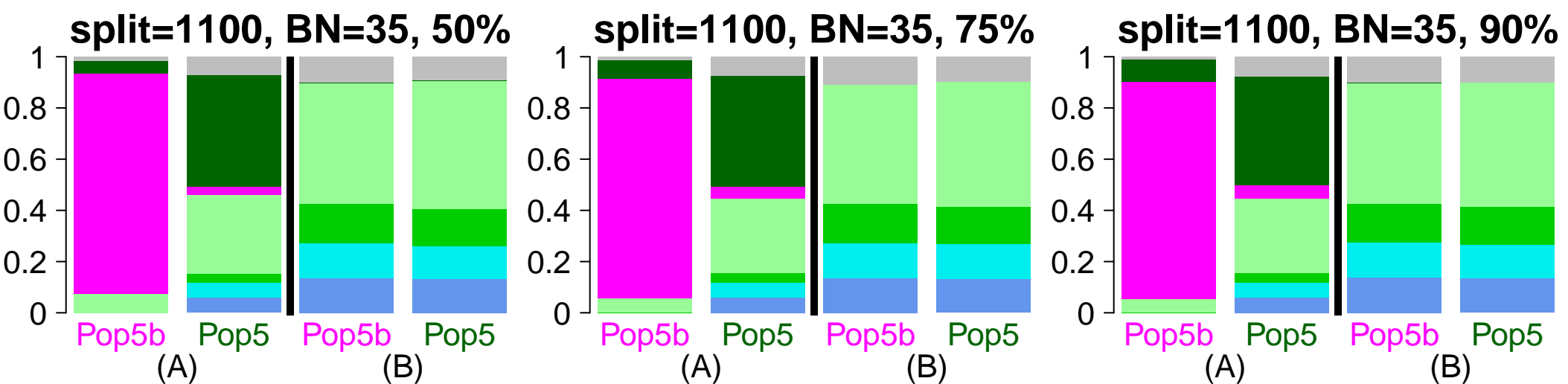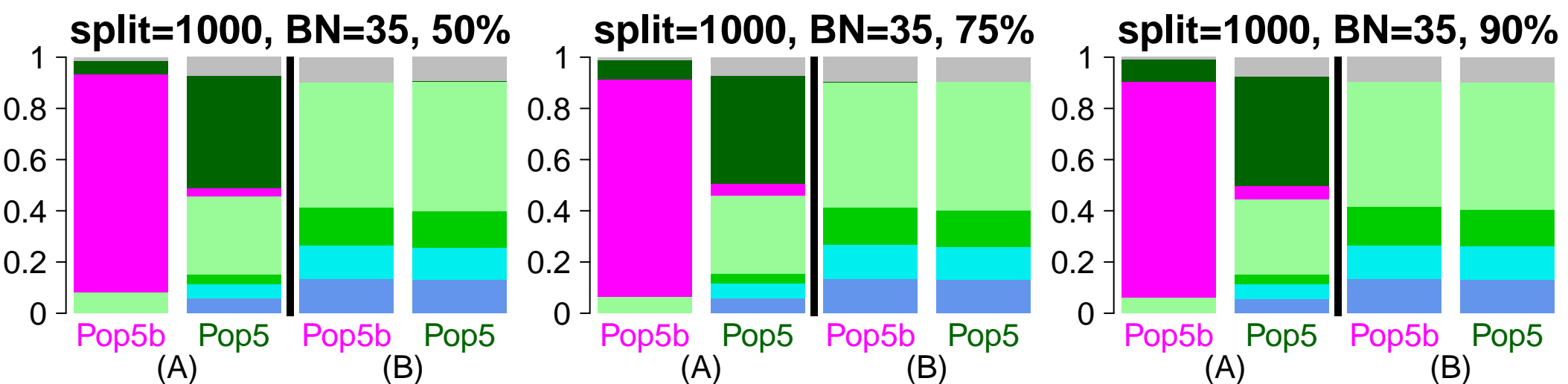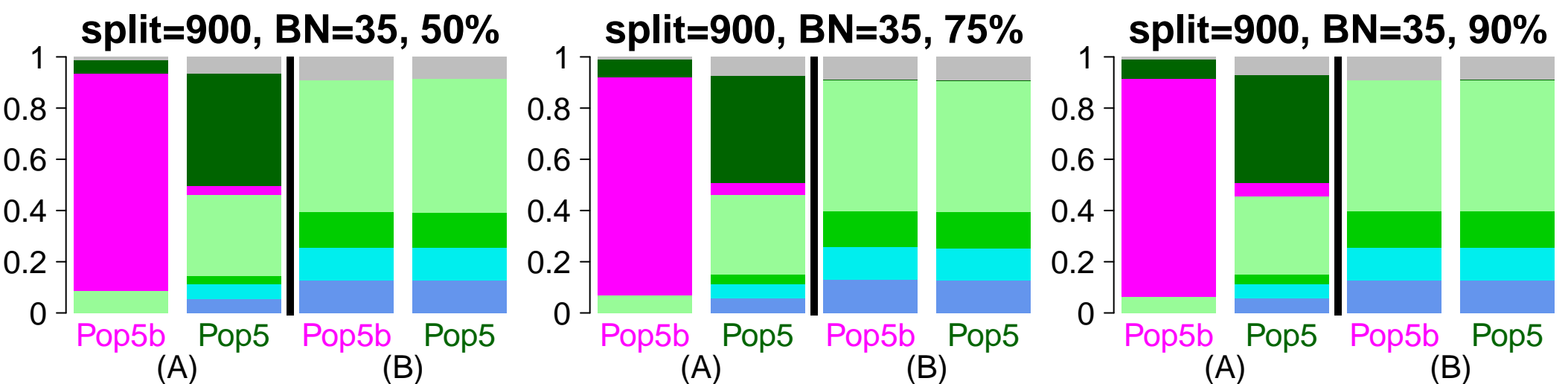

Supplement: S13 Fig — Proportions of ancestry, as inferred using CHROMOPAINTER and linear modelling, for simulations under the simplified Remnants simulations (see Methods). Pop5b is meant to represent “ARIb” and Pop5 is meant to represent “ARIc”. The titles above each plot describe the simulation parameters, which vary in the number of generations ago Pop5 and Pop5b split (“split”), the number of generations Pop5b is bottlenecked (“BN”), and the proportion of migrants contributed from Pop5b to Pop5 (%). Results are shown for CHROMOPAINTER analyses using (A) all available groups as donors to describe ancestry and (B) using all non-Pop5 groups only. Each distinct color denotes the inferred ancestry from a distinct group, with the legend for each non-Pop5 group given in the third bar of the top left plot. (PDF) [file pgen.1005397.s038.pdf]

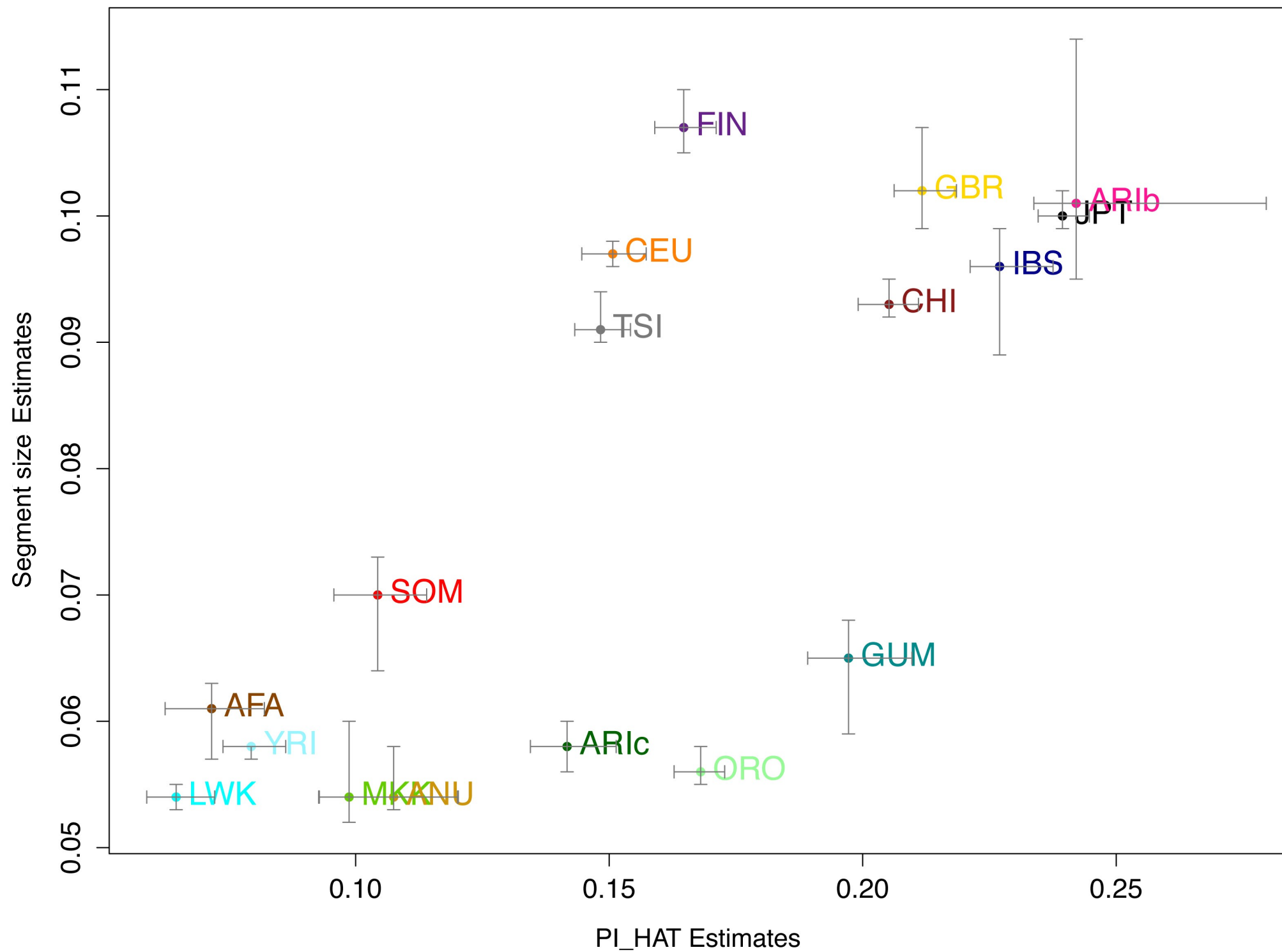

Supplement: S14 Fig — CHROMOPAINTER’s inferred average haplotype segment sizes (in cM) from S14 Table, i.e. when allowing individuals to copy only from members of their own group, versus PI_HAT values inferred by PLINK v1.07 [28] from S13 Table for the same groups. Median and 95% empirical quantile values across all individuals (“segment size”) or across all pairwise comparisons of individuals (“PI_HAT”) are shown. Each technique measures homogeneity within a group, though note that the haplotype-based technique clearly separates African and non-African groups, with the exception of the likely bottlenecked Blacksmiths (ARIb), while ignoring haplotypes leads to several non-African groups having lower values than African groups, likely due to ascertainment bias. These patterns were similar when inferring PI_HAT values after sub-sampling SNPs to decrease linkage disequilibrium levels (S13 Table). (PDF) [file pgen.1005397.s039.pdf]

**(A) all-donors**

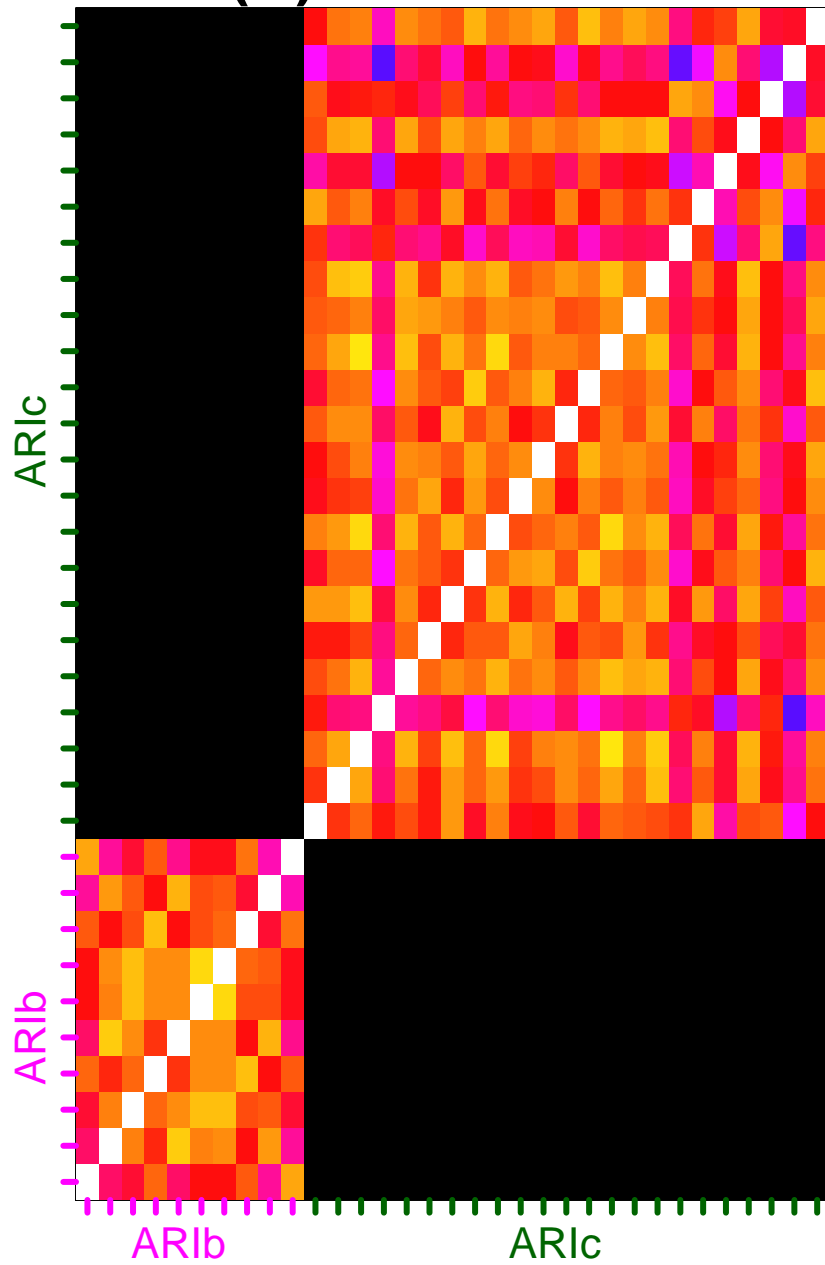

**(B) non-Ari-donors**

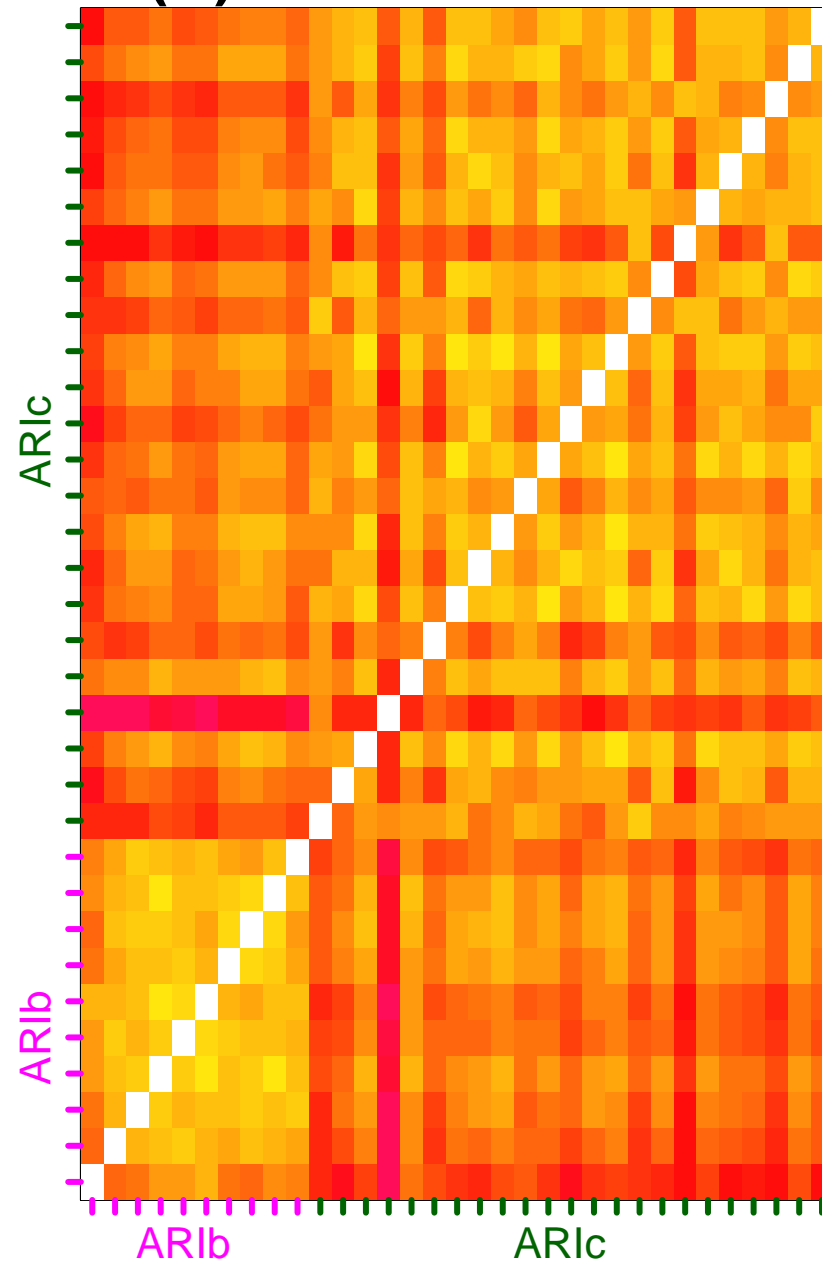

**(C) non-Pagani-donors**

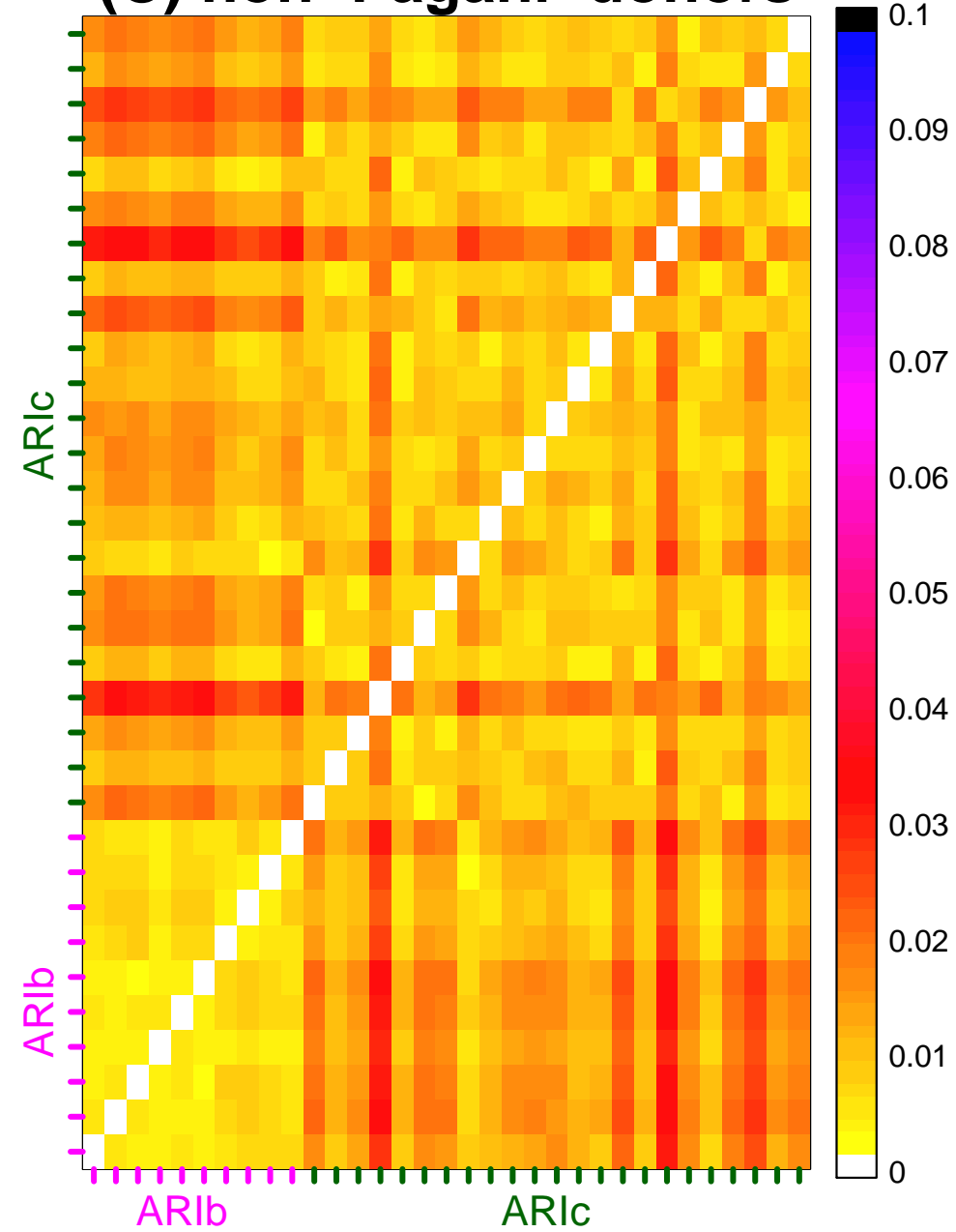

Supplement: S15 Fig — TVD XY between every pair of Ari individuals X, Y, with group labels (ARIb/ARIc) on the axes, under each of analyses (A)-(C). (PDF) [file pgen.1005397.s040.pdf]

**(A) all-donors**

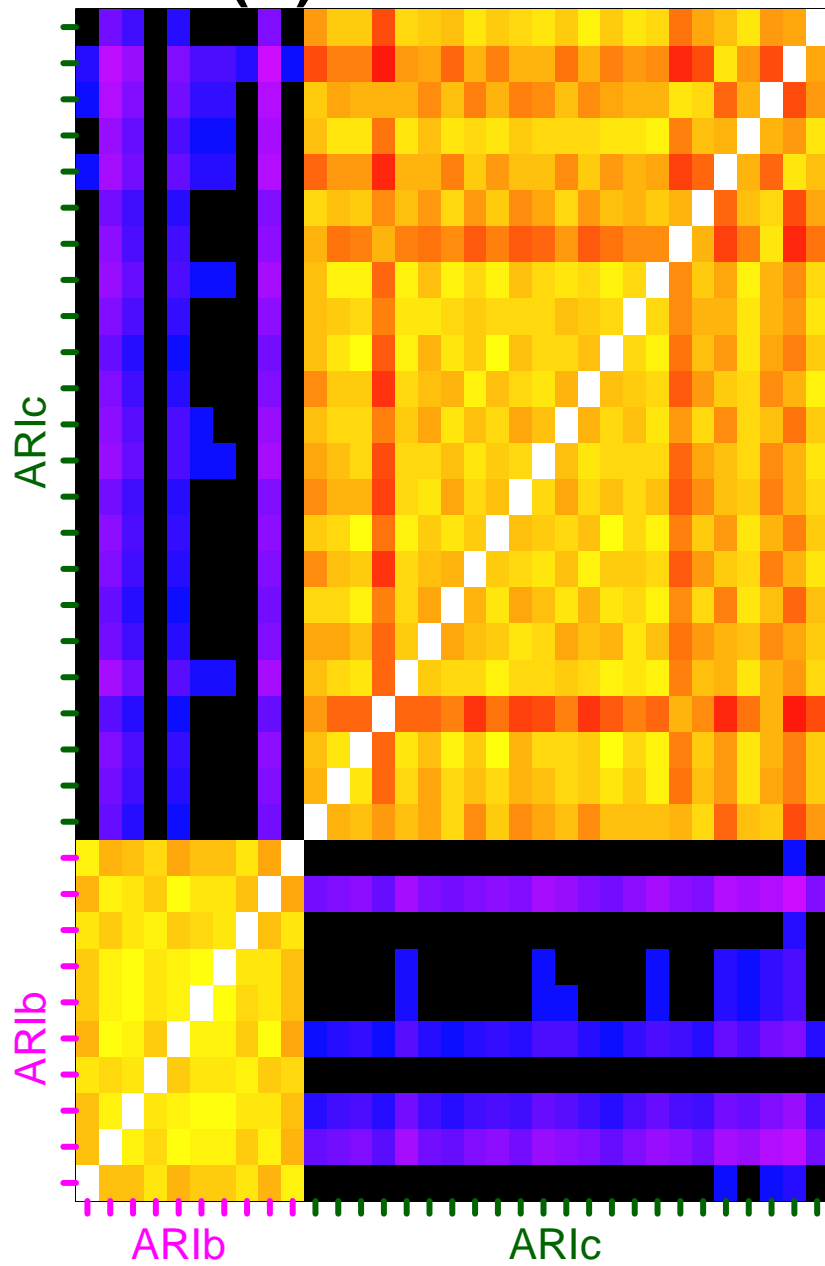

**(B) non-Ari-donors**

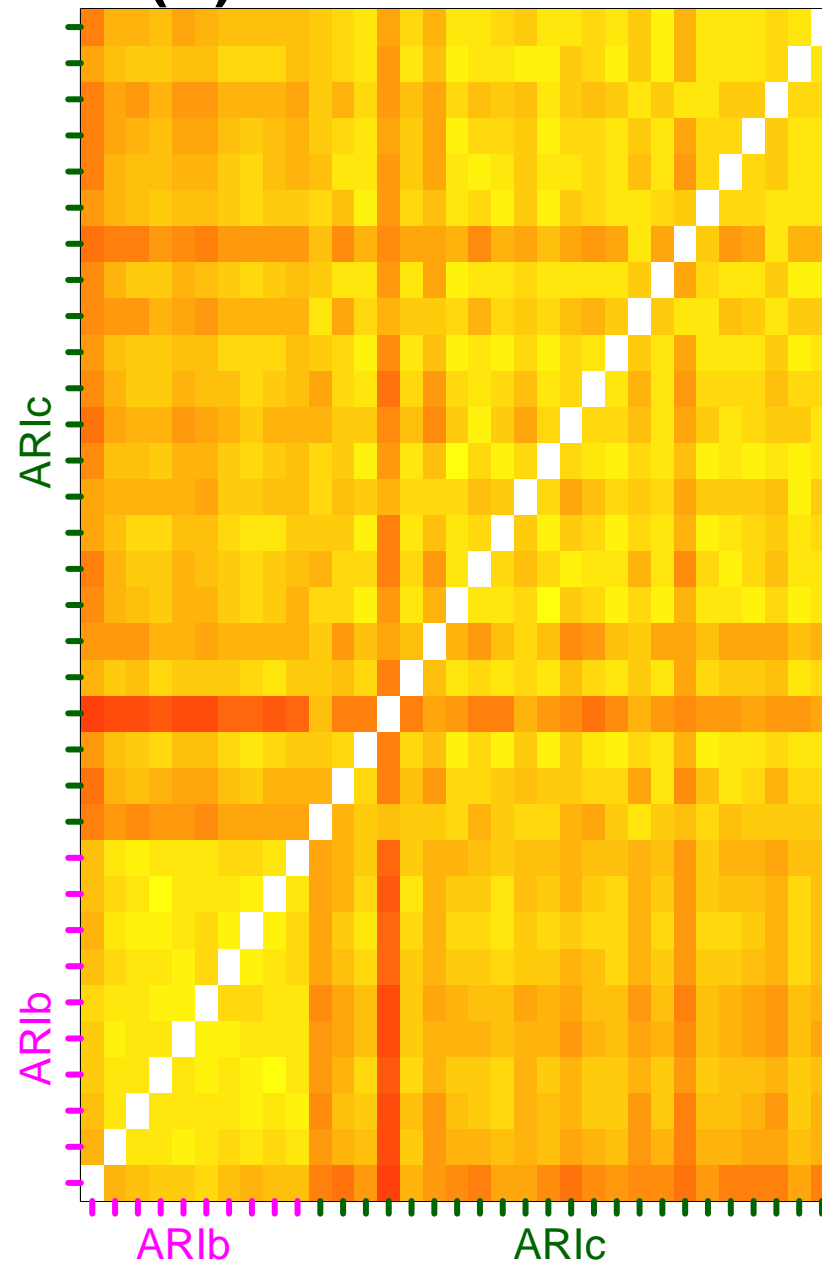

**(C) non-Pagani-donors**

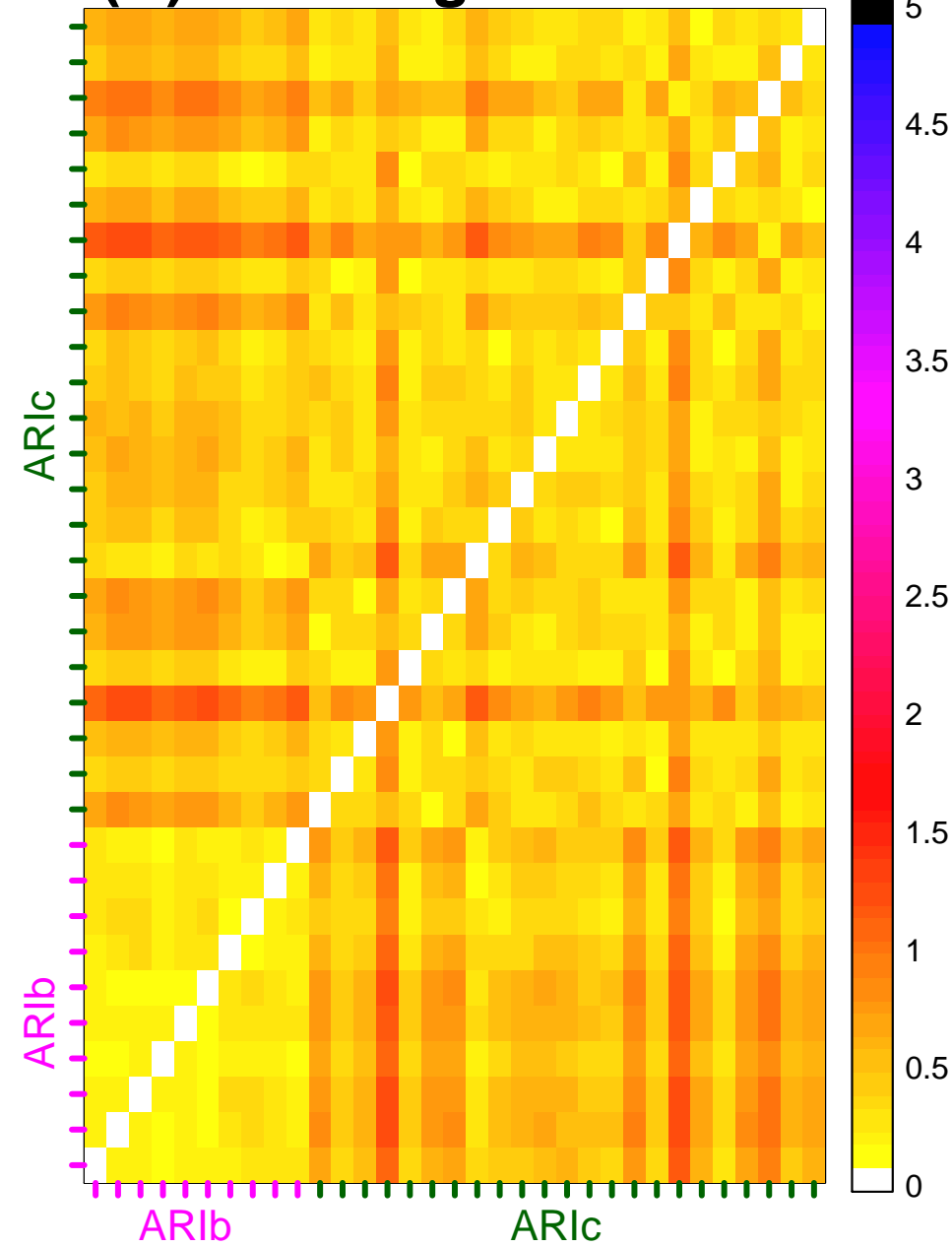

Supplement: S17 Fig — F XY between every pair of Ari individuals X, Y, with group labels (ARIb/ARIc) on the axes, under each of analyses (A)-(C). (PDF) [file pgen.1005397.s042.pdf]

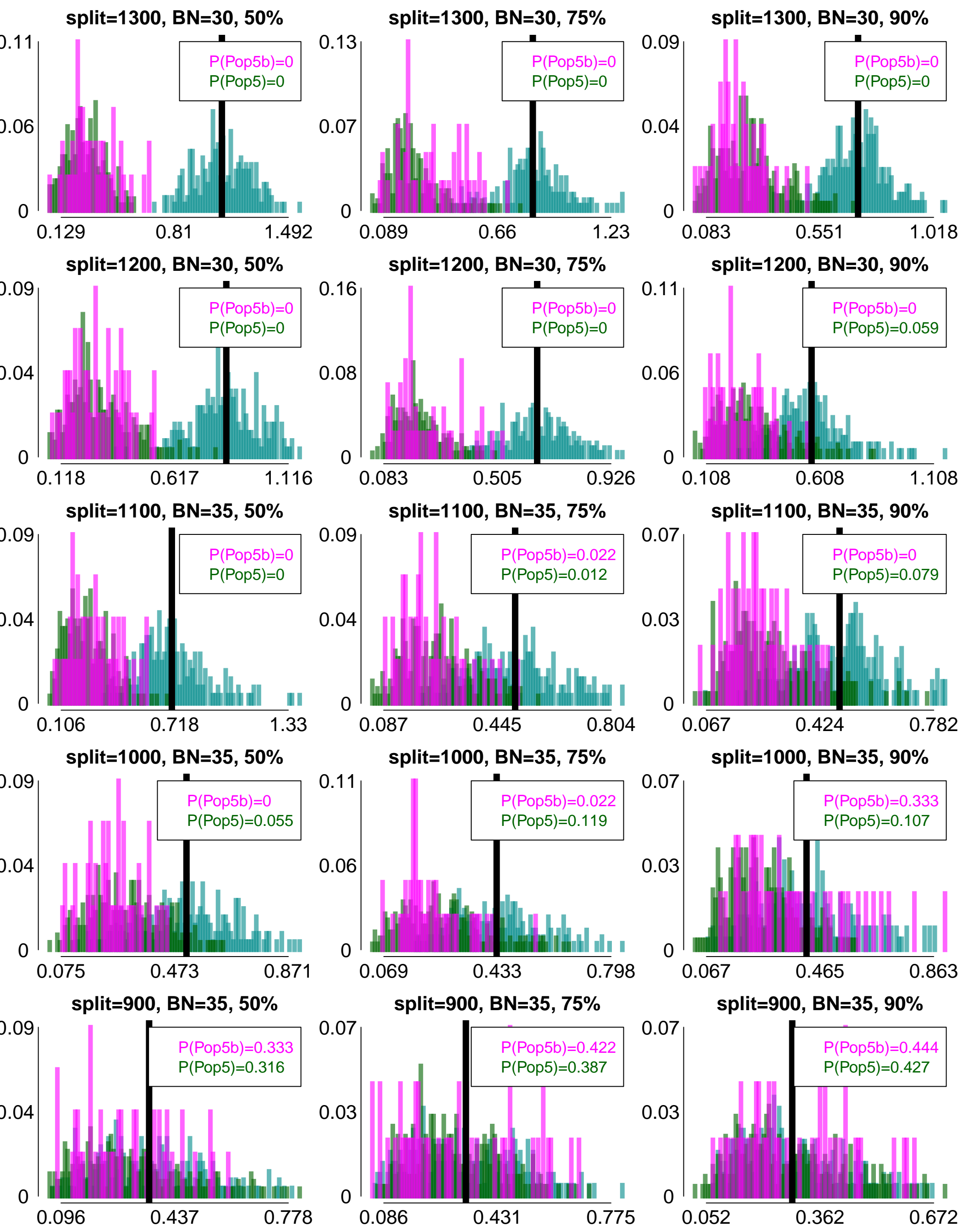

Supplement: S19 Fig — Differences in inferred ancestry under analysis (B) between all pairings of simulated “ARIb” individuals (Pop5b, pink), all pairings of simulated “ARIc” individuals (Pop5, green), and all pairings of one “ARIb” and one “ARIc” individual (cyan), for 15 of the “simplified” simulations. In each plot the black vertical line gives the mean difference across the pairings of one “Pop5b” and one “Pop5”, with P(Pop5b), P(Pop5) giving the proportion of Pop5b and Pop5 pairings, respectively, with a difference greater than or equal to this mean. (PDF) [file pgen.1005397.s044.pdf]

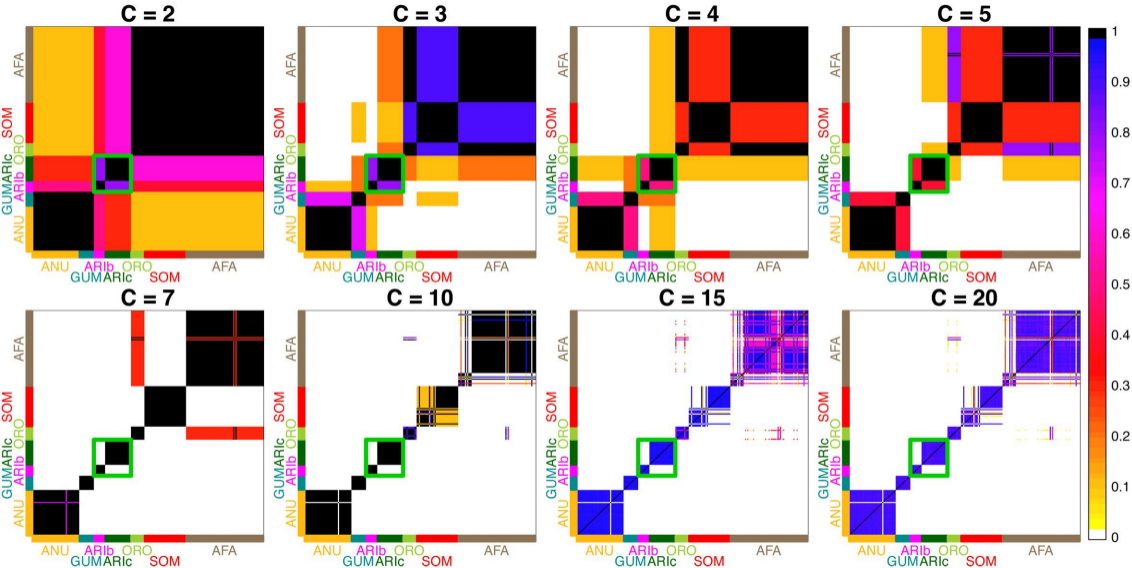

Supplement: S20 Fig — Proportion of MCMC samples for which each pair of Pagani dataset individuals were clustered together when fixing various numbers of clusters C, using inferred ancestries from the “all-donors” CHROMOPAINTER analysis (A). Ari individuals are enclosed in a green border. Results are averaged over ten different runs of the MCMC chain. (PDF) [file pgen.1005397.s045.pdf]

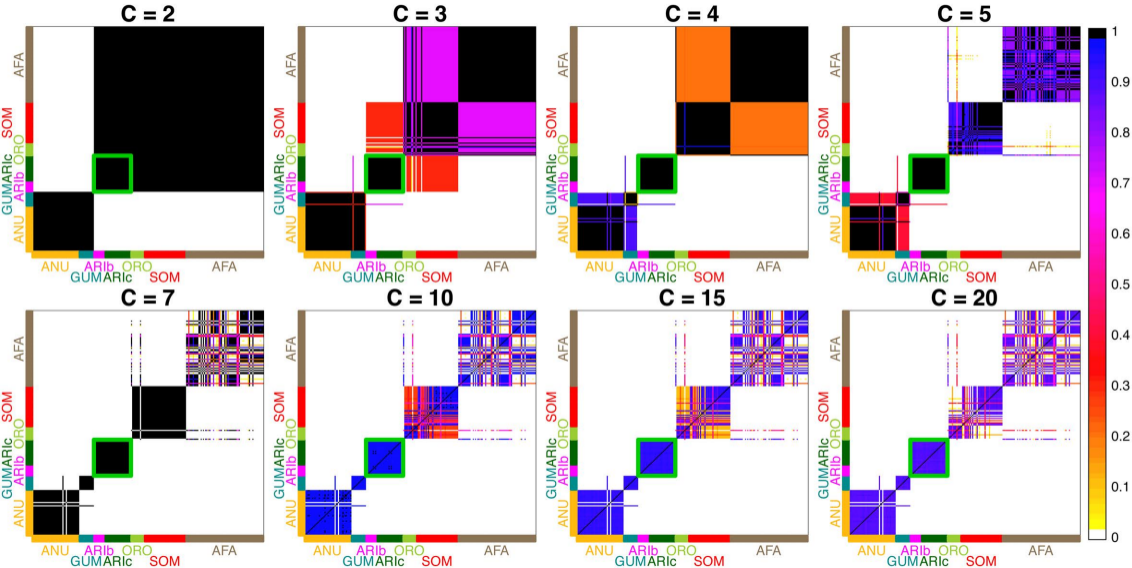

Supplement: S22 Fig — Proportion of MCMC samples for which each pair of Pagani dataset individuals were clustered together when fixing various numbers of clusters C, using inferred ancestries from the “non-Ari-donors” CHROMOPAINTER analysis (B). Ari individuals are enclosed in a green border, and are not separated when C = 50. Results are averaged over ten different runs of the MCMC chain. (PDF) [file pgen.1005397.s046.pdf]

**ARlb: ANU-ARlc**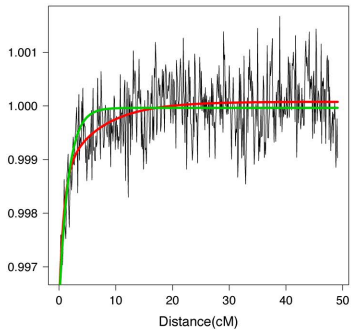**ARlb: TSI-ARlc**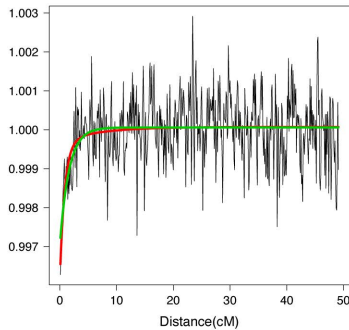**ARlb: TSI-ANU**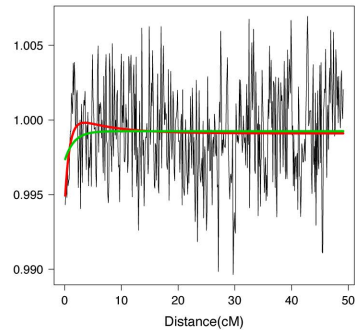**ARlc: ANU-ARlb**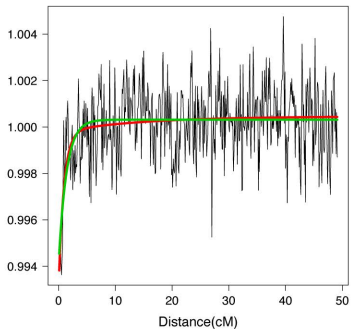**ARlc: TSI-ARlb**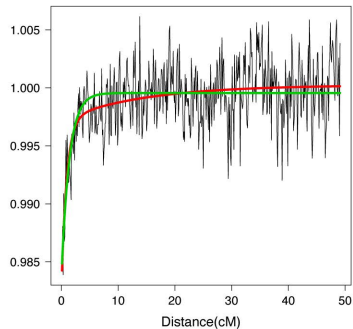**ARlc: TSI-ANU**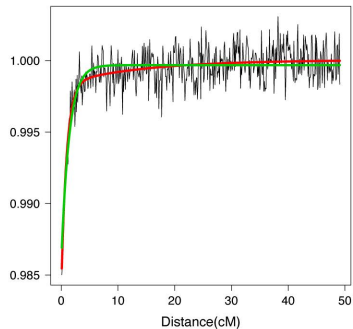

Supplement: S24 Fig — GLOBETROTTER coancestry curves under simplified all-donors analysis (A-sim) for the ARIb (top row) and the ARIc (bottom row) using ANU, TSI, ORO and {ARIc,ARIb} as surrogates. Black lines give the (scaled) probability that two DNA segments within the Ari group are inferred as most ancestrally related to the two donor groups given in the title (y-axis) versus the genetic distance between the two segments’ midpoints (x-axis). Green lines give the best fitting exponential distributions to the black lines assuming a single date of admixture, and red lines give the best fit assuming two distinct dates of admixture. (PDF) [file pgen.1005397.s049.pdf]

**ARib: AFA-AFA**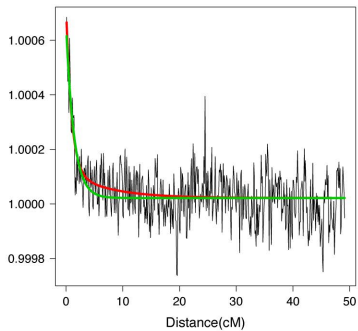**ARib: ANU-AFA**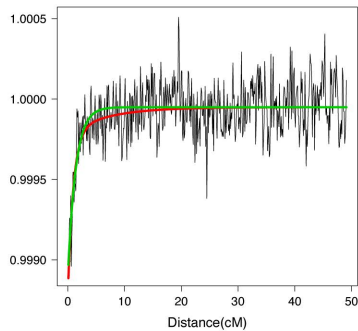**ARib: ANU-ANU**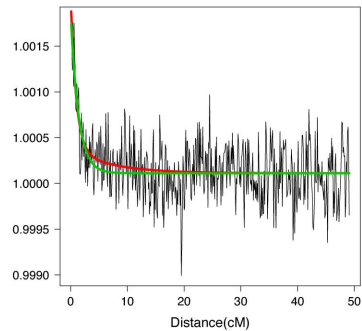**ARic: AFA-AFA**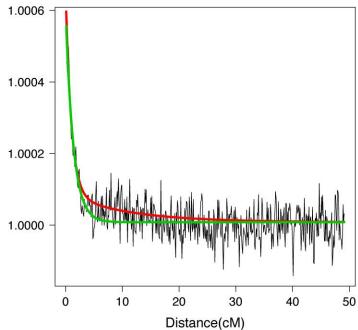**ARic: ANU-AFA**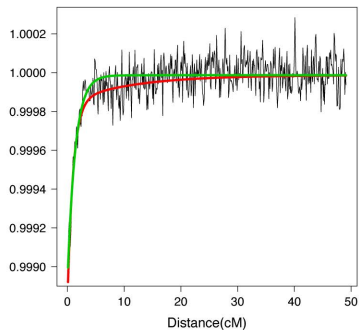**ARic: ANU-ANU**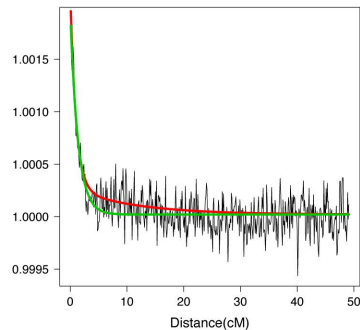

Supplement: S25 Fig — GLOBETROTTER coancestry curves under non-Ari-donors analysis (B) for the ARIb (top row) and the ARIc (bottom row). Black lines give the (scaled) probability that two DNA segments within the Ari group are inferred as most ancestrally related to the two donor groups given in the title (y-axis) versus the genetic distance between the two segments’ midpoints (x-axis). Green lines give the best fitting exponential distributions to the black lines assuming a single date of admixture, and red lines give the best fit assuming two distinct dates of admixture. (PDF) [file pgen.1005397.s050.pdf]

**AR1b: CEU-CEU**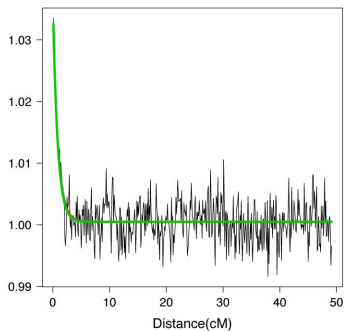**AR1b: CEU-MKK**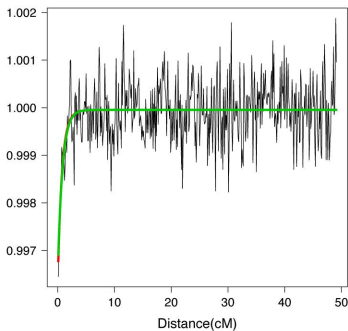**AR1b: MKK-MKK**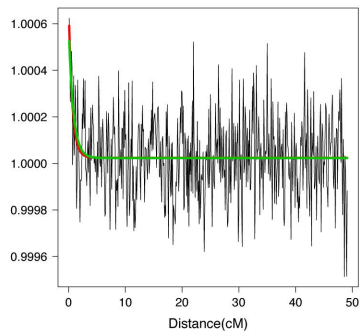**AR1c: CEU-CEU**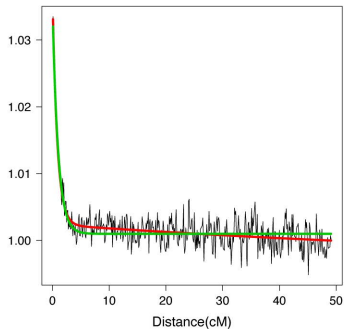**AR1c: CEU-MKK**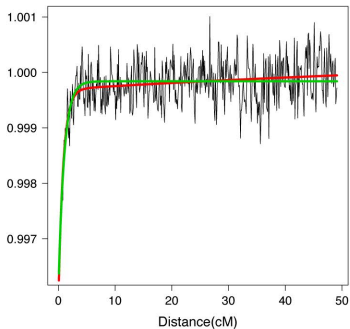**AR1c: MKK-MKK**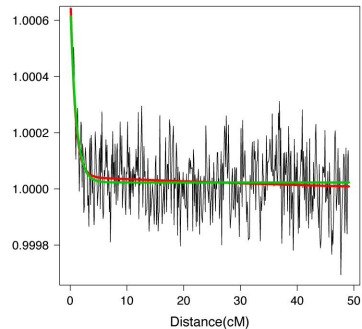

Supplement: S26 Fig — GLOBETROTTER coancestry curves under non-Pagani-donors analysis (C) for the ARIb (top row) and the ARIc (bottom row). Black lines give the (scaled) probability that two DNA segments within the Ari group are inferred as most ancestrally related to the two donor groups given in the title (y-axis) versus the genetic distance between the two segments’ midpoints (x-axis). Green lines give the best fitting exponential distributions to the black lines assuming a single date of admixture, and red lines give the best fit assuming two distinct dates of admixture. (PDF) [file pgen.1005397.s051.pdf]

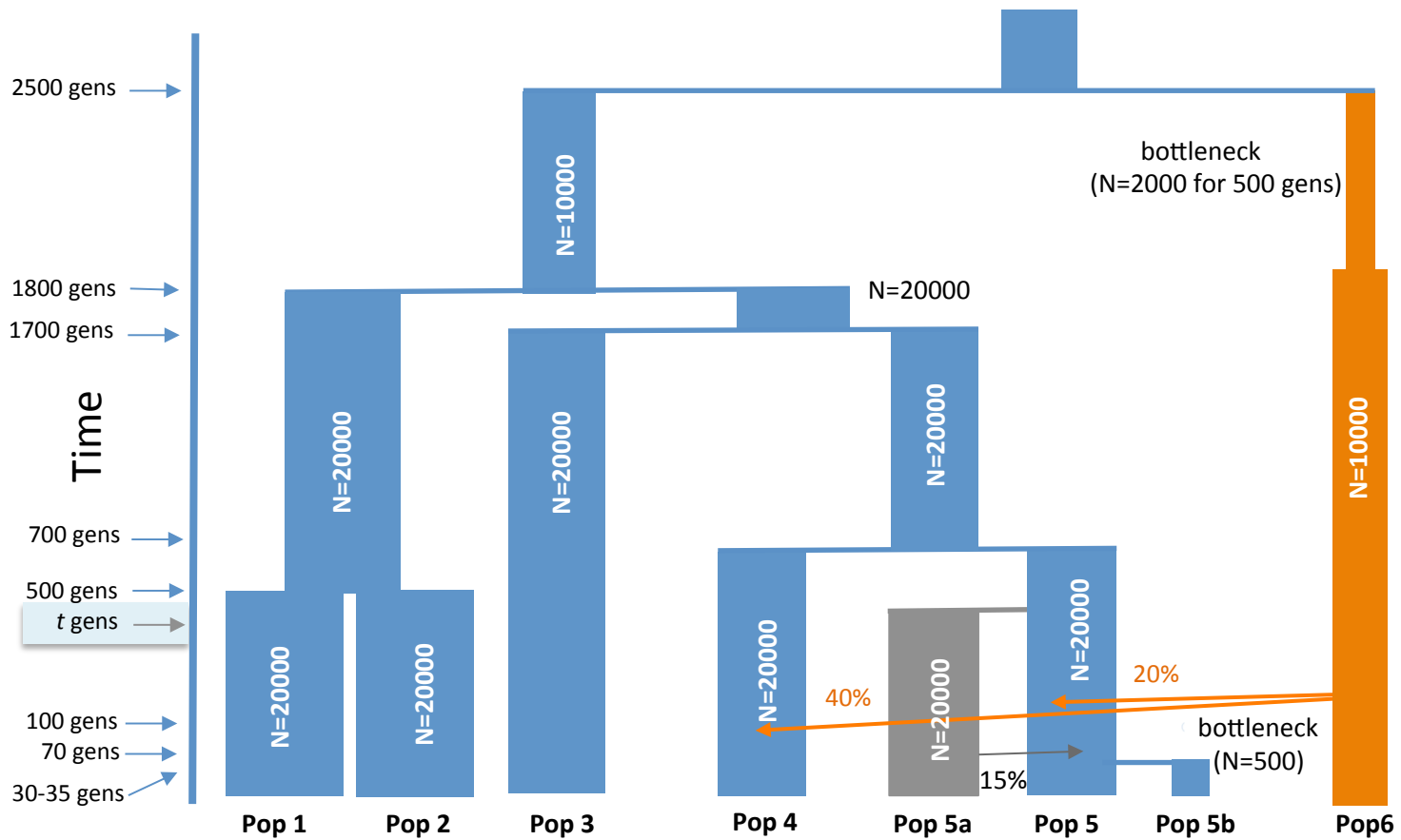

Supplement: S27 Fig — Eight populations simulated under the Marginalisation (MA) model with an additional group Pop5a (shown in grey) providing admixture from an unsampled “Ethiopian” group. Orange arrows indicate migration from Pop6 into Pop4 and Pop5, and the grey arrow indicates migration from Pop5a into Pop5. Pop5 and Pop5a split at varying times t ∈ {300, …, 500}, with a bottleneck in Pop5b occurring {30, 35} generation ago. These parameters were chosen to give F ST values similar to those observed between the ARIb, ARIc and ORO in the real data (see S23 Table). (PDF) [file pgen.1005397.s052.pdf]

**split\*=300, BN=30**

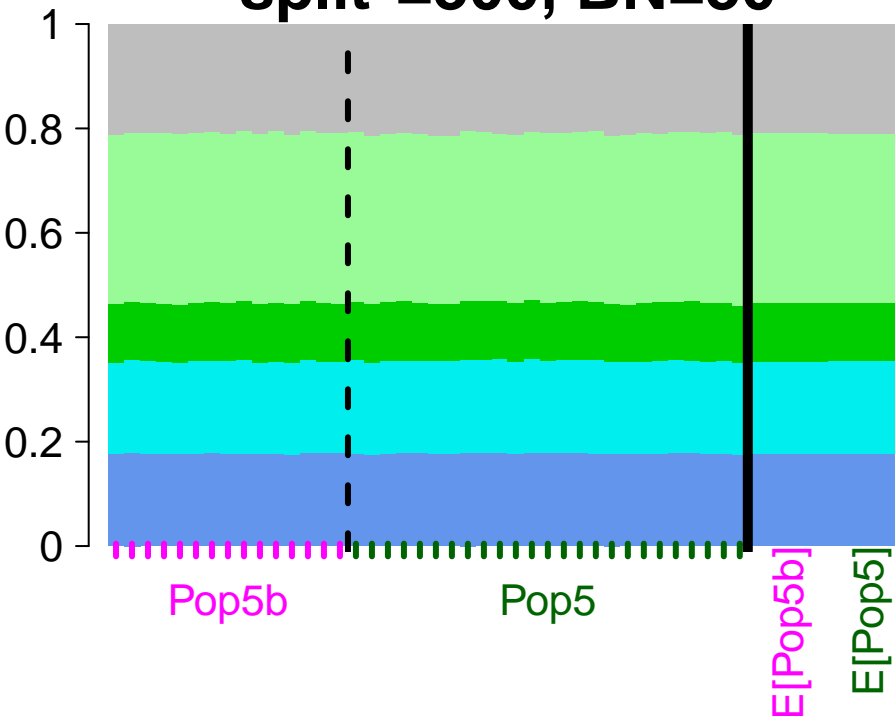

**split\*=400, BN=30**

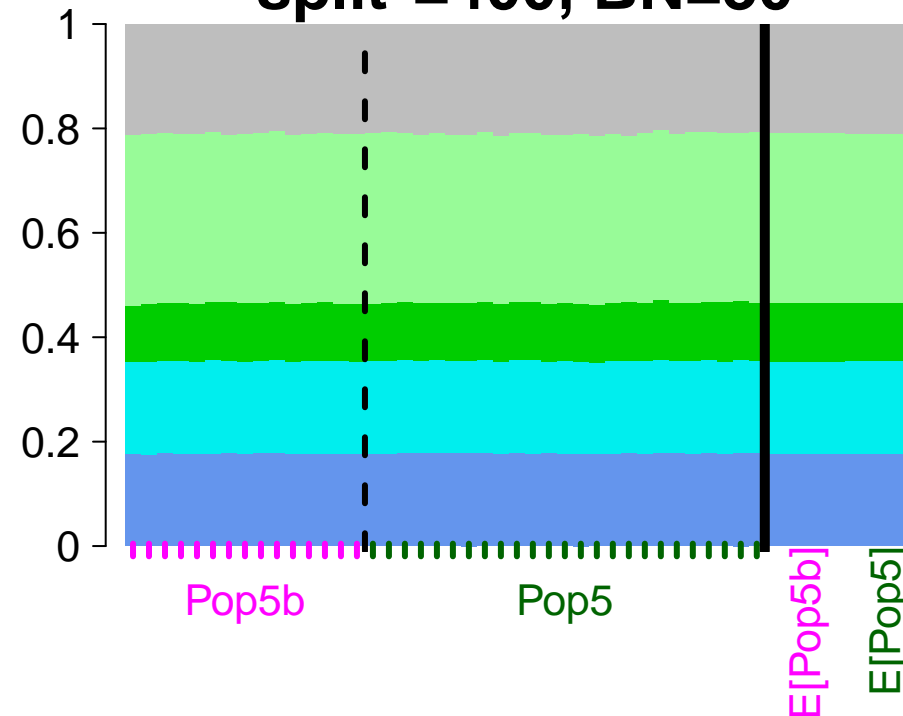

**split\*=500, BN=30**

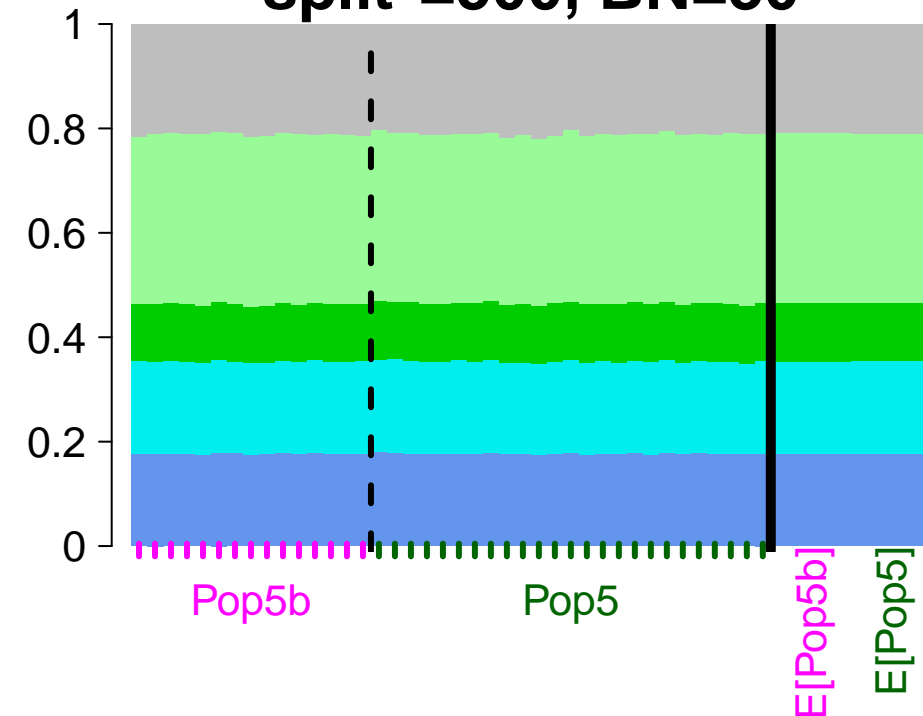

**split\*=300, BN=35**

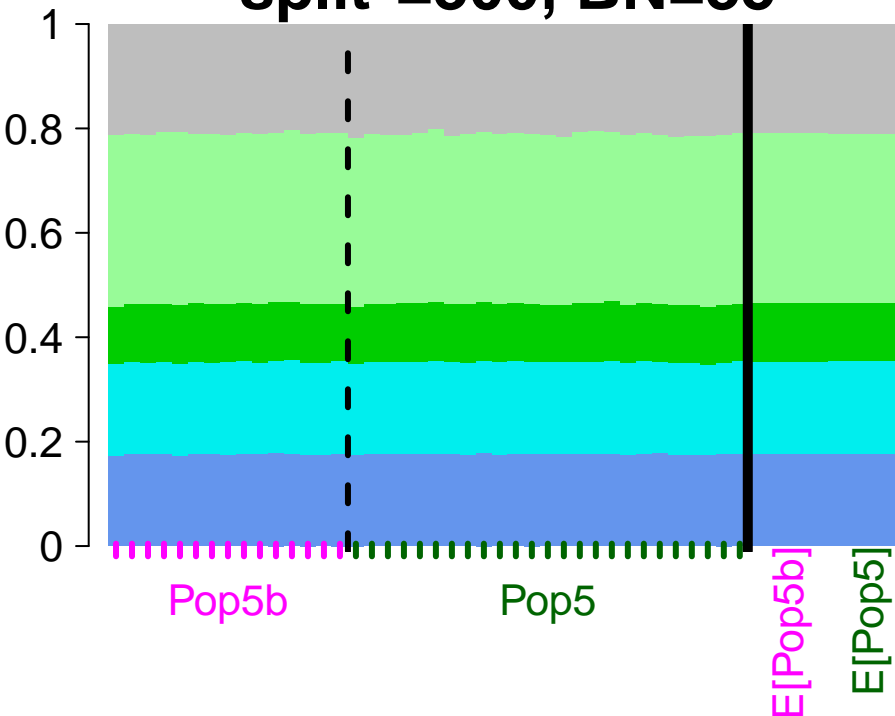

**split\*=400, BN=35**

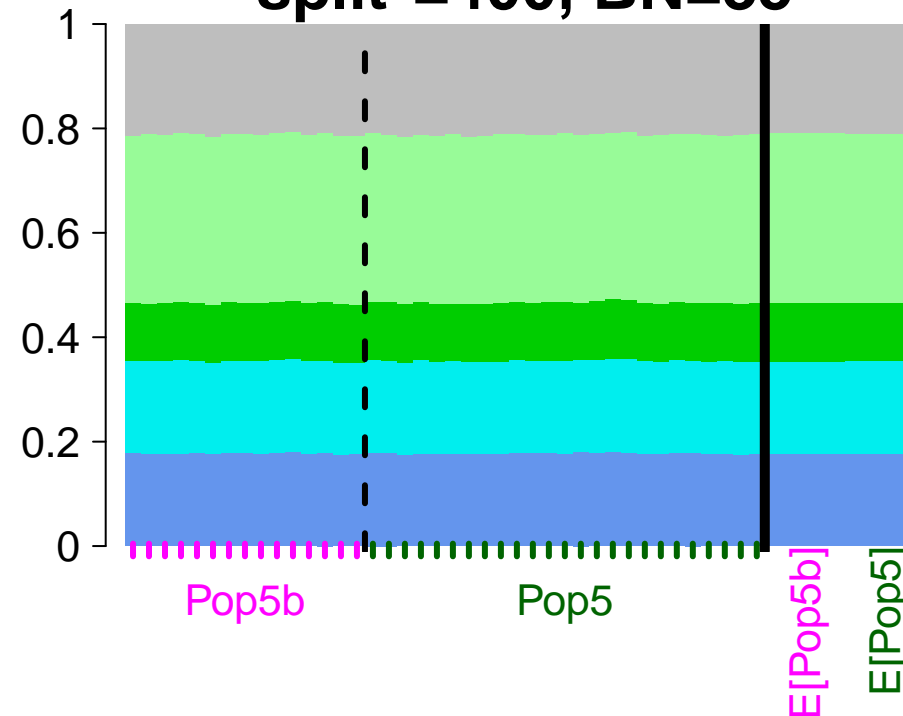

**split\*=500, BN=35**

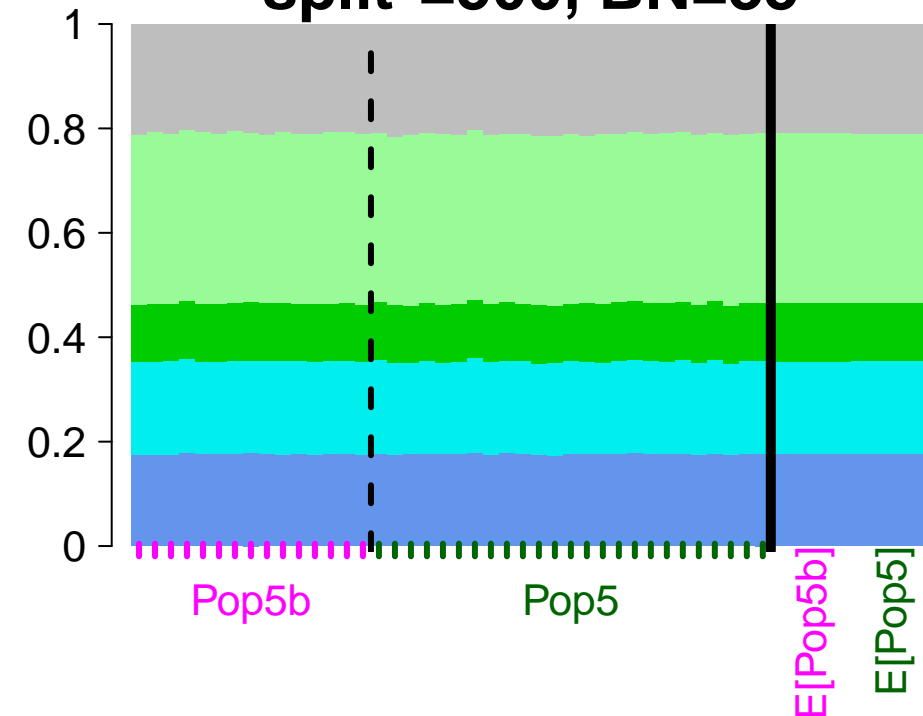

Supplement: S28 Fig — CHROMOPAINTER’s inferred painting profiles for each simulated “Ari” individual in 6 different simulations under the MA model incorporating an unsampled contributing population Pop5a (see Methods), showing the proportion of DNA copied from each simulated donor group (color). Group labels (Pop5b = “ARIb” / Pop5 = “ARIc”) are given on the x-axis, with group means (E[Pop5b],E[Pop5]) at far right. The titles above each plot describe the simulation parameters, which vary in the number of generations ago Pop5a and Pop5 split (“split*”) and the number of generations Pop5b is bottlenecked (“BN”). Results are shown for CHROMOPAINTER analyses using (B) using all non-Pop5 groups only as donors. Each distinct color denotes the inferred painting from a distinct group, with the legend for each non-Pop5 group given in the third bar of the top left plot of S13 Fig. (PDF) [file pgen.1005397.s053.pdf]

**split\*=300, BN=30**

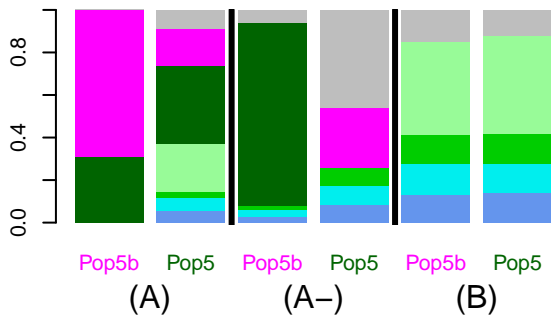

**split\*=400, BN=30**

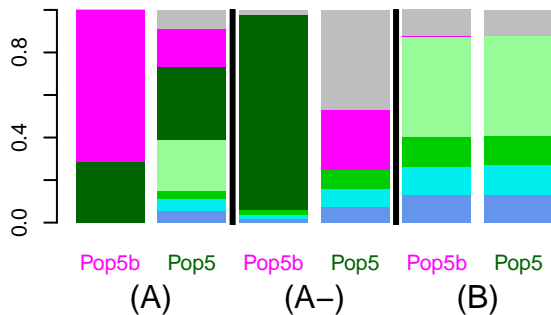

**split\*=500, BN=30**

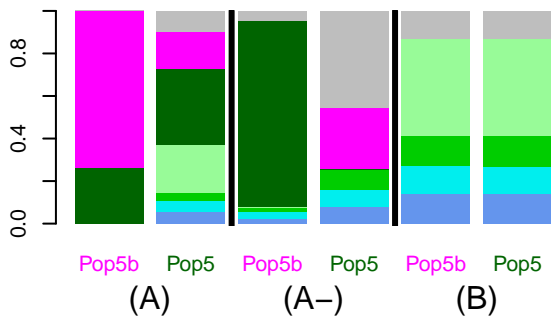

**split\*=300, BN=35**

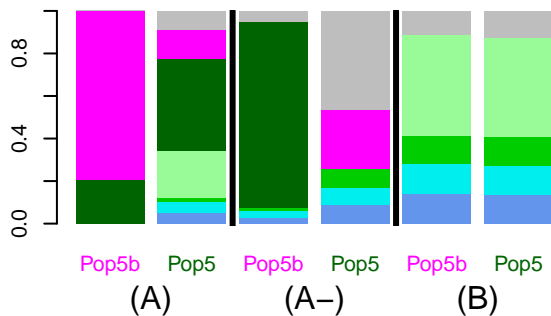

**split\*=400, BN=35**

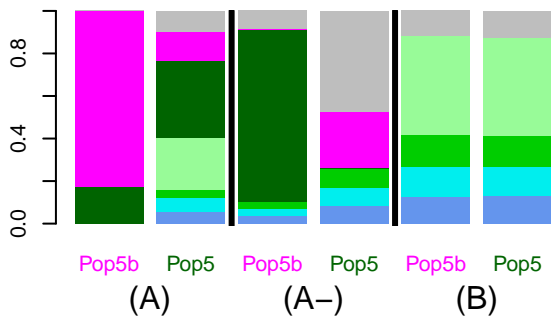

**split\*=500, BN=35**

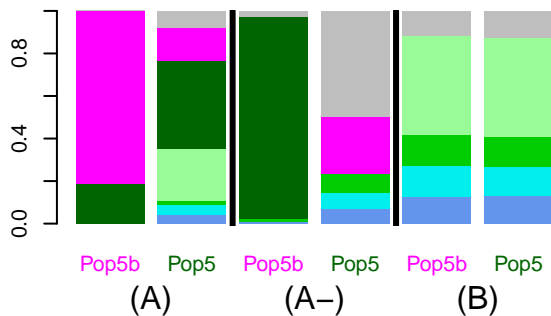

Supplement: S29 Fig — Inferred proportions of ancestry for each simulated “Ari” individual in a simulation scenario under the MA model incorporating an unsampled contributing population 5a (see S27 Fig), as inferred using CHROMOPAINTER and additional linear modelling. The simulations vary in the number of generations Pop5a and Pop5 split (“split*”), and the length of subsequent bottleneck (“BN”) in Pop5b. Results are shown for CHROMOPAINTER analyses using all-donors (A), all-donors after removing self-copying (A-), and using all non-Pop5 groups as donors (B) (see Methods). Each distinct color denotes the inferred painting from a distinct group, with the legend for each non-Pop5 group given in the third bar of the top left plot of S13 Fig. Note that the inferred ancestry proportions under analysis (A-), which is analogous to the proportion and soruce estimation technique used in GLOBETROTTER analysis (A), illustrate that the inferred contribution of Pop5b to Pop5 is much less than the reverse, even though the same group (Pop5a) contributed equally to each. (PDF) [file pgen.1005397.s054.pdf]

split\*=300, BN=30

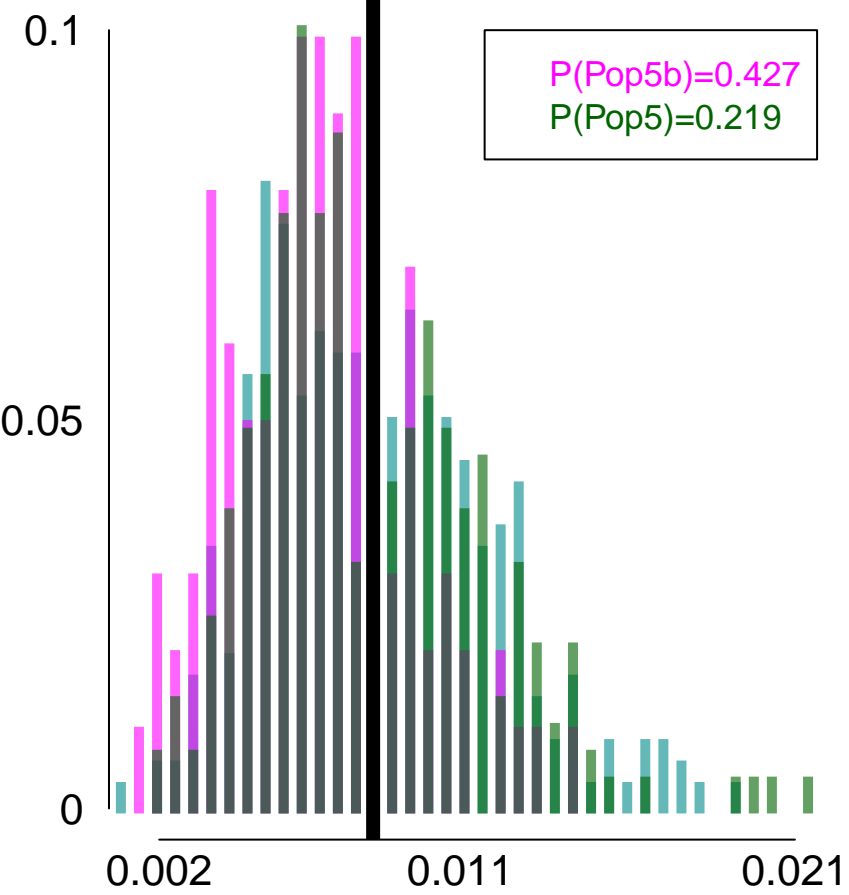

split\*=400, BN=30

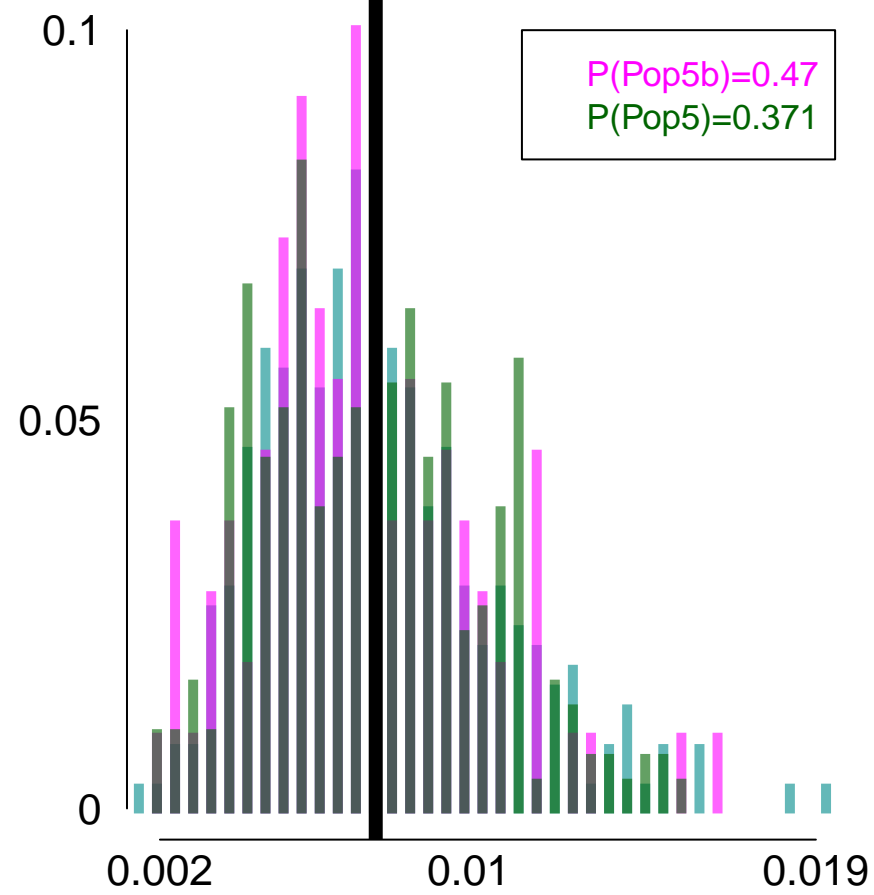

split\*=500, BN=30

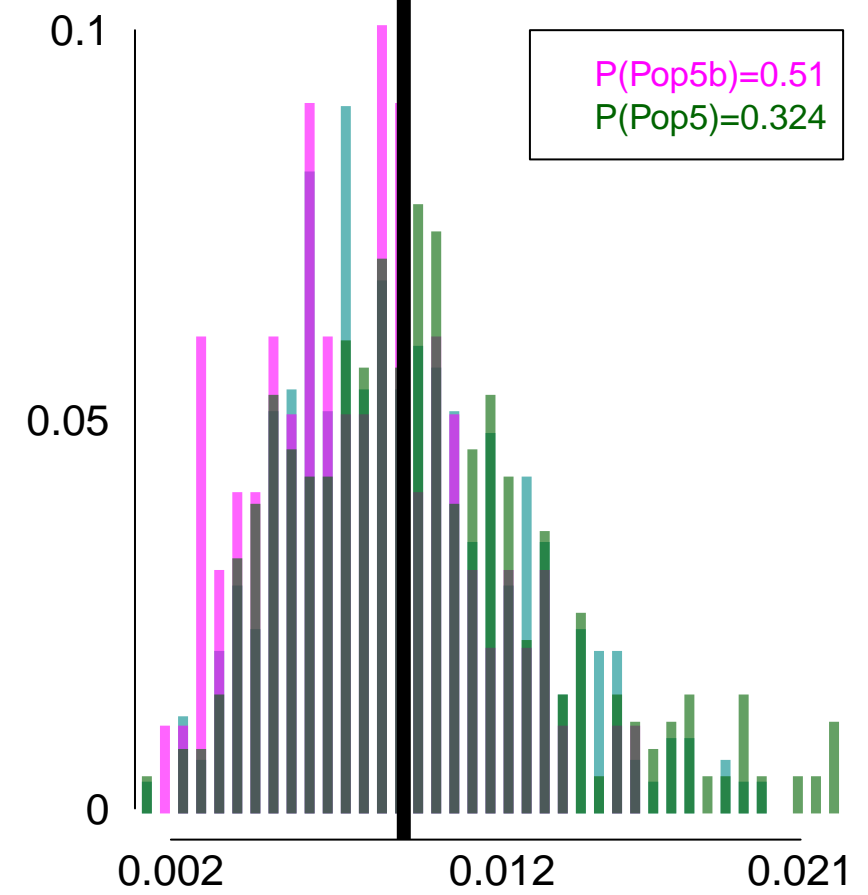

split\*=300, BN=35

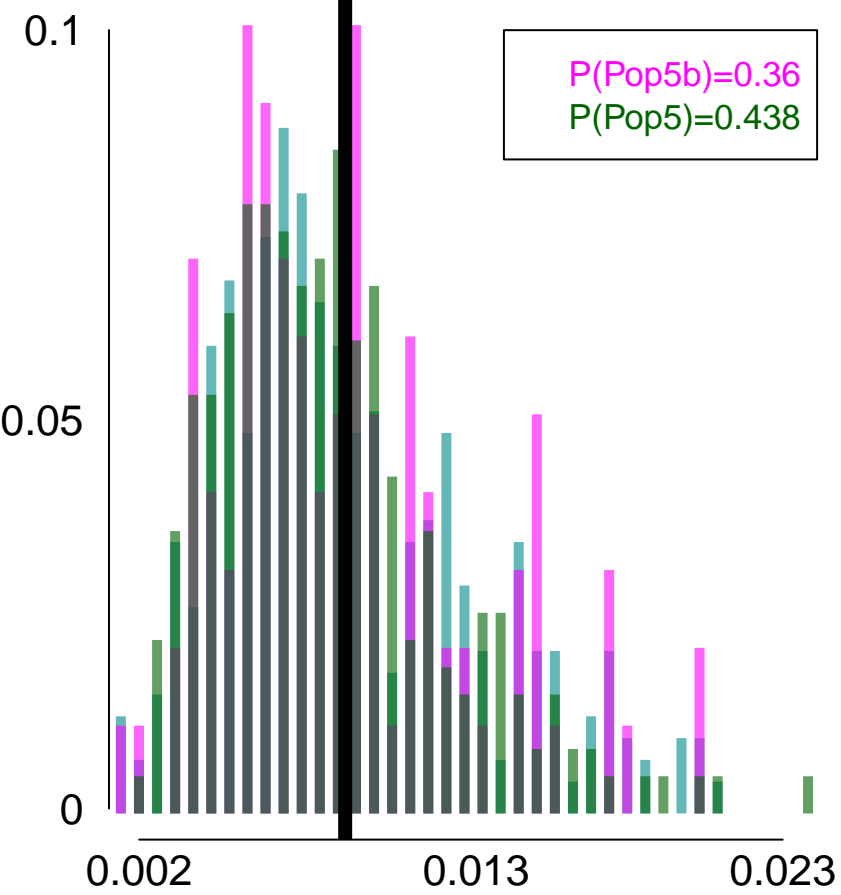

split\*=400, BN=35

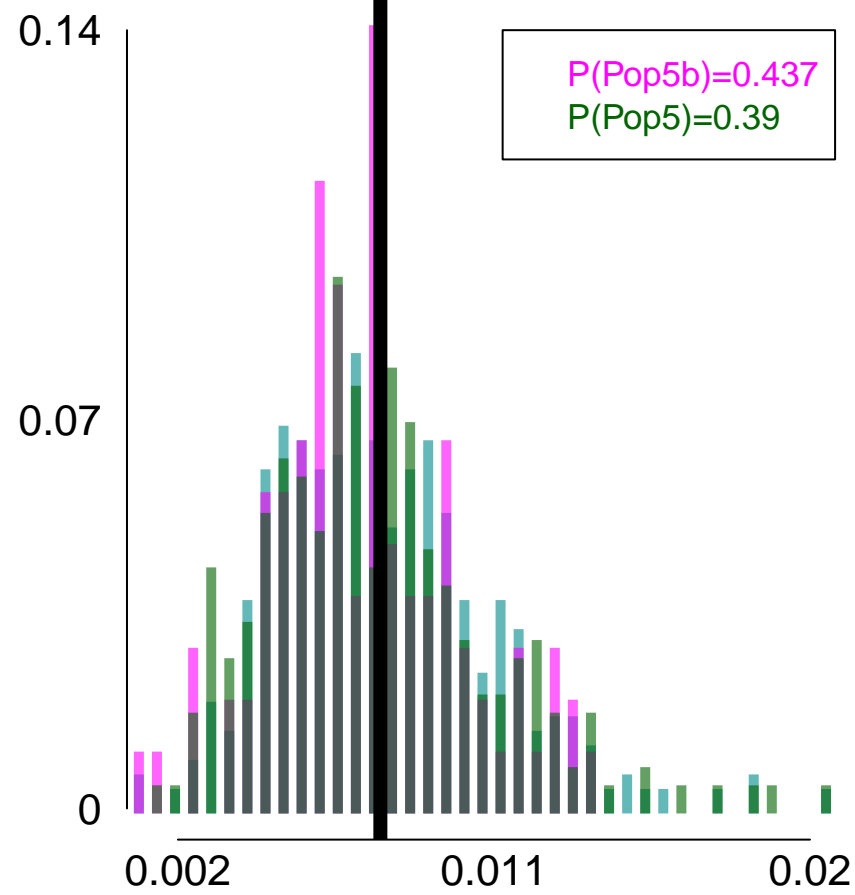

split\*=500, BN=35

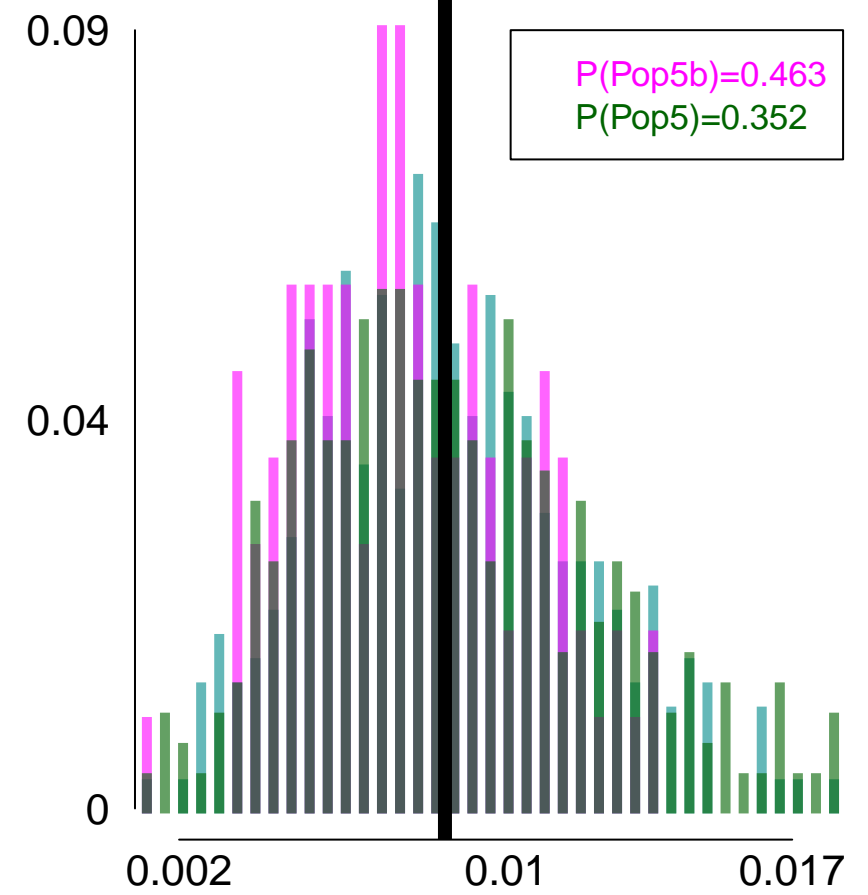

Supplement: S30 Fig — Differences in inferred ancestry under analyses (B) between all pairings of simulated “ARIb” individuals (Pop5b, pink), all pairings of simulated “ARIc” individuals (Pop5, green), and all pairings of one “ARIb” and one “ARIc” individual (cyan), for simulations under an MA model that incorporate an unsampled contributing population 5a (see S27 Fig). In each plot the black vertical line gives the mean difference across the pairings of one “Pop5b” and one “Pop5”, with P(Pop5b), P(Pop5) giving the proportion of Pop5b and Pop5 pairings, respectively, with a difference greater than or equal to this mean. The simulations vary in the number of generations Pop5a and Pop5 split (“split*”) and the number of generations Pop5b is bottlenecked (“BN”). (PDF) [file pgen.1005397.s055.pdf]

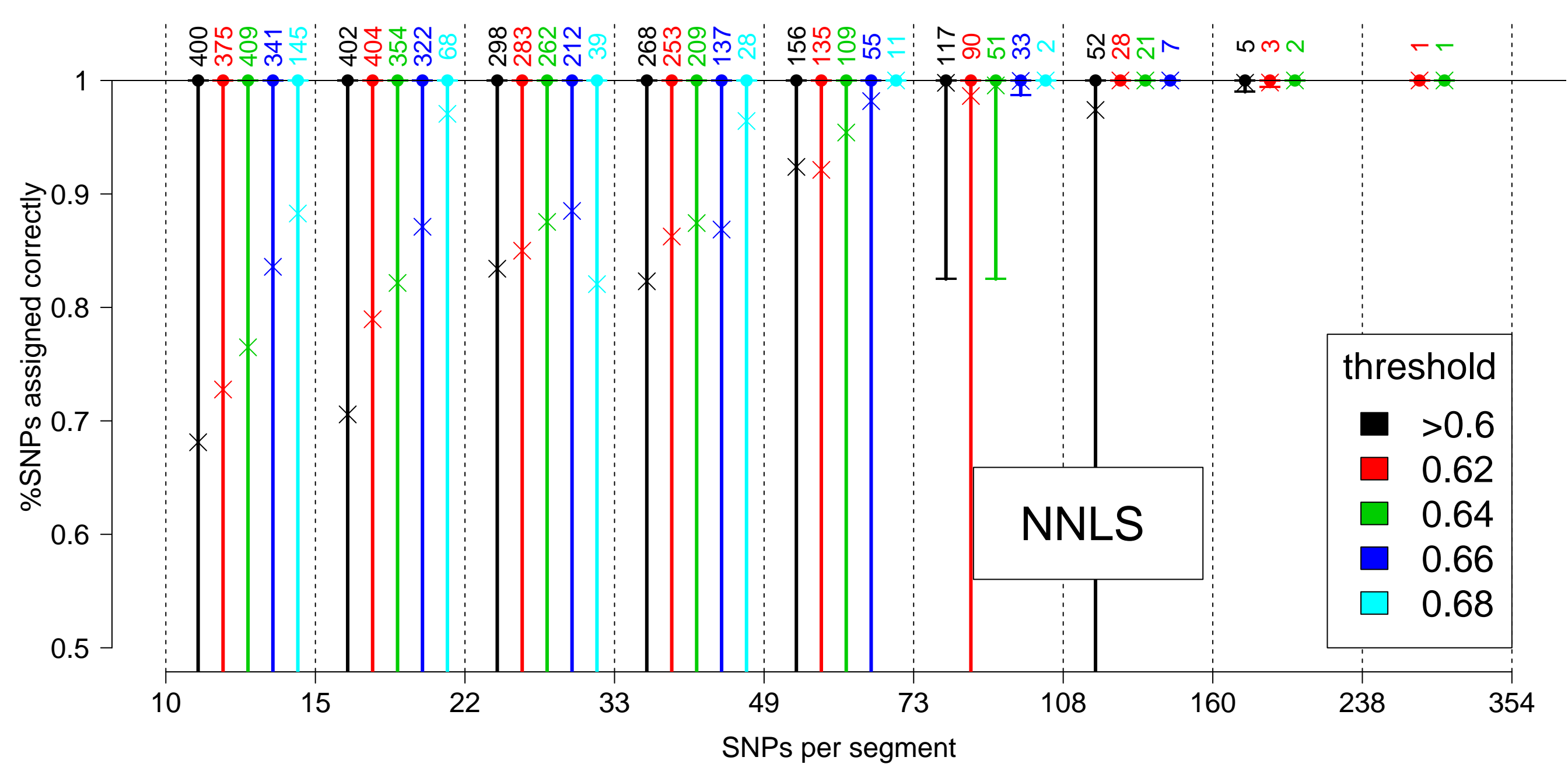

Supplement: S31 Fig — The proportion of SNPs whose true simulated local ancestry is Saudi (y-axis) among segments that have X contiguous SNPs (x-axis) all inferred as CEU under the CHROMOPAINTER “NNLS” approach. Each color represents a different threshold for confidently calling a SNP as CEU (see legend), which is meant to act as a surrogate for Saudi. Vertical solid lines show the range, dots the median, and crosses the mean of proportions across all segments whose number of contiguous CEU-inferred SNPs falls between a given pair of consecutive dashed lines (these SNP number bins are on a log10 scale). The number of segments falling within each SNP number bin are given at top for each threshold value; only chromosome 2 was simulated. (PDF) [file pgen.1005397.s056.pdf]

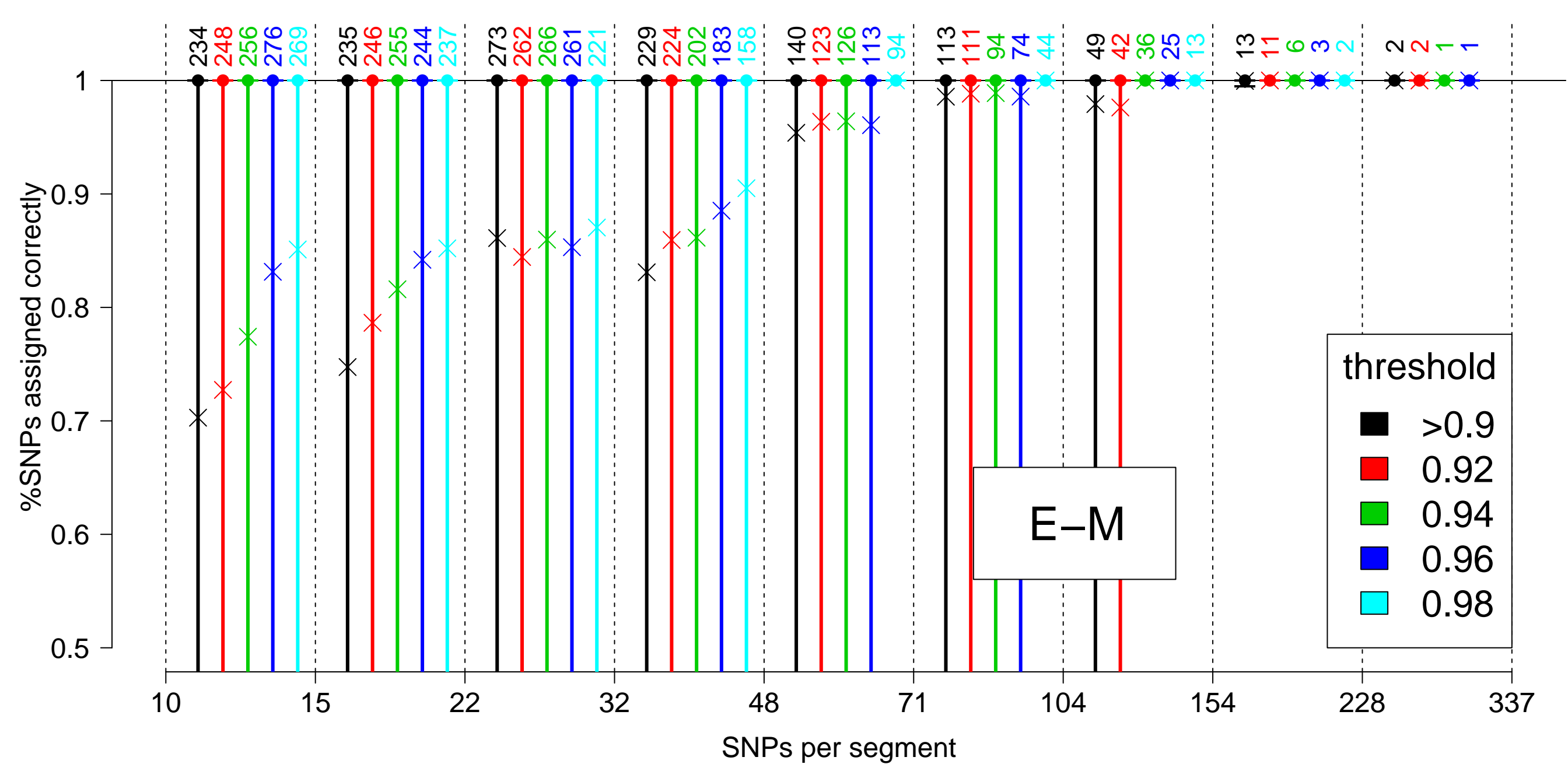

Supplement: S32 Fig — The proportion of SNPs whose true simulated local ancestry is Saudi (y-axis) among segments that have X contiguous SNPs (x-axis) all inferred as CEU under the CHROMOPAINTER “E-M” approach. Each color represents a different threshold for confidently calling a SNP as CEU (see legend), which is meant to act as a surrogate for Saudi. Vertical solid lines show the range, dots the median, and crosses the mean of proportions across all segments whose number of contiguous CEU-inferred SNPs falls between a given pair of consecutive dashed lines (these SNP number bins are on a log10 scale). The number of segments falling within each SNP number bin are given at top for each threshold value; only chromosome 2 was simulated. (PDF) [file pgen.1005397.s057.pdf]

# empirical distribution of $(\phi_I^k / \phi_A^k)$ (NNLS)

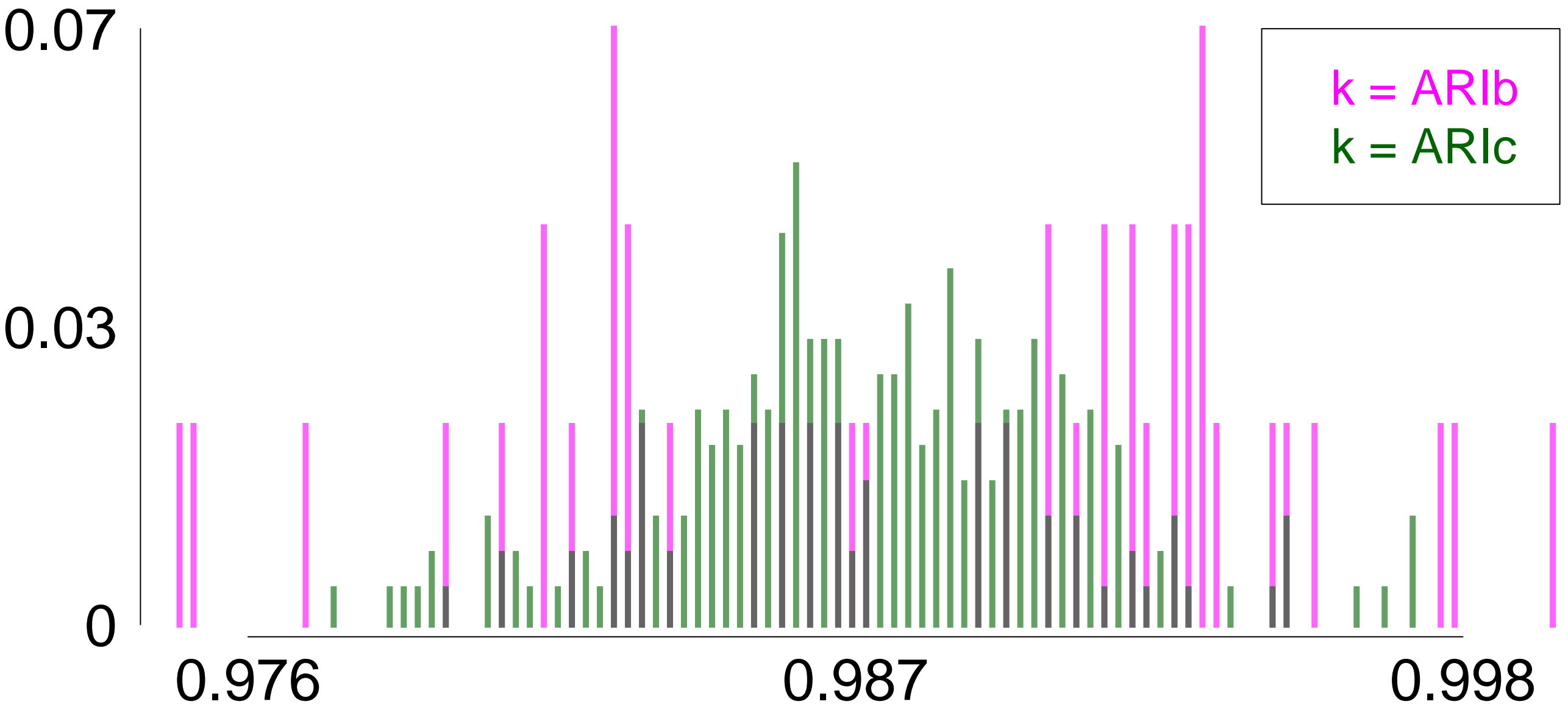

Supplement: S34 Fig — The distributions of (ϕIARIb/ϕAARIb) and (ϕIARIc/ϕAARIc) (see text and Fig 6a) across all pairwise comparisons of individuals within each Ari group, for segments inferred as from the introgressing (I) or ancestral (A) sources using the NNLS model with a threshold of 0.66 and assuming I is the “West Eurasian” source (see Methods). As in the corresponding Fig 6b that instead used the E-M model to infer segments’ sources, the strong similarity in distributions is consistent with the introgression occurring before the split. (PDF) [file pgen.1005397.s059.pdf]
