# Supplementary material for: Evidence for a Common Origin of Blacksmiths and Cultivators in the Ethiopian Ari within the Last 4500 Years: Lessons for Clustering-Based Inference
Source: PLoS Genet. 2015 Aug 20;11(8):e1005397. doi: 10.1371/journal.pgen.1005397 (PMC4546361; doi:10.1371/journal.pgen.1005397)
Supplement: S16 Fig — TVD XY between every pair of individuals X, Y simulated under the Marginalisation (MA; top row) and Remnants (RN; rows 2–4) models in the “full” simulations, with group labels (Pop5/Pop5b) on the axes, under each of analyses (A)-(C). (PDF) [file pgen.1005397.s041.pdf]

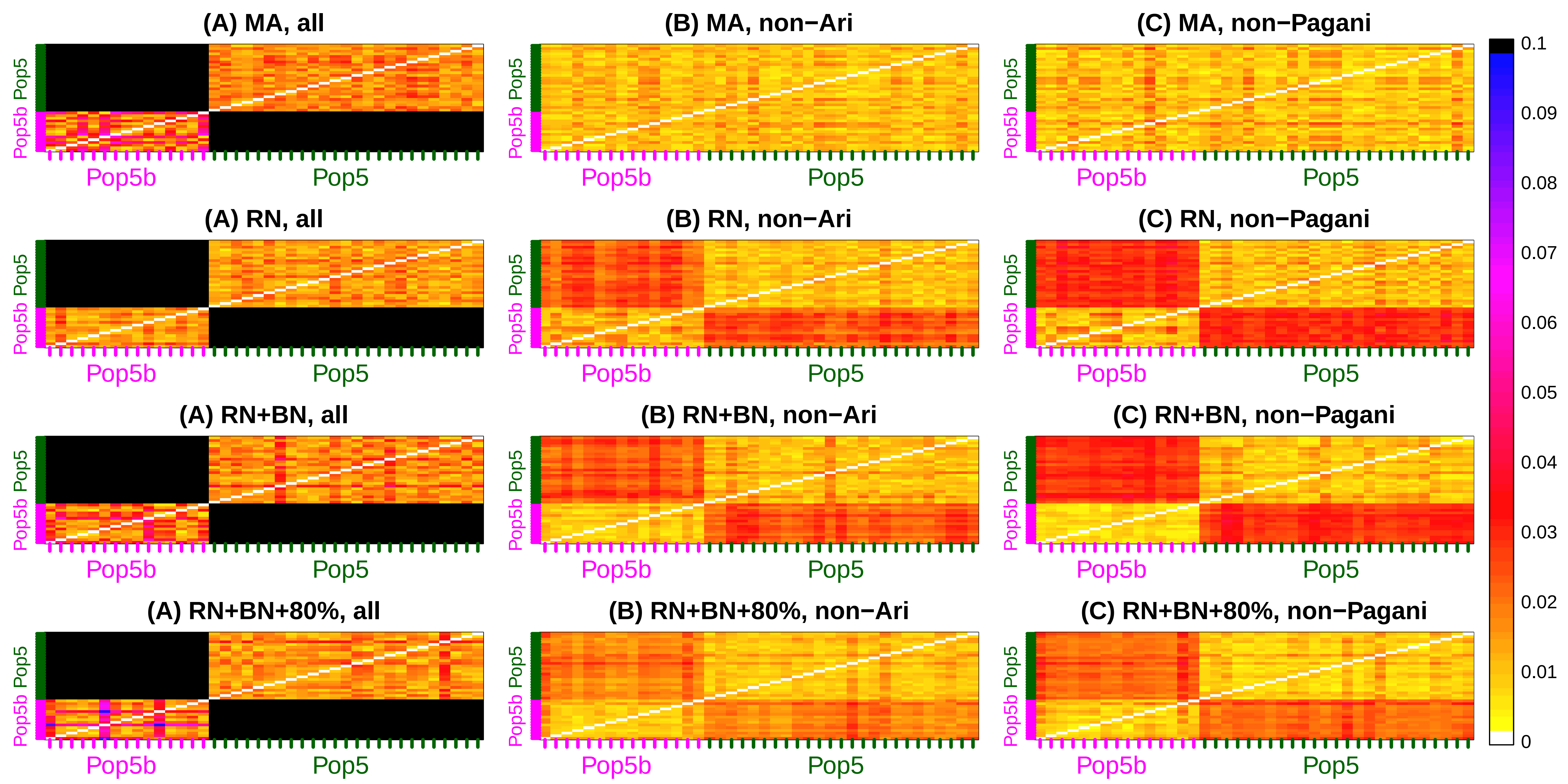
(A) MA, all
(B) MA, non−Ari
(C) MA, non−Pagani
(A) RN, all
(B) RN, non−Ari
(C) RN, non−Pagani
(A) RN+BN, all
(B) RN+BN, non−Ari
(C) RN+BN, non−Pagani
(A) RN+BN+80%, all
(B) RN+BN+80%, non−Ari
(C) RN+BN+80%, non−Pagani
Pop5
Pop5b
0.1
0.09
0.08
0.07
0.06
0.05
0.04
0.03
0.02
0.01
0
