# Supplementary material for: Evidence for a Common Origin of Blacksmiths and Cultivators in the Ethiopian Ari within the Last 4500 Years: Lessons for Clustering-Based Inference
Source: PLoS Genet. 2015 Aug 20;11(8):e1005397. doi: 10.1371/journal.pgen.1005397 (PMC4546361; doi:10.1371/journal.pgen.1005397)
Supplement: S23 Fig — GLOBETROTTER coancestry curves under all-donors analysis (A) for the ARIb (top row) and the ARIc (rows 2–3). Black lines give the (scaled) probability that two DNA segments within the Ari group are inferred as most ancestrally related to the two donor groups given in the title (y-axis) versus the genetic distance between the two segments’ midpoints (x-axis). Green lines give the best fitting exponential distributions to the black lines assuming a single date of admixture, and red lines give the best fit assuming two distinct dates of admixture. Note in the ARIc that the coancestry curves for each pairwise combination of {ARIb,ANU,ORO} increase with increasing genetic distance, suggesting clear evidence of admixture from three distinct source groups around the same time. (PDF) [file pgen.1005397.s048.pdf]

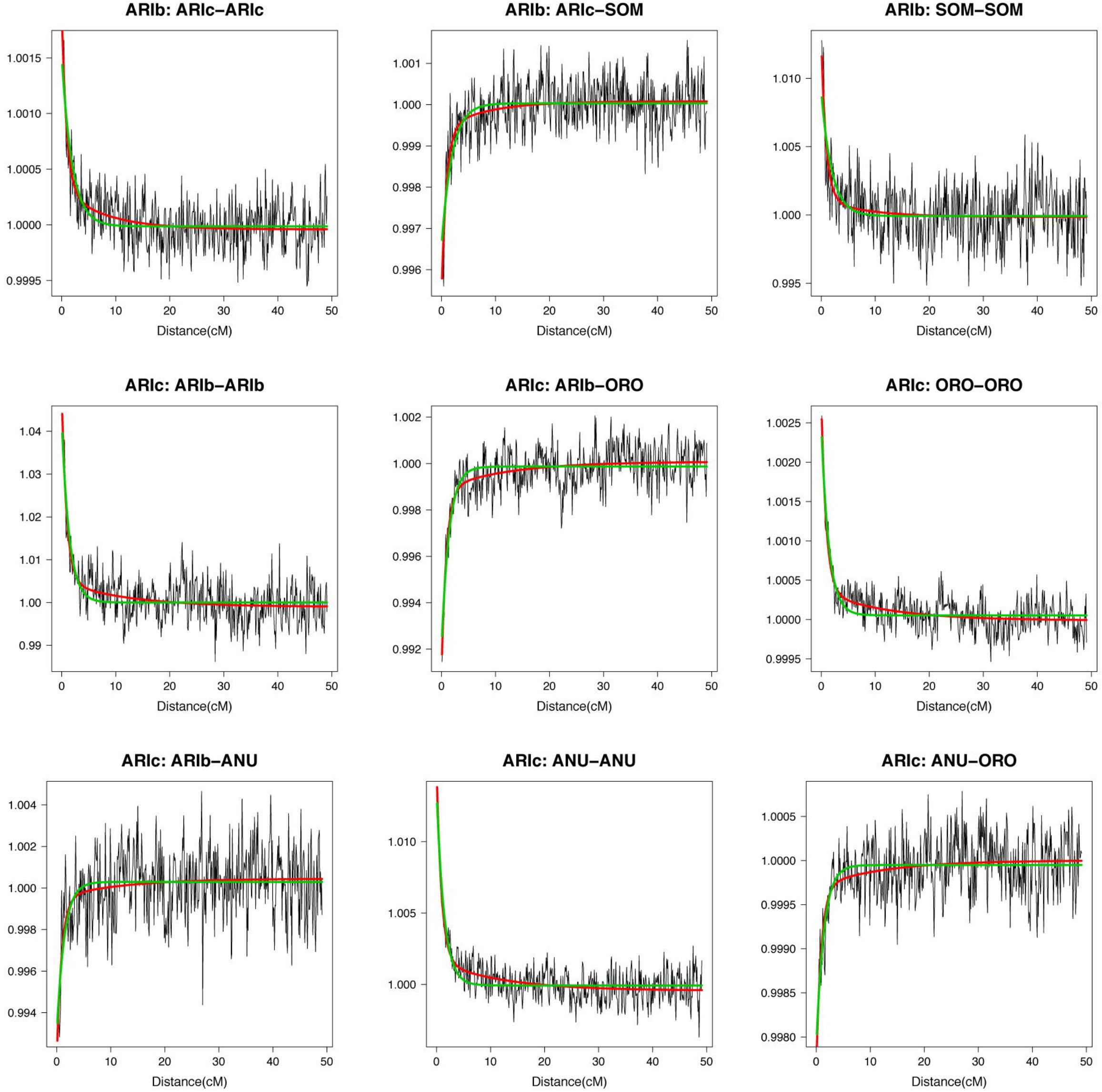
ARIb: ARIc–ARIc
ARIb: ARIc–SOM
ARIb: SOM–SOM
ARIc: ARIb–ARIb
ARIc: ARIb–ORO
ARIc: ORO–ORO
ARIc: ARIb–ANU
ARIc: ANU–ANU
ARIc: ANU–ORO
Distance(cM)
0
10
20
30
40
50
