# Supplementary material for: Evidence for a Common Origin of Blacksmiths and Cultivators in the Ethiopian Ari within the Last 4500 Years: Lessons for Clustering-Based Inference
Source: PLoS Genet. 2015 Aug 20;11(8):e1005397. doi: 10.1371/journal.pgen.1005397 (PMC4546361; doi:10.1371/journal.pgen.1005397)
Supplement: S33 Fig — The distributions of genetic similarity scores, separately within segments matched to CEU (top row) and YRI (bottom row), across all pairwise comparisons of individuals within the ARIb (pink), within the ARIc (green), and with one ARIb and one ARIc individual (cyan). Results are shown for segments assigned to CEU and YRI using (left) the E-M model with a threshold of 0.94, and (right) the NNLS model with a threshold of 0.66. The solid black vertical line gives the average across all pairwise combinations of one ARIb and one ARIc individual, with dotted black lines giving the 95% empirical quantile. Note that the ARIb have higher similarity scores within each of the CEU and YRI segments, as expected from bottleneck effects. In addition, differences between ARIb and ARIc pairs are very similar to those among ARIc pairs, consistent with identical non-African and African ancestral sources in each Ari group. (PDF) [file pgen.1005397.s058.pdf]

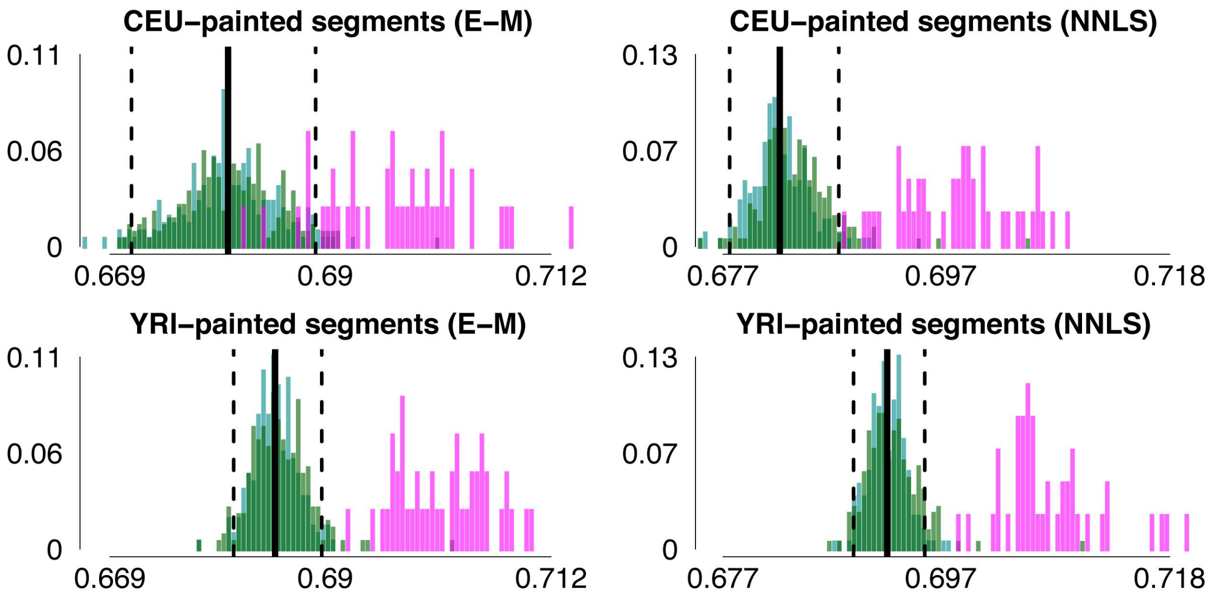
CEU–painted segments (E–M)
CEU–painted segments (NNLS)
YRI–painted segments (E–M)
YRI–painted segments (NNLS)
0.11
0.06
0
0.13
0.07
0
0.669
0.69
0.712
0.677
0.697
0.718
